# Stereospecific Conversion of Boronic Esters into Enones using Methoxyallene: Application in the Total Synthesis of 10‐Deoxymethynolide

**DOI:** 10.1002/anie.202312054

**Published:** 2023-11-09

**Authors:** Kristian J. Chambers, Patthadon Sanghong, Daniel Carter Martos, Giorgia Casoni, Rory C. Mykura, Durga Prasad Hari, Adam Noble, Varinder K. Aggarwal

**Affiliations:** ^1^ School of Chemistry University of Bristol Cantock's Close BS8 1TS Bristol UK

**Keywords:** Enones, Lithiation-Borylation, Organoboron Compounds, Stereospecificity, Total Synthesis

## Abstract

Enones are widely utilized linchpin functional groups in chemical synthesis and molecular biology. We herein report the direct conversion of boronic esters into enones using commercially available methoxyallene as a three‐carbon building block. Following boronate complex formation by reaction of the boronic ester with lithiated‐methoxyallene, protonation triggers a stereospecific 1,2‐migration before oxidation generates the enone. The protocol shows broad substrate scope and complete enantiospecificity is observed with chiral migrating groups. In addition, various electrophiles could be used to induce 1,2‐migration and give a much broader range of α‐functionalized enones. Finally, the methodology was applied to a 14‐step synthesis of the enone‐containing polyketide 10‐deoxymethynolide.

## Graphical abstract

A one‐pot strategy for the stereospecific conversion of boronic esters into enones has been developed using methoxyallene as a commercially available, three‐carbon building block. The methodology is suitable for primary, secondary, and tertiary boronic esters and has been applied to a 14‐step total synthesis of the polyketide 10‐deoxymethynolide.

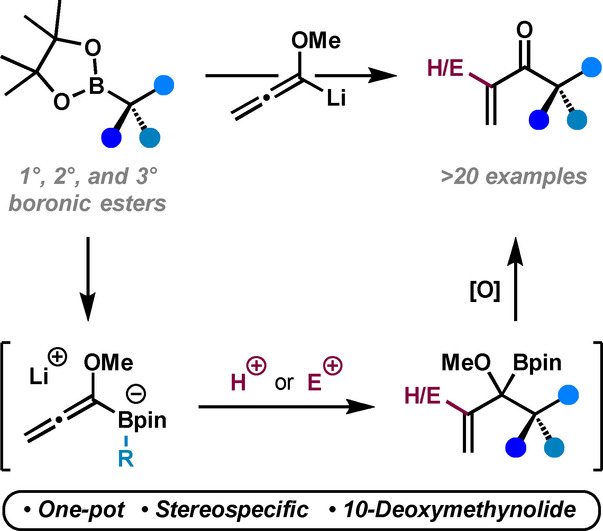

Enones (α,β ‐Unsaturated ketones) are widely used linchpin functional groups due to their ability to undergo a myriad of transformations, including 1,2‐additions, conjugate additions, and Diels–Alder reactions.^[1]^ Beyond their synthetic utility, enones are common features of many natural products and bioactive compounds, including dexamethasone,^[2]^ adenanthin,^[3]^ and hypothemycin (Figure 1a).^[4]^ Enones are potent Michael acceptors that react readily with a broad range of nucleophiles, which is often a key mechanism for exerting their biological effect. For example, reaction with cysteine residues when bound in the active site of proteins is a process that is commonly exploited in protein kinase inhibitors, where enones act as covalent warheads targeting the cysteinome (Figure 1b).^[5]^ This ability to react with nucleophilic sites in target proteins has led to the enone moiety garnering significant attention in medicinal chemistry.^[6]^ In addition, enones have been used for site selective PEGylation of proteins, technology which has become of increasing importance in commercially available pharmaceuticals, including its application in the preparation of CoVid vaccines (Figure 1b).^[7]^ Hence, strategies by which enones may be conveniently accessed remain in demand, since they provide the potential for more direct, and therefore efficient syntheses of these important molecules from readily available precursors.^[8]^

**Figure 1:**
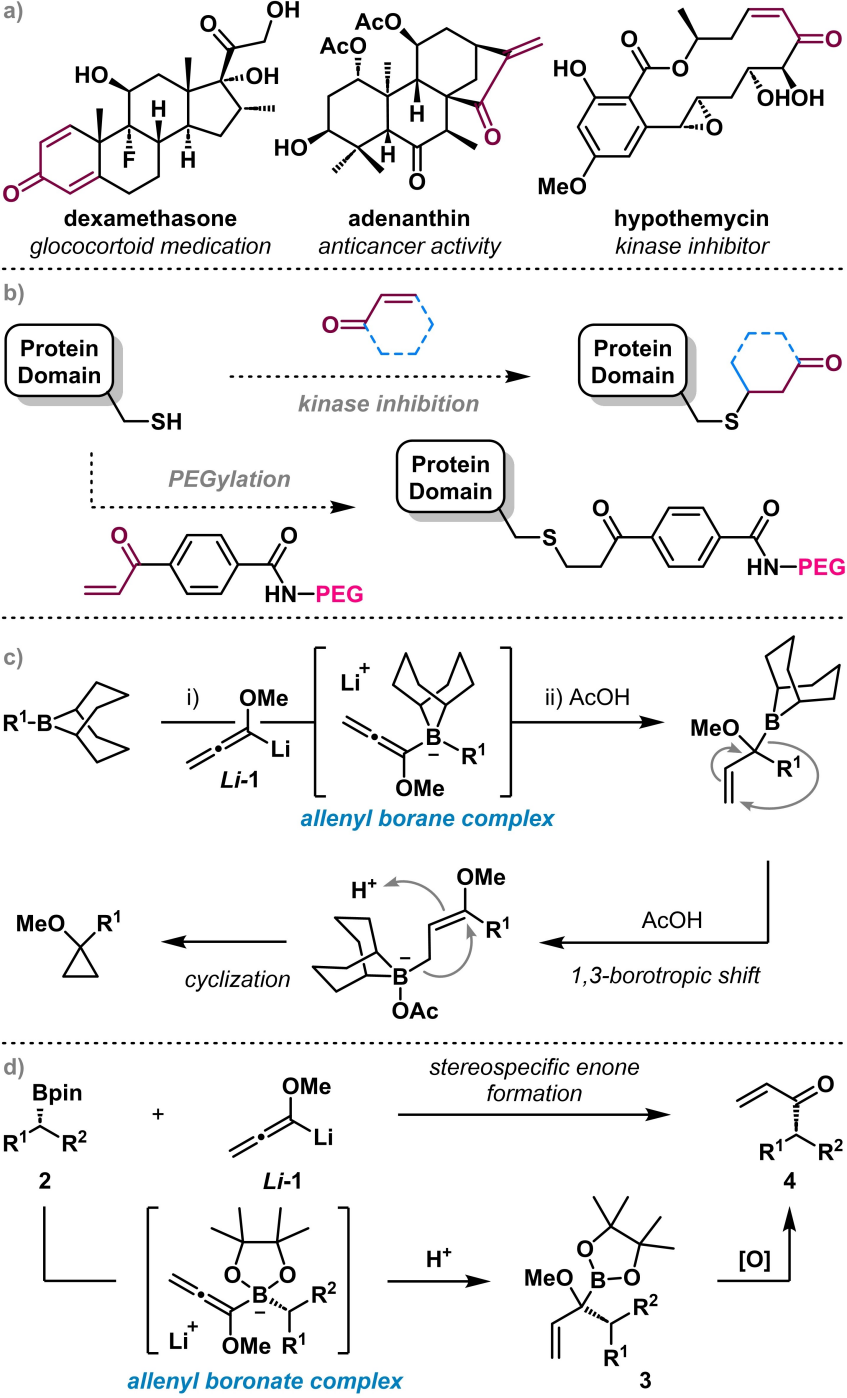
a) Examples of bioactive compounds containing enones. b) Mode of action of enones as covalent protein inhibitors and as linkers for PEGylation. c) Synthesis of alkoxycyclopropanes from allenyl borane complexes by Suzuki and co‐workers. d) Synthesis of terminal enones from boronic esters via allenyl boronate complexes (this work).

Owing to their inherent stability and low toxicity, boronic esters have found widespread application in synthetic organic chemistry.^[9]^ A significant aspect of this application has been in the use of boronic esters as versatile tools for asymmetric synthesis,^[10]^ since they can be readily obtained in high enantioselectivity^[11]^ and subsequently transformed stereospecifically into other functional groups.[^10^, ^12^] We were attracted to expanding the suite of stereospecific boronic ester functionalizations to include direct conversion into enones, thus providing a valuable method to access chiral variants of these synthetically useful molecules.

We envisioned adapting a Zweifel‐type reaction to access the enone functional group utilizing alkoxyallenes as three‐carbon building blocks. This approach is related to work by Suzuki, who reacted methoxyallenyl lithium ***Li*** **‐1** with alkyl boranes to form allenyl borane complexes (Figure 1c). Subsequent proton‐mediated 1,2‐migration gave intermediate allylic boranes, which underwent rapid 1,3‐borotropic shift^[13]^ followed by protonation and cyclization to give methoxycyclopropanes.^[14]^ In order to access enones we needed to avoid the 1,3‐borotropic shift, which could potentially be achieved by simply substituting the borane for a boronic ester, since boronic esters are considerably less prone to 1,3‐borotropic shifts.^[15]^ Thus, we reasoned that following reaction of methoxyallenyl lithium ***Li*** **‐1** with a boronic ester **2** to generate an allenyl boronate complex, protonation should trigger a 1,2‐migration to give the allylic boronic ester intermediate **3** (Figure 1d).^[12a]^ Conversion of **3** to the desired enone **4** could be achieved by oxidation of the boronic ester and collapse of the resulting hemiacetal. We were mindful of the difficulty attributed to this final oxidation, as enones are themselves sensitive to oxidation, particularly by the nucleophilic oxidants that are commonly employed for boronic ester oxidation (e.g., peroxides), and so particular care would have to be taken to minimize product destruction.

At the outset of our studies, we elected to use commercially available methoxyallene (**1**)^[16]^ as a three‐carbon building block and phenethyl pinacol boronic ester **2 a** as our model substrate (Table 1). After optimization, we found that reaction of allenyl lithium ***Li*** **‐1** with boronic ester **2 a** at −40 °C in THF, followed by addition of AcOH at −78 °C, and subsequent oxidation of the resulting allylic boronic ester **3 a** with NaBO_3_ ⋅ 4H_2_O gave enone **4 a** in 89 % yield (entry 1). Temperature was found to play an important role, with decreased yields obtained when acetic acid was added at higher temperatures than −78 °C (entries 2, 3, and 4). Weaker proton sources (MeOH, HFIP) could also promote the 1,2‐migration but were found to give reduced yields in comparison to glacial acetic acid (entries 5 and 6). Standard basic hydrogen peroxide oxidation conditions (NaOH/H_2_O_2_) led to rapid (ca. 30 min) and significant over‐oxidation of the desired enone, affording a mixture of **4 a** and epoxyketone (entry 7). The use of the milder oxidant NaBO_3_ ⋅ 4H_2_O was much more effective, since it prevented over‐oxidation, although reducing the stoichiometry from 2 to 1 equivalent gave reduced yields (entry 8).

**Table 1:** Optimization of the synthesis of enones from boronic esters.

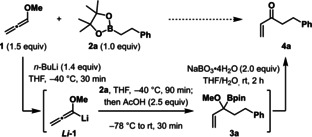		
Entry^[a]^	Change from standard conditions	Enone **4a** yield [%]^[a]^
**1**	None	89
**2**	AcOH addition at −40 °C	80
**3**	AcOH addition at 0 °C	74
**4**	AcOH addition at rt	67
**5**	MeOH instead of AcOH	28
**6**	HFIP instead of AcOH	58
**7**	2 equiv H_2_O_2_ oxidation	47 (45 % epoxyketone)
**8**	1 equiv NaBO_3_ ⋅ 4H_2_O	71

[a] Yields determined by analysis of quantitative ^1^H NMR spectra against an internal standard.

We then turned our attention to the scope of boronic esters that could be used in the transformation (Scheme 1a). Pleasingly, we found that all primary boronic esters smoothly converted to the desired enones **4 a**–**4 g** in moderate to good yields. Whilst secondary and tertiary boronic esters were successfully converted to the allyl boronic ester intermediate **3**, these more sterically encumbered substrates led to slower oxidation of the boronic ester, which resulted in competing over‐oxidation of the enone. To address this issue, further optimization of the oxidation was conducted, and two more sets of conditions were developed that minimized over‐oxidation: (i) for secondary boronic esters: NaBO_3_ ⋅ 4H_2_O (1.1 eq) at 50 °C; (ii) for hindered secondary and tertiary boronic esters: NaOH/H_2_O_2_ at RT. With these conditions in hand, both secondary and tertiary boronic esters could be converted to the desired enones **4 h**–**4 t** in moderate to good yields. Furthermore, good functional group tolerance was demonstrated with aryl and alkyl halides (**4 h** and **4 l**), nitriles (**4 j**), protected amines (**4 o**), protected alcohols (**4 n**), and azetidines (**4 s**) all tolerating the reaction conditions. The reaction could also be used for the formation of complex natural product derivatives (**4 k**, **4 l**, and **4 m**). Finally, the use of diastereo‐ and enantioenriched secondary and tertiary substrates (**4 i**–**4 n**, **4 q**, **4 r** and **4 t**) demonstrated that the reaction was completely stereospecific.

**Scheme 1:**
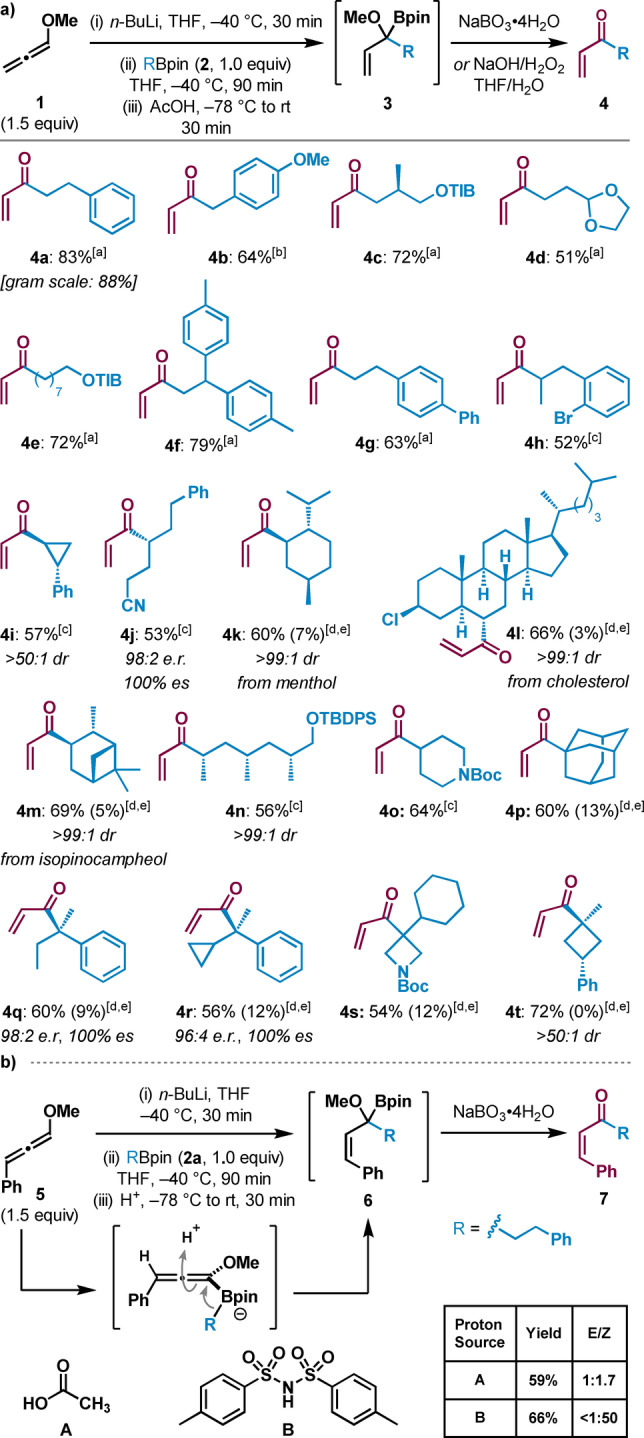
a) Substrate scope of the one‐pot synthesis of α,β‐unsaturated ketones from boronic esters. Reaction conditions: methoxyallene (**1**; 0.38 mmol), *n*‐BuLi (0.35 mmol), THF (3.0 mL), −40 °C, then alkyl boronic ester (0.25 mmol), THF (0.3 mL) at −40 °C, then AcOH (0.63 mmol) at −78 °C, then RT for 30 min. Oxidation conditions: [a] NaBO_3_ ⋅ 4H_2_O (0.5 mmol, 2.0 equiv) at RT for 2 h; [b] oxidation performed with NaBO_3_ ⋅ 4H_2_O (1.1 equiv) at RT for 2 h; [c] NaBO_3_ ⋅ 4H_2_O (0.28 mmol, 1.1 equiv) at 50 °C for 16 h; [d] NaOH (0.75 mmol, 3.0 eq) and H_2_O_2_ (1.5 mmol, 6.0 equiv) at RT. [e] The yield in parentheses is for the epoxyketone formed by oxidation of **4**. TIB=2,4,6‐triisopropylbenzoyl. b) Application of substituted methoxyallenes and the E/Z selectivity observed when using acetic acid (**A**) and 4‐methyl‐*N*‐tosylbenzenesulfonamide (**B**) as a proton source.

The methodology could be further extended to substituted methoxyallenes (Scheme 1b). Application of phenyl substituted allene^[17]^ **5** led to an approximately 1 : 1 mixture of E/Z enones in moderate yield under our standard conditions. Recognizing that approach of a proton source to the two faces of the allene would encounter quite different steric environments, we tested the bulkier acid 4‐methyl‐*N*‐tosylbenzenesulfonamide (**B**) and were pleased to find that the Z enone was formed with >50 : 1 selectivity.

We recognized that triggering the 1,2‐migration of the allenyl boronate complex intermediate with alternative electrophiles instead of a proton source could significantly expand the scope of the reaction and enable access to a much broader range of densely functionalized enones. α‐Substituted enones are privileged scaffolds in organic chemistry owing to their diverse reactivities and potential therapeutic applications.^[18]^ However, routes for accessing these products frequently require multiple steps and/or pre‐functionalization of starting materials.^[19]^ Therefore, we explored the addition of a variety of electrophiles after formation of the allenyl boronate complex (Scheme 2a). Eschenmoser's salt proved to be an effective electrophile in this reaction, allowing for the introduction of an amine functionality into enone **8 a** in 68 % yield. Cationic carbon‐based electrophiles such as tropylium and benzodithiolylium were also found to promote 1,2‐migration, affording α‐substituted enones **8 b** and **8 c** in 64 % and 48 % yields, respectively. Application of phenylsulfenyl chloride and phenylselenyl chloride gave enones **8 d** and **8 e** in moderate yields. Selectfluor could also be used to afford fluorinated enone **8 f**. Finally, it was found that the use of iodine and base gave the iodo acetal **9** (Scheme 2b), which results from Zweifel olefination^[20]^ followed by iodomethoxylation of the resulting methoxyallene intermediate. Pleasingly, **9** could be converted into iodoenone **8 g** by acid‐mediated hydrolysis of the dimethyl acetal. Alternatively, elimination of the iodide from **9** gave terminal alkyne **10**, which could then be hydrolyzed to ynone **11** in excellent yield. Alkyne **10** was also found to undergo copper‐catalyzed hydroboration^[21]^ to afford an unstable alkenyl boronic ester (74 % NMR yield, see Supporting Information for details) that could be engaged in a subsequent Suzuki coupling^[22]^ to give acetal **12** in 49 % yield over two steps.

**Scheme 2:**
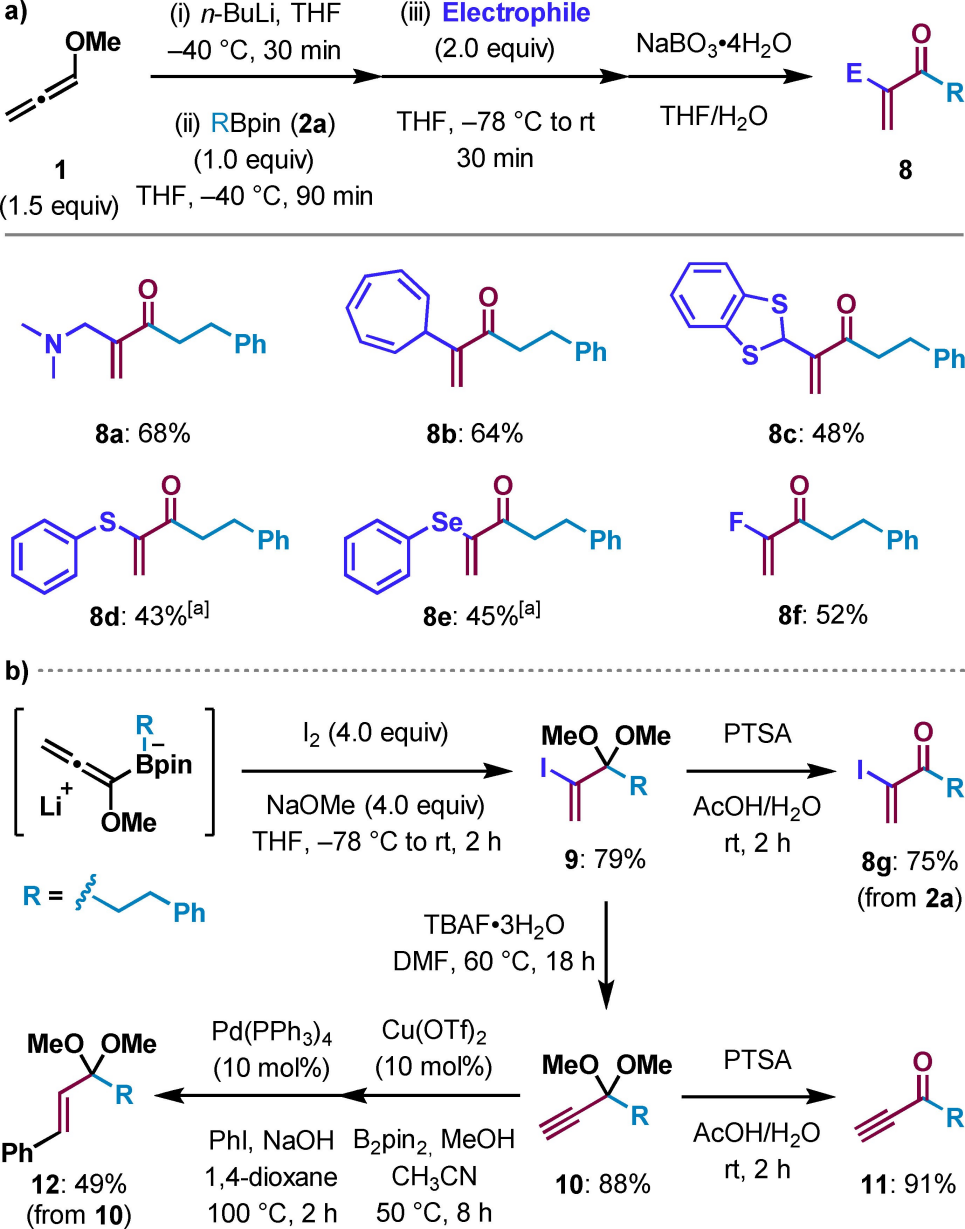
a) Synthesis of α‐substituted enones from boronic esters through variation of electrophiles. b) Use of I_2_ as an electrophile and subsequent transformations. [a] Yields given are ^1^H NMR yields owing to the products being unstable to column chromatography. PTSA=*p*‐toluenesulfonic acid.

We next sought to demonstrate the applicability of this enone synthesis in the total synthesis of the polyketide macrolide 10‐deoxymethynolide.^[23]^ Bearing four stereocenters and an internal enone, this particular target would provide an ideal test for this methodology in tandem with other lithiation‐borylation methodologies (Scheme 3a).^[24]^ 10‐Deoxymethynolide has been a popular target in total synthesis and previous work was used to guide the endgame of our synthesis,^[25]^ in particular, Kang and co‐workers’ use of a Yamaguchi esterification followed by a ring closing metathesis (RCM).^[25c]^ Starting from the enantioenriched commercially available (*S*)‐Roche ester (**13**), TBS protection and subsequent reduction of **14** with DIBAL‐H afforded alcohol **15**, which was then converted to benzoate **16** via a Mitsunobu reaction.^[24]^ This set the stage for the first stereoselective lithiation‐borylation reaction, which used (dimethylphenylsilyl)boronic acid pinacol ester (PhMe_2_SiBpin) to furnish boronic ester **17** with the dimethylphenyl silyl group installed as a masked hydroxy group. Treatment of the resulting stereochemically pure boronic ester **17** with lithiated carbenoid ***Li‐*** **18** (generated in situ from *n*‐BuLi and stannane **18**) led to formation of the intermediate boronate complex **19**, which upon warming to room temperature underwent a 1,2‐metallate rearrangement to give boronic ester **20**. To our surprise, this was accompanied by the formation of significant quantities of over homologated product **20 b** (ca. 30–50 %; Scheme 3b). Over homologation is rare but can occur if 1,2‐metallate rearrangement is rapid and begins before excess lithiated TIB ester ***Li*** **‐18** has fully decomposed, which typically occurs at temperatures above approximately −50 °C.^[26]^ For boronic ester **17**, it was suspected that these two processes (1,2‐metallate rearrangement and carbenoid decomposition) overlapped, leading to over homologation of the desired product. To address this, we sought to trap excess ***Li*** **‐18** with an electrophile, and therefore added allyl bromide at −78 °C after complete boronate complex formation.^[27]^ Using this modification, over homologation was prevented and boronic ester **20** was isolated in 86 % yield. The resulting boronic ester **20** could then undergo further sequential homologations with lithiated carbenoids **21** and ***Li*** **‐18**, growing the lipophilic chain with precise reagent‐mediated stereocontrol and yielding the secondary boronic ester **22** as a single stereoisomer. From here, we applied our new methodology to install the enone. Thus, treatment with lithiated methoxyallene, followed by AcOH and sodium perborate gave terminal enone **24** in 65 % yield, demonstrating the envisioned generality of the disclosed methodology. Deprotection of the TBS group with TBAF/AcOH gave alcohol **25** which was subjected to sequential Dess–Martin periodinane and Pinnick oxidations to afford carboxylic acid **26** in 80 % yield over two steps. Coupling with the homoallylic alcohol **27** ^[25c]^ through a modified Yamaguchi esterification then allowed for closure of the 12‐membered ring by a Grubbs II ring‐closing metathesis reaction to give macrolide **29** in 84 %.^[25c]^ Finally, conversion of the phenyldimethylsilyl group to a hydroxyl group was all that remained to complete the synthesis of 10‐deoxymethynolide. Although such transformations have been reported in the presence of an enone, they are often low yielding,^[28]^ which is presumably due to competing oxidation of the enone. Nevertheless, using Hg(II)‐mediated conditions for proto‐desilylation of the phenyl ring, followed by oxidation with peracetic acid,^[29]^ gave 10‐deoxymethynolide (**30**) in 34 % yield, completing to the total synthesis in 14 steps longest linear sequence.

**Scheme 3:**
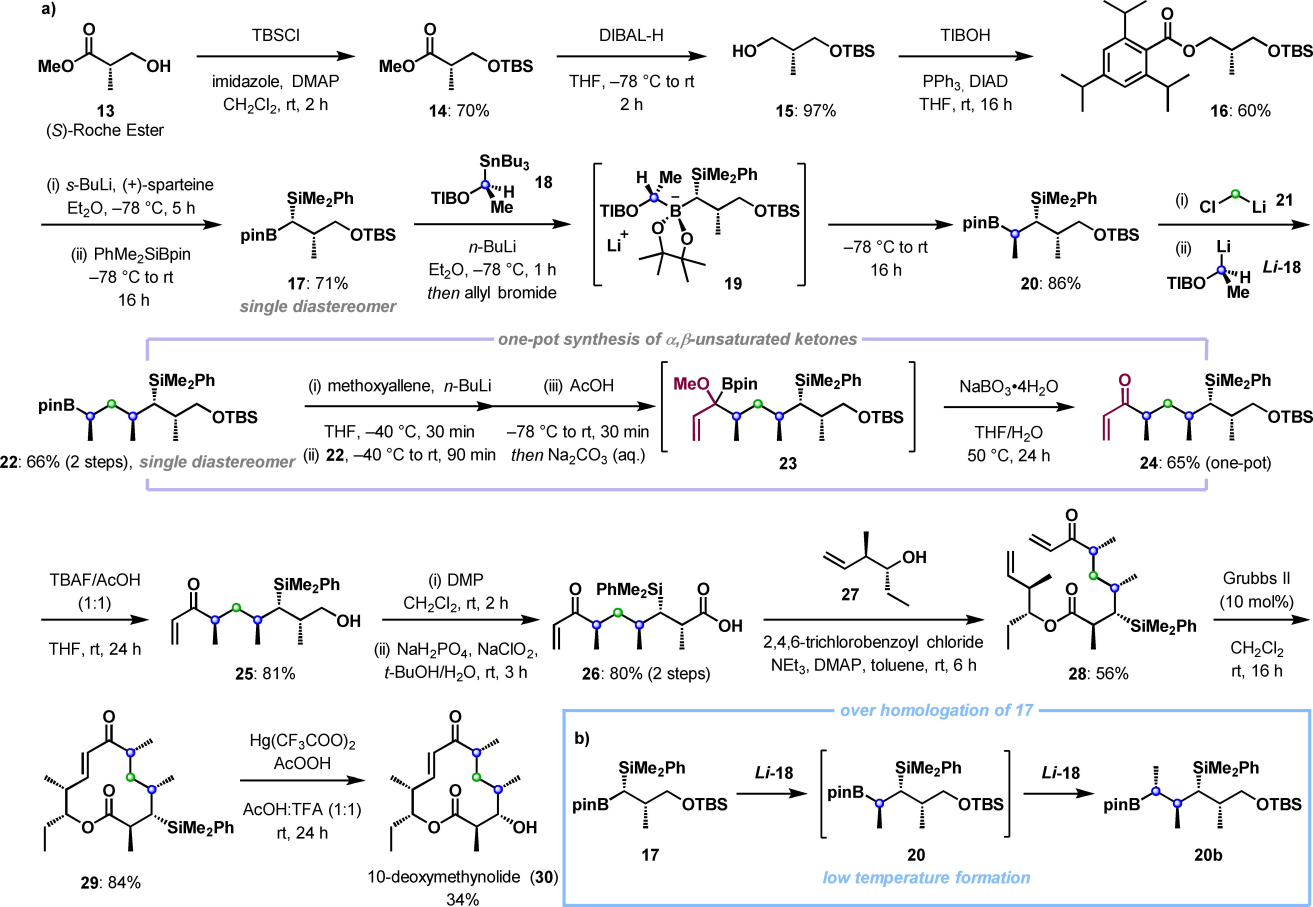
a) Total synthesis of 10‐deoxymethynolide utilising a one‐pot synthesis of enones from boronic esters in cooperation with lithiation‐borylation methodology. b) Observation of over homologation at low temperature. DIBAL‐H=diisobutylaluminium hydride, TIB=2,4,6‐triisopropylbenzoyl, DIAD=diisopropyl azodicarboxylate, DMAP=4‐dimethylaminopyridine.

In conclusion, we have developed an efficient one‐pot protocol for the conversion of boronic esters to the highly desirable enone functional group. This process uses methoxyallene as a commercially available building block, and can be applied to primary, secondary, and tertiary boronic esters in moderate to excellent yields and with complete enantiospecificity. In addition to a proton‐mediated reaction for the formation of mono‐substituted enones, the intermediate allenyl boronate complex can react with a range of electrophiles to give α‐substituted enones, significantly expanding the scope of the overall process. Finally, the utility of this methodology was demonstrated in a 14‐step total synthesis of the enone‐containing polyketide 10‐deoxymethynolide.

## Conflict of interest

The authors declare no conflict of interest.

## Supporting information

As a service to our authors and readers, this journal provides supporting information supplied by the authors. Such materials are peer reviewed and may be re‐organized for online delivery, but are not copy‐edited or typeset. Technical support issues arising from supporting information (other than missing files) should be addressed to the authors.

Supporting Information

## Data Availability

The data that support the findings of this study are available in the supplementary material of this article.
