# Supplementary material for: Stereospecific Conversion of Boronic Esters into Enones using Methoxyallene: Application in the Total Synthesis of 10‐Deoxymethynolide
Source: Angew Chem Int Ed Engl. 2023 Nov 9;62(50):e202312054. doi: 10.1002/anie.202312054 (PMC10953306; doi:10.1002/anie.202312054)

Angewandte Chemie
Eine Zeitschrift der Gesellschaft Deutscher Chemiker

Supporting Information

Stereospecific Conversion of Boronic Esters into Enones using Methoxyallene: Application in the Total Synthesis of 10-Deoxymethynolide

*K. J. Chambers, P. Sanghong, D. Carter Martos, G. Casoni, R. C. Mykura, D. Prasad Hari, A. Noble, V. K. Aggarwal**

TABLE OF CONTENTS

LIST OF SUPPLEMENTARY SCHEMES, FIGURES AND TABLES 2

LIST OF CHARACTERISED PRODUCTS 2

1. MATERIALS AND GENERAL METHODS 4

1.1. Glassware, Solvents and Reagents 4

1.2. Chromatography and Instrumentation 4

1.3. Naming of Compounds 4

2. EXPERIMENTAL DATA 5

2.1. Synthesis of Boronic Esters and Starting Materials 5

2.2. General Procedures 11

2.2.1. General Procedure 1: Synthesis of Terminal Enones from Boronic Esters 11

2.2.2. Oxidation Conditions 1a: Primary Boronic Esters 11

2.2.3. Oxidation Conditions 1b: Secondary Boronic Esters 11

2.2.4. Oxidation Conditions 1c: Sterically Encumbered Secondary and Tertiary Boronic Esters 11

2.3. Synthesis of Terminal Enones from Boronic Esters 13

2.4. Synthesis of α-Substituted Enones from Boronic Esters 31

2.4.1. General Procedure 2: Synthesis of α-Substituted Enones from Boronic Esters 31

2.5. Total Synthesis of 10-Deoxymethynolide 40

3. REFERENCES 55

4. SPECTROSCOPIC DATA 56

LIST OF SUPPLEMENTARY SCHEMES, FIGURES AND TABLES

Figure 1: Synthesis of starting boronic esters 5

LIST OF CHARACTERISED PRODUCTS

1-Methoxypropa-1,2-diene (Methoxyallene (**1**)) 6
4,4,4,5-Tetramethyl-2-phenethyl-1,3,2-dioxaborolane (**2a**) 7
Synthesis of (1*S*, 2*R*, 4*R*)-2-chloro-1-isopropyl-4-methylcyclohexane (**SI-2**) 8
Synthesis of 2-((1*R*, 2*R*, 5*R*)-2-isopropyl-5-methylcyclohexyl)-4,4,5,5-tetramethyl-1,3,2-dioxaborolane (**B11**). 9
Synthesis of (3-methoxyprop-1-yn-1-yl)benzene (**SI-3**) 10
5-phenyl-1-penten-3-one (**4a**) 13
1-(4-methoxyphenyl)but-3-en-2-one (**4b**) 13
(*R*)-2-methyl-4-oxohex-5-en-1-yl 2,4,6-triisopropylbenzoate (**4c**) 14
5-(1,3-dioxolan-2-yl)pent-1-en-3-one (**4d**) 15
9-oxoundec-10-en-1-yl 2,4,6-triisopropylbenzoate (**4e**) 15
5,5-di-*p*-tolylpent-1-en-3-one (**4f**) 16
5-([1,1'-biphenyl]-4-yl)pent-1-en-3-one (**4g**) 16
5-(2-bromophenyl)-4-methylpent-1-en-3-one (**4h**) 17
1-((1*S*,2*S*)-2-phenylcyclopropyl)prop-2-en-1-one (**4i**) 18
5-oxo-4-phenethylhept-6-enenitrile (**4j**) 18
1-((1*S*,2*S*,5*R*)-2-isopropyl-5-methylcyclohexyl)prop-2-en-1-one (**4k**) 20
1-((3*S*,5*R*,6*S*,8*S*,9*S*,10*R*,13*R*,14*S*,17*R*)-3-chloro-10,13-dimethyl-17-((*R*)-6-methylheptan-2-yl)hexadecahydro-1H-cyclopenta[a]phenanthren-6-yl)prop-2-en-1-one (**4l**) 21
1-((2*R*,3*R*)-2,6,6-trimethylbicyclo[3.1.1]heptan-3-yl)prop-2-en-1-one (**4m**) 22
(4*R*,6*R*,8*S*)-9-((tert-butyldiphenylsilyl)oxy)-4,6,8-trimethylnon-1-en-3-one (**4n**) 22
tert-butyl 4-acryloylpiperidine-1-carboxylate (**4o**) 23
1-((3*r*,5*r*,7*r*)-adamantan-1-yl)prop-2-en-1-one (**4p**) 24
(*R*)-4-methyl-4-phenylhex-1-en-3-one (**4q**) 24
(*R*)-4-cyclopropyl-4-phenylpent-1-en-3-one (**4r**) 25
tert-butyl 3-acryloyl-3-cyclohexylazetidine-1-carboxylate (**4s**) 27
1-(1-methyl-3-phenylcyclobutyl)prop-2-en-1-one (**4t**) 27
Synthesis of (Z)-1,5-diphenylpent-1-en-3-one (**7**) 29
2-((dimethylamino)methyl)-5-phenylpent-1-en-3-one (**8a**) 32
2-(cyclohepta-2,4,6-trien-1-yl)-5-phenylpent-1-en-3-one (**8b**) 32
2-(benzo[d][1,3]dithiol-2-yl)-5-phenylpent-1-en-3-one (**8c**) 33
5-phenyl-2-(phenylthio)pent-1-en-3-one (**8d**) 34
5-phenyl-2-(phenylselanyl)pent-1-en-3-one (**8e**) 34
2-fluoro-5-phenylpent-1-en-3-one (**8f**) 35
2-iodo-5-phenylpent-1-en-3-one (**8g**) 35
(4-iodo-3,3-dimethoxypent-4-en-1-yl)benzene (**9**) 36
(3,3-dimethoxypent-4-yn-1-yl)benzene (**10**) 37
5-phenylpent-1-yn-3-one (**11**) 37
((E)-(3,3-dimethoxypent-1-ene-1,5-diyl)dibenzene (**12**) 38

methyl (*S*)-3-hydroxy-2-methylpropanoate (**14**) 40
(*R*)-2-methyl-3-((trimethylsilyl)oxy)propan-1-ol (**15**) 41
(*R*)-3-((tert-butyldimethylsilyl)oxy)-2-methylpropyl 2,4,6-triisopropylbenzoate (**16**) 42
tert-butyl((2*S*,3*S*)-3-(dimethyl(phenyl)silyl)-2-methyl-3-(4,4,5,5-tetramethyl-1,3,2-dioxaborolan-2-yl)propoxy)dimethylsilane (**17**)........ 43
(*S*)-1-(trimethylstannyl)ethyl 2,4,6-triisopropylbenzoate (**18**) 44
tert-butyl(((2S,4R)-3-(dimethyl(phenyl)silyl)-2-methyl-4-(4,4,5,5-tetramethyl-1,3,2-dioxaborolan-2-yl)pentyl)oxy)dimethylsilane (**20**) 46
tert-butyl(((2S,3S,4S,6R)-3-(dimethyl(phenyl)silyl)-2,4-dimethyl-6-(4,4,5,5-tetramethyl-1,3,2-dioxaborolan-2-yl)heptyl)oxy)dimethylsilane (**22**) 47
(4*R*,6*S*,7*S*,8*S*)-9-((tert-butyldimethylsilyl)oxy)-7-(dimethyl(phenyl)silyl)-4,6,8-trimethylnon-1-en-3-one (**24**) ... 48
(4*R*,6*S*,7*S*,8*S*)-7-(dimethyl(phenyl)silyl)-9-hydroxy-4,6,8-trimethylnon-1-en-3-one (**25**) 49
(2*S*,3*S*,4*S*,6*R*)-3-(dimethyl(phenyl)silyl)-2,4,6-trimethyl-7-oxonon-8-enoic acid (**26**) 50
(3*R*,4*R*)-4-methylhex-5-en-3-yl(2*S*,3*S*,4*S*,6*R*)-3-(dimethyl(phenyl)silyl)-2,4,6-trimethyl-7-oxonon-8-enoate (**28**) 51
(3*S*,4*S*,5*S*,7*R*,11*R*,12*R*,E)-4-(dimethyl(phenyl)silyl)-12-ethyl-3,5,7,11-tetramethyloxacyclododec-9-ene-2,8-dione (**29**)........ 52
10-Deoxymethynolide (**30**) 53

1. MATERIALS AND GENERAL METHODS

1.1. Glassware, Solvents and Reagents

All manipulations were performed with oven-dried (130 °C for a minimum of 12 h) or flame-dried glassware using standard Schlenk techniques under an atmosphere of nitrogen, unless otherwise stated.

All anhydrous solvents were commercially supplied or dried using an Anhydrous Engineering alumina column drying system (THF, toluene, Et_2O, CH_2Cl_2) and stored over 3 Å mol sieves. Reagents were purchased from commercial sources and used as received. ***Exceptions***: Methoxyallene (**1**) (when purchased) and *N, N, N', N',*-tetramethylethylenediamine (TMEDA) were distilled under an inert atmosphere at standard pressure. Methoxyallene (**1**) was purchased from Sigma Aldrich. Alternatively, methoxyallene (**1**) was also be prepared in a single step from methyl propargyl ether. No impact on the effectiveness of the reactions was noted by the authors in either case. *n*-Butyllithium (1.6 M in hexanes) and *s*-butyllithium (1.3 M in cyclohexane/hexane (92/8)) were purchased from Acros organics and titrated against N-benzylbenzamide prior to use.

1.2. Chromatography and Instrumentation

Thin layer chromatography (TLC) was performed using Merck Kieselgel 60 F254 fluorescent treated silica, which was visualised under UV light, or by staining with aqueous basic potassium permanganate followed by heating, *p*-anisaldehyde solution followed by heating, Hanessian's stain (CAM stain) followed by heating.

Flash column chromatography (FCC) was carried out using Sigma-Aldrich silica gel (60 Å, 230–400 mesh, 40–63 μm) or a Biotage Isolera™ flash purification system. In cases where automated column chromatography was employed the solvent gradient and flow rate are indicated.

NMR spectra were recorded at various field strengths, as indicated, using Bruker 400 MHz, Varian VNMR 400 MHz, Varian VNMR 500 MHz, or Bruker Cryo 500 MHz for ^{1}H, ^{11}B, ^{13}C and ^{19}F acquisitions. All NMR spectra were recorder at 25 °C unless otherwise stated. Chemical shifts (δ) are reported in parts per million (ppm) and referenced $CDCl_3$ (^{1}H: 7.26 ppm; ^{13}C: 77.0 ppm). Coupling constants (*J*) are given in Hertz (Hz) and refer to apparent multiplicities (s = singlet, d = doublet, t = triplet, q = quartet, quin = quintet, sex = sextet, h = heptet, m = multiplet, br = broad signal, dd = doublet of doublets, etc.). The ^{1}H NMR spectra are reported as follows: chemical shift (multiplicity, coupling constants, number of protons)

High resolution mass spectra (HRMS) were recorded on a Bruker Daltonics MicrOTOF II by Electrospray Ionisation (ESI); a Thermo Scientific QExactive by Electron Ionisation (EI); a Thermo Scientific Orbitrap Elite by ESI or Atmospheric Pressure Chemical Ionisation (APCI); or a Bruker UltrafleXtreme by Matrix-assisted Laser Desorption/Ionisation (MALDI).

IR spectra were recorded neat as a thin film on a Perkin Elmer Spectrum One FT-IR. Selected absorption maxima (ν_{max}) are reported in wavenumbers (cm^{-1}).

1.3. Naming of Compounds

Compound names are those generated by ChemDraw Professional 20.0 software (PerkinElmer), following the IUPAC nomenclature.

2. EXPERIMENTAL DATA

2.1. Synthesis of Boronic Esters and Starting Materials

Boronic esters were prepared according to literature procedures[1-14] or purchased from suppliers indicated in Figure **1**.

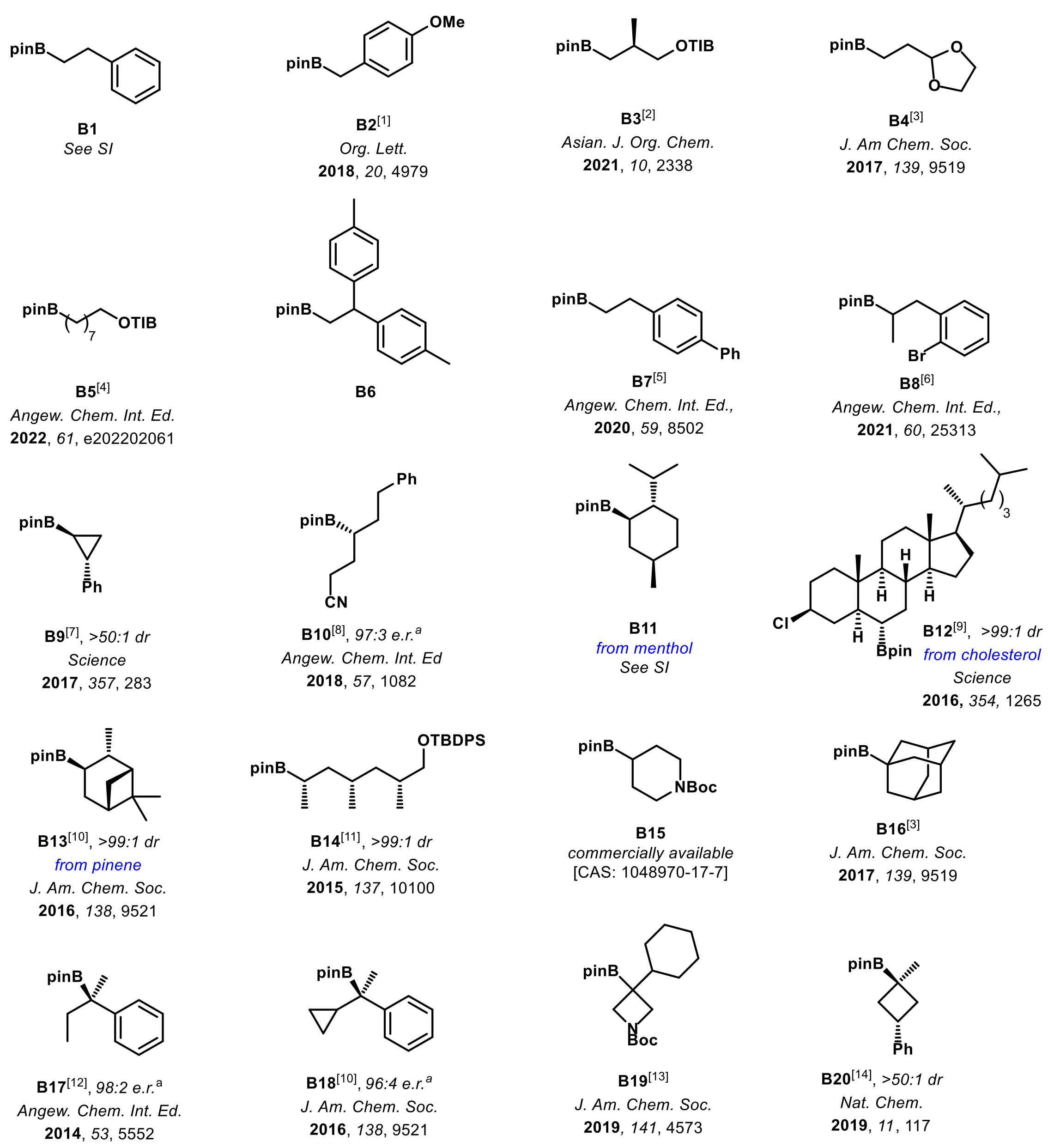

Figure 1: Synthesis of starting boronic esters

[a]Enantiomeric ratio (e.r.) determined by chiral HPLC traces of the oxidized parent boronic ester.

Ethyl 2,4,6-triisopropylbenzoate (**SI-1)** was prepared according to the following literature procedure.[15]

(3R,4R)-4-methylhex-5-en-3-ol (27) was prepared according to the procedure outlined by Brown and co-workers from (−)-B-methoxydiisopinocampheylborane (Ipc_2BOMe) and purified by kugelrohr distillation (120 °C, 100 mbar).[16]

1-Methoxypropa-1,2-diene (Methoxyallene (1))

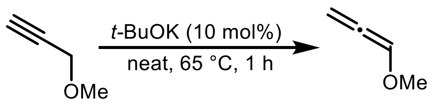

1

Prepared according to a modified literature procedure.[17]

To a round bottom flask, *t*-BuOK (0.73 g, 6.5 mmol, 10 mol%) was added to neat 3-methoxyprop-1-yne (5.5 mL, 65 mmol, 1 equiv.). A condenser was attached, and the resulting suspension was refluxed (65 °C, 1 h) under the pressure of nitrogen. The reaction was cooled to room temperature and a receiver flask attached to the condenser. The receiver flask was submerged in an acetone/dry ice bath (−78 °C) and the crude product distilled (60 °C) to afford the purified 1-methoxypropa-1,2-diene (**1**) (3.22 g, 71%).

R*f* = n.d. (volatile product)

^{1}H and ^{13}C NMR data in accordance with the literature.[18]

^{1}H NMR (400 MHz, Chloroform-*d*) δ_H 6.76 (t, *J* = 5.9 Hz, 1H), 5.47 (d, *J* = 5.9 Hz, 2H), 3.40 (s, 3H).

^{13}C NMR (101 MHz, Chloroform-*d*) δ_C 201.2, 123.0, 91.4, 56.0.

N.B. Methoxyallene (**1**) can also be purchased ([CAS: **13169-00-1**]) and should be distilled prior to use. The authors utilized both methods during this study and observed no impact on the methodology. Methoxyallene can be stored for up to 3 months in a freezer under a blanket of N_2, after this time the authors observed a reduction in reaction yields.

4,4,4,5-Tetramethyl-2-phenethyl-1,3,2-dioxaborolane (2a)

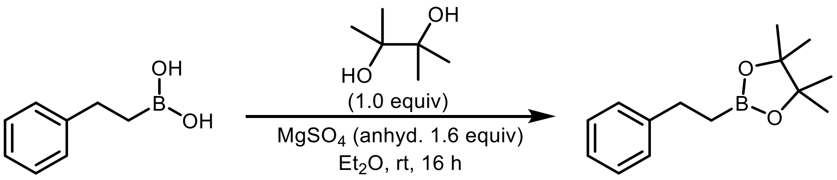

2a

Prepared according to a modified literature procedure.[19]

A two-necked round bottom flask was charged with anhydrous $MgSO_4$ (12.0 g, 99.7 mmol, 1.6 equiv.) and a magnetic follower. The flask was carefully flame dried and cooled under vacuum before evacuating three times with nitrogen. Phenethylboronic acid (9.39 g, 62.6 mmol, 1.0 equiv.) was added to the flask, followed by the addition of pinacol (7.40 g, 62.6 mmol, 1 equiv.) and anhydrous Et_2O (70 mL). The reaction was left to stir (rt, 16 h). The resulting suspension was filtered, and the filter cake washed with Et_2O (3 x 15 mL). The filtrate was concentrated under reduced pressure, followed by purification by column chromatography (10% Et_2O/Pentane). The purified boronic ester **2a** was obtained as a white solid (12.0 g, 83%).

R*f* = 0.54 (50% CH_2Cl_2/Pentane)

^{1}H and ^{13}C NMR data in accordance with the literature.[19]

^{1}H NMR (400 MHz, Chloroform-*d*) δ_H 7.31 – 7.21 (m, 4H), 7.21 – 7.14 (m, 1H), 2.75 (t, *J* = 8.2 Hz, 2H), 1.24 (s, 12H), 1.14 (t, *J* = 8.2 Hz, 2H).

^{13}C NMR (101 MHz, Chloroform-*d*) δ_C 144.5, 128.3, 128.1, 125.6, 83.2, 30.1, 24.9.

Synthesis of (*1S, 2R, 4R*)-2-chloro-1-isopropyl-4-methylcyclohexane (SI-2)

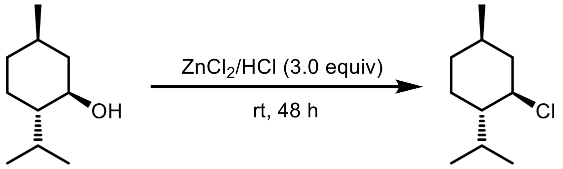

SI-2

Prepared according to a literature procedure.[20]

Concentrated HCl (aq.) (28 mL, 340 mmol, 3.4 equiv.) was cooled (0 °C) in an ice bath. Zinc chloride (41.0 g, 300 mmol, 3.0 equiv.) was added portion wise. Upon observing complete dissolution, the rection was warmed (rt) and (*L)*-menthol (15.6 g, 100 mmol, 1 equiv.) was added in a single portion. The resulting suspension was stirred (rt, 48 h). The reaction was checked for completion by TLC (pentane) which showed a single spot (R_f = 0.60). The aqueous layer was extracted with pentane (50 mL), and the crude organic concentrated under reduced pressure. The crude material was purified by column chromatography (pentane), to obtain the purified (*1S, 2R, 4R*)-2-chloro-1-isopropyl-4-methylcyclohexane (**SI-2**) as a colourless oil (13.8 g, 78%)

R_f = 0.60 (pentane)

^{1}H and ^{13}C NMR data in accordance with the literature.[20]

^{1}H NMR (400 MHz, Chloroform-*d*) δ_H 3.78 (td, *J* = 11.0, 4.1 Hz, 1H), 2.35 (septd, *J* = 7.0, 2.9 Hz, 1H), 2.26 – 2.19 (m, 1H), 1.79 – 1.67 (m, 2H), 1.48 – 1.32 (m, 3H), 1.09 – 0.95 (m, 3H), 0.93 (d, *J* = 6.9 Hz, 3H), 0.91 (d, *J* = 0.7 Hz, 3H), 0.77 (d, *J* = 6.9 Hz, 3H).

^{13}C NMR (101 MHz, Chloroform-*d*) δ_C 64.0, 50.6, 46.9, 34.4, 33.5, 27.3, 24.4, 22.1, 21.1, 15.3.

Synthesis of 2-((1*R*, 2*R*, 5*R*)-2-isopropyl-5-methylcyclohexyl)-4,4,5,5-tetramethyl-1,3,2-dioxaborolane (B11)

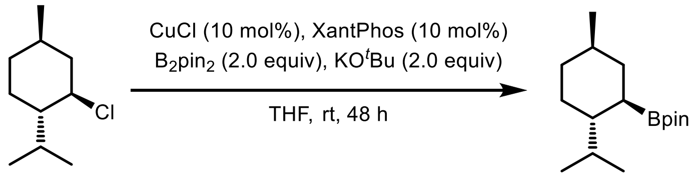

SI-2 **B-11**

Prepared according to a literature procedure.[20]

A flame dried two necked round bottom flask was placed under a nitrogen atmosphere and charged with copper (I) chloride (3.60 g, 3.5 mmol, 10 mol%) and XantPhos (2.01 g, 3.5 mmol, 10 mol%). Anhydrous THF (28 mL) was added, followed by the addition of B_2pin_2 (17.8 g, 70 mmol, 2.0 equiv.). The reaction mixture was subsequently cooled (0 °C) in a water/ice bath, and a 1 M solution of potassium *tert*-butoxide in THF (70 mL, 70 mmol, 2.0 equiv.) was added slowly. The reaction was warmed (rt, 5 min) before returning to the water/ice bath and cooling (0 °C). (*1S, 2R, 4R*)-2-chloro-1-isopropyl-4-methylcyclohexane (**SI-2**) (6.1 g, 35 mmol, 1.0 equiv.) was added dropwise via syringe. The resulting suspension was warmed (rt) and left to stir (48 h). The reaction was checked by taking an aliquot of the reaction and filtering through a plug of silica gel (eluting with Et_2O). A TLC was performed on the resulting solution (100% petroleum ether, R_f = 0.53). Petroleum ether was added to the reaction, and the crude material vacuum filtered through silica gel (10% EtOAc/ petroleum ether). The crude material was concentrated under reduced pressure and purified by flash column chromatography (4% Et_2O/petroleum ether) to boronic ester **B-11** as a colourless liquid (2.48 g, 27%).

$\mathbf{R}_f$ = 0.53 (Petroleum ether)

^{1}H and ^{13}C NMR data in accordance with the literature.[20]

^{1}H NMR (400 MHz, Chloroform-*d*) δ_H 1.76 – 1.56 (m, 4H), 1.44 – 1.17 (m, 14H), 0.98 – 0.86 (m, 7H), 0.84 (d, *J* = 6.7, 3H), 0.76 (d, *J* = 6.9 Hz, 3H).

^{13}C NMR (101 MHz, Chloroform-*d*) δ_C 82.8, 43.9, 37.3, 35.5, 33.6, 32.2, 26.1, 24.9, 24.8, 22.9, 21.8, 16.6.

Synthesis of (3-methoxyprop-1-yn-1-yl)benzene (SI-3)

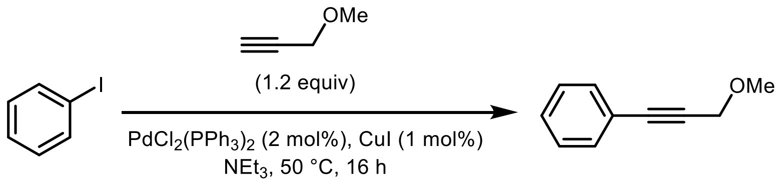

SI-3

Prepared according to a literature procedure.[21]

Methyl propargyl ether (0.51 mL, 6.0 mmol, 1.2 equiv.), iodobenzene (0.56 mL, 5.0 mmol, 1.0 equiv.), and $PdCl_2(PPh_3)_2$ (70.2 mg, 0.1 mmol, 2 mol%) were mixed (5 min, rt) in NEt_3 (20 mL) under a nitrogen atmosphere. CuI (9.5 mg, 0.05 mmol, 1 mol%) was added and the reaction was warmed and stirred (50 °C, 16 h). The reaction was cooled (rt) and filtered. The reaction was concentrated under reduced pressure and the residue purified by column chromatography (BIOTAGE 25 g, 1-10% EtOAc/Pentane) to give alkyne **SI-3** as a yellow oil (0.536 g, 73%).

R*f* = 0.38 (5% EtOAc/pentane)

^{1}H and ^{13}C NMR data in accordance with the literature.[21]

^{1}H NMR (400 MHz, Chloroform-*d*) δ_H δ 7.50 – 7.43 (m, 2H), 7.36 – 7.29 (m, 3H), 4.33 (s, 2H), 3.46 (s, 3H).

^{13}C NMR (101 MHz, Chloroform-*d*) δ_C 131.9 (2 × C), 128.6, 128.4 (2 × C), 122.7, 86.5, 85.0, 60.5, 57.8.

2.2. General Procedures

2.2.1. General Procedure 1: Synthesis of Terminal Enones from Boronic Esters

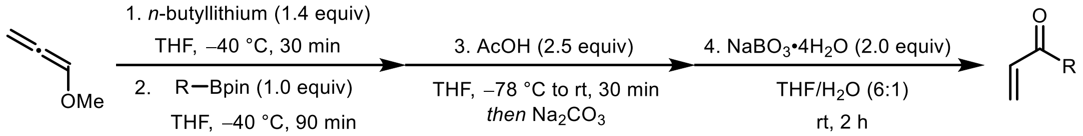

1
(1.5 eq)

To a dried flame Schlenk flask equipped with a magnetic follower under a nitrogen atmosphere, dry THF (2.0 mL) was cooled (−40 °C) using a cryostat. Methoxyallene (**1**) (32 µL, 0.38 mmol 1.5 equiv.) was added and the flask walls washed with dry THF (1.0 mL, to give 3.0 mL total). *n*-Butyllithium (1.6 M in hexanes, 0.35 mmol, 1.4 equiv.) was added dropwise, and the solution stirred (−40 °C, 30 min). Boronic ester (0.25 mmol) was added dropwise to the reaction as a solution in dry THF (0.30 mL) and the flask walls washed with dry THF (0.30 mL, 3.6 mL total). For primary boronic esters, the reaction was stirred (−40 °C, 90 min). For secondary and tertiary boronic esters, the reaction was warmed and stirred (rt, 90 min). In all cases, the reaction was then cooled (−78 °C) and glacial acetic acid (36 µL, 0.63 mmol, 2.5 equiv.) added dropwise. The reaction warmed to room temperature and left to stir (30 min). In all cases the reaction was then quenched with sodium carbonate (sat. aq., 0.6 mL).

2.2.2. Oxidation Conditions 1a: Primary Boronic Esters

Sodium perborate tetrahydrate (77 mg, 0.50 mmol, 2.0 equiv.) was added as a single portion to the reaction. The reaction was allowed to stir (rt, 2 h) before dilution with water (10 mL). The aqueous layer was extracted with diethyl ether (3 × 10 mL) and the combined organic layers dried over $MgSO_4$, filtered, and concentrated under reduced pressure. The crude material was purified by column chromatography.

2.2.3. Oxidation Conditions 1b: Secondary Boronic Esters

Sodium perborate tetrahydrate (42.3 mg, 0.28 mmol, 1.1 equiv.) was added as a single portion to the reaction. The reaction was warmed (50 °C) and vigorously stirred (16 h). The reaction was cooled to room temperature and diluted with water (10 mL). The aqueous layer was extracted with diethyl ether (3 × 10 mL) and the combined organic layers dried over $MgSO_4$, filtered, and concentrated under reduced pressure. The crude material was purified by column chromatography.

2.2.4. Oxidation Conditions 1c: Sterically Encumbered Secondary and Tertiary Boronic Esters

The reaction was cooled (0 °C) in a water/ice bath. Sodium hydroxide (2.0 M, 3.0 equiv.) and hydrogen peroxide (30%, 6.0 equiv.) was added dropwise simultaneously. The reaction was warmed to room temperature and closely followed by TLC. Upon either complete consumption of allylic boronic ester intermediate **3** or observation of epoxide formation, the reaction was quenched with sodium thiosulphate (aq. sat., 10 mL). The aqueous layer was extracted with Et_2O (3 × 10 mL), and the combined layers dried over $MgSO_4$, filtered, and concentrated under reduced pressure. The crude material was purified by column chromatography.

N.B. The authors used *p*-anisaldehyde as the preferred TLC stain. In the experience of the authors, this stain

would generally distinguish the allylic boronic ester intermediates from the enone products by staining the former blue and the latter yellow.

2.3. Synthesis of Terminal Enones from Boronic Esters

5-phenyl-1-penten-3-one (4a)

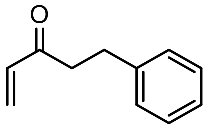

4a

Prepared following **General Procedure 1 and Oxidation Conditions 1a**, using methoxyallene (**1**) (32 μL, 0.38 mmol, 1.5 equiv.), *n*-BuLi (1.6 M in hexanes, 0.35 mmol, 1.4 equiv.), glacial acetic acid (36 μL, 0.63 mmol, 2.5 equiv.), 4,4,5,5,-tetramethyl-2-(2-phenylbutan-2-yl-)-1,3,2-dioxaborolane (**2a**) (58 mg, 0.25 mmol, 1.0 equiv.), and sodium perborate tetrahydrate (77 mg, 0.50 mmol, 2.0 equiv.). Purification by column chromatography (15% CH_2Cl_2/pentane) gave enone **4a** (33.6 mg, 83%) as a colourless oil.

R_f = 0.23 (15% CH_2Cl_2/pentane)

^{1}H and ^{13}C NMR data in accordance with the literature.[22]

^{1}H NMR (400 MHz, Chloroform-*d*) δ_H 7.31 – 7.27 (m, 2H), 7.22 – 7.18 (m, 3H), 6.36 (dd, *J* = 17.8, 10.6 Hz, 1H), 6.22 (dd, *J* = 17.8, 1.2 Hz, 1H), 5.84 (dd, *J* = 10.6, 1.2 Hz, 1H), 2.99 – 2.89 (m, 4H).

^{13}C NMR (101 MHz, Chloroform-*d*): δ_C 200.0, 141.2, 136.6, 128.5, 128.5, 126.4, 126.1, 41.4, 29.9

Gram Scale

Prepared following **General Procedure 1 and Oxidation Conditions 1a** on a 1.0 g scale, using methoxyallene (**1**) (0.54 mL, 6.46 mmol, 1.5 equiv.), *n*-BuLi (1.6 M in hexanes, 6.0 mmol, 1.4 equiv.), glacial acetic acid (0.62 mL, 10.8 mmol, 2.5 equiv.), 4,4,5,5,-tetramethyl-2-(2-phenylbutan-2-yl-)-1,3,2-dioxaborolane (**2a**) (1.0 g, 4.3 mmol, 1.0 equiv.), and sodium perborate tetrahydrate (1.3 g, 8.6 mmol, 2.0 equiv.) in anhydrous THF (60 mL). Purification by column chromatography on an automated system (BIOTAGE 25 g, 12-50% CH_2Cl_2/pentane) gave enone **4a** (608 mg, 88%) as a colourless oil.

1-(4-methoxyphenyl)but-3-en-2-one (4b)

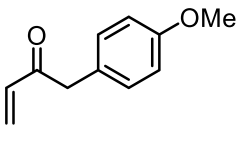

4b

Prepared following **General Procedure 1 and <u>modified</u> Oxidation Conditions 1a**, using methoxyallene (**1**) (32 μL, 0.38 mmol, 1.50 equiv.), *n*-BuLi (1.6 M in hexanes, 0.35 mmol, 1.4 equiv.), glacial acetic acid (36 μL, 0.63 mmol, 2.50 equiv.), 2-(4-methoxybenzyl)-4,4,5,5-tetramethyl-1,3,2-dioxaborolane (62.0 mg, 0.25 mmol, 1.0 equiv.) and sodium perborate tetrahydrate (42.3 mg, 0.28 mmol, <u>1.1 equiv.</u>). Purification by column chromatography (20% Et_2O/pentane) gave enone **4b** (28.5 mg, 65%) as a colourless oil.

R$_f$ = 0.38 (20% Et_2O/pentane)

^{1}H and ^{13}C NMR data in accordance with the literature.[23]

^{1}H NMR (400 MHz, Chloroform-*d*) δ_H 7.12 (d, *J* = 8.7 Hz, 2H), 6.88 (d, *J* = 8.7 Hz, 2H), 6.40 (dd, *J* = 17.6, 10.2 Hz, 1H), 6.29 (dd, *J* = 17.6, 1.5 Hz, 1H), 5.81 (dd, *J* = 10.2, 1.5 Hz, 1H), 3.81 (s, 2H), 3.79 (s, 3H).

^{13}C NMR (101 MHz, Chloroform-*d*) δ_C 198.2, 158.8, 135.7, 130.6, 129.0, 126.1, 114.3, 55.4, 46.5.

(*R*)-2-methyl-4-oxohex-5-en-1-yl 2,4,6-triisopropylbenzoate (4c)

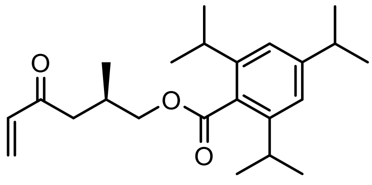

4c

Prepared following **General Procedure 1 and Oxidation Conditions 1a**, using methoxyallene (**1**) (32 μL, 0.38 mmol, 1.5 equiv.), *n*-BuLi (1.6 M in hexanes, 0.35 mmol, 1.4 equiv.), glacial acetic acid (36 μL, 0.63 mmol, 2.5 equiv.), (*R*)-2-methyl-3-(4,4,5,5-tetramethyl-1,3,2-dioxaborolan-2-yl)propyl 2,4,6-triisopropylbenzoate (108 mg, 0.25 mmol, 1.0 equiv.) and sodium perborate tetrahydrate (77 mg, 0.50 mmol, 2.0 equiv.). Purification by column chromatography (10% Et_2O in pentane) gave enone **4c** (62.2 mg, 72%) as a colourless oil.

R$_f$ = 0.27 (10% Et_2O/pentane)

$[\boldsymbol{\alpha}]_{\boldsymbol{D}}^{\boldsymbol{25}}$: −4 (c = 1)

^{1}H NMR (400 MHz, Chloroform-*d*) δ_H 7.01 (s, 2H), 6.34 (dd, *J* = 17.7, 10.5 Hz, 1H), 6.19 (dd, *J* = 17.7, 1.2 Hz, 1H), 5.81 (dd, *J* = 10.5, 1.2 Hz, 1H), 4.28 – 4.14 (m, 2H), 2.97 – 2.71 (m, 4H), 2.60 – 2.46 (m, 2H), 1.27 – 1.22 (m, 18H), 1.04 (d, *J* = 6.5 Hz, 3H).

^{13}C NMR (101 MHz, $CDCl_3$): δ_C 199.3, 171.1, 150.3 , 144.9 (2 × C), 136.9, 130.6, 128.3, 121.0 (2 × C), 69.2, 43.1, 34.5, 31.7, 28.8, 24.3 (4 × C), 24.1 (2 × C), 17.2.

HRMS (ESI$^+$): m/z calc'd for $C_{23}H_{34}NaO$ $[M + Na]^+$ requires 381.2400, found 381.2414.

IR (thin film) $\boldsymbol{\nu}_{\mathbf{max}}$: 2962, 1721, 1606, 1462,1251,1075 cm^{-1}

5-(1,3-dioxolan-2-yl)pent-1-en-3-one (4d)

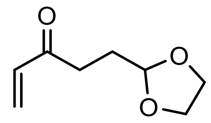

4d

Prepared following **General Procedure 1 and Oxidation Conditions 1a**, using methoxyallene (**1**) (32 µL, 0.38 mmol, 1.5 equiv.), *n*-BuLi (1.6 M in hexanes, 0.35 mmol, 1.4 equiv), glacial acetic acid (36 µL, 0.63 mmol, 2.5 equiv.), 2-(2-(1,3-dioxolan-2-yl)ethyl)-4,4,5,5-tetramethyl-1,3,2-dioxaborolane (59.0 mg, 0.25 mmol, 1.0 equiv.) and sodium perborate tetrahydrate (77 mg, 0.50 mmol, 2.0 equiv.). Purification by column chromatography (40% Et_2O/pentane) gave enone **4d** (19.7 mg, 50%) as a colourless oil.

^{1}H and ^{13}C NMR data in accordance with the literature.[24]

R*f* = 0.33 (40% Et_2O/pentane)

^{1}H NMR (400 MHz, Chloroform-*d*) δ_H 6.36 (dd, *J* = 17.7, 10.4 Hz, 1H), 6.23 (dd, *J* = 17.7, 1.2 Hz, 1H), 5.83 (dd, *J* = 10.4, 1.2 Hz, 1H), 4.93 (t, *J* = 4.3 Hz, 1H), 4.00 – 3.82 (m, 4H), 2.73 (t, *J* = 7.8, 7.0 Hz, 2H), 2.02 (dt, *J* = 7.8, 7.0, 4.3 Hz, 2H).

^{13}C NMR (101 MHz, $CDCl_3$): δ_C 200.0, 136.6, 128.3, 103.5, 65.1, 33.5, 27.7.

9-oxoundec-10-en-1-yl 2,4,6-triisopropylbenzoate (4e)

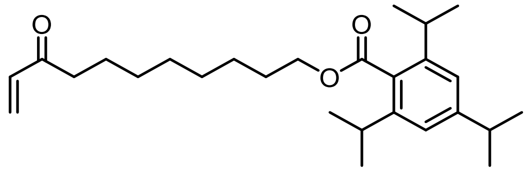

4e

Prepared following **General Procedure 1 and Oxidation Conditions 1a**, using methoxyallene (**1**) (32 µL, 0.38 mmol, 1.5 equiv.), *n*-BuLi (1.6 M in hexanes, 0.35 mmol, 1.4 equiv.), glacial acetic acid (36 µL, 0.63 mmol, 2.5 equiv.), 8-(4,4,5,5-tetramethyl-1,3,2-dioxaborolan-2-yl)octyl 2,4,6-triisopropylbenzoate (122 mg, 0.25 mmol, 1.0 equiv.) and sodium perborate tetrahydrate (77.0 mg, 0.50 mmol, 2.0 equiv.). Purification by column chromatography (20% Et_2O/pentane) gave enone **4e** (74.8 mg, 72%) as a colourless oil.

R*f* = 0.27 (10% Et_2O/pentane)

^{1}H NMR (400 MHz, Chloroform-*d*) δ_H 7.00 (s, 2H), 6.35 (dd, *J* = 17.7, 10.5 Hz, 1H), 6.20 (dd, *J* = 17.7, 1.3 Hz, 1H), 5.80 (dd, *J* = 10.5, 1.3 Hz, 1H), 4.29 (t, *J* = 6.6 Hz, 2H), 2.96 – 2.78 (m, 3H), 2.57 (t, *J* = 7.4 Hz, 2H), 1.71 (dt, *J* = 8.2, 6.6 Hz, 2H), 1.68 – 1.57 (m, 2H), 1.45 – 1.29 (m, 8H), 1.24 (d, *J* = 6.9, 18H).

^{13}C NMR (101 MHz, Chloroform-*d*) δ_C 201.1, 171.1, 150.1, 144.8 (2 × C), 136.7, 130.8, 128.0, 121.0 (2xC), 65.1, 39.7, 34.5, 31.6, 29.4, 29.3, 29.2, 28.7, 26.1, 24.3 (4 × C), 24.1 (2 × C), 24.1.

HRMS (ESI$^+$): m/z calc'd for $C_{27}H_{42}NaO_3$ [M + Na]$^+$ requires 437.3026, found 437.3026.

IR (thin film) ν_{max}: 2961, 2930, 2869, 1724, 1607, 1462, 1384,1251, 1138, 1104, 1076 cm^{-1}

5,5-di-*p*-tolylpent-1-en-3-one (4f)

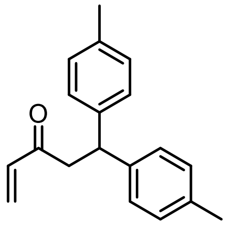

4f

Prepared following **General Procedure 1 and Oxidation Conditions 1a**, using methoxyallene (**1**) (32 μL, 0.38 mmol, 1.5 equiv.), *n*-BuLi (1.6 M in hexanes, 0.35 mmol, 1.4 equiv.), glacial acetic acid (36 μL, 0.63 mmol, 2.5 equiv.), 2-(2,2-di-p-tolylethyl)-4,4,5,5-tetramethyl-1,3,2-dioxaborolane (84.1 mg, 0.25 mmol, 1.0 equiv.) and sodium perborate tetrahydrate (77.0 mg, 0.50 mmol, 2.0 equiv.). Purification by flash column chromatography (BIOTAGE 10 g, 2-10% Et_2O/hexane) gave enone **4f** as an amorphous white solid (46.9 mg, 71%).

R_f = 0.24 (10% Et_2O/pentane)

^{1}H NMR (400 MHz, Chloroform-*d*) δ_H 7.14 – 7.04 (m, 8H), 6.32 (dd, *J* = 17.7, 10.4 Hz, 1H), 6.20 (dd, *J* = 17.7, 1.2 Hz, 1H), 5.79 (dd, *J* = 10.4, 1.2 Hz, 1H), 4.60 (t, *J* = 7.5 Hz, 1H), 3.31 (d, *J* = 7.5 Hz, 2H), 2.28 (s, 6H).

^{13}C NMR (101 MHz, Chloroform-*d*) δ_C 198.9, 141.3 (2 × C), 136.8, 136.0 (2 × C), 129.4 (4 × C), 128.4, 127.7 (4 × C), 46.0, 45.2, 21.1.

HRMS (ESI$^+$): m/z calc'd for $C_{19}H_{20}Na$ [M + Na]$^+$ requires 287.1406, found 287.1382.

IR (thin film) ν_{max}: 2961, 2930, 2869, 1724, 1683, 1607, 1462, 1401, 1384, 1363, 1189, 1104, 1076 cm^{-1}

5-([1,1'-biphenyl]-4-yl)pent-1-en-3-one (4g)

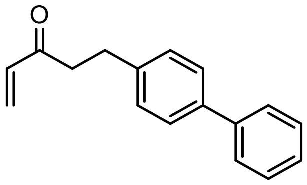

4g

Prepared following **General Procedure 1 and Oxidation Conditions 1a**, using methoxyallene (**1**) (32 μL, 0.38 mmol, 1.5 equiv.), *n*-BuLi (1.6 M in hexanes, 0.35 mmol, 1.4 equiv.), glacial acetic acid (36 μL, 0.63 mmol, 2.5 equiv.), 2-(2-([1,1'-biphenyl]-4-yl)ethyl)-4,4,5,5-tetramethyl-1,3,2-dioxaborolane (77.1 mg, 0.25 mmol, 1.0 equiv.), and sodium perborate tetrahydrate (77.0 mg, 0.50 mmol, 2.0 equiv.). Purification by column

chromatography (10% Et_2O/pentane) gave enone **4g** (37.2 mg, 63%) as a colourless oil.

R*f* = 0.30 (10% Et_2O/pentane)

^{1}H NMR (400 MHz, Chloroform-*d*) δ_H 7.61 – 7.57 (m, 2H), 7.55 – 7.51 (m, 2H), 7.46 – 7.42 (m, 2H,), 7.37 – 7.32 (m, 1H), 7.32 – 7.28 (m, 2H), 6.39 (dd, *J* = 17.7, 10.5 Hz, 1H), 6.25 (dd, *J* = 17.7, 1.2 Hz, 1H), 5.86 (dd, *J* = 10.5, 1.2 Hz, 1H), 3.06 – 2.93 (m, 4H).

^{13}C NMR (101 MHz, $CDCl_3$): δ_C 199.9, 141.1, 140.3, 139.2, 136.6, 128.9 (2 × C), 128.9 (2 × C), 128.5, 127.4 (2 × C), 127.2, 127.1 (2 × C), 41.3, 29.5.

IR (thin film) ν_{max}: 3027, 2927, 1678, 1614, 1487,1399, 1092, 1008 cm^{-1}

HRMS (EI): m/z calc'd for $C_{17}H_{16}O$ $[M]^+$ requires 236.1196, found 236.1194.

5-(2-bromophenyl)-4-methylpent-1-en-3-one (4h)

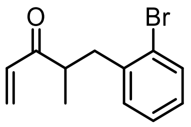

4h

Prepared following **General Procedure 1 and Oxidation Conditions 1b**, using methoxyallene (**1**) (32 μL, 0.38 mmol, 1.5 equiv.), *n*-BuLi (1.6 M in hexanes, 0.35 mmol, 1.4 equiv.), glacial acetic acid (36 μL, 0.63 mmol, 2.5 equiv.), 2-(1-(2-bromophenyl)propan-2-yl)-4,4,5,5-tetramethyl-1,3,2-dioxaborolane (78.7 mg, 0.25 mmol, 1.0 equiv.) and sodium perborate tetrahydrate (42.3 mg, 0.28 mmol, 1.1 equiv.). Purification by column chromatography on an automated system (BIOTAGE 10 g, 1-10% Et_2O/pentane) gave enone **4h** (30.8 mg, 52%) as a colourless oil.

R*f* = 0.37 (5% Et_2O/pentane)

^{1}H NMR (400 MHz, Chloroform-*d*) δ_H 7.53 (dd, *J* = 8.0, 1.3 Hz, 1H), 7.25 – 7.12 (m, 2H), 7.07 (ddd, *J* = 8.0, 6.9, 2.2 Hz, 1H), 6.39 (dd, *J* = 17.5, 10.5 Hz, 1H), 6.24 (dd, *J* = 17.5, 1.3 Hz, 1H), 5.76 (dd, *J* = 10.5, 1.3 Hz, 1H), 3.39 – 3.25 (m, 1H), 3.14 (dd, *J* = 13.5, 6.8 Hz, 1H), 2.71 (dd, *J* = 13.5, 7.7 Hz, 1H), 1.12 (d, *J* = 6.9 Hz, 3H).

^{13}C NMR (101 MHz, Chloroform-*d*) δ_C 203.5, 139.1, 135.6, 133.0, 132.0, 128.7, 128.2, 127.5, 124.7, 42.8, 39.3, 16.6.

HRMS (APCI): m/z calc'd for $C_{12}H_{13}BrO$ $[M + H]^+$ requires 253.0223, found 253.0215.

IR (thin film) ν_{max}: 2969, 1698, 1677, 1612, 1471, 1402, 1254, 1042 cm^{-1}

1-((1*S*,2*S*)-2-phenylcyclopropyl)prop-2-en-1-one (4i)

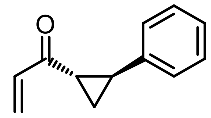

4i

Prepared following **General Procedure 1 and Oxidation Conditions 1b**, using methoxyallene (**1**) (32 µL, 0.38 mmol, 1.5 equiv.), *n*-BuLi (1.6 M in hexanes, 0.35 mmol, 1.4 equiv.), glacial acetic acid (36 µL, 0.63 mmol, 2.5 equiv.), 4,4,5,5-tetramethyl-2-((2S)-2-phenylcyclopropyl)-1,3,2-dioxaborolane (61.0 mg, 0.25 mmol, 1.0 equiv.) and sodium perborate tetrahydrate (42.3 mg, 0.28 mmol, 1.1 equiv.). Purification by column chromatography on an automated system (BIOTAGE 10 g, 5-20% Et_2O/pentane) gave enone **4i** (24.5 mg, 57%, dr >50:1) as a colourless oil.

R*f* = 0.27 (10% Et_2O/pentane)

$[\alpha]_D^{25}$: −8 (c = 0.25)

^{1}H NMR (400 MHz, Chloroform-*d*) δ_H 7.32 – 7.27 (m, 2H), 7.24 – 7.19 (m, 1H), 7.16 – 7.09 (m, 2H), 6.51 (dd, *J* = 17.6, 10.6 Hz, 1H), 6.29 (dd, *J* = 17.6, 1.1 Hz, 1H), 5.85 (dd, *J* = 10.6, 1.1 Hz, 1H), 2.59 (ddd, *J* = 9.0, 6.6, 4.0 Hz, 1H), 2.45 (ddd, *J* = 8.2, 5.3, 4.0 Hz, 1H), 1.77 (ddd, *J* = 9.0, 5.3, 4.2 Hz,1H), 1.46 (ddd, *J* = 8.2, 6.6, 4.2 Hz, 1H).

^{13}C NMR (101 MHz, Chloroform-*d*) δ_C 198.7, 140.5, 136.9, 128.7 (2 × C), 128.3, 126.7, 126.3 (2 × C), 30.4, 29.8, 19.4.

HRMS (EI): m/z calc'd for $C_{12}H_{12}O$ $[M-H]^+$ requires 171.0804, found 171.0803.

IR (thin film) ν_{max}: 2988, 1682, 1663, 1614, 1498, 1456, 1432, 1407, 1104, 1077 cm^{-1}

5-oxo-4-phenethylhept-6-enenitrile (4j)

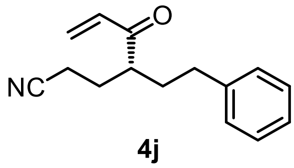

4j

Prepared following **General Procedure 1 and Oxidation Conditions 1b**, using methoxyallene (**1**) (32 µL, 0.38 mmol, 1.5 equiv.), *n*-BuLi (1.6 M in hexanes, 0.35 mmol, 1.4 equiv.), glacial acetic acid (36 µL, 0.63 mmol, 2.5 equiv.), 6-phenyl-4-(4,4,5,5-tetramethyl-1,3,2-dioxaborolan-2-yl)hexanenitrile (75.0 mg, 0.25 mmol, 1.0 equiv.), and sodium perborate tetrahydrate (42.3 mg, 0.28 mmol, 1.1 equiv.). Purification by column chromatography (30% Et_2O/pentane) gave enone **4j** (30.0 mg, 53%, 97:3 e.r., 100% e.s.) as a colourless oil.

R*f* = 0.33 (30% Et_2O/pentane)

$[\alpha]_D^{25}$: +12 (c = 1)

^{1}H NMR (400 MHz, Chloroform-d) δ_H 7.34 – 7.27 (m, 2H), 7.24 – 7.18 (m, 1H), 7.17 – 7.12 (m, 2H), 6.42 (dd, *J* = 17.5, 10.5 Hz, 1H), 6.25 (dd, *J* = 17.5, 1.0 Hz, 1H), 5.86 (dd, *J* = 10.5, 1.0 Hz, 1H), 3.04 (dtd, *J* = 9.2, 6.5, 4.4 Hz, 1H), 2.59 (t, *J* = 7.9 Hz, 2H), 2.38 (ddd, *J* = 17.0, 7.2, 5.8 Hz, 1H), 2.24 (ddd, *J* = 17.0, 8.4, 7.2 Hz, 1H), 2.16 – 2.06 (m, 1H), 2.06 – 1.96 (m, 1H), 1.87 – 1.70 (m, 2H).

^{13}C NMR (101 MHz, Chloroform-*d*) δ_C 202.1, 140.9, 135.8, 129.8, 128.7 (2 × C), 128.5 (2 × C), 126.4, 119.3, 46.3, 33.5, 33.2, 26.4, 15.3.

HRMS (EI): m/z calc'd for $C_{15}H_{17}NO$ $[M]^+$ requires 227.1305, found 227.1305.

IR (thin film) ν_{max}: 2931, 2245, 1694, 1673, 1611. 1497, 1404, 1207, 1041 cm^{-1}

e.r. determined by HPLC analysis of ent-**4j** and rac-**4j** against the oxidised parent boronic esters.

Chiral HPLC (Chiralpak IA column, 100% hexane, flow rate 1.0 mL min^{-1}, λ = 210 nm, rt): 4.1 min (major), 4.6 min (minor), 97:3

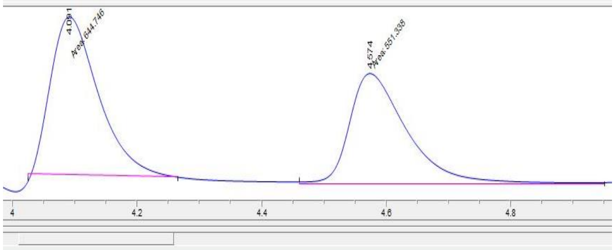

#	Time	Type	Area	Height	Width	Area%	Symmetry
1	4.091	MM	644.7	120.1	0.0895	53.905	0.624
2	4.574	MM	551.3	83.7	0.1098	46.095	0.52

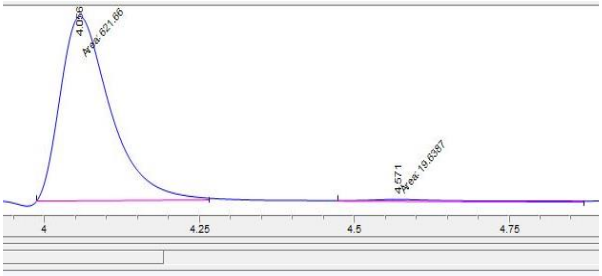

#	Time	Type	Area	Height	Width	Area%	Symmetry
1	4.056	MM	621.7	109.9	0.0943	96.938	0.611
2	4.571	MM	19.6	1.3	0.2529	3.062	0.297

1-((1*S*,2*S*,5*R*)-2-isopropyl-5-methylcyclohexyl)prop-2-en-1-one (4k)

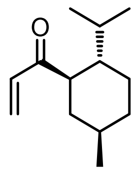

4k

Prepared following **General Procedure 1 and Oxidation Conditions 1c**, using methoxyallene (**1**) (32 μL, 0.38 mmol, 1.5 equiv.), *n*-BuLi (1.6 M in hexanes, 0.35 mmol, 1.4 equiv.), glacial acetic acid (36 μL, 0.63 mmol, 2.5 equiv.), 2-((2*R*,5*R*)-2-isopropyl-5-methylcyclohexyl)-4,4,5,5-tetramethyl-1,3,2-dioxaborolane (67.0 mg, 0.25 mmol, 1.0 equiv.), sodium hydroxide (0.75 mmol, 2 M, 3.0 equiv.), and hydrogen peroxide (1.5 mmol, 30%, 6.0 equiv.). Purification by column chromatography (50% toluene/pentane) gave enone **4k** (29.1 mg, 60%, dr >99:1) as a colourless oil.

$\mathbf{R_f}$ = 0.21 (50% toluene/pentane)

^{1}H and ^{13}C data in accordance with the literature.[25]

$[\alpha]_D^{25}$: −40 (c = 0.50)

^{1}H NMR (400 MHz, Chloroform-*d*) δ_H 6.44 (dd, *J* = 17.4, 10.4 Hz, 1H), 6.27 (dd, *J* = 17.4, 1.5 Hz, 1H), 5.76 (dd, *J* = 10.4, 1.5 Hz, 1H), 2.72 (ddd, *J* = 12.0, 10.8, 3.4 Hz, 1H), 1.78 – 1.55 (m, 5H), 1.48 – 1.31 (m, 1H), 1.09 – 0.93 (m, 4H), 0.89 (d, *J* = 6.6 Hz, 3H), 0.87 (d, *J* = 6.9 Hz, 3H), 0.75 (d, *J* = 6.9 Hz, 3H).

^{13}C NMR (101 MHz, Chloroform-*d*) δ_C 204.6, 135.8, 128.1, 51.5, 44.1, 38.9, 34.8, 32.6, 29.0, 24.2, 22.5, 21.5, 16.3.

HRMS (EI): m/z calc'd for $C_{13}H_{22}O$ $[M]^+$ requires 194.1665, found 194.1664.

IR (thin film) ν_{max}: 2955, 2922, 1693, 1673, 1609. 1455, 1432, 983 cm^{-1}

1-((3*S*,5*R*,6*S*,8*S*,9*S*,10*R*,13*R*,14*S*,17*R*)-3-chloro-10,13-dimethyl-17-((*R*)-6-methylheptan-2-yl)hexadecahydro-1H-cyclopenta[a]phenanthren-6-yl)prop-2-en-1-one (4l)

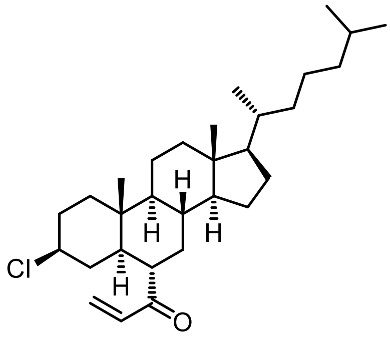

4l

Prepared following **General Procedure 1 and Oxidation Conditions 1c**, using methoxyallene (**1**) (32 μL, 0.38 mmol, 1.5 equiv.), *n*-BuLi (1.6 M in hexanes, 0.35 mmol, 1.4 equiv.), glacial acetic acid (36 μL, 0.63 mmol, 2.5 equiv.), 2-((3*S*,5*R*,6*S*,8*R*,9*S*,10*S*,13*R*,14*S*,17*R*)-3-chloro-10,13-dimethyl-17-((*R*)-6-methylheptan-2-yl)hexadecahydro-1H-cyclopenta[a]phenanthren-6-yl)-4,4,5,5-tetramethyl-1,3,2-dioxaborolane (133 mg, 0.25 mmol, 1.0 equiv.) sodium hydroxide (0.75 mmol, 2 M, 3.0 equiv.), and hydrogen peroxide (1.5 mmol, 30%, 6.0 equiv.). Purification by column chromatography (25% CH_2Cl_2/pentane) gave enone **4l** (75.4 mg, 66%, dr >99:1) as a waxy solid.

R_f = 0.19 (25% CH_2Cl_2/pentane)

$[\alpha]_D^{25}$: +48 (c = 0.25)

^{1}H NMR (400 MHz, Chloroform-*d*) δ_H 6.38 (dd, *J* = 17.5, 10.4 Hz, 1H), 6.24 (dd, *J* = 17.5, 1.4 Hz, 1H), 5.80 (dd, *J* = 10.4, 1.4 Hz, 1H), 3.81 (tt, *J* = 11.5, 5.0 Hz, 1H), 2.83 – 2.71 (m, 1H), 2.00 (ddt, *J* = 16.1, 12.6, 3.7 Hz, 2H), 1.88 – 1.64 (m, 5H), 1.59 – 1.38 (m, 5H), 1.39 – 0.93 (m, 16H), 0.93 – 0.88 (m, 6H), 0.86 (d, *J* = 1.9 Hz, 3H), 0.85 (d, *J* = 1.9 Hz, 3H), 0.81 – 0.68 (m, 1H), 0.65 (s, 3H).

^{13}C NMR (101 MHz, Chloroform-*d*) δ_C 204.0, 136.5, 128.7, 59.5, 56.3, 56.1, 53.5, 47.4, 47.3, 42.8, 39.9, 39.6, 38.6, 36.3 (x2), 35.9, 35.6, 35.2, 34.8, 33.0, 28.3, 28.1, 24.2, 24.0, 23.0, 22.7, 21.2, 18.8, 13.2, 12.2.

HRMS (MALDI): m/z calc'd for $C_{30}H_{49}ClNaO$ $[M+Na]^+$ requires 483.3364, found 483.3372

IR (thin film) ν_{max}: 2941, 2867, 1694, 1674, 1468. 1379, 756 cm^{-1}

1-((2*R*,3*R*)-2,6,6-trimethylbicyclo[3.1.1]heptan-3-yl)prop-2-en-1-one (4m)

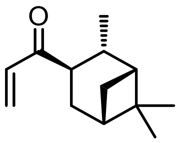

4m

Prepared following **General Procedure 1 and Oxidation Conditions 1c**, using methoxyallene (**1**) (32 μL, 0.38 mmol, 1.5 equiv.), *n*-BuLi (1.6 M in hexanes, 0.35 mmol, 1.4 equiv.), glacial acetic acid (36 μL, 0.63 mmol, 2.5 equiv.), 4,4,5,5-tetramethyl-2-((2*S*,3*R*)-2,6,6-trimethylbicyclo[3.1.1]heptan-3-yl)-1,3,2-dioxaborolane (66.1 mg, 0.25 mmol, 1.0 equiv.) sodium hydroxide (0.75 mmol, 2 M, 3.0 equiv.), and hydrogen peroxide (1.5 mmol, 30%, 6.0 equiv.). Purification by column chromatography (30% CH_2Cl_2/pentane) gave enone **4m** (33.3 mg, 69%, dr >99:1) as a colourless oil.

R_f = 0.28 (30% CH_2Cl_2/pentane)

$[\alpha]_D^{25}$: −24 (c = 0.25)

^{1}H NMR (400 MHz, Chloroform-*d*) δ_H 6.49 (dd, *J* = 17.4, 10.5 Hz, 1H), 6.28 (dd, *J* = 17.4, 1.2 Hz, 1H), 5.77 (dd, *J* = 10.5, 1.2 Hz, 1H), 2.98 (dt, *J* = 10.8, 6.7 Hz, 1H), 2.51 (pd, *J* = 7.0, 2.2 Hz, 1H), 2.34 – 2.16 (m, 2H), 1.96 (m, 1H), 1.91 – 1.80 (m, 2H), 1.22 (s, 3H), 1.07 – 1.02 (2 overlapping s, 6H), 0.90 (d, *J* = 9.8 Hz, 1H).

^{13}C NMR (101 MHz, Chloroform-*d*) δ_C 203.0, 136.0, 128.2, 47.4, 46.9, 41.0, 38.9, 36.6, 32.7, 30.3, 28.0, 23.1, 22.3.

HRMS (EI): m/z calc'd for $C_{13}H_{20}O$ $[M]^+$ requires 192.1509, found 192.1508.

IR (thin film) ν_{max}: 2911, 1677, 1670, 1613, 1454. 1400, 1122, 1082, 984 cm^{-1}

(4*R*,6*R*,8*S*)-9-((tert-butyldiphenylsilyl)oxy)-4,6,8-trimethylnon-1-en-3-one (4n)

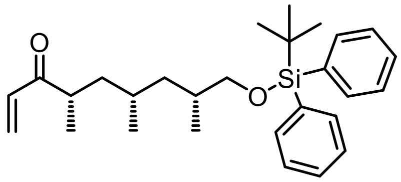

4n

Prepared following **General Procedure 1 and <u>modified</u> Oxidation Conditions 1b**, using methoxyallene (**1**) (32 μL, 0.38 mmol, 1.5 equiv.), *n*-BuLi (1.6 M in hexanes, 0.35 mmol, 1.4 equiv.), glacial acetic acid (36 μL, 0.63 mmol, 2.5 equiv.), tert-butyl(((2*S*,4*S*,6*R*)-2,4-dimethyl-6-(4,4,5,5-tetramethyl-1,3,2-dioxaborolan-2-yl)heptyl)oxy)diphenylsilane (132 mg, 0.25 mmol, 1.0 equiv.) and sodium perborate tetrahydrate (77.0 mg, 0.50 mmol, 2.0 equiv.). Purification by column chromatography (2% Et_2O/pentane) gave enone **4n** (63.1 mg, 56%, dr >99:1) as a colourless oil.

R*f* = 0.28 (2% Et_2O/pentane)

$[\alpha]_D^{25}$: +8 (c = 1)

^{1}H NMR (400 MHz, Chloroform-*d*) δ_H 7.72 – 7.63 (m, 4H), 7.46 – 7.35 (m, 6H), 6.43 (dd, *J* = 17.5, 10.4 Hz, 1H), 6.27 (dd, *J* = 17.5, 1.5 Hz, 1H), 5.75 (dd, *J* = 10.4, 1.5 Hz, 1H), 3.49 (dd, *J* = 9.8, 5.4 Hz, 1H), 3.43 (dd, *J* = 9.8, 6.3 Hz, 1H), 2.99 – 2.86 (m, 1H), 1.77 (m, 2H), 1.50 – 1.33 (m, 2H), 1.07 (m, 13H), 0.92 (d, *J* = 6.7 Hz, 3H), 0.88 (m, 1H), 0.84 (d, *J* = 6.4 Hz, 3H).

^{13}C NMR (101 MHz, Chloroform-*d*) δ_C 204.4, 135.8 (2 × C), 135.8 (2 × C), 135.2, 134.1 (2 × C), 129.6 (2 × C), 128.1, 127.7 (4 × C), 69.0, 41.3, 41.2, 40.5, 33.2, 28.3, 27.0 (3 × C), 20.8, 19.4, 17.8, 17.7.

HRMS (ESI): m/z calc'd for $C_{28}H_{40}KOSi$ $[M + K]^+$ requires 459.2490, found 459.2480.

IR (thin film) ν_{max}: 2958, 2929, 2857, 1698, 1679, 1612, 1590, 1472, 1460, 1428,1110, 1085 cm^{-1}

tert-butyl 4-acryloylpiperidine-1-carboxylate (4o)

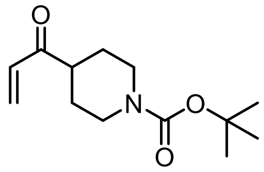

4o

Prepared following **General Procedure 1 and Oxidation Conditions 1b**, using methoxyallene (**1**) (32 µL, 0.38 mmol, 1.5 equiv.), *n*-BuLi (1.6 M in hexanes, 0.35 mmol, 1.4 equiv.), glacial acetic acid (36 µL, 0.63 mmol, 2.5 equiv.), tert-butyl 4-(4,4,5,5-tetramethyl-1,3,2-dioxaborolan-2-yl)piperidine-1-carboxylate (78.0 mg, 0.25 mmol, 1.0 equiv.) and sodium perborate tetrahydrate (42.3 mg, 0.28 mmol, 1.1 equiv.). Purification by column chromatography on an automated system (BIOTAGE 10 g, 10-80% Et_2O/pentane) gave enone **4o** (38.7 mg, 65%) as a colourless oil.

R*f* = 0.31 (40% Et_2O/pentane)

^{1}H NMR (400 MHz, Chloroform-*d*) δ_H 6.45 (dd, *J* = 17.4, 10.4 Hz, 1H), 6.29 (dd, *J* = 17.4, 1.4 Hz, 1H), 5.80 (dd, *J* = 10.4, 1.4 Hz, 1H), 4.11 (d, *J* = 13.3 Hz, 2H), 2.88 – 2.76 (t, *J* = 13.3, 2H), 2.74 (dt, *J* = 11.5, 3.8 Hz, 1H), 1.79 (d, *J* = 13.5 Hz, 2H), 1.57 (dtd, *J* = 13.5, 11.5, 4.3 Hz, 2H), 1.45 (s, 9H).

^{13}C NMR (101 MHz, Chloroform-*d*) δ_C 201.7, 154.8, 134.5, 128.7, 79.8, 46.2, 43.2 (2 × C), 28.6 (3 × C), 27.7 (2 × C).

HRMS (ESI$^+$): m/z calc'd for $C_{13}H_{21}NNaO_3$ $[M + Na]^+$ requires 262.1414, found 262.1420.

IR (thin film) ν_{max}: 2974, 1691, 1404, 1366, 1167, 1135, 1066 cm^{-1}

1-((3*r*,5*r*,7*r*)-adamantan-1-yl)prop-2-en-1-one (4p)

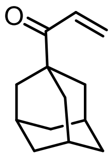

4p

Prepared following **General Procedure 1 and Oxidation Conditions 1c**, using methoxyallene (**1**) (32 μL, 0.38 mmol, 1.5 equiv.), *n*-BuLi (1.6 M in hexanes, 0.35 mmol, 1.4 equiv.), glacial acetic acid (36 μL, 0.63 mmol, 2.5 equiv.), 2-((3*r*,5*r*,7*r*)-adamantan-1-yl)-4,4,5,5-tetramethyl-1,3,2-dioxaborolane (65.6 mg, 0.25 mmol, 1.0 equiv.) sodium hydroxide (0.75 mmol, 2 M, 3.0 equiv.), and hydrogen peroxide (1.5 mmol, 30%, 6.0 equiv.). Purification by column chromatography (30% CH_2Cl_2/pentane) gave enone **4p** (28.4 mg, 60%) as a colourless oil.

R_f = 0.20 (30% CH_2Cl_2/pentane)

^{1}H and ^{13}C NMR data in accordance with the literature.[26]

^{1}H NMR (400 MHz, Chloroform-*d*) δ_H 6.84 (dd, *J* = 16.9, 10.4 Hz, 1H), 6.32 (dd, *J* = 16.9, 2.2 Hz, 1H), 5.63 (dd, *J* = 10.4, 2.2 Hz, 1H), 2.06 (m, 3H), 1.81 (d, *J* = 3.0 Hz, 6H), 1.78 – 1.66 (m, 6H).

^{13}C NMR (101 MHz, Chloroform-*d*) δ_C 204.1, 130.5, 128.4, 45.4, 37.8 (3 × C), 36.7 (3 × C), 28.0 (3 × C).

(*R*)-4-methyl-4-phenylhex-1-en-3-one (4q)

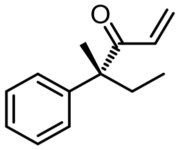

4q

Prepared following **General Procedure 1 and Oxidation Conditions 1c**, using methoxyallene (**1**) (32 μL, 0.38 mmol, 1.5 equiv.), *n*-BuLi (1.6 M in hexanes, 0.35 mmol, 1.4 equiv.), glacial acetic acid (36 μL, 0.63 mmol, 2.5 equiv.), (*S*)-4,4,5,5-tetramethyl-2-(2-phenylbutan-2-yl)-1,3,2-dioxaborolane (65.0 mg, 0.25 mmol, 1.0 equiv.) sodium hydroxide (0.75 mmol, 2 M, 3.0 equiv.), and hydrogen peroxide (1.5 mmol, 30%, 6.0 equiv.). Purification by column chromatography (50% toluene/pentane) gave enone **4q** (28.4 mg, 60%, 98:2 e.r., 100% e.s.) as a colourless oil.

R_f = 0.20 (50% toluene/pentane)

$[\alpha]_D^{25}$: −56 (c = 0.25)

^{1}H NMR (400 MHz, Chloroform-*d*) δ_H 7.34 (m, 2H), 7.28 – 7.23 (m, 1H), 7.23 – 7.18 (m, 2H), 6.35 – 6.28 (dd, 16.9, 2.4 Hz, 1H), 6.27 – 6.19 (dd, *J* = 16.9, 9.7 Hz, 1H), 5.48 (dd, *J* = 9.7, 2.4 Hz, 1H), 2.01 (qd, *J* = 7.4, 4.5 Hz, 2H), 1.45 (s, 3H), 0.76 (t, *J* = 7.4 Hz, 3H).

^{13}C NMR (101 MHz, Chloroform-*d*) δ_C 201.5, 142.8, 132.7, 128.9 (2 × C), 127.9, 127.0 (3 × C), 54.9, 29.8, 21.1, 8.7.

HRMS (APCI): m/z calc'd for $C_{13}H_{16}O$ $[M+H]^+$ requires 189.1274, found 189.1270

IR (thin film): ν_{max} 2970, 2937, 1696, 1611, 1495, 1446, 1398, 755, 700 cm^{-1}

e.r. determined by HPLC analysis of ent-**4q** and rac-**4q** against the oxidised parent boronic esters.

Chiral HPLC (Chiralpak IA column, 100% hexane, flow rate 0.5 mL min^{-1}, λ = 210 nm, rt): 24.8 min (major), 27.5 min (minor), 98:2

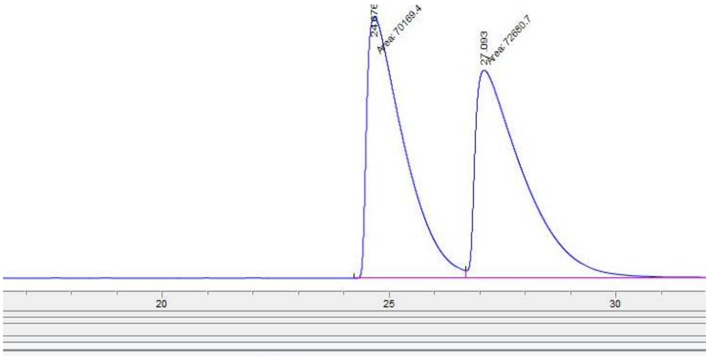

#	Time	Type	Area	Height	Width	Area%	Symmetry
1	24.676	MF	70169.4	1234.3	0.9475	49.121	0.246
2	27.093	FM	72680.7	981.3	1.2344	50.879	0.218

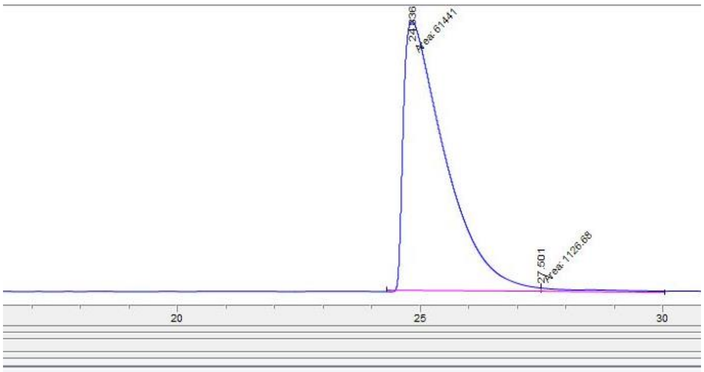

#	Time	Type	Area	Height	Width	Area%	Symmetry
1	24.836	MF	61441	1026.1	0.998	98.199	0.254
2	27.501	FM	1126.7	14.3	1.3087	1.801	0

(*R*)-4-cyclopropyl-4-phenylpent-1-en-3-one (4r)

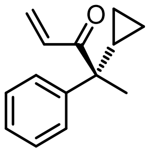

4r

Prepared following **General Procedure 1 and Oxidation Conditions 1c**, using methoxyallene (**1**) (32 μL, 0.38 mmol, 1.5 equiv.), *n*-BuLi (1.6 M in hexanes, 0.35 mmol, 1.4 equiv.), glacial acetic acid (36 μL, 0.63 mmol, 2.5 equiv.), (*S*)-2-(1-cyclopropyl-1-phenylethyl)-4,4,5,5-tetramethyl-1,3,2-dioxaborolane (68.1 mg, 0.25 mmol, 1.0

equiv.) sodium hydroxide (0.75 mmol, 2 M, 3.0 equiv.), and hydrogen peroxide (1.5 mmol, 30%, 6.0 equiv.). Purification by column chromatography (50% toluene/pentane) gave enone **4r** (27.8 mg, 56%, 96:4 e.r., 100% e.s.) as a colourless oil.

R*f* = 0.24 (50% toluene/pentane)

$[\alpha]_D^{25}$: +72 (c = 0.25)

^{1}H NMR (400 MHz, Chloroform-*d*) δ_H 7.38 – 7.23 (m, 5H), 6.41 – 6.26 (m, 2H), 5.53 (dd, *J* = 8.8, 3.5 Hz, 1H), 1.40 (m, 1H), 1.10 (s, 3H), 0.68 (m, 1H), 0.45 (m, 2H), 0.20 (m, 1H).

^{13}C NMR (101 MHz, Chloroform-*d*) δ_C 200.5, 143.9, 133.1, 128.8 (2 × C), 127.8, 127.4 (2 × C), 127.0, 54.2, 19.8, 16.2, 2.3, 0.5.

HRMS (APCI): m/z calc'd for $C_{14}H_{16}O$ $[M+H]^+$ requires 201.1274, found 201.1270

IR (thin film): ν_{max} 3084, 2983, 1696, 1610, 1492, 1445, 1398, 701 cm^{-1}

e.r. determined by HPLC analysis of ent-**4r** and rac-**4r** against the oxidised parent boronic esters.

Chiral HPLC (Chiralpak IA column, 100% hexane, flow rate 0.5 mL min^{-1}, λ = 210 nm, rt): 21.0 min (major), 24.5 min (minor), 96:4

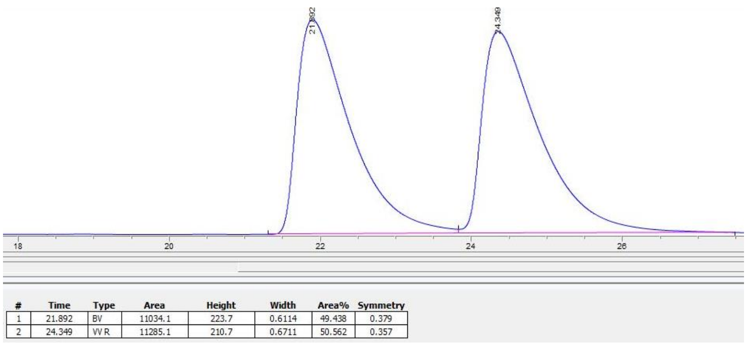

#	Time	Type	Area	Height	Width	Area%	Symmetry
1	21.892	BV	11034.1	223.7	0.6114	49.438	0.379
2	24.349	VV R	11285.1	210.7	0.6711	50.562	0.357

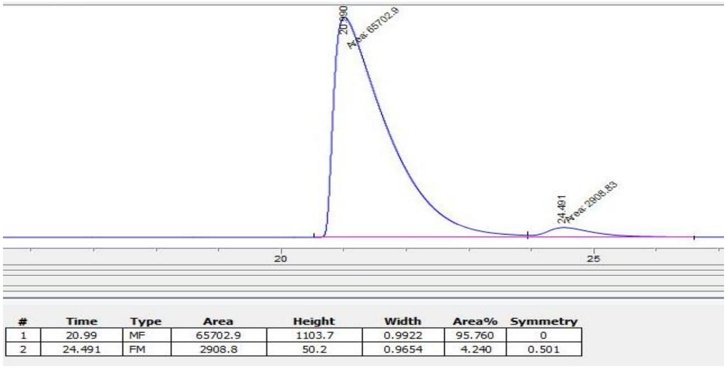

#	Time	Type	Area	Height	Width	Area%	Symmetry
1	20.99	MF	65702.9	1103.7	0.9922	95.760	0
2	24.491	FM	2908.8	50.2	0.9654	4.240	0.501

tert-butyl 3-acryloyl-3-cyclohexylazetidine-1-carboxylate (4s)

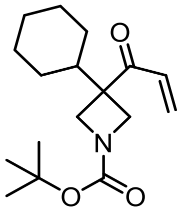

4s

Prepared following **General Procedure 1 and Oxidation Conditions 1c**, using methoxyallene (**1**) (32 μL, 0.38 mmol, 1.5 equiv.), *n*-BuLi (1.6 M in hexanes, 0.35 mmol, 1.4 equiv.), glacial acetic acid (36 μL, 0.63 mmol, 2.5 equiv.), tert-butyl 3-cyclohexyl-3-(4,4,5,5-tetramethyl-1,3,2-dioxaborolan-2-yl)azetidine-1-carboxylate (91.3 mg, 0.25 mmol, 1.0 equiv.), sodium hydroxide (0.75 mmol, 2 M, 3.0 equiv.), and hydrogen peroxide (1.5 mmol, 30%, 6.0 equiv.). Purification by column chromatography (25% Et_2O/pentane) gave enone **4s** (39.3 mg, 54%) as a colourless oil.

R_f = 0.22 (25% Et_2O/pentane)

^{1}H NMR (400 MHz, Chloroform-*d*) δ_H 6.45 (dd, *J* = 17.1, 9.7 Hz, 1H), 6.37 (dd, *J* = 17.1, 2.4 Hz, 1H), 5.76 (dd, *J* = 9.7, 2.4 Hz, 1H), 4.08 (d, *J* = 8.9 Hz, 2H), 3.84 (d, *J* = 8.9 Hz, 2H), 1.84 – 1.65 (m, 6H), 1.42 (s, 9H), 1.30 – 0.92 (m, 5H).

^{13}C NMR (101 MHz, Chloroform-*d*) δ_C 199.7, 156.4, 131.7, 129.8, 79.9, 52.0 (2 × C), 42.2, 28.5 (3 × C), 27.4 (2 × C), 26.5 (3 × C), 26.2.

HRMS (**Nanospray):** m/z calc'd for $C_{17}H_{27}NO_3$ $[M+H]^+$ requires 294.2069, found 294.2062

IR (thin film): ν_{max} 2974, 2928, 2855, 1698, 1609, 1479, 1392, 1366, 1164, 1130 cm^{-1}

1-(1-methyl-3-phenylcyclobutyl)prop-2-en-1-one (4t)

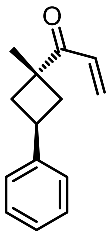

4t

Prepared following **General Procedure 1 and Oxidation Conditions 1c**, using methoxyallene (**1**) (32 μL, 0.38 mmol, 1.5 equiv.), *n*-BuLi (1.6 M in hexanes, 0.35 mmol, 1.4 equiv.), glacial acetic acid (36 μL, 0.63 mmol, 2.5 equiv.), 4,4,5,5-tetramethyl-2-(1-methyl-3-phenylcyclobutyl)-1,3,2-dioxaborolane (68.0 mg, 0.25 mmol, 1.0 equiv.), sodium hydroxide (0.75 mmol, 2 M, 3.0 equiv.), and hydrogen peroxide (1.5 mmol, 30%, 6.0 equiv.). Purification by column chromatography (70% toluene/pentane) gave enone **4t** (36.2 mg, 72%, dr >50:1) as a colourless oil.

R_f = 0.23 (70% toluene/pentane)

$[\alpha]_D^{25}$: −8 (c = 0.25)

^{1}H NMR (400 MHz, Chloroform-*d*) δ_H 7.35 – 7.28 (m, 2H), 7.23 – 7.17 (m, 3H), 6.73 (dd, *J* = 17.2, 10.3, 1H), 6.46 (dd, *J* = 17.2, 1.7 Hz, 1H), 5.78 (dd, *J* = 10.3, 1.7 Hz, 1H), 3.32 (p, *J* = 9.2 Hz, 1H), 2.96 (td, *J* = 8.9, 1.5 Hz, 2H), 2.08 (td, *J* = 9.6, 2.7 Hz, 2H), 1.39 (s, 3H).

^{13}C NMR (101 MHz, Chloroform-*d*) δ_C 202.6, 145.4, 131.4, 129.1, 128.5, 126.5 (2 × C), 126.1 (2 × C), 46.1, 38.4 (2 × C), 33.6, 25.3.

HRMS (APCI): m/z calc'd for $C_{14}H_{16}O$ $[M+H]^+$ requires 201.1274, found 201.1270

IR (film): ν_{max} 2960, 2930, 1693, 1612, 1495, 1456, 1401, 1039, 750, 698 cm^{-1}

Synthesis of (Z)-1,5-diphenylpent-1-en-3-one (7)

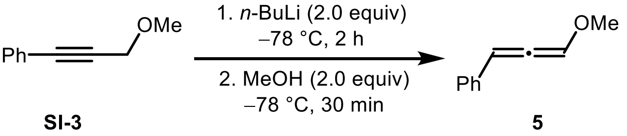

Compound **5** was prepared according to a modified literature procedure.[27]

(3-Methoxyprop-1-yn-1-yl)benzene (**SI-3**) (150 mg, 1.02 mmol, 1.0 equiv) was cooled (−78 °C) in anhydrous Et_2O (4.0 mL). *n*-Butyllithium (2.04 mmol, 2.0 equiv, 1.6 M in hexanes) was added dropwise and the reaction was left to stir (−78 °C, 2 h). Anhydrous MeOH (0.09 mL, 2.04 mmol, 2.0 equiv) was added dropwise and the reaction stirred (−78 °C, 30 min). The reaction was allowed to warm (rt) and quenched with $NaHCO_3$ (10 mL). The organic layer was separated, and the aqueous layer extracted with Et_2O (3 × 10 mL). The combined organic layers were dried ($MgSO_4$) and concentrated under reduced pressure to afford crude allene **5** which was checked by ^{1}H NMR and used in the next step without further purification.

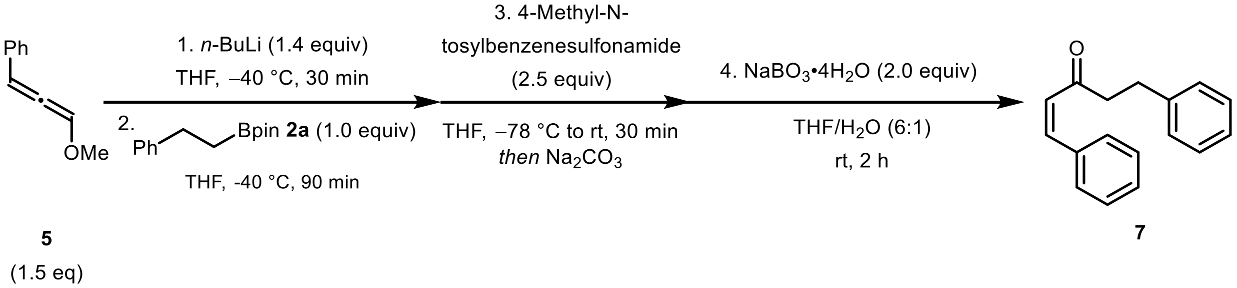

To a flame dried Schlenk flask with a magnetic follower under a nitrogen atmosphere, dry THF (2.0 mL) was cooled (−40 °C) using a cryostat. Crude (3-methoxypropa-1,2-dien-1-yl)benzene (**5**) (55.6 mg, 0.38 mmol, 1.5 equiv.) was added and the flask walls washed with dry THF (1.0 mL, to give 3 mL total). *n*-Butyllithium (0.35 mmol, 1.4 equiv., 1.6 M in hexanes) was added dropwise, and the solution stirred (−40 °C, 30 min). 4,4,5,5-tetramethyl-2-phenethyl-1,3,2-dioxaborolane (**2a**) (58 mg, 0.25 mmol, 1.0 equiv.) was added dropwise to the reaction as a solution in dry THF (0.30 mL) and the flask walls washed with dry THF (0.30 mL, 3.6 mL total). The reaction was stirred (−40 °C, 90 min) and then cooled (−78 °C) followed by the addition of 4-Methyl-*N*-tosylbenzenesulfonamide (proton source **B**) (203 mg, 0.625 mmol, 2.5 equiv.). The reaction was permitted to warm to room temperature and allowed to stir (30 min). The reaction was then quenched with Na_2CO_3 (aq. sat., 0.6 mL) followed by the addition of sodium perborate tetrahydrate (77 mg, 0.5 mmol, 2.0 equiv.). The reaction was allowed to stir (rt, 2 h), and the oxidation monitored by TLC. The reaction was diluted with water (10 mL) and the aqueous layer extracted with diethyl ether (3 × 10 mL). The combined organic layers were dried over $MgSO_4$, filtered, and concentrated under reduced pressure. The crude material was purified by column chromatography (BIOTAGE 10 g HC silica, 2-10% EtOAc/pentane) to afford (Z)-enone **7** (38.8 mg, 66%) as a yellow oil.

R_f = 0.23 (70% toluene/pentane)

^{1}H NMR (400 MHz, Chloroform-*d*) δ_H 7.48 (m, 2H), 7.37 – 7.31 (m, 3H), 7.28 – 7.23 (m, 2H), 7.21 – 7.10 (m, 3H), 6.85 (d, *J* = 12.7 Hz, 1H), 6.18 (d, *J* = 12.7 Hz, 1H), 2.92 (m, 2H), 2.82 – 2.74 (m, 2H).

^{13}C NMR (101 MHz, Chloroform-*d*) δ_C 202.4, 141.1, 140.1, 135.4, 129.6, 129.3, 128.6, 128.5, 128.4, 126.2, 45.2, 30.2.

HRMS (ESI): m/z calc'd $C_{17}H_{16}O$ $[M+H]^+$ requires 237.1274, found 237.1270

IR (film): ν_{max} 3026, 2926, 1689, 1602, 1494, 1453, 1090, 751, 695 cm^{-1}

2.4. Synthesis of α-Substituted Enones from Boronic Esters

2.4.1. General Procedure 2: Synthesis of α-Substituted Enones from Boronic Esters

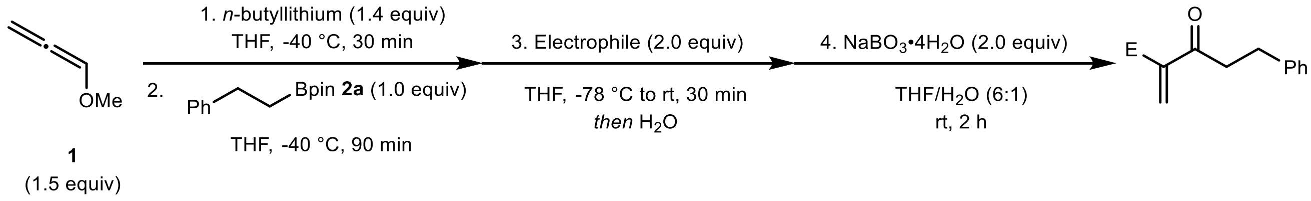

To a flame dried Schlenk flask with a magnetic follower under a nitrogen atmosphere, dry THF (2.0 mL) was cooled (−40 °C) using a cryostat. Methoxyallene (**1**) (32 µL, 0.38 mmol, 1.5 equiv.) was added and the flask walls washed with dry THF (1.0 mL, to give 3 mL total). *n*-Butyllithium (0.35 mmol, 1.4 equiv., 1.6 M in hexanes) was added dropwise, and the solution stirred (−40 °C, 30 min). 4,4,5,5-tetramethyl-2-phenethyl-1,3,2-dioxaborolane (**2a**) (58 mg, 0.25 mmol, 1.0 equiv.) was added dropwise to the reaction as a solution in dry THF (0.30 mL) and the flask walls washed with dry THF (0.30 mL, 3.6 mL total). The reaction was stirred (−40 °C, 90 min) and then cooled (−78 °C) followed by addition of the electrophile (0.5 mmol, 2.0 equiv.). The reaction was permitted to warm to room temperature and allowed to stir (30 min). The reaction was then quenched with either water (0.6 mL) or Na_2CO_3 (aq. sat., 0.6 mL) followed by the addition of sodium perborate tetrahydrate (77 mg, 0.5 mmol, 2.0 equiv.). The reaction was allowed to stir, and the oxidation monitored by TLC (see specific substrates for reaction time). The reaction was diluted with water (10 mL) and the aqueous layer extracted with diethyl ether (3 × 10 mL). The combined organic layers were dried over $MgSO_4$, filtered, and concentrated under reduced pressure. The crude material was purified by column chromatography.

2-((dimethylamino)methyl)-5-phenylpent-1-en-3-one (8a)

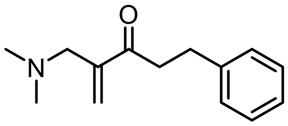

8a

Prepared following **General Procedure 2**, using methoxyallene (**1**) (32 μL, 0.38 mmol, 1.5 equiv.), *n*-BuLi (1.6 M in hexanes, 0.35 mmol, 1.4 equiv.), Eschenmoser's salt (93 mg, 0.50 mmol, 2.0 equiv.), 4,4,5,5-tetramethyl-2-phenethyl-1,3,2-dioxaborolane (**2a**) (58.0 mg, 0.25 mmol, 1.0 equiv.), sodium perborate tetrahydrate (77.0 mg, 0.5 mmol, 2.0 equiv., 2 h oxidation time at rt). Purification by column chromatography (1% NEt_3/CH_2Cl_2) gave enone **8a** (36.8 mg, 68%) as a yellow oil.

N.B. Due to the amine moiety, a modified work up was required for this substrate. Following oxidation with sodium perborate tetrahydrate, the reaction was diluted with HCl (2 M, 10 mL) and the aqueous layer washed with diethyl ether (5 × 10 mL). The aqueous layer was then basified with sodium carbonate (sat. aq., 10 mL) and extracted again with diethyl ether (3 × 10 mL). The combined organic layers were dried over $MgSO_4$, filtered, and concentrated under reduced pressure.

R_f = 0.23 (1% triethylamine in CH_2Cl_2)

^{1}H NMR (400 MHz, Chloroform-*d*) δ_H 7.32 – 7.26 (m, 2H), 7.23 – 7.16 (m, 3H), 6.09 (d, *J* = 1.0 Hz, 1H), 5.82 (d, *J* = 1.0 Hz, 1H), 3.12 (s, 2H), 3.03 (ddd, *J* = 8.6, 6.8, 1.8 Hz, 2H), 2.95 (ddd, *J* = 8.6, 6.8, 1.8 Hz, 2H), 2.21 (s, 6H).

^{13}C NMR (101 MHz, Chloroform-*d*) δ_C 200.9, 145.6, 141.5, 128.6 (2 × C), 128.5 (2 × C), 126.2, 125.7, 59.6, 45.6 (2 × C), 40.2, 30.3.

HRMS (ESI): m/z calc'd for $C_{14}H_{19}NO$ $[M+H]^+$ requires 218.1539, found 218.1532.

IR (film): ν_{max} 2942, 2818, 2769,1678, 1627, 1496, 1454, 1030, 749 cm^{-1}

2-(cyclohepta-2,4,6-trien-1-yl)-5-phenylpent-1-en-3-one (8b)

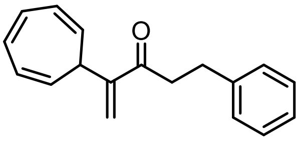

8b

Prepared following **General Procedure 2**, using methoxyallene (**1**) (32 μL, 0.38 mmol, 1.5 equiv.), *n*-BuLi (1.6 M in hexanes, 0.35 mmol, 1.4 equiv.), tropylium tetrafluoroborate (89.0 mg, 0.50 mmol, 2.0 equiv.), 4,4,5,5-tetramethyl-2-phenethyl-1,3,2-dioxaborolane (**2a**) (58.0 mg, 0.25 mmol, 1.0 equiv.), sodium perborate tetrahydrate (77.0 mg, 0.5 mmol, 2.0 equiv., 20 h oxidation time at rt). Purification by column chromatography (5% Et_2O/pentane) gave enone **8b** (40.1 mg, 64%) as a colourless oil.

R_f = 0.43 (10% Et_2O/pentane)

^{1}H NMR (400 MHz, Chloroform-*d*) δ_H 7.33 – 7.27 (m, 2H), 7.22 (m, 3H), 6.63 (*apparent* t, *J* = 3.4 Hz, 2H), 6.22 (dt, *J* = 9.5, 3.4 Hz, 2H), 6.16 (s, 1H), 5.93 (s, 1H), 5.26 (dd, *J* = 9.5, 6.2 Hz, 2H), 3.05 (m, 3H), 3.00 – 2.93 (m, 2H).

^{13}C NMR (101 MHz, Chloroform-*d*) δ_C 200.5, 148.6, 141.4, 130.8 (2 × C), 128.6 (2 × C), 128.5 (2 × C), 126.2, 125.2 (2 × C), 124.9 (2 × C), 124.1, 40.2, 40.1, 30.4.

HRMS (EI): m/z calc'd for $C_{18}H_{18}O$ $[M]^+$ requires 250.1352, found 250.1351.

IR (film): ν_{max} 3023, 2924, 1676, 1627, 1496, 1453, 939, 742 cm^{-1}

2-(benzo[d][1,3]dithiol-2-yl)-5-phenylpent-1-en-3-one (8c)

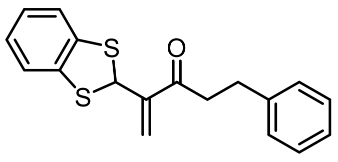

8c

Prepared following **General Procedure 2**, using methoxyallene (**1**) (32 µL, 0.38 mmol, 1.5 equiv.), *n*-BuLi (1.6 M in hexanes, 0.35 mmol, 1.4 equiv.), 1,3-dibenzathiolylium tetrafluoroborate (120 mg, 0.50 mmol, 2.0 equiv.), 4,4,5,5-tetramethyl-2-phenethyl-1,3,2-dioxaborolane (**2a**) (58.0 mg, 0.25 mmol, 1.0 equiv.), sodium perborate tetrahydrate (77.0 mg, 0.5 mmol, 2.0 equiv., 24 h oxidation time at rt). Purification by column chromatography (20% CH_2Cl_2/pentane) gave enone **8c** (40.1 mg, 64%) as a waxy white solid.

N.B. For substrate **8c** the authors found it necessary to extend the stirring time (rt, 120 min) for the 1,2-migration to ensure completion.

R_f = 0.50 (50% CH_2Cl_2/pentane)

^{1}H NMR (400 MHz, Chloroform-*d*) δ_H 7.31 – 7.26 (m, 2H), 7.25 – 7.17 (m, 5H), 7.07 – 7.01 (m, 2H), 6.32 (s, 1H), 6.20 (s, 1H), 5.77 (s, 1H), 3.04 (ddd, *J* = 8.6, 6.6, 1.8 Hz, 2H), 2.96 (ddd, *J* = 8.6, 6.6, 1.8 Hz, 2H).

^{13}C NMR (101 MHz, Chloroform-*d*) δ_C 199.4, 146.1, 141.0, 137.1 (2 × C), 128.7 (2 × C), 128.5 (2 × C), 126.4, 126.1, 125.9 (2 × C), 122.7 (2 × C), 49.9, 40.2, 30.2.

HRMS (EI): m/z calc'd for $C_{18}H_{16}OS_2$ $[M]^+$ requires 312.0637, found 312.0634.

IR (film): ν_{max} 3024, 2925, 1675, 1626, 1444, 743 cm^{-1}

5-phenyl-2-(phenylthio)pent-1-en-3-one (8d)

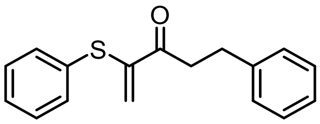

8d

Prepared following **General Procedure 2**, using methoxyallene (**1**) (32 μL, 0.38 mmol, 1.5 equiv.), *n*-BuLi (1.6 M in hexanes, 0.35 mmol, 1.4 equiv.), phenyl hypochlorothioite (72.3 mg, 0.50 mmol, 2.0 equiv.), 4,4,5,5-tetramethyl-2-phenethyl-1,3,2-dioxaborolane (**2a**) (58.0 mg, 0.25 mmol, 1.0 equiv.), sodium perborate tetrahydrate (77.0 mg, 0.5 mmol, 2.0 equiv., 2 h oxidation time at 50 °C). Enone **8d** was found to be highly unstable to silica gel, a quantitative ^{1}H NMR spectrum in the presence of CH_2Br_2 (1.0 equiv.) as an internal standard showed enone **8d** had formed in 43% NMR yield.

Crude ^{1}H NMR (400 MHz, Chloroform-*d*) δ_H 6.10 (d, *J* = 1.3 Hz, 1H), 5.28 (d, *J* = 1.3 Hz, 1H), 3.05 – 3.0 (m, 2H), 2.93 – 2.89 (m, 2H). Protons in the aromatic region could not be assigned.

HRMS (EI): m/z calc'd for $C_{17}H_{16}OS$ $[M]^+$ requires 268.0916, found 268.0915.

5-phenyl-2-(phenylselanyl)pent-1-en-3-one (8e)

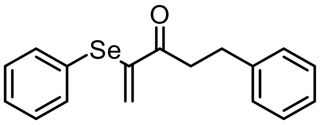

8e

Prepared following **General Procedure 2**, using methoxyallene (**1**) (32 μL, 0.38 mmol, 1.5 equiv.), *n*-BuLi (1.6 M in hexanes, 0.35 mmol, 1.4 equiv.), phenyl hypochloroselenoite (95.8 mg, 0.50 mmol, 2.0 equiv.), 4,4,5,5-tetramethyl-2-phenethyl-1,3,2-dioxaborolane (**2a**) (58.0 mg, 0.25 mmol, 1.0 equiv.), sodium perborate tetrahydrate (77.0 mg, 0.5 mmol, 2.0 equiv., 2 h oxidation time at rt). Enone **8e** was found to be highly unstable to silica gel, a quantitative ^{1}H NMR spectrum in the presence of CH_2Br_2 (1.0 equiv.) as an internal standard showed enone **8e** had formed in 45% NMR yield.

Crude ^{1}H NMR (400 MHz, Chloroform-*d*) δ_H 6.44 (d, *J* = 2.0 Hz, 1H), 5.46 (d, *J* = 2.0 Hz, 1H), 3.11 – 3.04 (m, 2H), 2.95 (m, 2H). Protons in the aromatic region could not be assigned.

HRMS (EI): m/z calc'd for $C_{17}H_{16}OSe$ $[M]^+$ requires 316.0361, found 316.0356.

2-fluoro-5-phenylpent-1-en-3-one (8f)

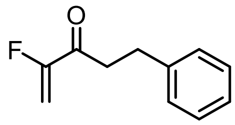

8f

Prepared following **General Procedure 2**, using methoxyallene (**1**) (32 μL, 0.38 mmol, 1.5 equiv.), *n*-BuLi (1.6 M in hexanes, 0.35 mmol, 1.4 equiv.), Selectfluor (177 mg, 0.50 mmol, 2.0 equiv.), 4,4,5,5-tetramethyl-2-phenethyl-1,3,2-dioxaborolane (**2a**) (58.0 mg, 0.25 mmol, 1.0 equiv.), sodium perborate tetrahydrate (77.0 mg, 0.5 mmol, 2.0 equiv., 2 h oxidation time at rt). Purification by column chromatography on an automated system (BIOTAGE 10 g, 2-10% Et_2O/pentane) gave enone **8f** (23.1 mg, 52%) as a colourless oil.

N.B. For substrate **8f**, the authors found it necessary to include an aqueous work up before oxidation to achieve best results. After stirring (30 min, rt) with Selectfluor, the reaction was diluted with H_2O (10 mL) and extracted with Et_2O (3 × 10 mL). The combined organic layers were dried ($MgSO_4$) and concentrated under reduced pressure. The crude material was redissolved in THF/H_2O (3.6:0.6 mL, 6:1) and $NaBO_4$•$4H_2O$ (77.0 mg, 0.5 mmol, 2.0 equiv.) added as a single portion. The reaction was left to stir (rt, 2 h) before work up according to **General Procedure 2** and purification.

$\mathbf{R_f}$ = 0.60 (10% Et_2O/pentane)

^{1}H NMR (400 MHz, Chloroform-*d*) δ_H 7.33 – 7.28 (m, 2H), 7.22 (m, 3H), 5.57 (dd, *J* = 45.2, 3.4 Hz, 1H), 5.21 (dd, *J* = 14.3, 3.4 Hz, 1H), 3.02 – 2.94 (m, 4H).

^{13}C NMR (101 MHz, Chloroform-*d*) δ_C 193.5 (d, $J_{C\text{-}F}$ = 32.3 Hz), 159.8 (d, $J_{C\text{-}F}$ = 268.2 Hz), 140.6, 128.7 (2 × C), 128.5 (2 × C), 126.4, 100.8, 100.6, 39.9, 29.3.

^{19}F NMR (376 MHz, Chloroform-*d*) δ_F −117.12 (dd, *J* = 45.2, 14.3 Hz, 1F).

HRMS (EI): m/z calc'd for $C_{11}H_{11}FO$ $[M]^+$ requires 178.0786, found 178.0788.

IR (film): ν_{max} 3029, 1670, 1641, 1496, 1454, 1373, 898, 750, 699

2-iodo-5-phenylpent-1-en-3-one (8g)

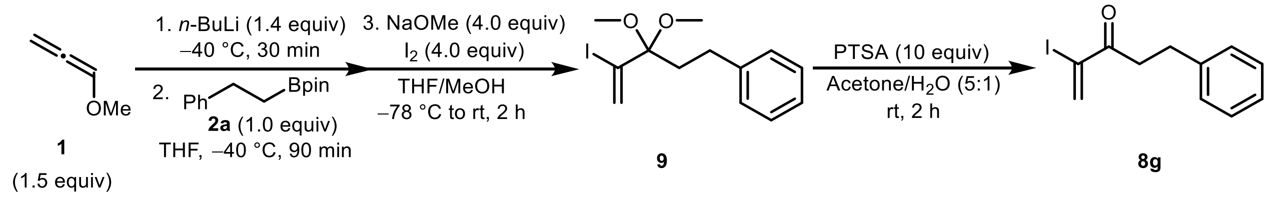

Methoxyallene (**1**) (32 μL, 0.38 mmol, 1.5 equiv.) was cooled (−40 °C) in anhydrous THF (3.0 mL). n-Butyllithium (1.6 M, 1.4 mmol, 1.4 equiv.) was added dropwise and the reaction stirred (30 min, −40 °C). 4,4,5,5-tetramethyl-

2-phenethyl-1,3,2-dioxaborolane (**2a**) (58 mg, 0.25 mmol, 1.0 equiv.) was added dropwise as a solution in anhydrous THF (0.3 mL) and the flask walls washed with anhydrous THF (0.3 mL, 3.6 mL total). The reaction was left to stir (−40 °C, 90 min) and then cooled (−78 °C) followed by the addition of NaOMe as a 0.5 M solution in MeOH (2.0 mL, 1.0 mmol, 4.0 equiv.) and I_2 (284 mg, 1.0 mmol, 4.0 equiv.) as a 0.5 M solution in MeOH. The reaction was left to stir (−78 °C, 30 min) and then allowed to warm and stir (rt, 2 h). The reaction was quenched with sodium thiosulfate (aq. sat. 10 mL) and the aqueous layer extracted (Et_2O 3 × 10 mL). The combined organic layers were dried ($MgSO_4$) and concentrated under reduced pressure. Crude iodoacetal **9** was dissolved in acetone/H_2O (5:1 mixture, 5.0 mL total) and PTSA (431 mg, 2.5 mmol, 10 equiv.) was added as a single portion. The reaction was left to stir (rt, 2 h) before quenching with $NaHCO_3$ (aq. sat. 10 mL). The aqueous phase was extracted with Et_2O (3 × 10 mL), and the combined organic layers dried ($MgSO_4$) and concentrated under reduced pressure. The crude material was purified via column chromatography on an automated system (BIOTAGE 10 g, 1-10%, Et_2O/pentane) to afford enone **8g** (53.4 mg, 75%) as a colourless oil.

R*f* = 0.28 (5% Et_2O/pentane)

^{1}H NMR (400 MHz, Chloroform-*d*) δ_H 7.26 – 7.19 (m, 2H), 7.19 – 7.12 (m, 3H), 6.72 (s, 1H), 6.71 (s, 1H), 3.13 – 3.05 (m, 2H), 2.92 (m, 2H).

^{13}C NMR (101 MHz, Chloroform-*d*) δ_C 194.2, 140.7, 137.7, 128.7 (2 × C), 128.5 (2 × C), 126.5, 112.8, 38.6, 30.7.

HRMS (EI): m/z calc'd for $C_{11}H_{11}IO$ $[M]^+$ requires 285.9849, found 285.9846.

IR (film): ν_{max} 2931, 1721, 1454, 1266, 732, 699 cm^{-1}

(4-iodo-3,3-dimethoxypent-4-en-1-yl)benzene (9)

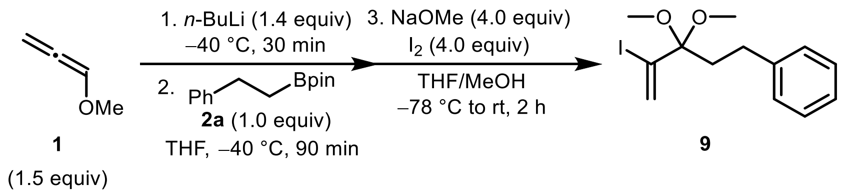

Methoxyallene (**1**) (126 µL, 1.5 mmol, 1.5 equiv.) was cooled (−40 °C) in anhydrous THF (12 mL). n-Butyllithium (1.6 M, 1.4 mmol, 1.4 equiv.) was added dropwise and the reaction stirred (30 min, −40 °C). 4,4,5,5-tetramethyl-2-phenethyl-1,3,2-dioxaborolane (**2a**) (232 mg, 1.0 mmol, 1.0 equiv.) was added dropwise as a solution in anhydrous THF (1 mL) and the flask walls washed with further anhydrous THF (1 mL, 14 mL total). The reaction was left to stir (−40 °C, 90 min) and then cooled (−78 °C) followed by the addition of NaOMe as a 0.5 M solution in MeOH (8.0 mL, 4.0 mmol, 4.0 equiv.) and I_2 (1.02 g, 4.0 mmol, 4.0 equiv.) as a 0.5 M solution in MeOH. The reaction was left to stir (−78 °C, 30 min) and then warmed and stirred (rt, 2 h). The reaction was quenched with sodium thiosulfate (aq. sat. 20 mL) and the aqueous layer extracted (Et_2O, 3 × 20 mL). The combined organic layers were dried ($MgSO_4$) and concentrated under reduced pressure. The crude compound was purified by column chromatography on an automated system (BIOTAGE 25 g, 0-4% Et_2O/pentane) to afford acetal **9** (262 mg, 79%) as a colourless oil.

R$_f$ = 0.46 (2% Et_2O/pentane)

^{1}H NMR (400 MHz, Chloroform-*d*) δ_H 7.31 – 7.26 (m, 2H), 7.22 – 7.17 (m, 3H), 6.76 (d, *J* = 1.1 Hz, 1H), 6.20 (d, *J* = 1.1 Hz, 1H), 3.24 (s, 6H), 2.49 – 2.40 (m, 2H), 2.17 – 2.08 (m, 2H).

^{13}C NMR (101 MHz, Chloroform-*d*) δ_C 141.6, 131.5, 128.6 (2 × C), 128.4 (2 × C), 126.1, 111.3, 102.7, 49.5 (2 × C), 35.0, 29.6.

HRMS (EI): m/z calc'd for $C_{13}H_{17}IO_2$ [M − MeOH]$^+$ requires 300.0006, found 300.0000.

IR (film): ν_{max} 2958, 2934, 1773, 1608, 1497, 1057, 1045, 698 cm^{-1}

(3,3-dimethoxypent-4-yn-1-yl)benzene (10)

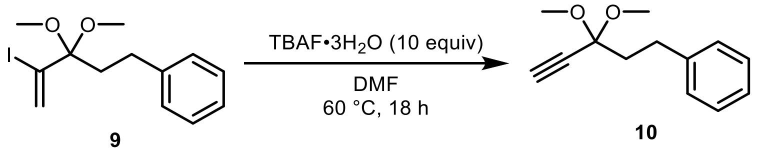

(4-iodo-3,3-dimethoxypent-4-en-1-yl)benzene (**9**) (200 mg, 0.60 mmol, 1.0 equiv.) and TBAF•3H_2O (1.89 g, 6.0 mmol, 10 equiv.) were added to anhydrous DMF (7.5 mL, 0.08 M) and the reaction left to stir (18 h, 60 °C). The reaction was permitted to cool (rt) and diluted with H_2O (10 mL). The aqueous layer was extracted with Et_2O (3 × 10 mL) and the combined organic layers dried ($MgSO_4$) and concentrated under reduced pressure. The crude material was purified by column chromatography on an automated system (BIOTAGE 25 g, 2-5% Et_2O/pentane) to afford alkyne **10** (108 mg, 88%) as a colourless oil.

R$_f$ = 0.22 (2% Et_2O/pentane)

^{1}H NMR (400 MHz, Chloroform-*d*) δ_H 7.35 – 7.24 (m, 2H), 7.27 – 7.15 (m, 3H), 3.36 (s, 6H), 2.87 – 2.79 (m, 2H), 2.62 (s, 1H), 2.16 – 2.08 (m, 2H).

^{13}C NMR (101 MHz, Chloroform-*d*) δ_C 141.7, 128.6 (2 × C), 128.5 (2 × C), 126.1, 99.0, 80.2, 74.0, 50.2 (2 × C), 39.0, 30.6.

HRMS (EI): m/z calc'd for $C_{13}H_{16}O_2$ [M − H]$^+$ requires 203.1067, found 203.1064.

IR (film): ν_{max} 2962, 2940, 1497, 1104, 1071, 698 cm^{-1}

5-phenylpent-1-yn-3-one (11)

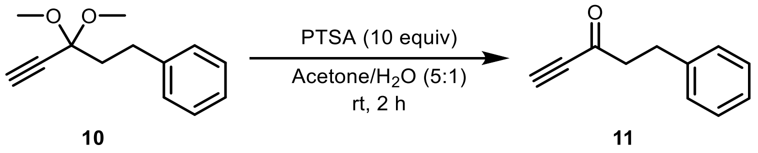

(3,3-dimethoxypent-4-yn-1-yl)benzene (**10**) (40.9 mg, 0.20 mmol, 1.0 equiv.) was stirred (rt, 2 h) with PTSA (344 mg, 2.0 mmol, 10 equiv.) in acetone/H_2O (5:1, 4.0 mL total). The reaction was quenched with $NaHCO_3$ (5 mL), and the aqueous layer extracted with Et_2O (3 × 10 mL). The combined organic layers were dried ($MgSO_4$)

and concentrated under reduced pressure. The crude material was purified by column chromatography on an automated system (BIOTAGE 10 g, 1-10% Et_2O/Pentane) to afford alkyne **11** (28.9 mg, 91%) as a colourless oil.

R*f* = 0.37 (5% Et_2O/pentane)

^1H and ^{13}C NMR data in accordance with the literature.[28]

^1H NMR (400 MHz, Chloroform-*d*) δ_H 7.30 – 7.27 (m, 2H), 7.25 – 7.16 (m, 3H), 3.25 (s, 1H), 3.04 – 2.98 (m, 2H), 2.97 – 2.90 (m, 2H).

^{13}C NMR (101 MHz, Chloroform-*d*) δ_C 186.4, 140.1, 128.7 (2 × C), 128.5 (2 × C), 126.5, 81.4, 79.0, 47.0, 29.7.

((E)-(3,3-dimethoxypent-1-ene-1,5-diyl)dibenzene (12)

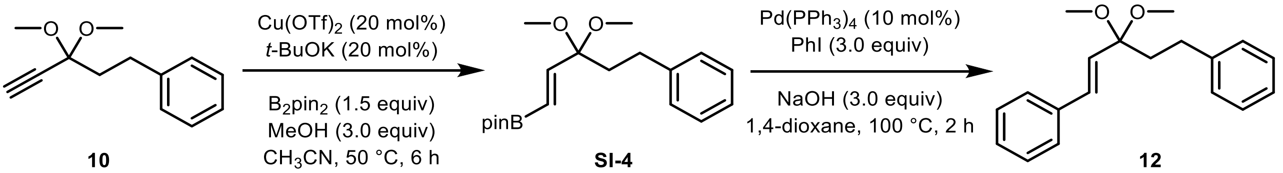

10 **SI-4** **12**

Prepared according to modified literature procedures.[29, 30]

$Cu(OTf)_2$ (10.9 mg, 0.03 mmol, 20 mol%), *t*-BuOK (3.37 mg, 0.03 mmol, 20 mol%), and bis(pinacolato)diboron (57.1 mg, 0.23 mmol, 1.5 equiv.) were added to a flame dried flask and the flask evacuated and backfilled with nitrogen (× 3). Anhydrous MeCN (1.0 mL) was added, followed by anhydrous MeOH (18 µL, 0.22 mmol, 3.0 equiv.). The contents of the vial was left to stir (rt, 30 min) before (3,3-dimethoxypent-4-yn-1-yl)benzene (**10**) (30.6 mg, 0.15 mmol, 1.0 equiv.) was added as a solution in anhydrous CH_3CN (0.50 mL). The reaction was heated (50 °C) and left to stir (6 h). After 6 h, the reaction was cooled (rt) and diluted with H_2O (5 mL). The aqueous phase was extracted with ethyl acetate (3 × 5 mL), and the combined organic layers dried ($MgSO_4$) and concentrated under reduced pressure. Compound **SI-4** was found to be highly unstable to silica gel. A quantitative ^1H NMR spectrum in the presence of CH_2Br_2 (1.0 equiv.) as an internal standard showed compound **SI-4** had formed in 74% NMR yield.

Crude ^1H NMR (400 MHz, Chloroform-*d*) δ_H 7.17 (m, 2H), 7.12 – 7.04 (m, 3H), 6.32 (d, J = 18.3 Hz, 1H), 5.81 (d, J = 18.3 Hz, 1H), 3.11 (s, 6H), 2.50 – 2.38 (m, 2H), 1.94 – 1.88 (m, 2H). Pinacol protons could not be assigned.

Crude ^{13}C NMR (101 MHz, Chloroform-*d*) δ_C 142.1, 128.5, 128.4, 125.9, 101.8, 83.5, 48.9, 36.7, 29.8.

Carbon bonded to boron not present due to quadrupolar relaxation.

HRMS (ESI): m/z calc'd for $C_{18}H_{25}BO_3$ [M − MeOH]$^+$ requires 300.1891, found 300.1887.

Crude compound **SI-4** was redissolved in anhydrous 1,4-dioxane (0.5 mL) and $Pd(PPh_3)_4$ (17.3 mg, 0.015 mmol, 10 mol%) was added followed by iodobenzene (50 µL, 0.45 mmol, 3.0 equiv). Aqueous 2 M NaOH (0.23 mL, 0.45 mmol, 3.0 equiv.) was added and the reaction was heated and left and to stir (100 °C, 2 h). The reaction was cooled (rt) and diluted with water (5 mL) and the aqueous phase extracted with Et_2O (3 × 5 mL). The

combined organic layers were dried ($MgSO_4$) and concentrated under reduced pressure. The crude residue was purified by column chromatography with neutralised silica (10% Et_2O/pentane, 2% NEt_3) to afford acetal **12** as a colourless oil (21.3 mg, 49% over 2 steps).

R_f = 0.60 (10% Et_2O/pentane)

^{1}H NMR (400 MHz, Chloroform-*d*) δ_H 7.47 – 7.43 (m, 2H), 7.37 – 7.33 (m, 2H), 7.29 – 7.25 (m, 3H), 7.21 – 7.14 (m, 3H), 6.83 (d, *J* = 16.2 Hz, 1H), 6.07 (d, *J* = 16.2, 1H), 3.26 (s, 6H), 2.64 – 2.57 (m, 2H), 2.13 – 2.05 (m, 2H).

^{13}C NMR (151 MHz, Chloroform-*d*) δ_C 142.1, 136.6, 133.2, 129.3, 128.8, 128.5, 128.4, 128.1, 126.8, 125.9, 102.0, 48.8, 37.6, 30.1.

HRMS (EI): m/z calc'd for $C_{18}H_{18}O$ [M − OMe]$^+$ requires 251.1430, found 251.1425

IR (film): ν_{max} 3025, 2952, 1496, 1449, 1128, 1053, 745, 694 cm^{-1}

N.B. Compound **12** was found to be highly sensitive to acid, base treated chloroform was necessary to avoid hydrolysis when collecting NMR data.

2.5. Total Synthesis of 10-Deoxymethynolide

methyl (*S*)-3-hydroxy-2-methylpropanoate (14)

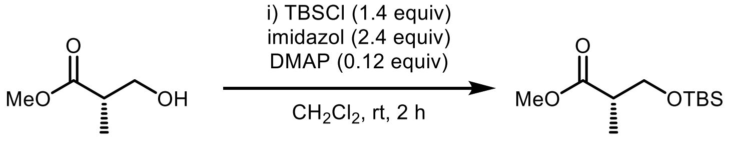

(*S*)-Roche Ester (**13**) **14**

Prepared according to a literature procedure.[31]

(*S*)-Roche ester (**13**) (5.0 g, 42.3 mmol, 1.0 equiv.), imidazole (6.9 g, 101.6 mmol, 2.4 equiv.), and DMAP (0.60 g, 5.1 mmol, 0.12 equiv.) was cooled to 0 °C in anhydrous CH_2Cl_2 (42.3 mL). *tert*-Butyldimethylsilyl chloride (TBSCl) (8.9 g, 59.3 mmol, 1.4 equiv.) was added and the reaction allowed to warm to room temperature. The mixture was stirred (rt, 2 h) before $NaHCO_3$ (aq. sat., 50 mL) was added. The phases were separated, and the aqueous phase extracted with CH_2Cl_2 (3 × 50 mL). The combined organic phase was dried ($MgSO_4$) and concentrated under reduced pressure. The resulting residue was purified by column chromatography (5% EtOAc/petroleum ether) to afford ester **14** as a colourless oil (6.96 g, 70%).

^{1}H and ^{13}C NMR data in accordance with the literature.[31]

R_f = 0.28 (5% EtOAc/petroleum ether)

^{1}H NMR (400 MHz, Chloroform-*d*) δ_H 3.76 (dd, *J* = 9.7, 6.9, 1H), 3.67 (s, 3H), 3.66 – 3.61 (dd, *J* = 9.7, 6.9 Hz, 1H), 2.63 (pd, *J* = 7.0, 5.9 Hz, 1H).1.13 (d, *J* = 7.0 Hz, 3H), 0.88 (s, 9H), 0.03 (s, 6H).

^{13}C NMR (101 MHz, Chloroform-*d*) δ_C 175.6, 65.4, 51.6, 42.7, 25.9 (3 × C), 18.3, 13.6, -5.4 (2 × C).

(*R*)-2-methyl-3-((trimethylsilyl)oxy)propan-1-ol (15)

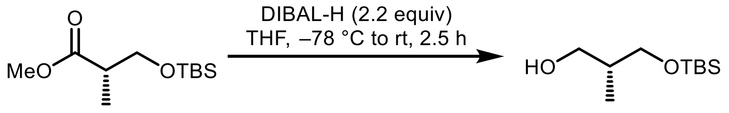

14 **15**

Prepared according to a literature procedure.[31]

Ester **14** (3.0 g, 12.9 mmol, 1.0 equiv.) was dissolved in anhydrous THF (29.0 mL, 0.45 M) in a flame dried flask under an inert atmosphere. The solution was cooled (−78 °C) and DIBAL-H (28.4 mL, 28.4 mmol, 2.2 equiv., 1 M in hexanes) was added dropwise via syringe pump over 20 min. The reaction was stirred (−78 °C, 2 h) before being permitted to warm to room temperature and stirring for a further 30 min. Magnesium sulfate was added to the reaction, and the mixture stirred (rt, 15 min) followed by filtering over celite (eluting with 20% EtOAc/petroleum ether). The crude alcohol **15** was concentrated under reduced pressure to give a colourless oil (2.44 g, 93%), which was deemed suitable for use in the next step without further purification.

^{1}H and ^{13}C NMR data in accordance with the literature.[31]

^{1}H NMR (400 MHz, Chloroform-*d*) δ_H 3.73 (dd, J = 9.9, 4.5 Hz, 1H), 3.67 – 3.57 (m, 2H), 3.54 (dd, J = 9.9, 7.9 Hz, 1H), 2.89 (s (br), 1H) 2.01 – 1.86 (m, 1H), 0.89 (s, 8H), 0.83 (d, J = 7.0 Hz, 3H), 0.07 (s, 6H).

^{13}C NMR (101 MHz, Chloroform-*d*) δ_C 68.9, 68.5, 37.1, 26.0 (3 × C), 18.3, 13.2, -5.5 (2 × C).

(*R*)-3-((tert-butyldimethylsilyl)oxy)-2-methylpropyl 2,4,6-triisopropylbenzoate (16)

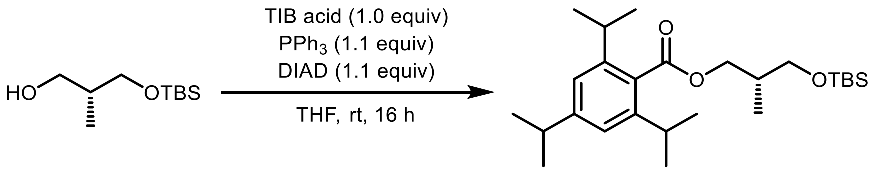

15 **16**

Prepared according to a literature procedure.[32]

Alcohol **15** (1.51 g, 7.35 mmol, 1.1 equiv.), triphenylphosphine (PPh_3) (1.94 g, 7.35 mmol. 1.1 equiv.), and 2,4,6-triisopropylbenzoic acid (1.65 g, 6.69 mmol, 1.0 equiv.) were cooled (0 °C) in anhydrous THF (30 mL) under an inert atmosphere. Diisopropyl azodicarboxylate (DIAD) (1.45 mL, 7.35 mmol, 1.1 equiv.) was added dropwise via syringe pump over 10 min. The mixture was allowed to warm to room temperature and was left to stir (16 h). The reaction was quenched with water, and the organic phase separated. The aqueous phase was extracted with CH_2Cl_2 (3 × 50 mL), and the combined organic phases dried ($MgSO_4$). The crude material was concentrated under reduced pressure and purified via column chromatography on an automated system (BIOTAGE 100 g, 12-50% toluene/pentane) to afford benzoate **16** as a colourless oil (1.92 g, 60%).

R_f = 0.40 (50% toluene/pentane)

$[\boldsymbol{\alpha}]_{\boldsymbol{D}}^{\boldsymbol{25}}$: +2 (c = 1)

^{1}H and ^{13}C NMR data in accordance with the literature.[32]

^{1}H NMR (400 MHz, Chloroform-*d*) δ_H 7.01 (s, 2H), 4.30 (dd, *J* = 10.8, 6.0 Hz, 1H), 4.26 – 4.16 (dd, *J* = 10.8, 5.9 Hz, 1H), 3.56 (d, *J* = 5.8 Hz, 2H), 2.97 – 2.77 (m, 3H), 2.13 – 1.98 (m, 1H), 1.25 (m, 18H), 0.99 (d, *J* = 6.9 Hz, 3H), 0.90 (s, 9H), 0.04 (s, 6H).

^{13}C NMR (101 MHz, Chloroform-*d*) δ_C 171.3, 150.1, 144.9, 130.9, 121.0, 67.1, 64.8, 35.6, 34.6, 31.7, 26.0, 24.3, 24.1, 18.4, 14.0, -5.3.

tert-butyl((2*S*,3*S*)-3-(dimethyl(phenyl)silyl)-2-methyl-3-(4,4,5,5-tetramethyl-1,3,2-dioxaborolan-2-yl)propoxy)dimethylsilane (17)

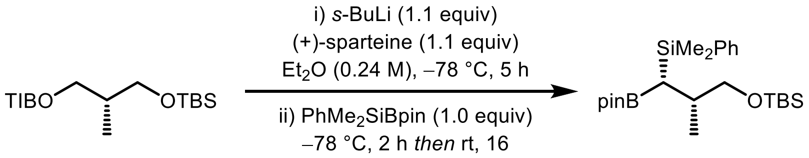

16 **17**

Prepared according to a literature review procedure.[31]

In a flame dried Schlenk flask under a nitrogen atmosphere, Benzoate **16** (1.0 g, 2.3 mmol, 1.1 equiv.) and (+)-sparteine (0.53 mL, 2.3 mmol, 1.1 equiv.) were cooled (−78 °C) in anhydrous Et_2O (9.6 mL. 0.24 M). *s*-BuLi (2.3 mmol, 1.1 equiv.,1.3 M in hexanes) was added dropwise via syringe pump and the reaction left to stir (−78 °C, 5 h). (Dimethylphenylsilyl)boronic acid pinacol ester (0.57 mL, 2.1 mmol, 1.0 equiv.) was added dropwise and the reaction left to stir (−78 °C, 2 h). The reaction was allowed to warm and stirred (rt, 16 h). The reaction was diluted (Et_2O, 20 mL) and the organic phase washed with HCl (2 M, 2 × 30 mL). The phases were separated, and the aqueous phase was extracted with Et_2O (3 × 30 mL). The combined organic phase was dried over magnesium sulfate and the residue concentrated under reduced pressure. The crude material was purified via prep HPLC (eluting in 0-2% EtOAc/hexane) to afford the pure boronic ester **17** as a colourless oil and as a single diastereomer (668.4 mg, 71%).

$\mathbf{R}_f$ = 0.52 (2% EtOAc/petroleum ether)

$[\alpha]_D^{25}$: −5 (c = 0.8)

^{1}H and ^{13}C NMR data in accordance with the literature.[31]

^{1}H NMR (400 MHz, Chloroform-*d*) δ_H 7.55 (m, 2H), 7.35 – 7.28 (m, 3H), 3.58 (dd, *J* = 9.7, 3.9 Hz, 1H), 3.22 (t, *J* = 9.5 Hz, 1H), 2.01 – 1.83 (m, 1H), 1.17 (s, 6H), 1.12 (s, 6H), 0.89 (d, *J* = 6.6 Hz, 3H), 0.86 (s, 9H), 0.62 (d, *J* = 8.4 Hz, 1H), 0.35 (s, 3H), 0.34 (s, 3H), -0.01 (s, 3H), -0.02 (s, 3H).

^{13}C NMR (101 MHz, Chloroform-*d*) δ_C 140.1, 134.0 (2 × C), 128.8, 127.7 (2 × C), 82.9 (2 × C), 69.7, 34.8, 26.1 (3 × C), 25.3 (2 × C), 25.2 (2 × C), 20.5, 18.5, -1.1 (2 × C) -5.1, -5.2.

(*S*)-1-(trimethylstannyl)ethyl 2,4,6-triisopropylbenzoate (18)

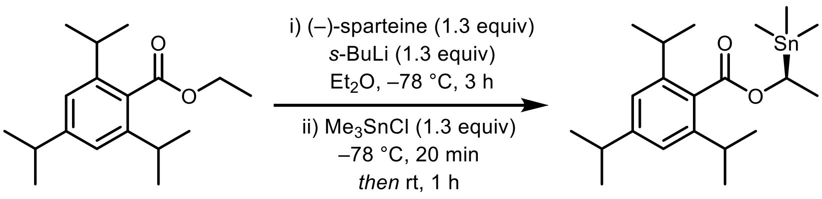

SI-1 **18**

Prepared according to a literature procedure.[2]

To a flame dried Schlenk flask under an inert atmosphere was added ethyl 2,4,6-triisopropylbenzoate (**SI-1**) (10 g, 36.2 mmol, 1.0 equiv.) and (−)-sparteine (10.8 mL, 47.0 mmol, 1.3 equiv.) followed by anhydrous Et_2O (200 mL). The mixture was cooled (−78 °C) followed by dropwise addition of *s*-BuLi (47.0 mmol, 1.3 equiv., 1.3 M in hexanes). The reaction was allowed to stir (−78 °C, 3 h), before the dropwise addition of Me_3SnCl (47 mL, 47.0 mmol, 1.3 equiv., 1.0 M in hexanes). The mixture was stirred (−78 °C, 20 min) and then allowed to warm to room temperature and stirred for a further hour. The mixture was diluted with 2 M HCl (150 mL) and stirred for 20 min. The organic phase was separated and washed with HCl (2 M, 3 × 150 mL). The combined aqueous phase was extracted with Et_2O (3 × 150 mL) and the resulting combined organic phases dried over magnesium sulfate. The residue was concentrated under reduced pressure. To the crude stannane MeOH was added (2 mL per gram) and an air condenser was fitted. The mixture was gently warmed until no solid remained. The mixture was left to **slowly** cool to room temperature. Crystals of stannane appeared after leaving the solution for multiple hours. The MeOH was removed, and the process repeated with fresh MeOH a further 4 times. With each iteration the speed at which the solid stannane crystallised increased until this process required < 10 min. The pure crystals were dried under vacuum to afford stannane **18** (5.91 g, 37%, 99.8:0.2 e.r.). Any uncrystallised product was stored in a freezer for later use. The enantiomeric ratio of the pure stannane was determined via chiral HPLC analysis.

^{1}H and ^{13}C NMR data in accordance with the literature.[2]

^{1}H NMR (400 MHz, Chloroform-*d*) δ_H 6.99 (s, 2H), 5.04 (q, *J* = 7.6 Hz, 1H), 2.86 (h, *J* = 6.8, 1H), 2.85 (h, *J* = 6.9 Hz, 2H) 1.59 (d, *J* = 7.5 Hz, 3H), 1.24 (d, *J* = 6.9 Hz, 18H), 0.18 (s, 9H).

^{13}C NMR (101 MHz, Chloroform-*d*) δ_C 171.4, 150.1, 144.9, 130.9, 120.9, 67.2, 34.5, 31.5, 24.5, 24.2, 24.1, 19.4, -9.8.

Chiral HPLC (Chiralpak IA column, 100% hexane, flow rate 0.5 mL min^{-1}, λ = 210 nm, rt): 24.8 min (major), 27.5 min (minor), 99.8:0.2

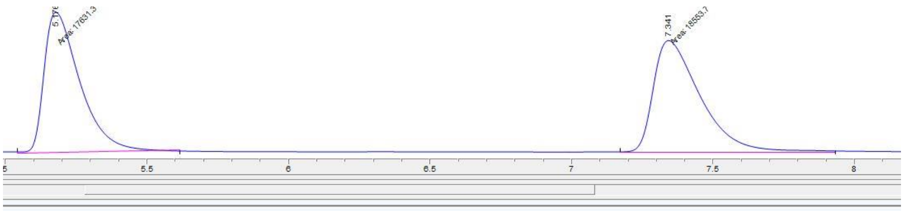

#	Time	Type	Area	Height	Width	Area%	Symmetry
1	5.176	MM	17631.3	1997.5	0.1471	48.725	0.475
2	7.341	MM	18553.7	1592.6	0.1942	51.275	0.46

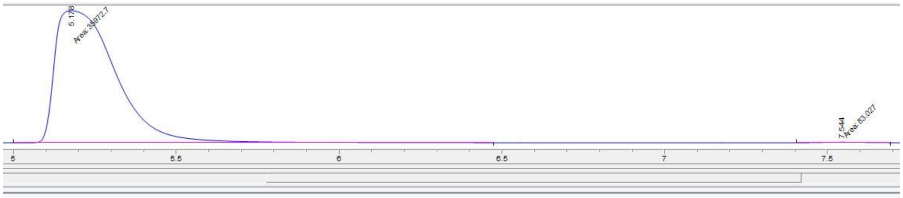

#	Time	Type	Area	Height	Width	Area%	Symmetry
1	5.178	MM	35972.7	2738.6	0.2189	99.770	0.374
2	7.544	MM	83	9.3	0.1484	0.230	1.039

tert-butyl(((2S,4R)-3-(dimethyl(phenyl)silyl)-2-methyl-4-(4,4,5,5-tetramethyl-1,3,2-dioxaborolan-2-yl)pentyl)oxy)dimethylsilane (20)

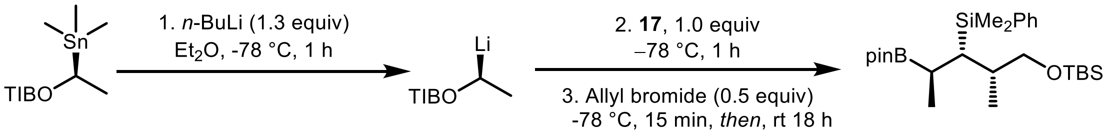

18 ***Li*-18** **20**

To a flame dried Schlenk flask under an inert atmosphere, (*S*)-1-(trimethylstannyl)ethyl 2,4,6-triisopropylbenzoate (**18**) (571 mg, 1.3 mmol, 1.3 equiv.) was cooled (−78 °C) in anhydrous Et_2O (6.5 mL, 0.2 M). *n*-BuLi (1.3 equiv., 1.6 M in hexanes) was added dropwise, and the solution left to stir (−78 °C, 1 h). Boronic ester **17** (450 mg, 1.0 mmol, 1.0 equiv.) was added dropwise to the mixture and the reaction was left to stir (−78 °C, 1 h). Allyl bromide (43 μL, 0.50 mmol, 0.50 equiv.) was added dropwise and the reaction stirred (−78 °C, 15 min), before allowing to warm with further stirring (rt, 18 h). The reaction was diluted with Et_2O (10 mL) and filtered through a pad of silica (eluting with Et_2O). The residue was concentrated under reduced pressure and the crude material purified via prep HPLC (eluting in 0-20% EtOAc/hexane) to afford boronic ester **20** (411.4 mg, 86%) as a colourless oil.

R_f = 0.27 (2% EtOAc/petroleum ether)

^{1}H and ^{13}C NMR data in accordance with the literature.[31]

^{1}H NMR (400 MHz, Chloroform-*D*) δ_H 7.58 – 7.51 (m, 2H), 7.32 (m, 3H), 3.49 (dd, *J* = 9.3, 4.1 Hz, 1H), 3.03 (*apparent* t, *J* = 9.8 Hz, 1H), 1.88–1.77 (m, 1H), 1.31 (qd, *J* = 7.5, 2.9 Hz, 1H), 1.22 (s, 13H), 0.99 (d, *J* = 7.6 Hz, 3H), 0.96 (d, *J* = 6.7 Hz, 3H), 0.84 (s, 9H), 0.40 (s, 3H), 0.33 (s, 3H), -0.05 (s, 3H), -0.06 (s, 3H).

^{13}C NMR (101 MHz, Chlorofom-*D*) δ 141.2, 134.1 (2 × C), 128.6, 127.7 (2 × C), 83.2 (2 × C), 68.8, 37.1, 30.5, 26.1 (3 × C), 25.0 (4 × C), 18.5 (2 × C), 14.1, -0.5, -0.6, -5.1 (2 × C).

tert-butyl(((2S,3S,4S,6R)-3-(dimethyl(phenyl)silyl)-2,4-dimethyl-6-(4,4,5,5-tetramethyl-1,3,2-dioxaborolan-2-yl)heptyl)oxy)dimethylsilane (22)

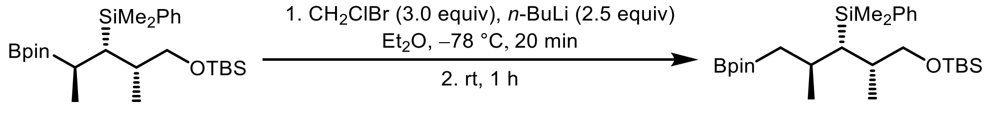

20 **SI-5**

To a flamed dried Schlenk flask under an inert atmosphere, boronic ester **20** (469 mg, 0.980 mmol, 1.0 equiv.) and CH_2ClBr (0.19 mL, 2.97 mmol, 3.0 equiv.) were cooled (−78 °C) in anhydrous Et_2O (5.0 mL, 0.2 M). *n*-BuLi (2.5 equiv., 1.6 M in hexanes) was added dropwise, and the mixture was left to stir (−78 °C, 20 min). The mixture was allowed to warm to room temperature and stirred for a further hour. The reaction was then diluted with Et_2O (10 mL) and filtered through a pad of silica (eluting with Et_2O). The residue was concentrated under reduced pressure to afford crude boronic ester **SI-5** which was used directly in the next step without further purification.

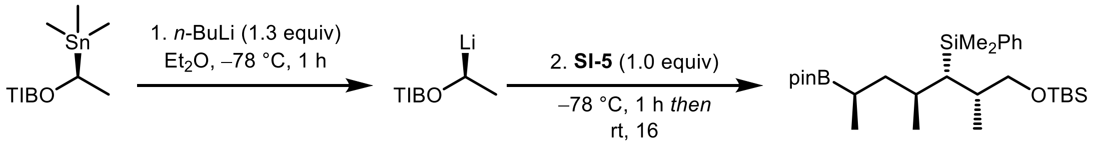

18 (1.3 eq) ***Li*-18** **22**

To a flame dried Schlenk flask under an inert atmosphere, (*S*)-1-(trimethylstannyl)ethyl 2,4,6-triisopropylbenzoate (**18**) (566 mg, 1.29 mmol, 1.3 equiv.) was cooled (−78 °C) in anhydrous Et_2O (6.5 mL, 0.2 M). *n*-BuLi (1.3 equiv.,1.6 M in hexanes) was added dropwise, and the solution left to stir (−78 °C, 1 h). Crude boronic ester **SI-5** (assumed 0.980 mmol, 1.0 equiv.) was added dropwise to the mixture and reaction was left to stir (−78 °C, 1 h) before allowing to warm to room temperature and leaving to stir (16 h). The mixture was diluted with Et_2O (10 mL) and filtered through a pad of silica (eluting with Et_2O). The residue was concentrated under reduced pressure and the crude material was purified via prep HPLC (eluting in 2% EtOAc/hexane) to afford boronic ester **22** as a colourless oil (337 mg, 66% over two steps).

$\mathbf{R_f}$ = 0.32 (50% toluene/pentane)

$[\boldsymbol{\alpha}]_{\boldsymbol{D}}^{\mathbf{25}}$: +8 (c = 0.25)

^{1}H NMR (400 MHz, Chloroform-*d*) δ_H 7.56 – 7.46 (m, 2H), 7.31 (m, 3H), 3.24 (dd, *J* = 9.6, 6.4 Hz, 1H), 3.11 (*apparent* t, *J* = 9.2 Hz, 1H), 1.99 – 1.81 (m, 2H), 1.44 (ddd, *J* = 13.5, 8.2, 6.3 Hz, 1H), 1.28 – 1.17(m ,13H), 1.06 (m, 1H), 1.05 – 0.95 (m, 1H), 0.98 (d, *J* = 7.3 Hz, 3H) 0.89 (d, *J* = 6.9 Hz, 3H), 0.86 (s, 9H), 0.78 (d, *J* = 7.3 Hz, 3H), 0.39 (s, 3H), 0.35 (s, 3H), -0.04 (s, 3H), -0.05 (s, 3H).

^{13}C NMR (101 MHz, Chloroform-*d*) δ_C 141.1, 134.1 (2 × C), 128.7, 127.8 (2 × C), 82.8 (2 × C), 69.3, 41.0, 34.2, 33.1, 31.7, 26.2 (3 × C, 24.9 (2 × C), 24.8 (2 × C), 18.6, 18.5, 18.4, 15.7, -0.2, -0.5, -5.1 (2 × C).

Carbon bonded to boron not present due to quadrupolar relaxation.

HRMS (MALDI with Graphite): m/z calc'd for $C_{29}H_{55}BNaO_3Si_2$ $[M+Na]^+$ requires 541.3681, found 541.3688.

IR (film): ν_{max} 2953, 2928, 1462, 1378, 1370, 1314, 1249, 1145, 1109, 1090, 810, 774 cm^{-1}

(4*R*,6*S*,7*S*,8*S*)-9-((tert-butyldimethylsilyl)oxy)-7-(dimethyl(phenyl)silyl)-4,6,8-trimethylnon-1-en-3-one (24)

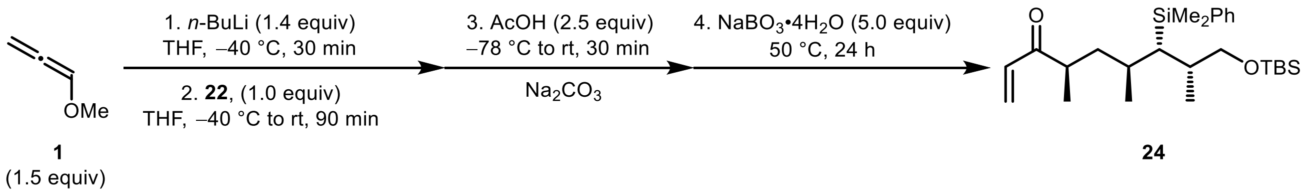

Methoxyallene (**1**) (20 µL, 0.24 mmol, 1.5 equiv.) was cooled (−40°C) in anhydrous THF (2.0 mL). *n*-Butyllithium (0.22 mmol, 1.4 equiv., 1.60 M in hexanes) was added dropwise and the reaction left to stir (−40 °C, 30 min). Boronic ester **22** (82 mg, 0.16 mmol, 1.0 equiv.) was added dropwise in anhydrous THF (0.20 mL) and the flask walls washed with a further portion of anhydrous THF (0.2 mL, to give 2.4 mL total). The reaction was allowed to warm and stir (rt, 90 min), followed by cooling (−78 °C) and the dropwise addition of glacial AcOH (23 µL, 0.40 mmol, 2.5 equiv.). The reaction was allowed to warm and stir (rt, 30 min), followed by quenching with Na_2CO_3 (aq. sat., 0.4 mL). The reaction was heated (50 °C), and $NaBO_3$•$4H_2O$ (122 mg, 0.79 mmol, 5.0 equiv.) was added portion wise with vigorous stirring (24 h). After complete oxidation (monitored by TLC), the reaction was cooled (rt) and diluted with H_2O (10 mL). The aqueous layer was extracted with Et_2O (3 × 10 mL). The combined organic layers were dried ($MgSO_4$) and concentrated under reduced pressure. The crude enone was purified by column chromatography on an automated system (BIOTAGE 10 g, 12-50% toluene/pentane) to afford enone **24** (45.6 mg, 65%) as a yellow oil.

R_f = 0.38 (50% toluene/pentane)

$[\alpha]_D^{25}$: −12 (c = 0.50)

^{1}H NMR (400 MHz, Chloroform-*d*) δ_H 7.55 – 7.45 (m, 2H), 7.39 – 7.27 (m, 3H), 6.35 (dd, *J* = 17.4, 10.4 Hz, 1H), 6.20 (dd, *J* = 17.4, 1.6 Hz, 1H), 5.66 (dd, *J* = 10.4, 1.6 Hz, 1H), 3.22 (m, 2H), 2.70 (dt, *J* = 7.6, 6.6 Hz, 1H), 1.92 (qd, *J* = 7.3, 1.6 Hz, 1H), 1.79 (qd, *J* = 6.9, 1.7 Hz, 1H), 1.69 (dt, *J* = 13.8, 6.8 Hz, 1H), 1.19 (dt, *J* = 13.7, 6.8 Hz, 1H), 1.13 (m, 1H), 1.00 (d, *J* = 7.0 Hz, 3H), 0.91 (d, *J* = 7.0 Hz, 3H), 0.87 (s, 9H), 0.82 (d, *J* = 6.8 Hz, 3H), 0.39 (s, 3H), 0.37 (s, 3H), -0.02 (s, 3H), -0.03 (s, 3H).

^{13}C NMR (101 MHz, Chloroform-*d*) δ_C 204.2, 140.5, 134.9, 134.1 (2 × C), 128.8, 127.91, 127.86 (2 × C), 69.2, 41.8, 40.7, 34.0, 32.3, 30.7, 26.2 (3 × C), 19.0, 18.6, 18.5, 16.1, -0.1, -0.9, -5.2 (2 × C).

HRMS (ESI): m/z calc'd for $C_{26}H_{46}O_2Si_2$ $[M+Na]^+$ requires 469.2929, found 469.2916.

IR (film): ν_{max} 2955, 2856, 1698, 1678, 1612, 1461, 1380, 1249, 1109. 1086, 835, 702 cm^{-1}

(4*R*,6*S*,7*S*,8*S*)-7-(dimethyl(phenyl)silyl)-9-hydroxy-4,6,8-trimethylnon-1-en-3-one (25)

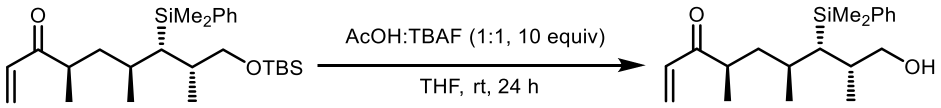

24 **25**

In a flame dried flask under an inert atmosphere anhydrous THF (0.43 mL) was added to enone **24** (15 mg, 0.034 mmol, 1.0 equiv.). An equimolar mixture of AcOH (20 µL, 0.34 mmol, 10.0 equiv.) and TBAF (0.34 mL, 0.34 mmol, 1.0 M in THF, 10.0 equiv.) was added dropwise, and the reaction stirred (rt, 24 h). The reaction was diluted with H_2O (1.0 mL) and the aqueous layer extracted with Et_2O (5 × 1.0 mL). The combined organic layers were dried ($MgSO_4$) and concentrated under reduced pressure. The crude reaction mixture was purified by column chromatography (30% Et_2O/pentane) to afford alcohol **25** (9.2 mg, 81%) as a colourless oil.

R_f = 0.26 (30% Et_2O/pentane)

$[\alpha]_D^{25}$: −12 (c = 1)

^{1}H NMR (400 MHz, Chloroform-*D*) δ_H 7.56 – 7.48 (m, 2H), 7.37 – 7.30 (m, 3H), 6.40 (dd, *J* = 17.4, 10.4 Hz, 1H), 6.23 (dd, *J* = 17.4, 1.5 Hz, 1H), 5.70 (dd, *J* = 10.4, 1.5 Hz, 1H), 3.32 (dd, *J* = 10.3, 7.0 Hz, 1H), 3.24 (dd, *J* = 10.3, 7.7 Hz, 1H), 2.72 (dq, *J* = 14.3, 6.9 Hz, 1H), 1.92 (pt, *J* = 8.4, 4.2 Hz, 1H), 1.83 (pt, *J* = 8.4, 1.2 Hz, 1H), 1.74 (ddd, *J* = 13.9, 7.8, 6.2 Hz, 1H), 1.19 (m, 1H), 1.14 (ddd, *J* = 13.6, 7.7, 5.9 Hz, 1H), 1.02 (d, *J* = 6.9 Hz, 3H), 0.93 (d, *J* = 6.9 Hz, 3H), 0.83 (d, *J* = 6.8 Hz, 3H), 0.41 (s, 3H), 0.39 (s, 3H).

^{13}C NMR (101 MHz, Chloroform-*D*) δ_C 204.6, 140.4, 135.1, 133.99 (2 × C), 129.0, 128.2, 127.9 (2 × C), 68.8, 41.7, 40.2, 34.2, 32.1, 30.8, 19.5, 18.5, 16.3, -0.2, -0.9.

HRMS (ESI): m/z calc'd for $C_{20}H_{32}NaO_2Si$ $[M+Na]^+$ requires 355.2064, found 355.2076.

IR (film): ν_{max} 3451, 2957, 2926, 1695, 1612, 1459, 1427, 1403, 1110, 809, 702 cm^{-1}

(2*S*,3*S*,4*S*,6*R*)-3-(dimethyl(phenyl)silyl)-2,4,6-trimethyl-7-oxonon-8-enoic acid (26)

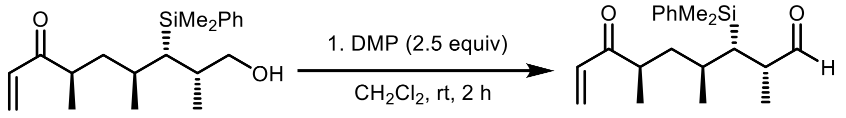

25 **SI-6**

Alcohol **25** (43.9 mg, 0.132 mmol, 1.0 equiv.) in anhydrous CH_2Cl_2 (13.2 mL, 0.01 M) was cooled (0 °C) and Dess–Martin periodinane (140 mg, 0.330 mmol, 2.5 equiv.) was added as a single portion. The reaction was allowed to warm and stirred (rt, 2 h), before quenching with $Na_2S_2O_3$ (aq. sat. 10 mL) and $NaHCO_3$ (aq. sat. 10 mL). The aqueous layer was extracted with hexane (3 × 10 mL) and the combined organic layers dried ($MgSO_4$) and concentrated under reduced pressure to afford aldehyde **SI-6** which was used directly in the next step without purification.

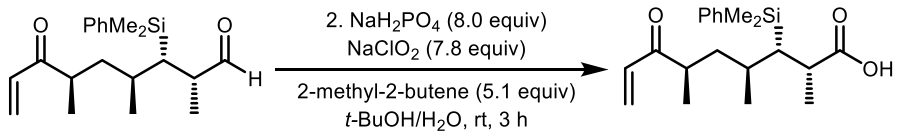

SI-6 **26**

To crude aldehyde **SI-6** (assumed 0.132 mmol) in *t*-BuOH (12 mL, 0.011 M) was added NaH_2PO_4 (127 mg, 1.06 mmol, 8.0 equiv.) and 2-methyl-2-butene (71 µL, 0.670 mmol, 5.1 equiv.). To this mixture, $NaClO_2$ (93.2 mg, 1.03 mmol, 8.0 equiv.) in H_2O (7.4 mL, 0.14 M) was added and the reaction left to stir (rt, 2 h). The reaction was the diluted with H_2O (10 mL) and the aqueous layer extracted with EtOAc (3 × 10 mL). The combined organic layers were dried ($MgSO_4$) and concentrated under reduced pressure. The crude material was purified by column chromatography on an automated system (BIOTAGE 10 g, 7-50% Et_2O/Pentane) to afford carboxylic acid **26** (36.8 mg, 80% over 2 steps) as a colourless oil.

R*f* = 0.21 (30% pentane)

$[\alpha]_D^{25}$: −26 (c = 1)

^{1}H NMR (400 MHz, Chloroform-*D*) δ_H 7.54 – 7.50 (m, 2H), 7.33 – 7.31 (m, 3H), 6.42 – 6.29 (dd, *J* = 17.5, 10.4 Hz, 1H), 6.21 (dd, *J* = 17.5, 1.5 Hz, 1H), 5.72 – 5.66 (dd, *J* = 10.4, 1.5 Hz, 1H), 2.81 – 2.71 (m, 2H), 1.84 – 1.70 (m, 3H), 1.24 (d, *J* = 7.1 Hz, 3H), 1.15 – 1.05 (m, 1H) 0.92 (d, *J* = 6.6 Hz, 3H), 0.77 (d, *J* = 6.8 Hz, 3H), 0.41 (s, 3H), 0.36 (s, 3H).

^{13}C NMR (101 MHz, Chloroform-*D*) δ_C 204.3, 183.5, 139.7, 135.2, 134.0 (2 × C), 129.1, 128.2, 127.9 (2 × C), 41.2, 39.9, 37.4, 33.4, 30.9, 19.2, 17.2, 16.2, -1.0, -1.5.

HRMS (−APCI): m/z calc'd for $C_{20}H_{30}O_3Si$ $[M-H]^-$ requires 345.1880, found 345.1869.

IR (film): ν_{max} 3336, 2959, 2923, 1701, 1458, 1110, 813, 766, 702 cm^{-1}

(3*R*,4*R*)-4-methylhex-5-en-3-yl(2*S*,3*S*,4*S*,6*R*)-3-(dimethyl(phenyl)silyl)-2,4,6-trimethyl-7-oxonon-8-enoate (28)

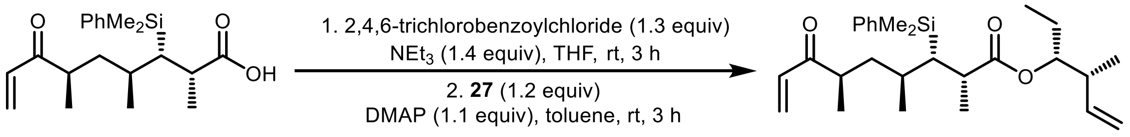

26 **28**

To carboxylic acid **26** (34.7 mg, 0.100 mmol, 1.0 equiv.) in anhydrous THF (1.52 mL), NEt_3 (20 μL, 0.140 mmol, 1.4 equiv.) was added followed by 2,4,6-trichlorobenzoylchloride (20 μL, 0.130 mmol, 1.3 equiv.). The reaction was allowed to stir (rt, 3 h), and then filtered over cotton (washing with 1.2 mL of hexane). The resulting mixture was concentrated and then redissolved in anhydrous toluene (0.43 mL). Alcohol **27** (13.7 mg, 0.120 mmol, 1.2 equiv.) was pre-mixed with DMAP (13.4 mg, 0.110 mmol, 1.1 equiv.) in anhydrous toluene (0.28 mL) and the solution added to the reaction. The reaction was left to stir (rt, 3 h) before diluting with Et_2O (1.0 mL) and washing with $NaHCO_3$ (2.0 mL) and brine (2.0 mL). The organic layer was dried ($MgSO_4$) and concentrated under reduced pressure. The crude material was purified by column chromatography (10% EtOAc/Hexane) to afford ester **28** (24.6 mg, 56%) as a colourless oil.

R_f = 0.45 (10% EtOAc/Hexane)

$[\alpha]_D^{25} = -4$ (*c* = 0.5)

^{1}H NMR (600 MHz, Chloroform-*D*) δ_H 7.57 – 7.51 (m, 2H), 7.33 (m, 3H), 6.37 (dd, *J* = 17.4, 10.5 Hz, 1H), 6.21 (dd, *J* = 17.4, 1.5 Hz, 1H), 5.76 (ddd, *J* = 17.5, 10.4, 7.4 Hz, 1H), 5.66 (dd, *J* = 10.5, 1.5 Hz, 1H), 5.08 – 5.01 (m, 2H), 4.74 (ddd, *J* = 7.7, 5.9, 4.3 Hz, 1H), 2.72 (m, 2H), 2.45 (dq, *J* = 14.0, 6.7 Hz, 1H), 1.81 – 1.75 (m, 2H), 1.71 (ddd, *J* = 13.7, 7.7, 5.9 Hz, 1H), 1.64 – 1.56 (m, 1H), 1.52 (dq, *J* = 14.6, 7.4 Hz, 1H), 1.25 (d, *J* = 7.0 Hz, 3H), 1.08 (ddd, *J* = 14.3, 8.2, 6.2 Hz, 1H), 1.02 (d, *J* = 6.9 Hz, 3H), 0.91 (d, *J* = 6.8 Hz, 3H), 0.85 (t, *J* = 7.5 Hz, 3H), 0.74 (d, *J* = 6.8 Hz, 3H), 0.42 (s, 3H), 0.36 (s, 3H).

^{13}C NMR (151 MHz, Chloroform-*D*) δ_C 204.1, 177.3, 140.1, 139.9, 135.1, 134.0 (2 × C), 129.0, 128.0, 127.9 (2 × C), 115.3, 78.4, 41.5, 40.7, 39.7, 37.6, 32.6, 30.8, 24.1, 19.2, 18.3, 15.8, 15.5, 9.9, -0.8, -1.3.

HRMS (ESI): m/z calc'd for $C_{27}H_{42}NaO_3Si$ $[M + Na]^+$ requires 465.2796, found 465.2791

IR (film): ν_{max} 3070, 2968, 2932, 1723, 1700, 1457, 1427, 1381, 1251, 1186, 1109, 915, 831, 813, 702 cm^{-1}

(3*S*,4*S*,5*S*,7*R*,11*R*,12*R*,E)-4-(dimethyl(phenyl)silyl)-12-ethyl-3,5,7,11-tetramethyloxacyclododec-9-ene-2,8-dione (29)

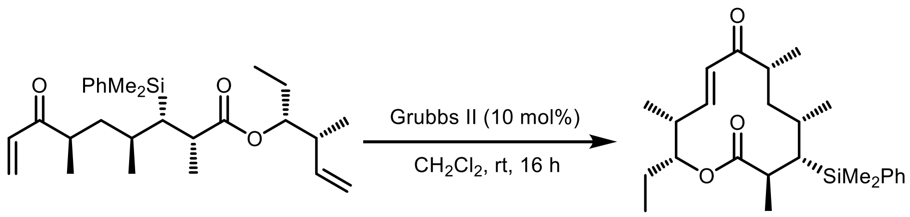

28 **29**

To a flame dried flask under an inert atmosphere, Grubbs II catalyst (4.70 mg, 0.00556 mmol, 10 mol%) was added to a solution of enone **28** (24.6 mg, 0.0556 mmol, 1.0 equiv.) in anhydrous CH_2Cl_2 (11.0 mL, 0.005 M). The reaction was left to stir (rt, 16 h) before concentrating under reduced pressure. The crude product was purified by column chromatography on an automated system (BIOTAGE 10 g, 3-30% EtOAc/Hexane) to afford cyclic enone **29** (18.6 mg, 81%) as an amorphous white solid.

R_f = 0.31 (15% EtOAc/Hexane)

$[\alpha]_D^{25} = +100$ (*c* = 0.5)

^{1}H NMR (400 MHz, Chloroform-*D*) δ_H 7.51 (m, 2H), 7.33 (m, 3H), 6.80 (dd, *J* = 15.8, 5.6 Hz, 1H), 6.38 (d, *J* = 15.8 Hz, 1H), 4.99 (ddd, *J* = 8.7, 5.4, 2.3 Hz, 1H), 2.71 – 2.50 (m, 3H), 1.78 – 1.61 (m, 2H), 1.60 – 1.46 (m, 2H), 1.43 – 1.37 (m, 2H), 1.21 (d, *J* = 7.0 Hz, 3H), 1.10 (d, *J* = 6.8 Hz, 3H), 0.93 (*apparent* t, *J* = 6.5 Hz, 6H), 0.87 (t, *J* = 7.4 Hz, 3H), 0.43 (s, 3H), 0.36 (s, 3H).

^{13}C NMR (101 MHz, Chloroform-*D*) δ_C 204.5, 176.9, 147.7, 141.1, 133.6 (2 × C), 128.8, 128.0 (2 × C), 125.7, 73.9, 45.6, 42.1, 37.9, 37.7, 36.7, 34.3, 25.5, 20.2, 20.1, 18.0, 10.5, 9.8, 2.4, -1.6.

HRMS (ESI): m/z calc'd $C_{25}H_{38}NaO_3Si$ $[M + Na]^+$ requires 437.2482, found 437.2477

IR (film): ν_{max} 3069, 2966, 2929, 1731, 1691, 1628, 1458, 1427, 1361, 1291, 1260, 1191, 1172, 1147, 1110, 982, 812, 702 cm^{-1}

10-Deoxymethynolide (30)

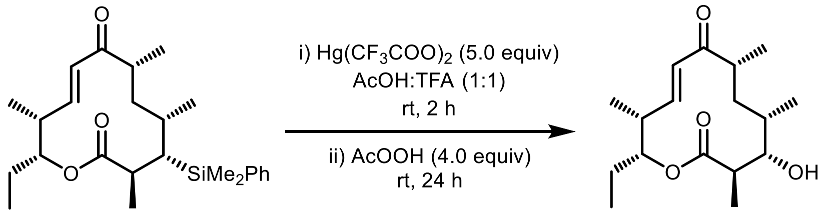

29 **10-Deoxymethynolide (30)**

To a solution of cyclic enone **29** (6.8 mg, 0.0164 mmol, 1.0 equiv.) in a 1:1 mixture of AcOH (0.27 mL) and TFA (0.27 mL), mercury (II) trifluoroacetate ($Hg(CF_3COO)_2$) (35 mg, 0.0820 mmol, 5.0 equiv.) was added. The reaction was allowed to stir (rt, 2 h) and followed by TLC (25% EtOAc/Hexane). After two hours a single spot was observed (R_F = 0.22), and the reaction was cooled (10 °C) and AcOOH (14 µL, 0.0656 mmol, 4.0 equiv.) was added dropwise. The reaction was stirred (10 °C, 10 min) and then allowed to warm and left to stir (rt, 24 h). The reaction was quenched (aq. sat. $Na_2S_2O_3$ 1.0 mL, and aq. sat. $NaHCO_3$ 1.0 mL) and the aqueous layer extracted (EtOAc, 5 × 1 mL). The combined organic layers were dried ($MgSO_4$) and concentrated under reduced pressure. The crude material was passed over a short plug of silica (eluting in 50% EtOAc/Hexane) and then purified by reverse phase prep HPLC (Phenomenex Kinetex column (2.6 µ, C18, 100 Å, 4.6 x 100 mm), 30-95% MeCN/H_2O, flow rate 1 ml/min, UV detection between 200 and 400 nm, retention time 9.6 min) to afford pure 10-deoxymethynolide (**30**) (1.6 mg, 34%) as a colourless oil.

N.B. Further information regarding reverse phase prep HPLC purification:

For analysis of assays, a sample was made up in HPLC-grade MeOH (1 mg of crude sample in 100 µL). 20 µL was injected onto a Waters 2795HT system equipped with the following analytical systems:

Waters 2795HT HPLC system: Waters 998 diode array detector for UV between 200 and 400 nm.

ELSD: Electrospray (ES) Waters ZQ mass spectrometry with detection between 150 and 800 m/z units in negative and positive modes.

Phenomenex Kinetex column (2.6 µ, C18, 100 Å, 4.6 x 100 mm) and a Phenomenex Security Guard column (Luna C5 300 Å). Flow rate was set at 1 mL/min and HPLC- Experimental 138 grade H2O (A) and acetonitrile (MeCN) (B) supplemented with 0.04 % formic acid (FA) were used as solvent.

30-95% gradient method: Solvent A (H_2O), Solvent B (MeCN)

Time (min)	Percentage B
0-1	5% B
1-2	Linear gradient to 30% B
2-15	Linear gradient to 95% B
15-17	95% B
17-18	Linear gradient from 95% B to 5% B
18-20	5% B

R_f = 0.49 (50% EtOAc/Hexane)

$[\alpha]_D^{25}$ = +20 (c = 0.15)

^{1}H and ^{13}C NMR data in accordance with the literature.[33, 34]

^{1}H NMR (600 MHz, Chloroform-*D*) δ_H 6.74 (dd, *J* = 15.7, 5.4 Hz, 1H), 6.42 (dd, *J* = 15.7, 1.4 Hz, 1H), 5.00 (ddd, *J* = 8.4, 5.4, 2.3 Hz, 1H), 3.56 (d, *J* = 10.4 Hz, 1H), 2.67 – 2.48 (m, 3H), 1.77 – 1.61 (m, 2H), 1.60 – 1.55 (m, 2H), 1.31 (d, *J* = 6.9 Hz, 3H), 1.33 – 1.26 (m, 2 H) 1.22 (d, *J* = 7.0 Hz, 3H), 1.12 (d, *J* = 6.8 Hz, 3H), 1.01 (d, *J* = 6.2 Hz, 3H), 0.91 (t, *J* = 7.4 Hz, 3H).

^{13}C NMR (151 MHz, Chloroform-*D*) δ_C 204.9, 174.7, 147.1, 125.6, 78.2, 73.7, 45.1, 43.3, 38.0, 33.2, 25.1, 17.7, 17.4, 16.4, 10.3, 9.5.

HRMS (ESI): m/z calc'd for $C_{17}H_{28}NaO_4$ $[M + Na]^+$ requires 319.1880, found 319.1877

IR (film): ν_{max} 3442 (br), 2963, 2926, 1729, 1688, 1629, 1459, 1378, 1085, 993, 807, 718 cm^{-1}

3. REFERENCES

[1] W.-Z. Weng, H. Liang, B. Zhang, *Org. Lett.* **2018**, *20*, 4979-4983.
[2] J. J. Rogers, V. K. Aggarwal, *Asian J. Org. Chem.* **2021**, *10*, 2338-2341.
[3] S. Aichhorn, R. Bigler, E. L. Myers, V. K. Aggarwal, *J. Am. Chem. Soc.* **2017**, *139*, 9519-9522.
[4] H. Wang, J. Wu, A. Noble, V. K. Aggarwal, *Angew. Chem. Int. Ed.* **2022**, *61*, e202202061.
[5] V. Fasano, N. Winter, A. Noble, V. K. Aggarwal, *Angew. Chem. Int. Ed.* **2020**, *59*, 8502-8506.
[6] R. Sang, A. Noble, V. K. Aggarwal, *Angew. Chem. Int. Ed.* **2021**, *60*, 25313-25317.
[7] A. Fawcett, J. Pradeilles, Y. Wang, T. Mutsuga, E. L. Myers, V. K. Aggarwal, *Science* **2017**, *357*, 283-286.
[8] R. Bigler, V. K. Aggarwal, *Angew. Chem. Int. Ed.* **2018**, *57*, 1082-1086.
[9] J. Schmidt, J. Choi, A. T. Liu, M. Slusarczyk, G. C. Fu, *Science* **2016**, *354*, 1265-1269.
[10] M. Odachowski, A. Bonet, S. Essafi, P. Conti-Ramsden, J. N. Harvey, D. Leonori, V. K. Aggarwal, *J. Am. Chem. Soc.* **2016**, *138*, 9521-9532.
[11] C. Sandford, R. Rasappan, V. K. Aggarwal, *J. Am. Chem. Soc.* **2015**, *137*, 10100-10103.
[12] D. J. Blair, C. J. Fletcher, K. M. P. Wheelhouse, V. K. Aggarwal, *Angew. Chem. Int. Ed.* **2014**, *53*, 5552-5555.
[13] A. Fawcett, A. Murtaza, C. H. U. Gregson, V. K. Aggarwal, *J. Am. Chem. Soc.* **2019**, *141*, 4573-4578.
[14] A. Fawcett, T. Biberger, V. K. Aggarwal, *Nature Chemistry* **2019**, *11*, 117-122.
[15] R. C. Mykura, S. Veth, A. Varela, L. Dewis, J. J. Farndon, E. L. Myers, V. K. Aggarwal, *J. Am. Chem. Soc.* **2018**, *140*, 14677-14686.
[16] H. C. Brown, K. S. Bhat, *J. Am. Chem. Soc.* **1986**, *108*, 5919-5923.
[17] J. Chengebroyen, M. Linke, M. Robitzer, C. Sirlin, M. Pfeffer, *J. Organomet. Chem.* **2003**, *687*, 313-321.
[18] K. R. Anderson, S. L. G. Atkinson, T. Fujiwara, M. E. Giles, T. Matsumoto, E. Merifield, J. T. Singleton, T. Saito, T. Sotoguchi, J. A. Tornos, E. L. Way, *Org. Process Res. Dev.* **2010**, *14*, 58-71.
[19] R. J. Armstrong, V. K. Aggarwal*, *Organic Syntheses* **2018**, 234-251.
[20] B. Rubial, B. S. L. Collins, R. Bigler, S. Aichhorn, A. Noble, V. K. Aggarwal, *Angew. Chem. Int. Ed.* **2019**, *58*, 1366-1370.
[21] K. R. Roesch, R. C. Larock, *J. Org. Chem.* **2001**, *66*, 412-420.
[22] N. Zhang, C. Zhang, X. Hu, X. Xie, Y. Liu, *Org. Lett.* **2021**, *23*, 6004-6009.
[23] H. Nemoto, T. Kawano, N. Ueji, M. Bando, M. Kido, I. Suzuki, M. Shibuya, *Org. Lett.* **2000**, *2*, 1015-1017.
[24] P. Dewi-Wülfing, S. Blechert, *Eur. J. Org. Chem.* **2006**, *2006*, 1852-1856.
[25] S. Wu, N. Yang, L. Yang, J. Cao, J. Liu, *J. Polym. Sci., Part A: Polym. Chem.* **2010**, *48*, 1441-1448.
[26] M. V. Riofski, J. P. John, M. M. Zheng, J. Kirshner, D. A. Colby, *J. Org. Chem.* **2011**, *76*, 3676-3683.
[27] M. A. Chowdhury, H.-U. Reissig, *Synlett* **2006**, *2006*, 2383-2386.
[28] M. J. Niphakis, B. J. Turunen, G. I. Georg, *J. Org. Chem.* **2010**, *75*, 6793-6805.
[29] S. Liu, X. Zeng, B. Xu, *Tetrahedron Lett.* **2016**, *57*, 3706-3710.
[30] Y. Shi, B. Jung, S. Torker, A. H. Hoveyda, *J. Am. Chem. Soc.* **2015**, *137*, 8948-8964.
[31] A. Millán, P. D. Grigol Martinez, V. K. Aggarwal, *Chem. Eur. J.* **2018**, *24*, 730-735.
[32] C. García-Ruiz, J. L. Y. Chen, C. Sandford, K. Feeney, P. Lorenzo, G. Berionni, H. Mayr, V. K. Aggarwal, *J. Am. Chem. Soc.* **2017**, *139*, 15324-15327.
[33] R. Xuan, H.-S. Oh, Y. Lee, H.-Y. Kang, *J. Org. Chem.* **2008**, *73*, 1456-1461.
[34] R. A. Pilli, C. K. Z. de Andrade, C. R. O. Souto, A. de Meijere, *J. Org. Chem.* **1998**, *63*, 7811-7819.

4. SPECTROSCOPIC DATA

^{1}H NMR (400 MHz, $CDCl_3$) of **4a**

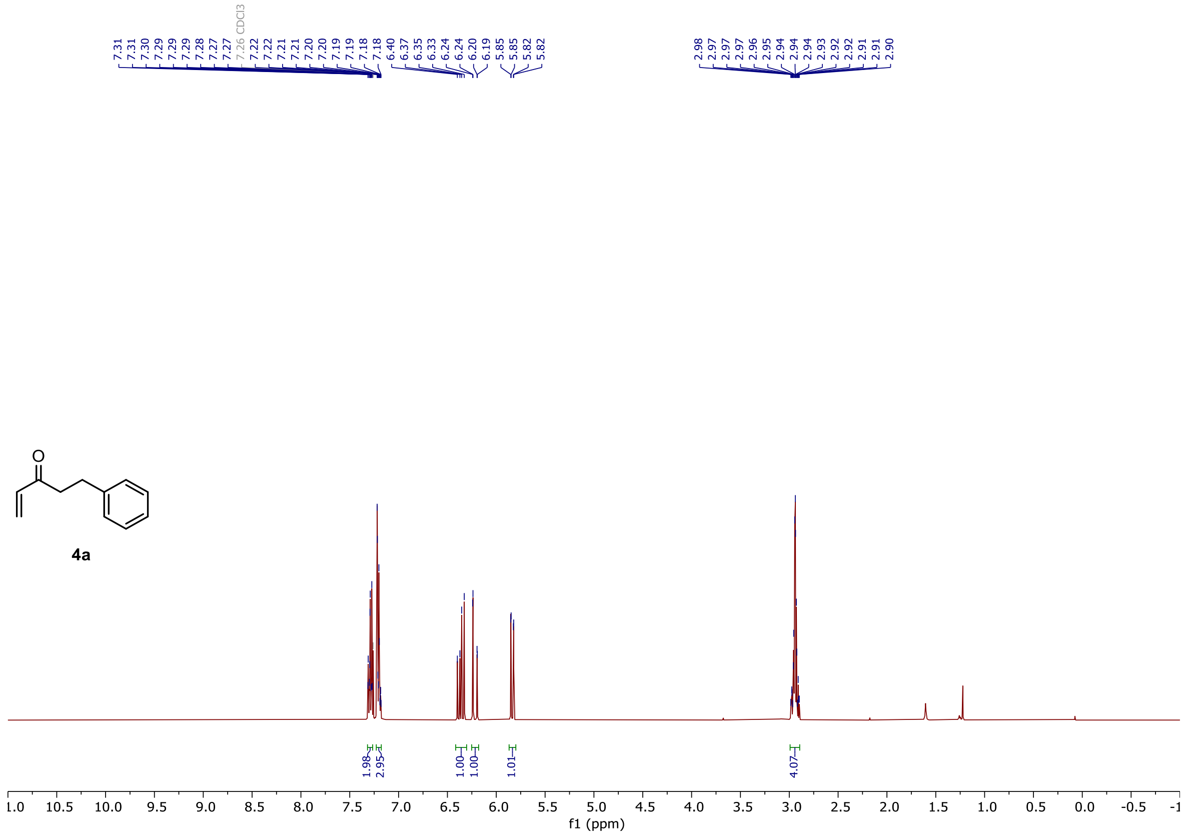

^{13}C NMR (101 MHz, $CDCl_3$) of **4a**

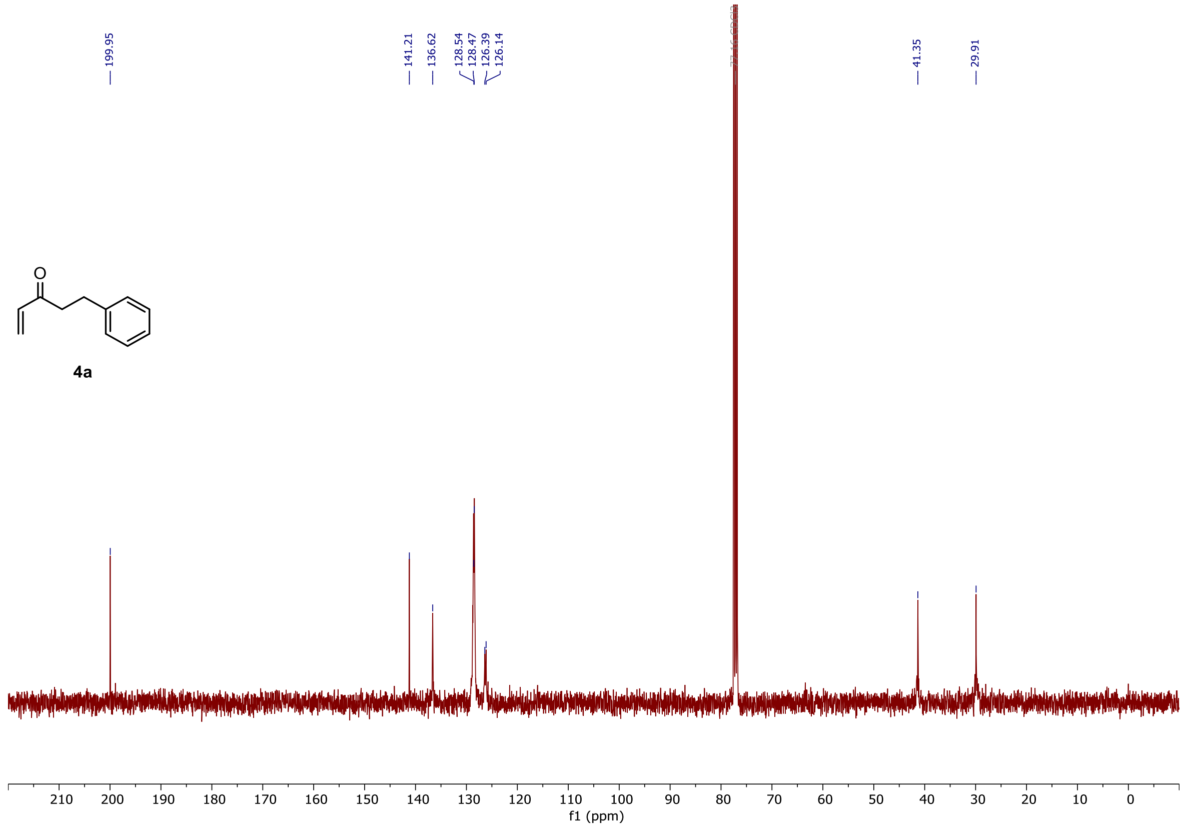

^{1}H NMR (400 MHz, $CDCl_3$) of **4b**

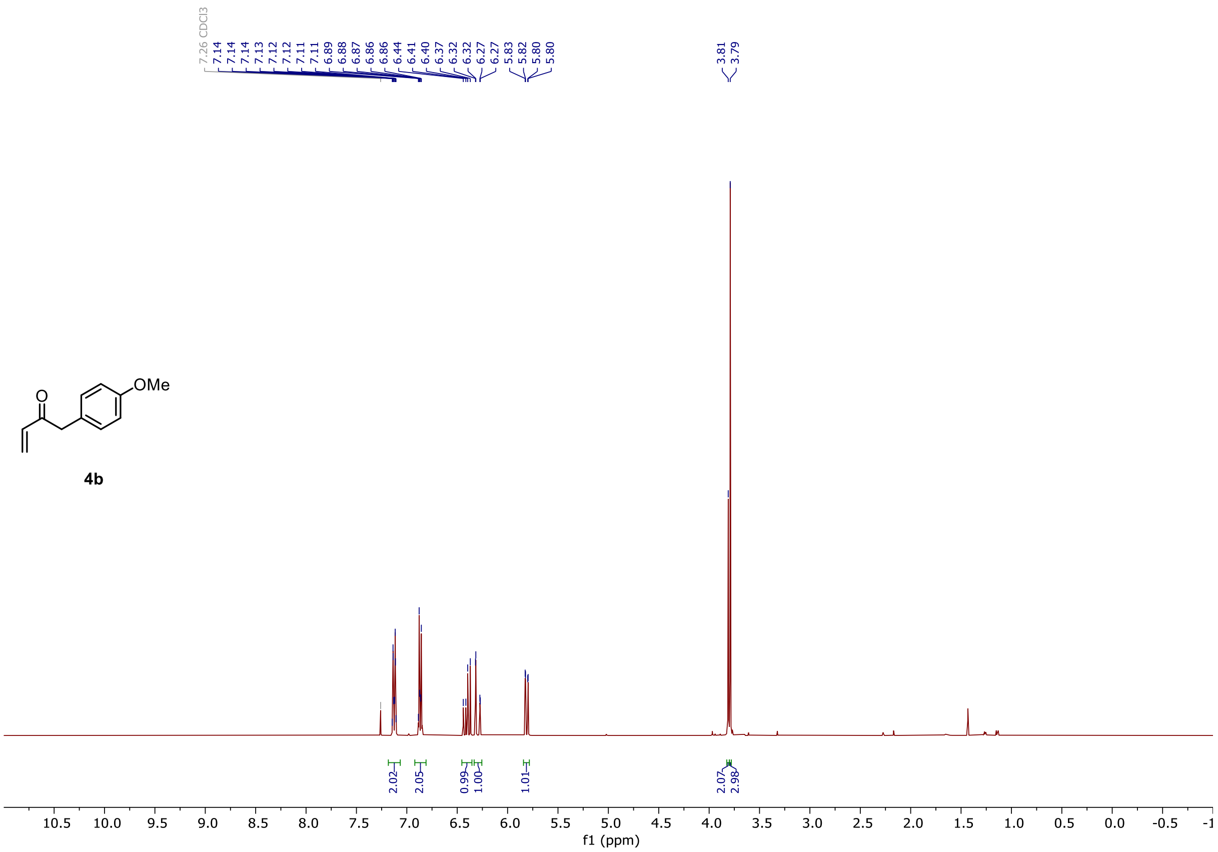

^{13}C NMR (101 MHz, $CDCl_3$) of **4b**

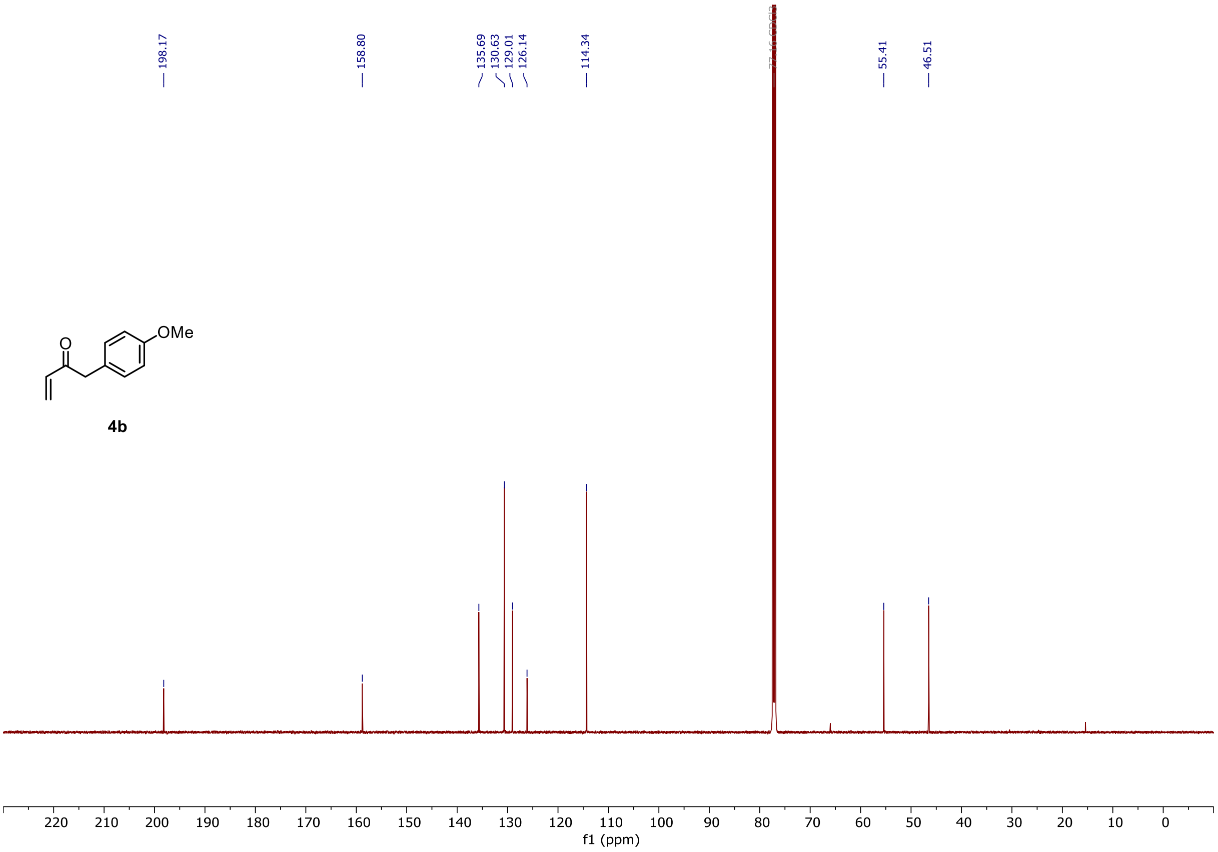

^{1}H NMR (400 MHz, $CDCl_3$) of **4c**

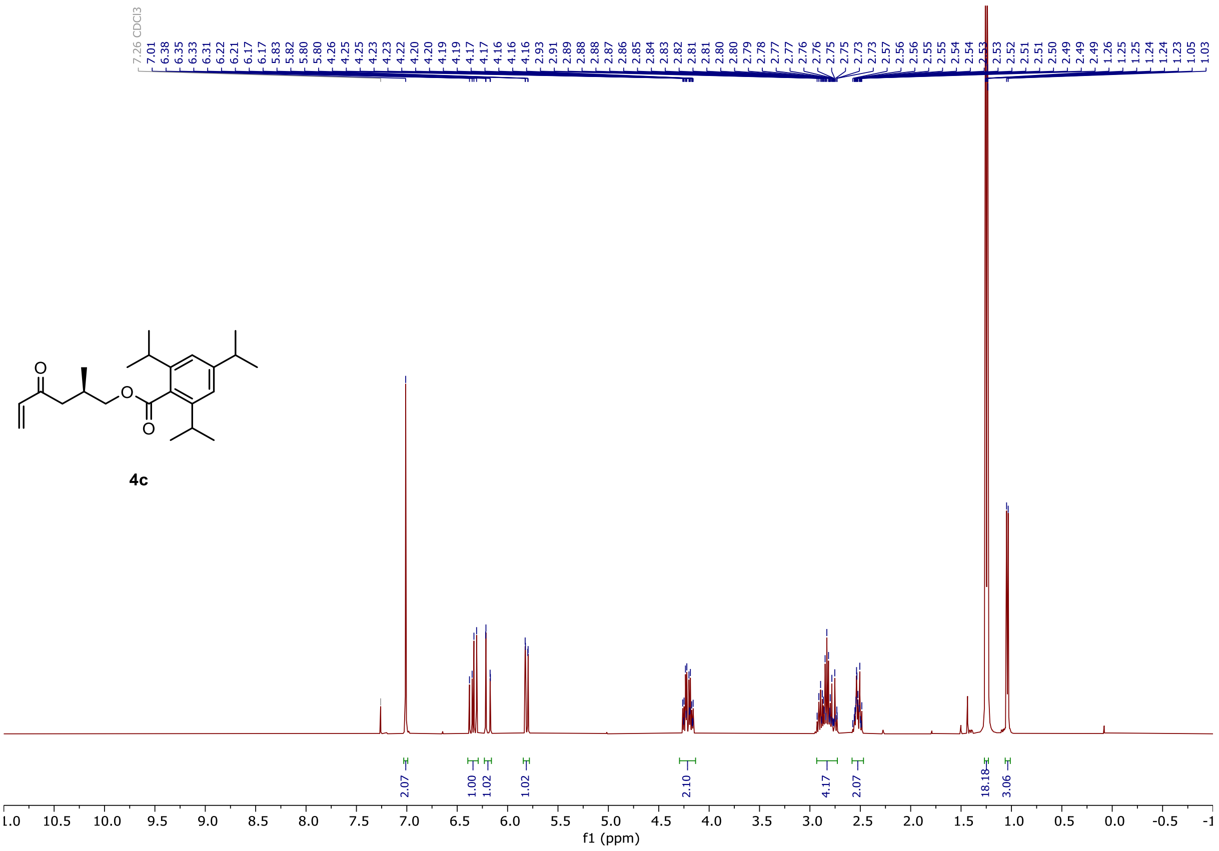

^{13}C NMR (101 MHz, $CDCl_3$) of **4c**

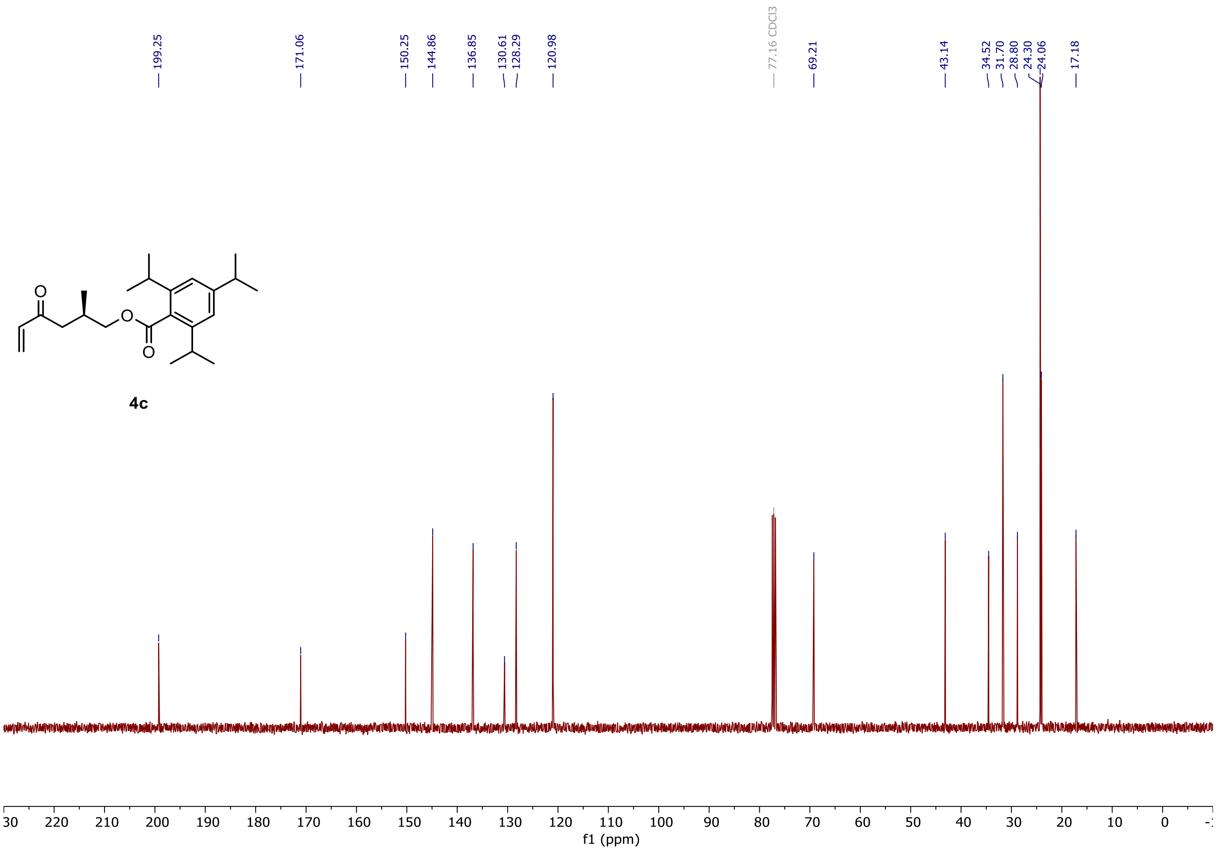

^{1}H NMR (400 MHz, $CDCl_3$) of **4d**

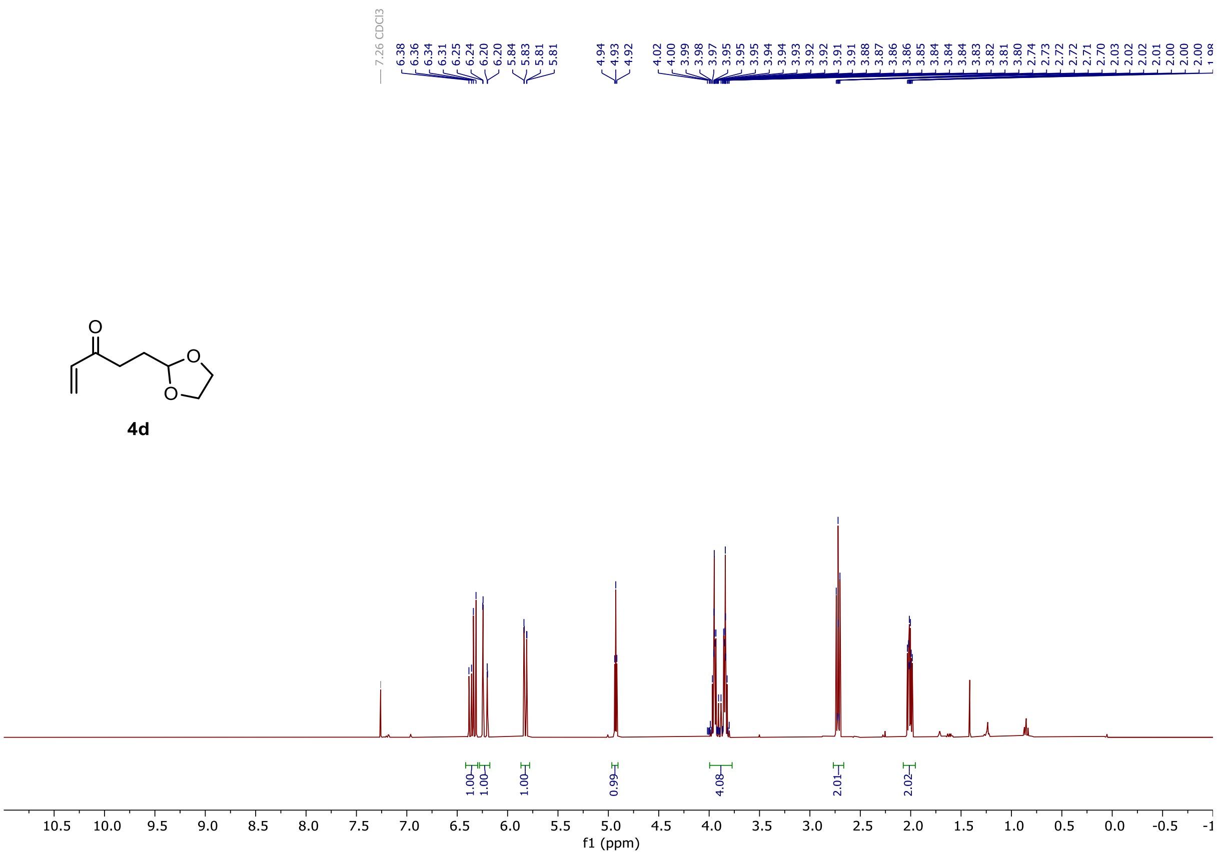

^{13}C NMR (101 MHz, $CDCl_3$) of **4d**

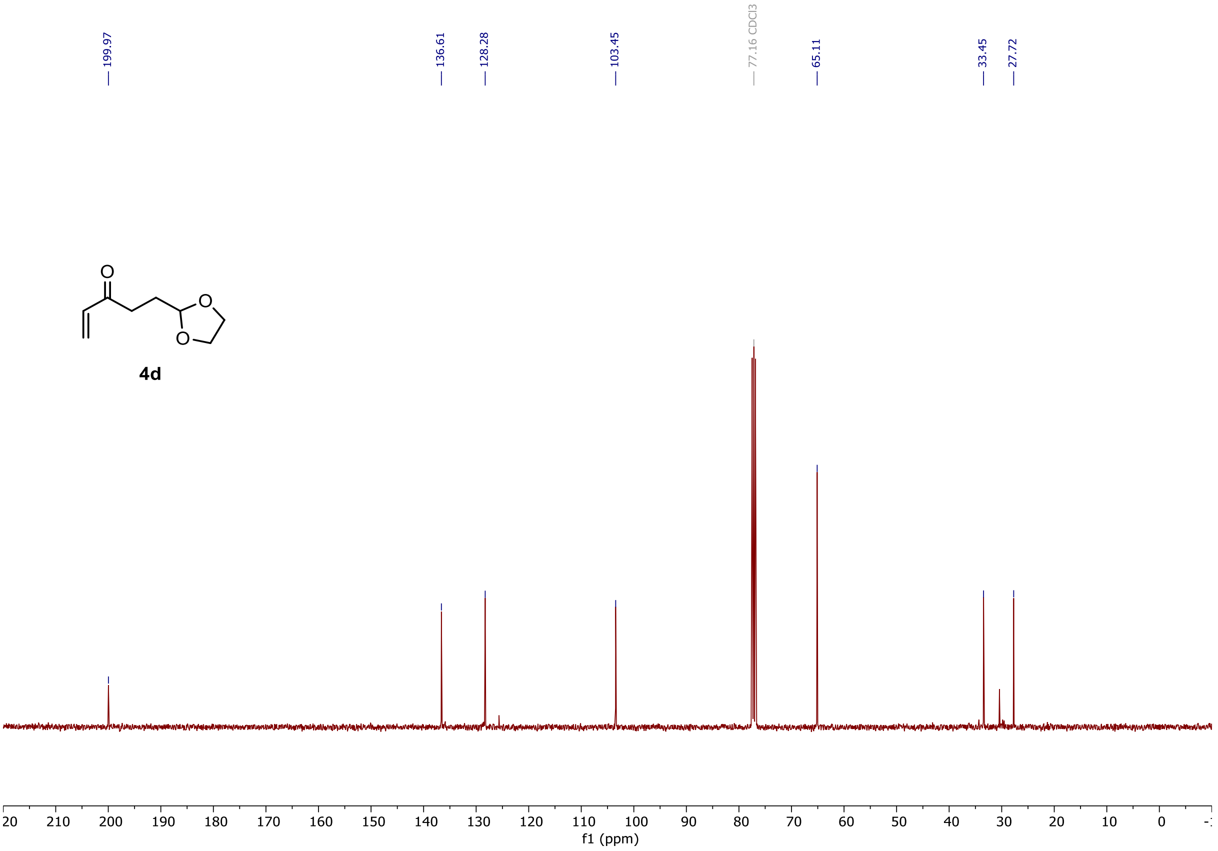

^{1}H NMR (400 MHz, $CDCl_3$) of **4e**

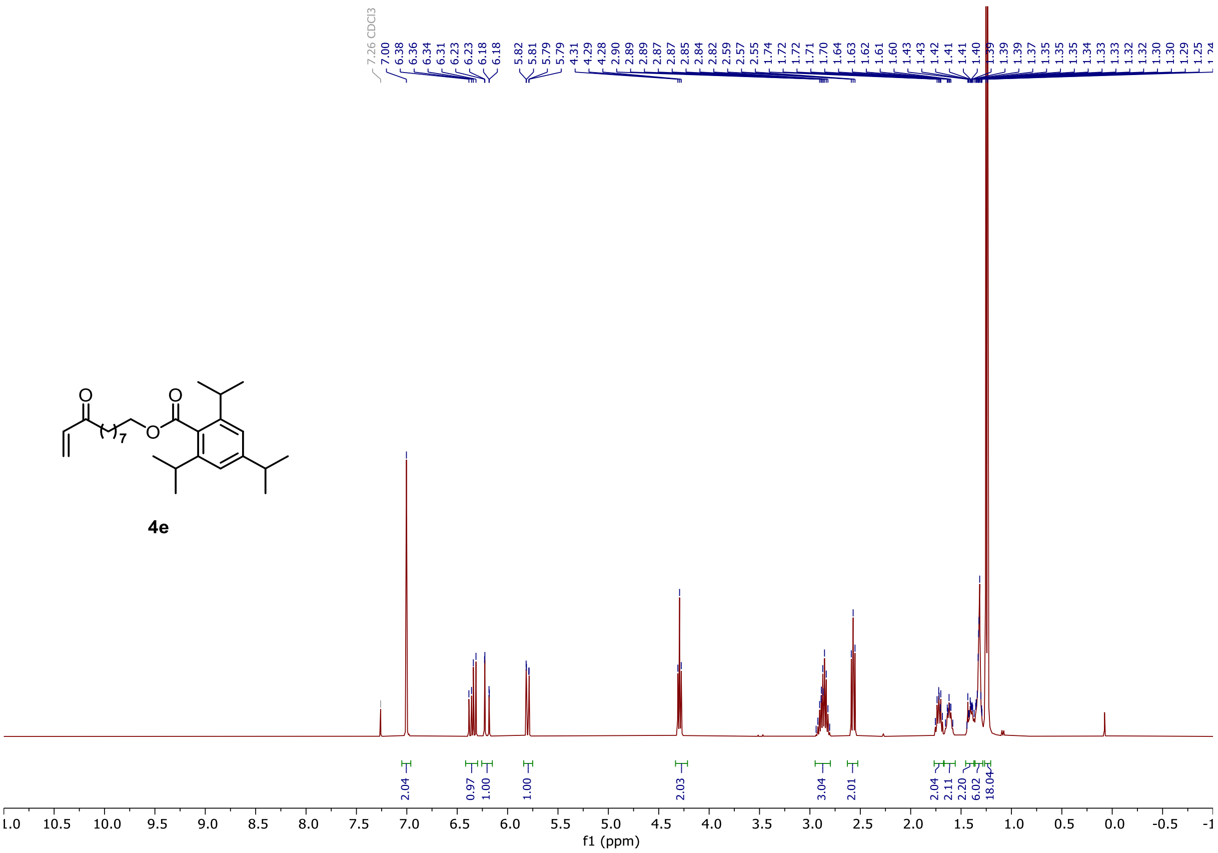

^{13}C NMR (101 MHz, $CDCl_3$) of **4e**

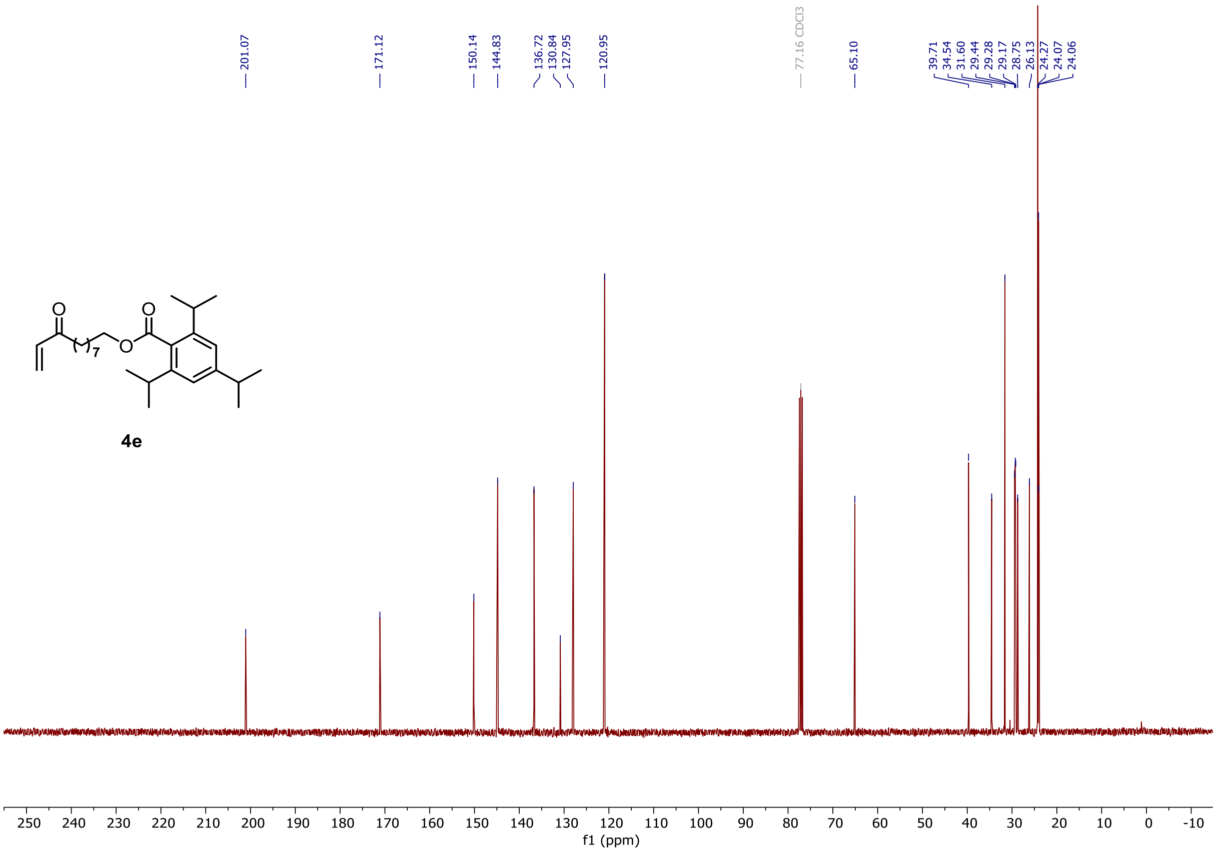

^{1}H NMR (400 MHz, $CDCl_3$) of **4f**

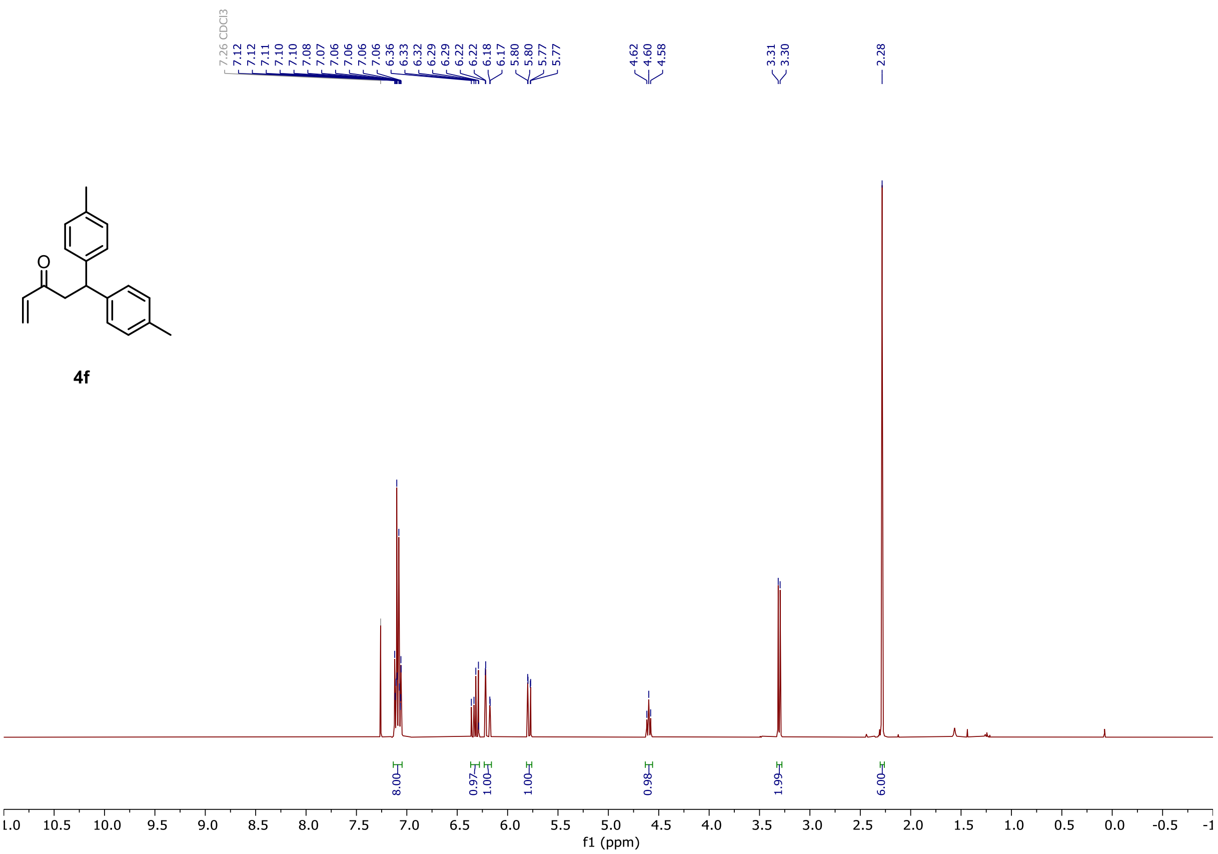

^{13}C NMR (101 MHz, $CDCl_3$) of **4f**

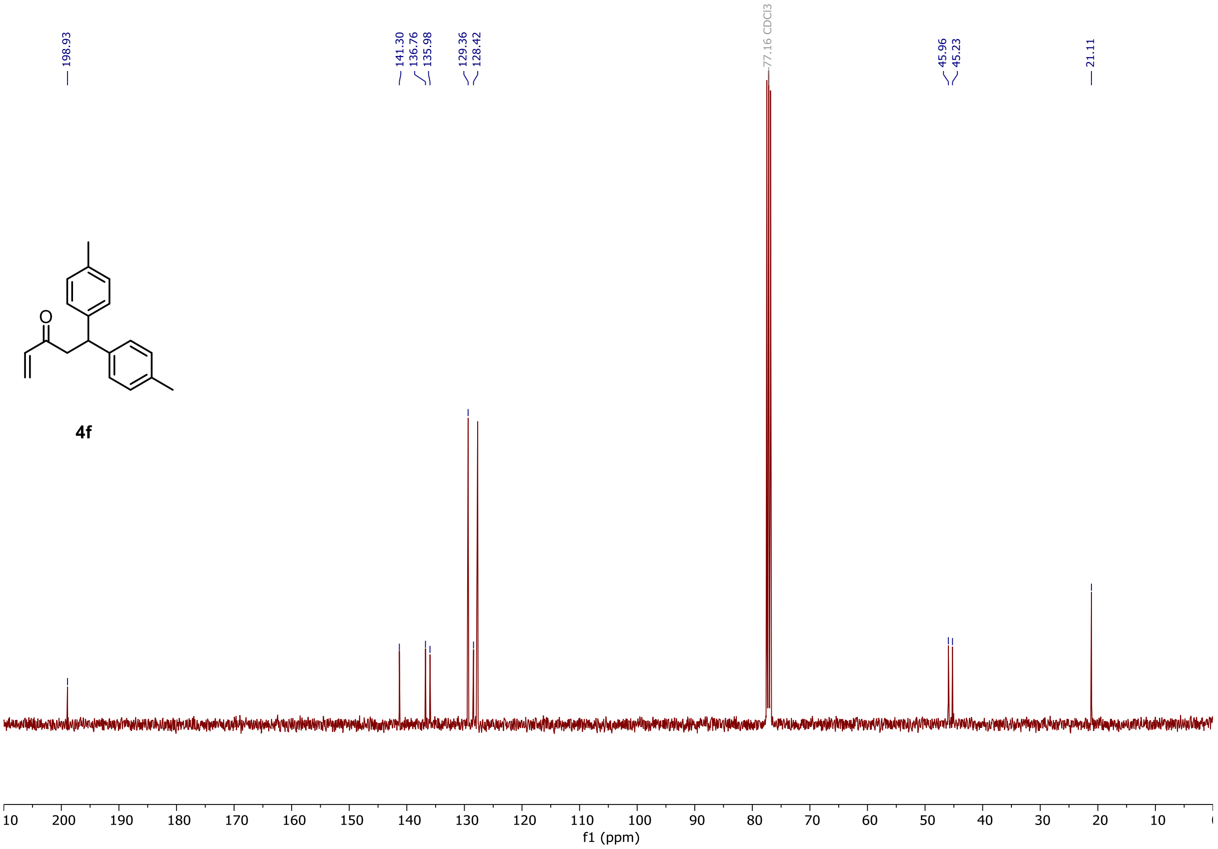

^{1}H NMR (400 MHz, $CDCl_3$) of **4g**

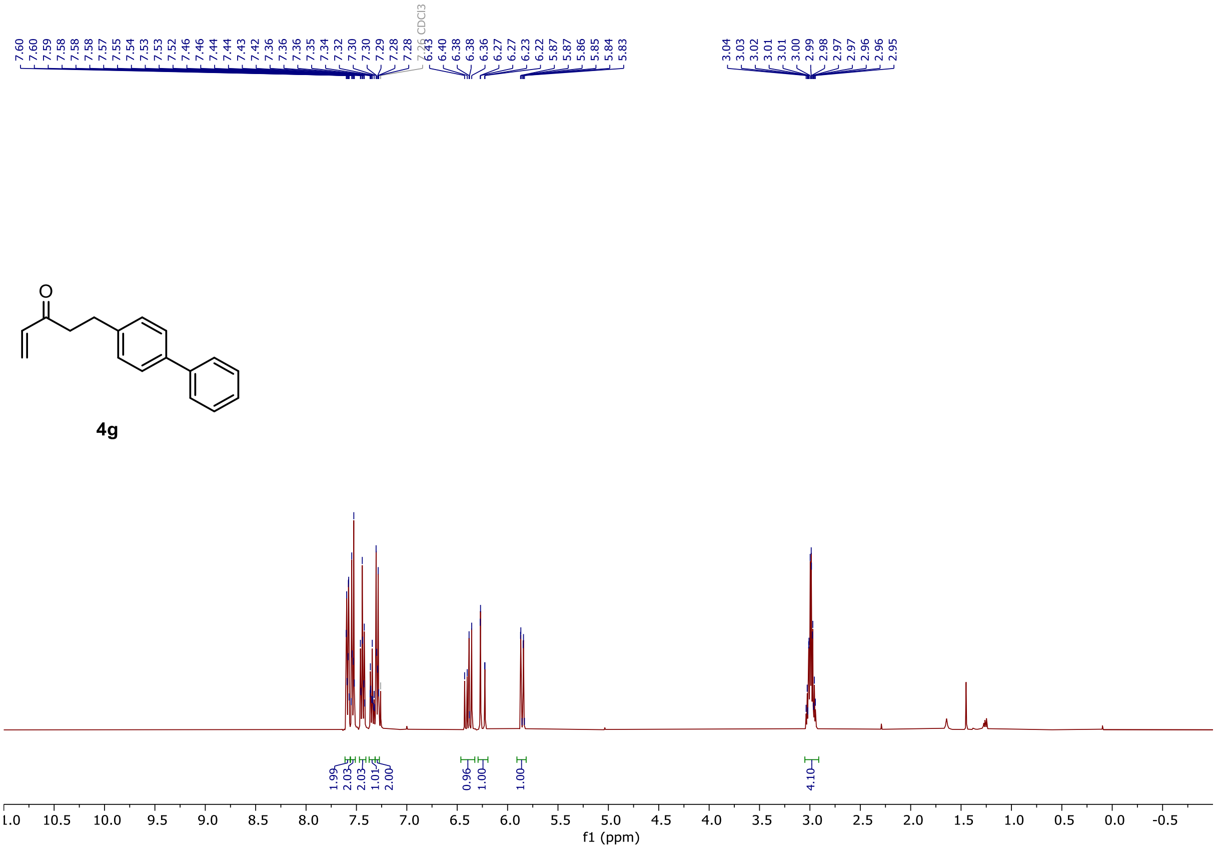

^{13}C NMR (101 MHz, $CDCl_3$) of **4g**

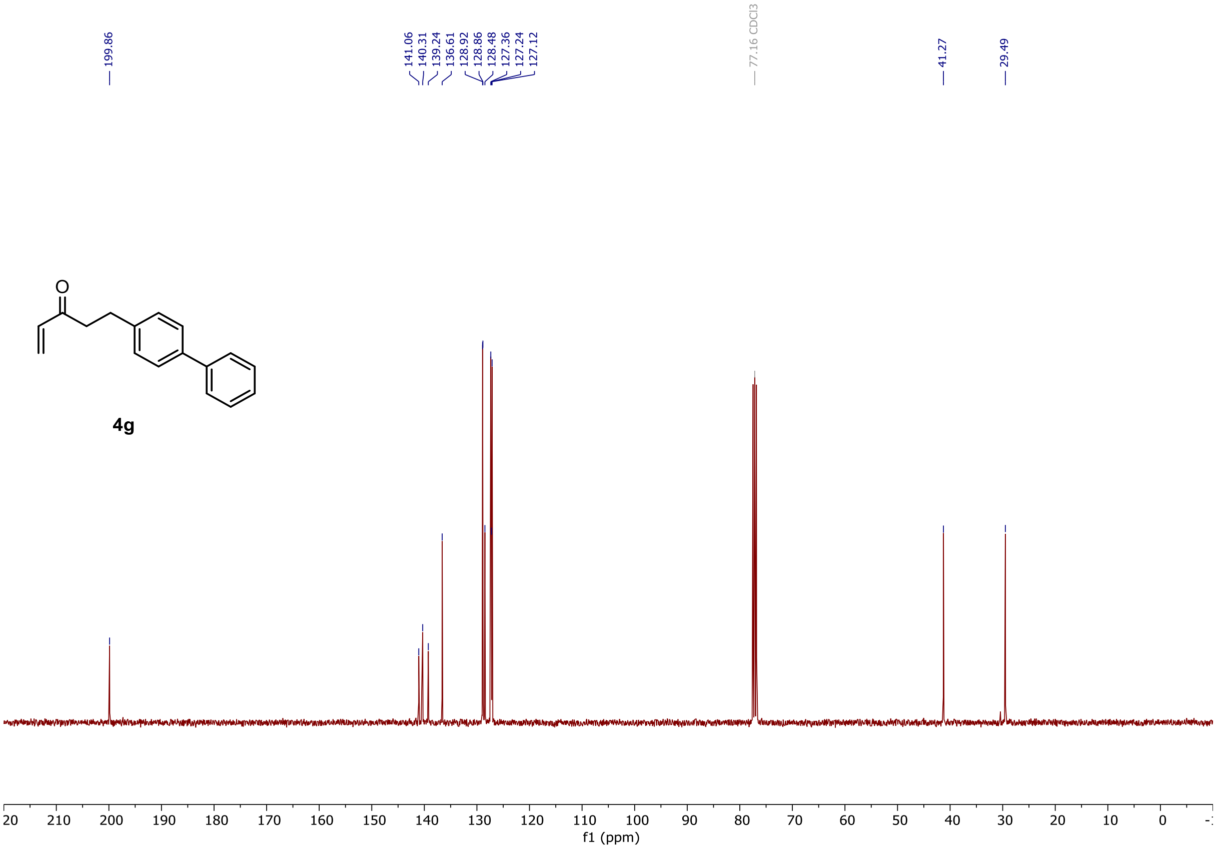

^{1}H NMR (400 MHz, $CDCl_3$) of **4h**

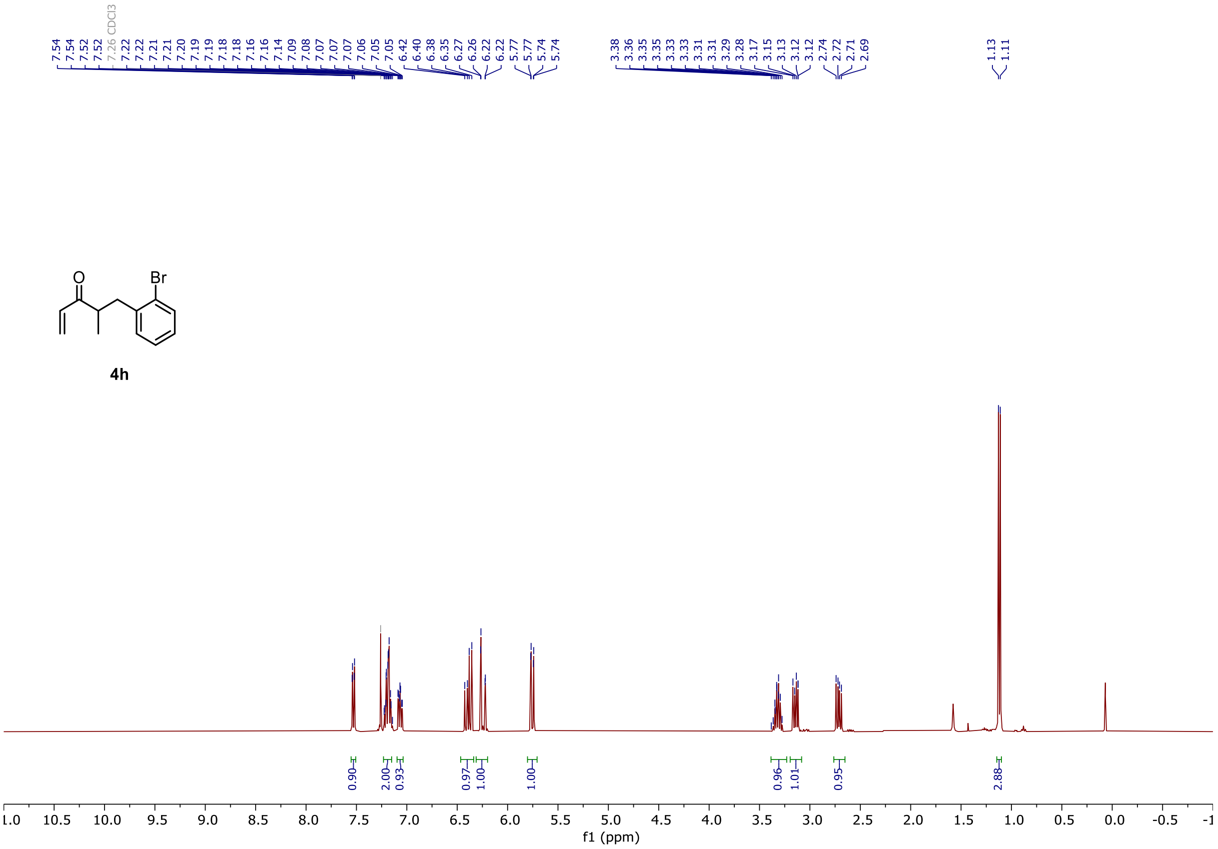

^{13}C NMR (101 MHz, $CDCl_3$) of **4h**

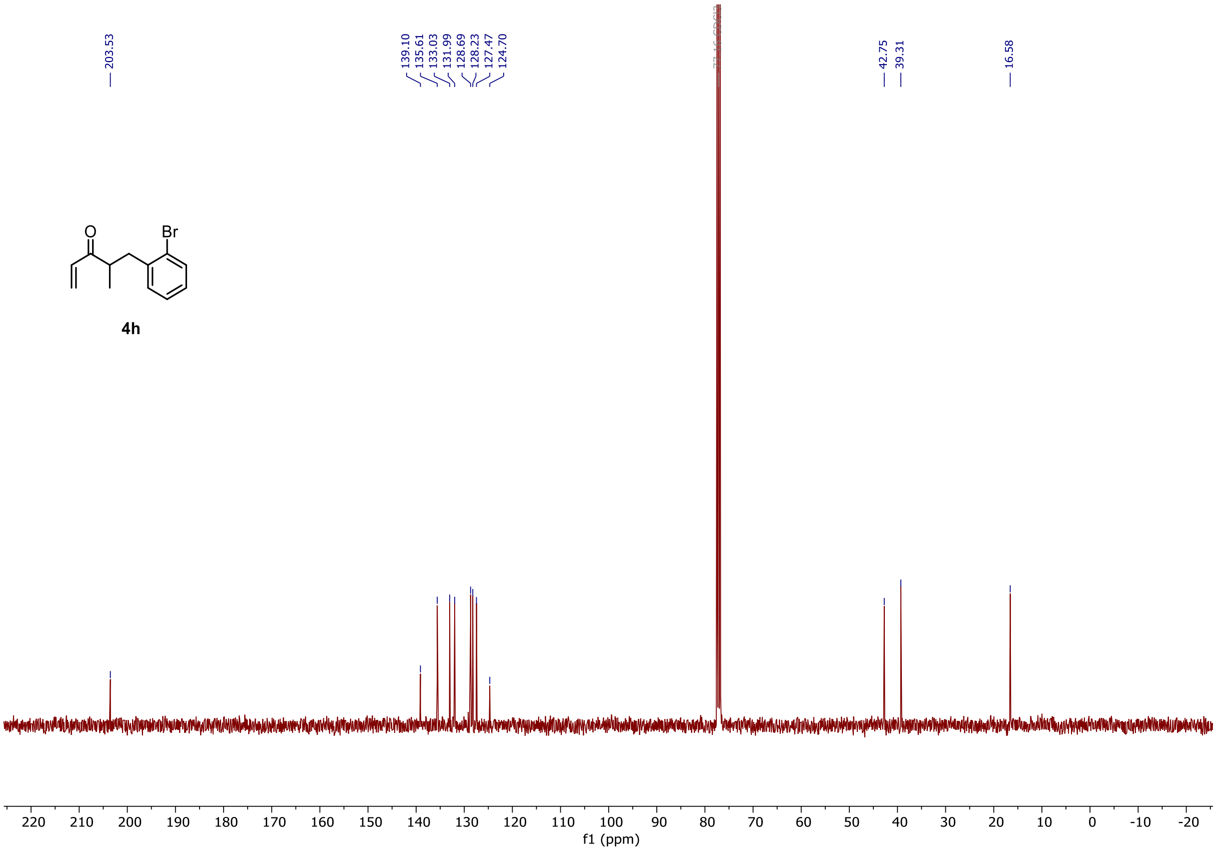

^{1}H NMR (400 MHz, $CDCl_3$) of **4i**

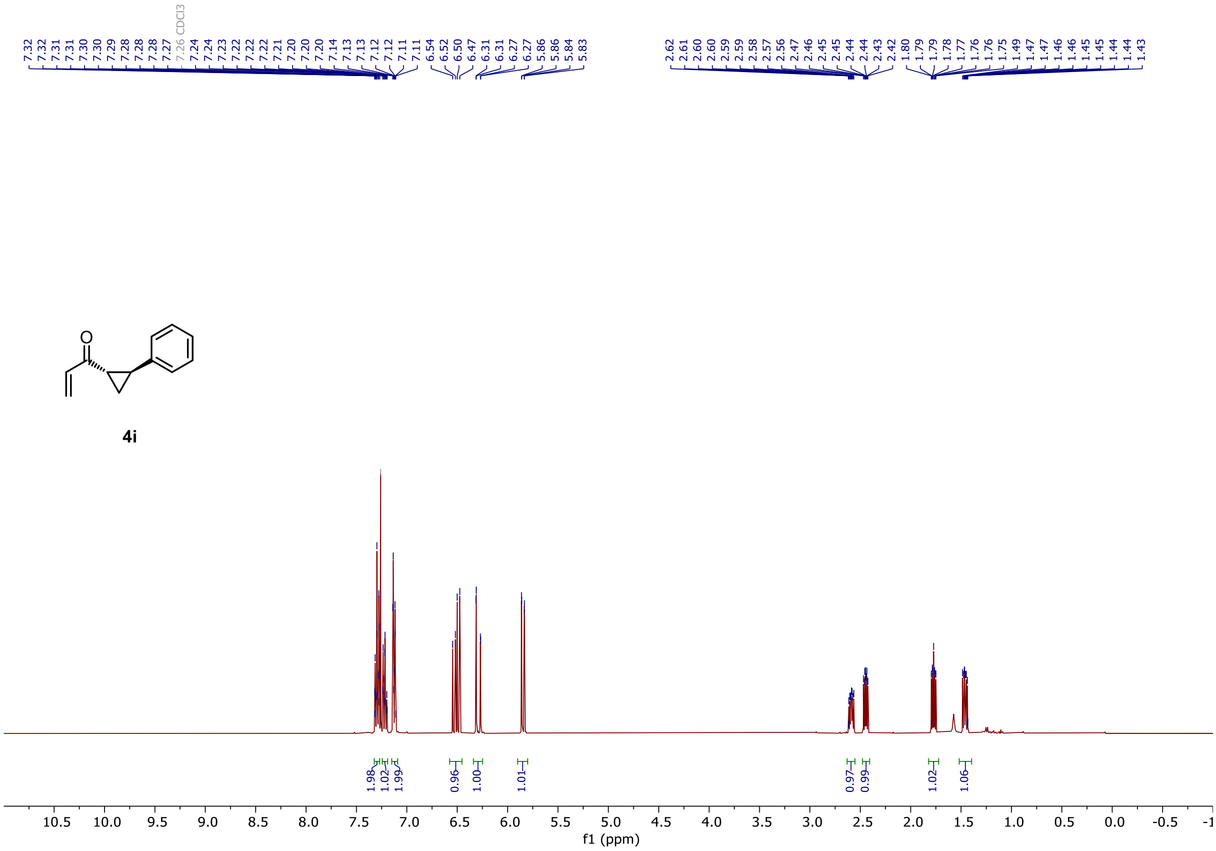

^{13}C NMR (101 MHz, $CDCl_3$) of **4i**

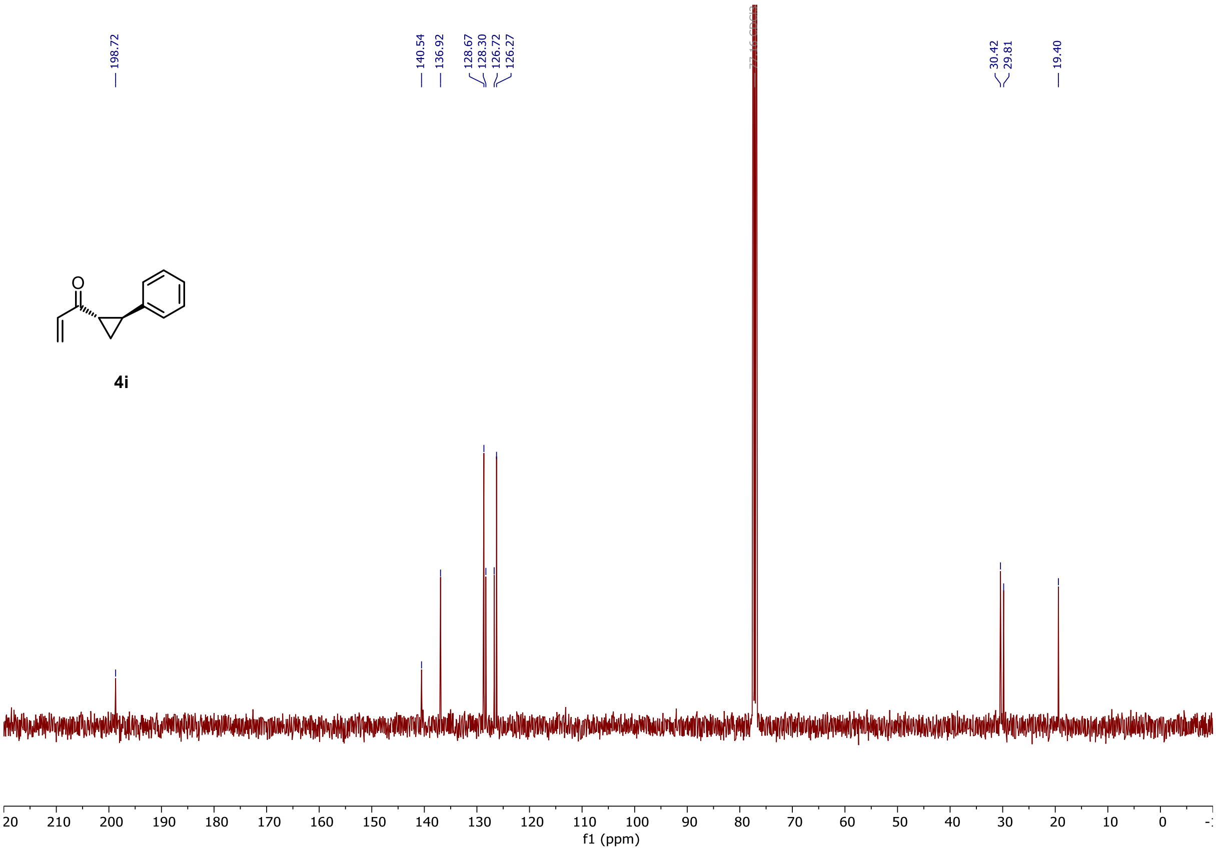

^{1}H NMR (400 MHz, CDCl$_3$) of **4j**

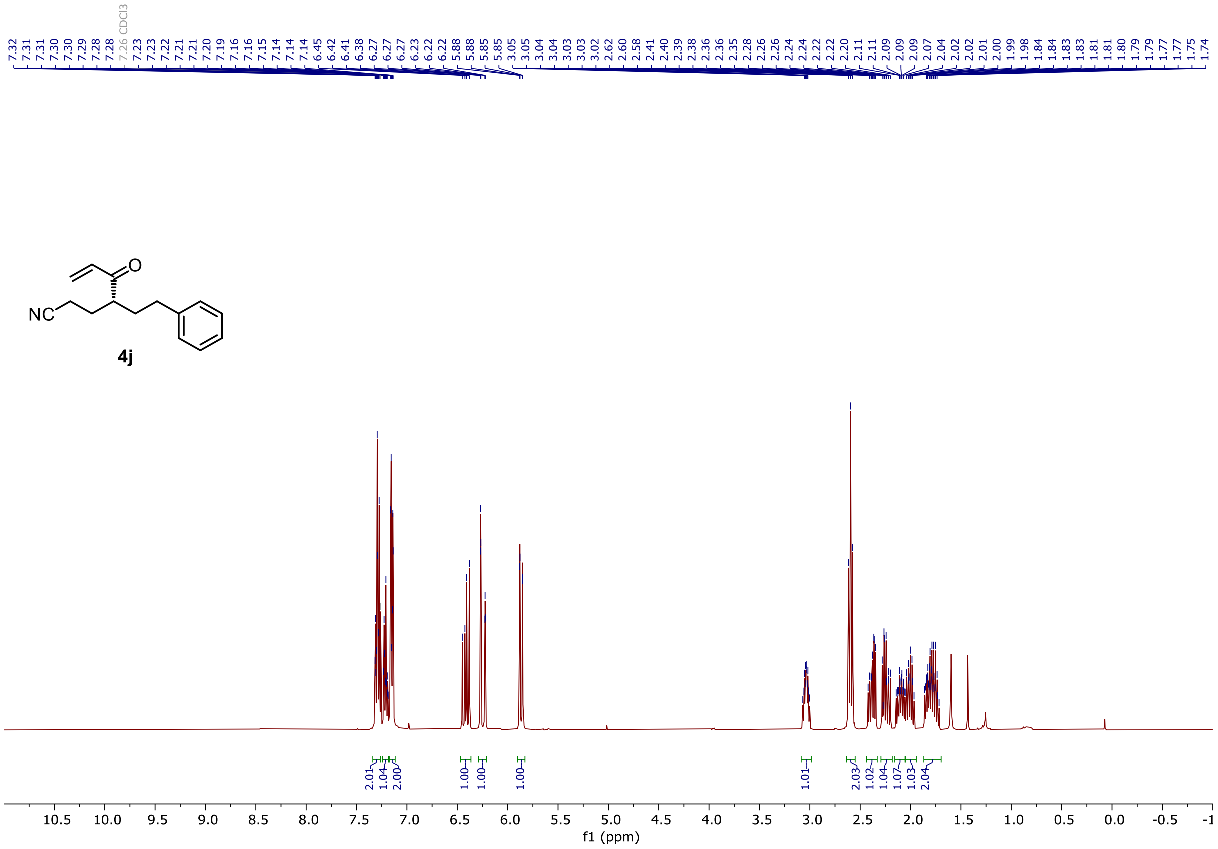

^{13}C NMR (101 MHz, CDCl$_3$) of **4j**

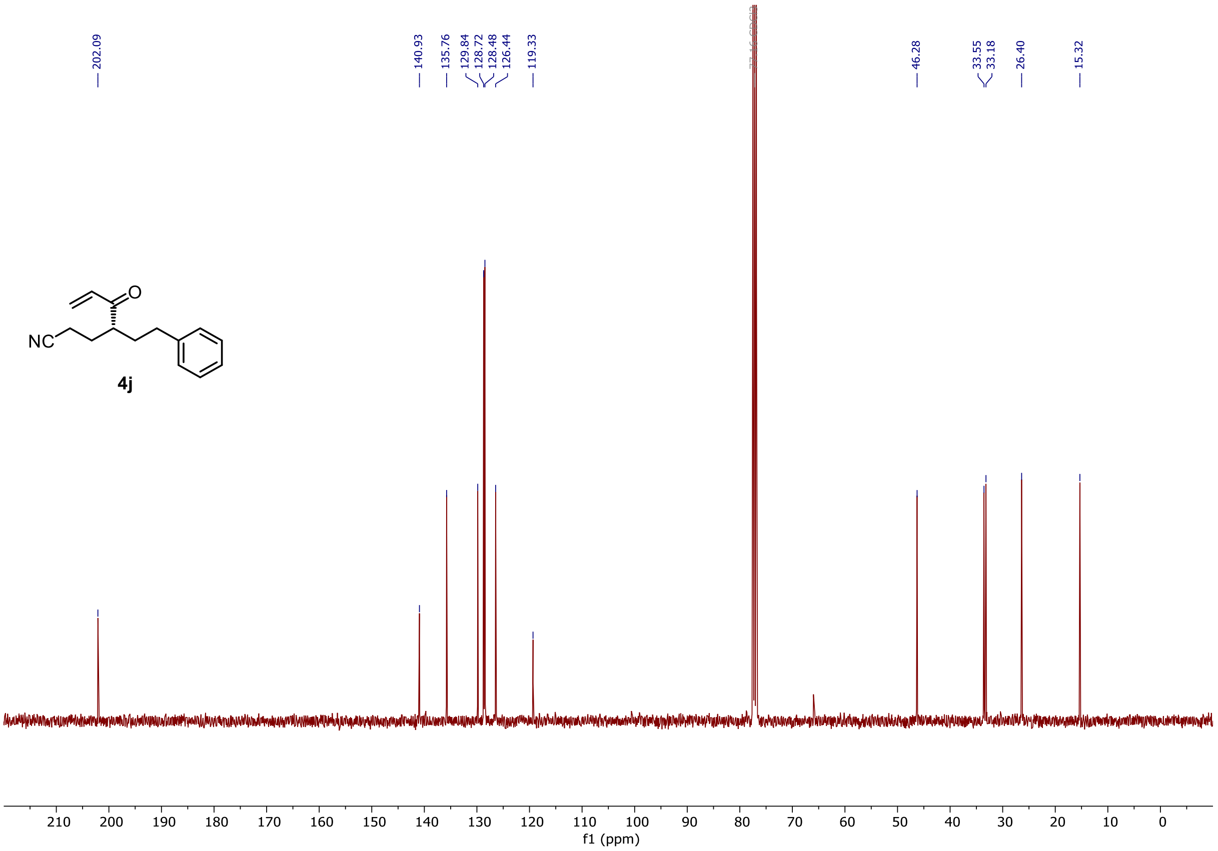

^{1}H NMR (400 MHz, $CDCl_3$) of **4k**

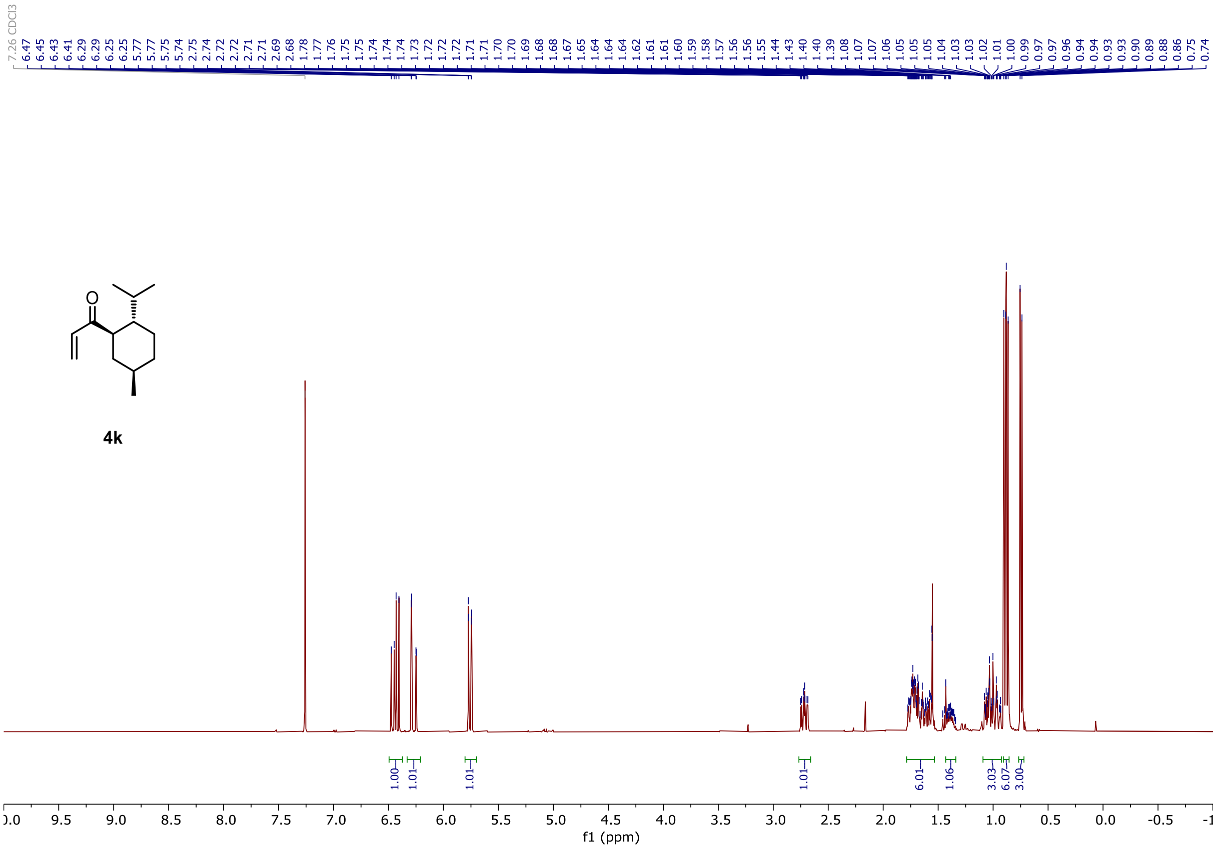

^{13}C NMR (101 MHz, $CDCl_3$) of **4k**

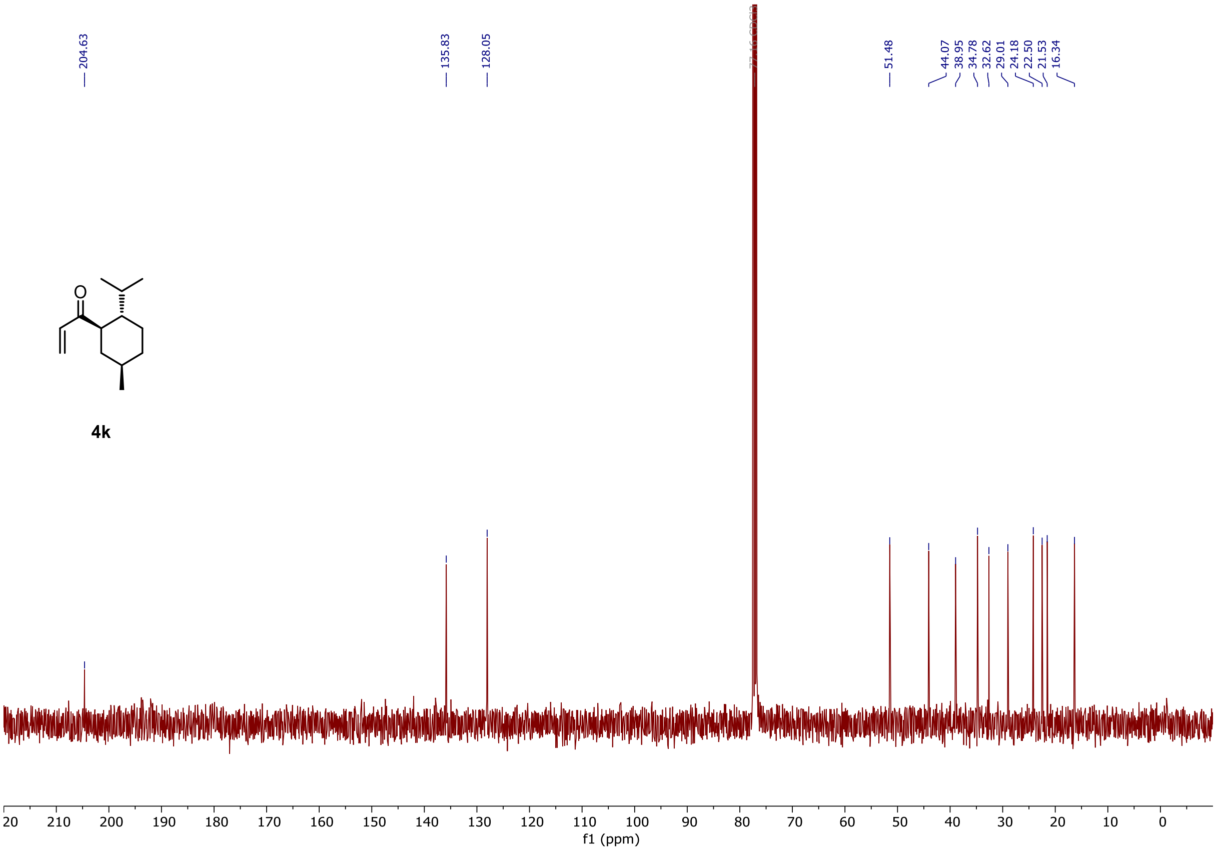

^{1}H NMR (400 MHz, CDCl$_3$) of **4l**

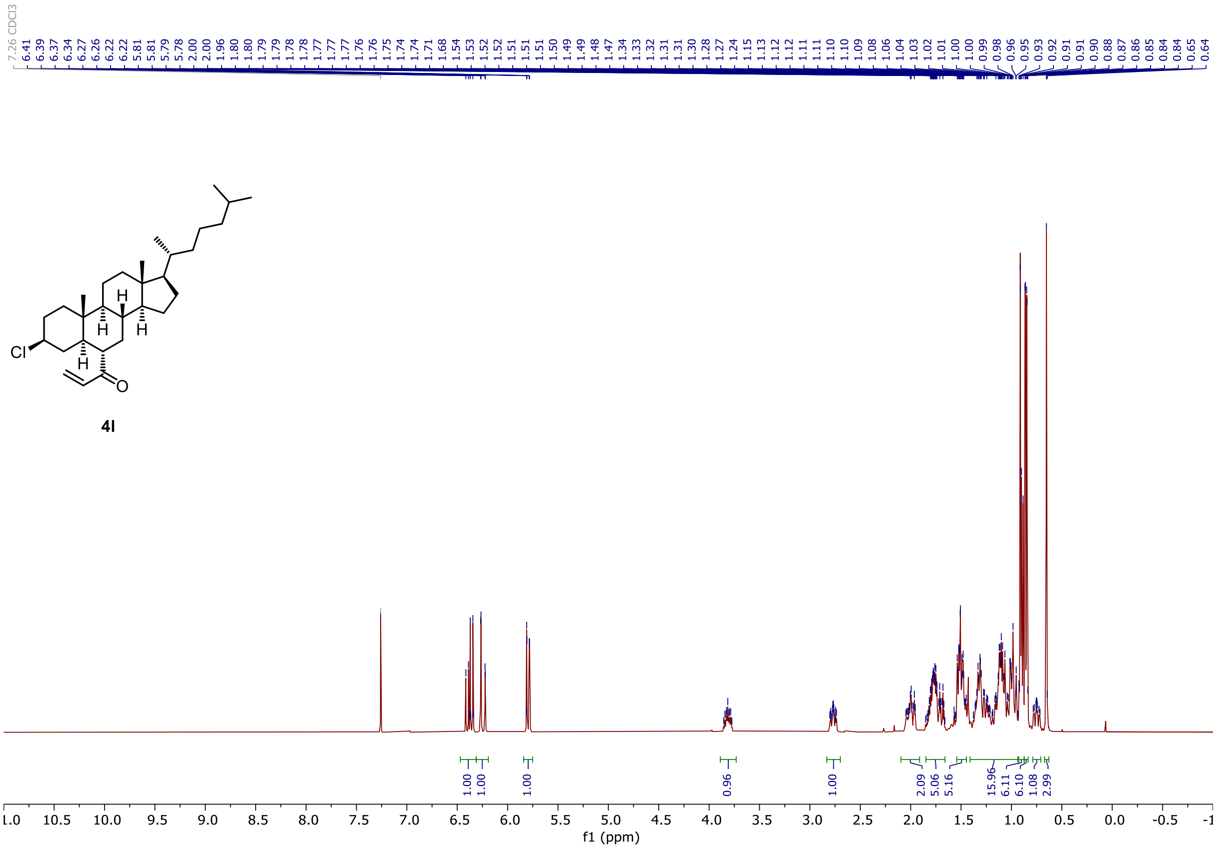

4l

^{13}C NMR (101 MHz, CDCl$_3$) of **4l**

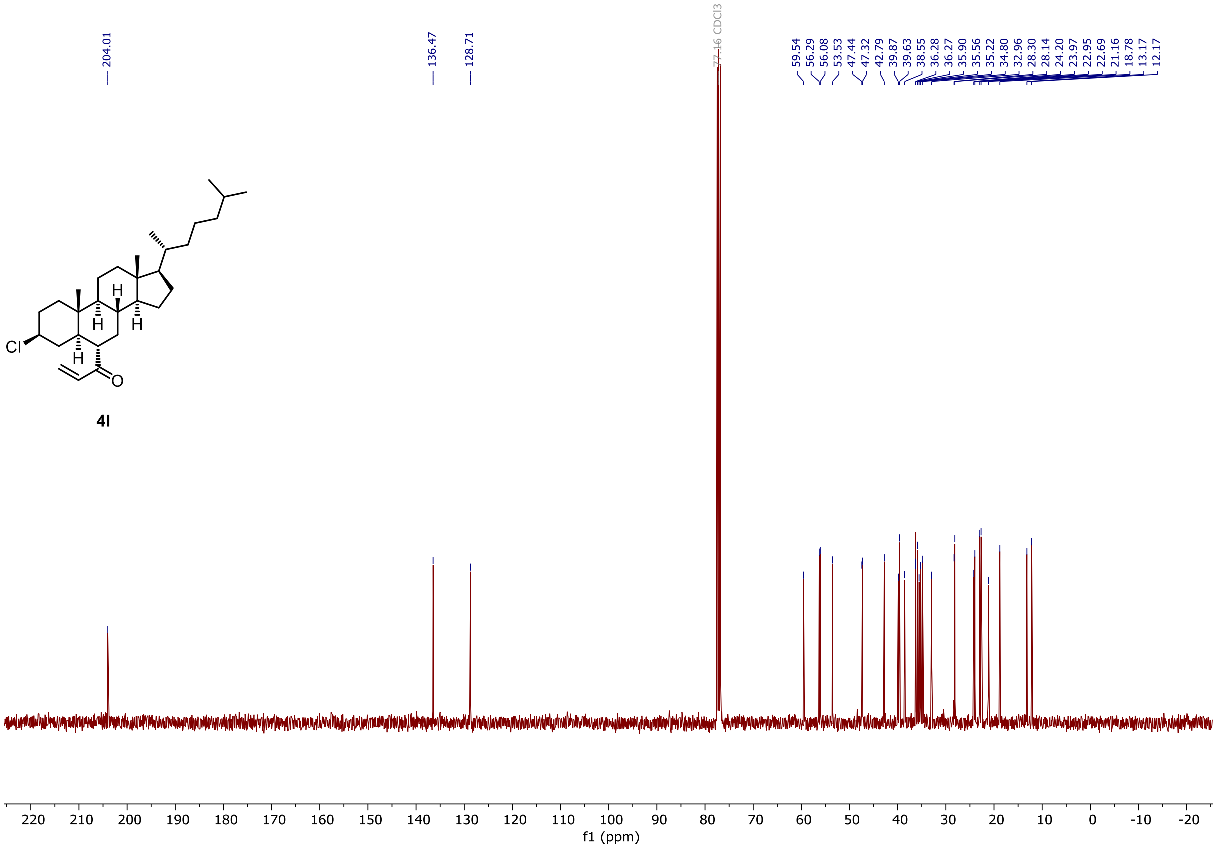

4l

^{1}H NMR (400 MHz, $CDCl_3$) of **4m**

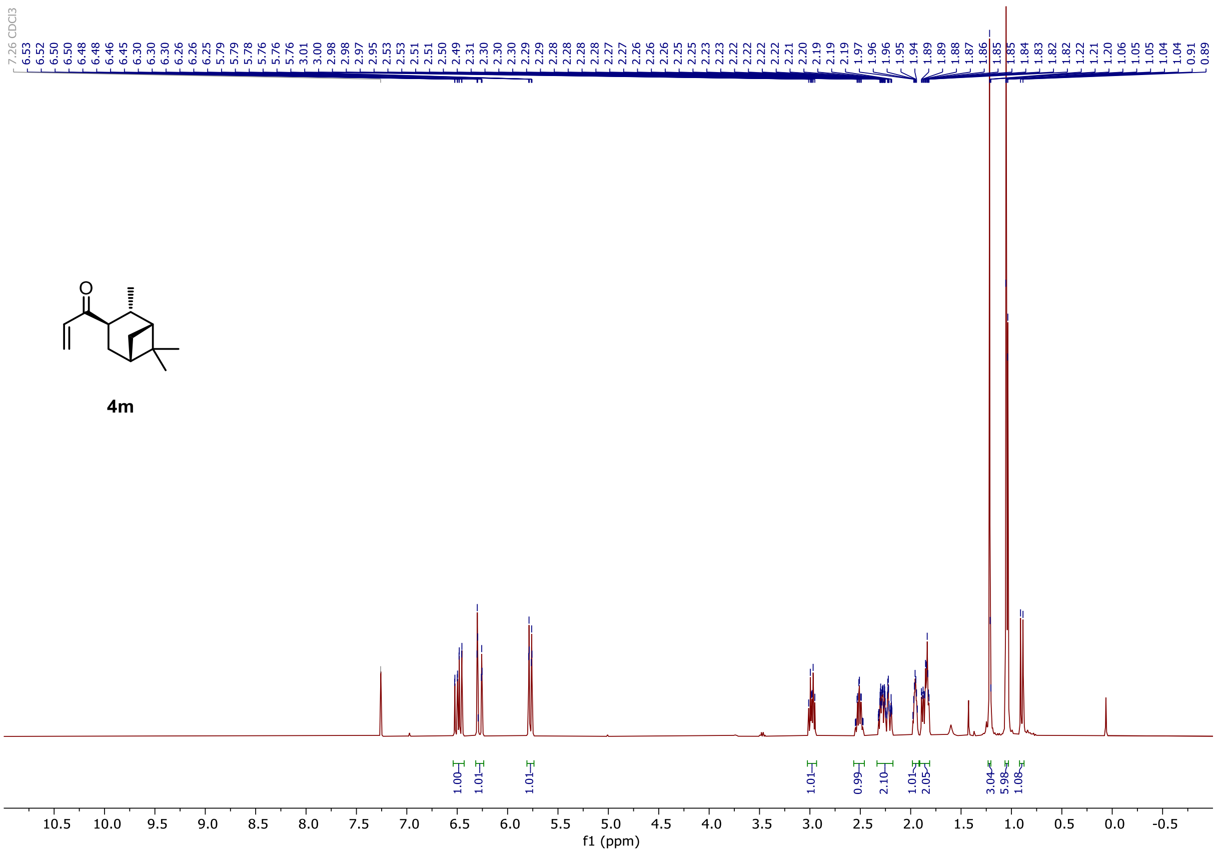

^{13}C NMR (101 MHz, $CDCl_3$) of **4m**

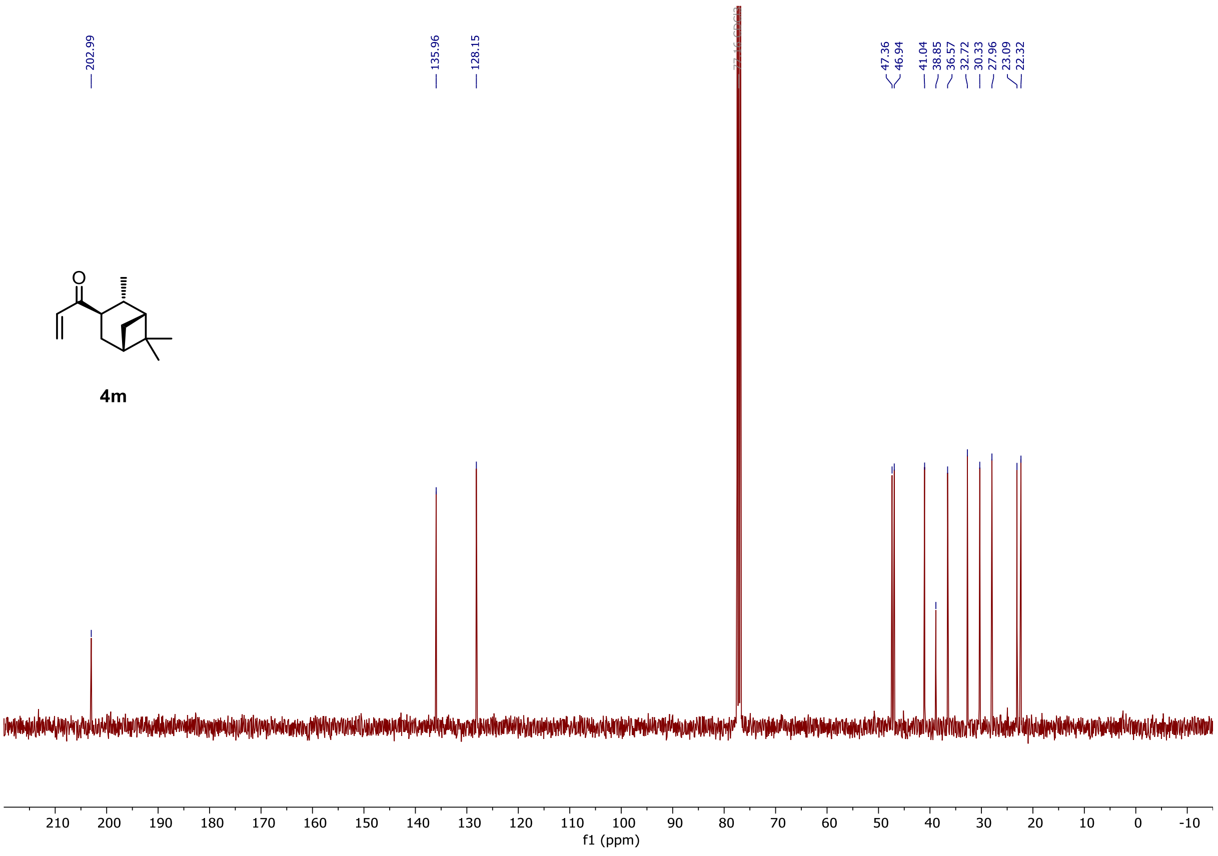

^{1}H NMR (400 MHz, $CDCl_3$) of **4n**

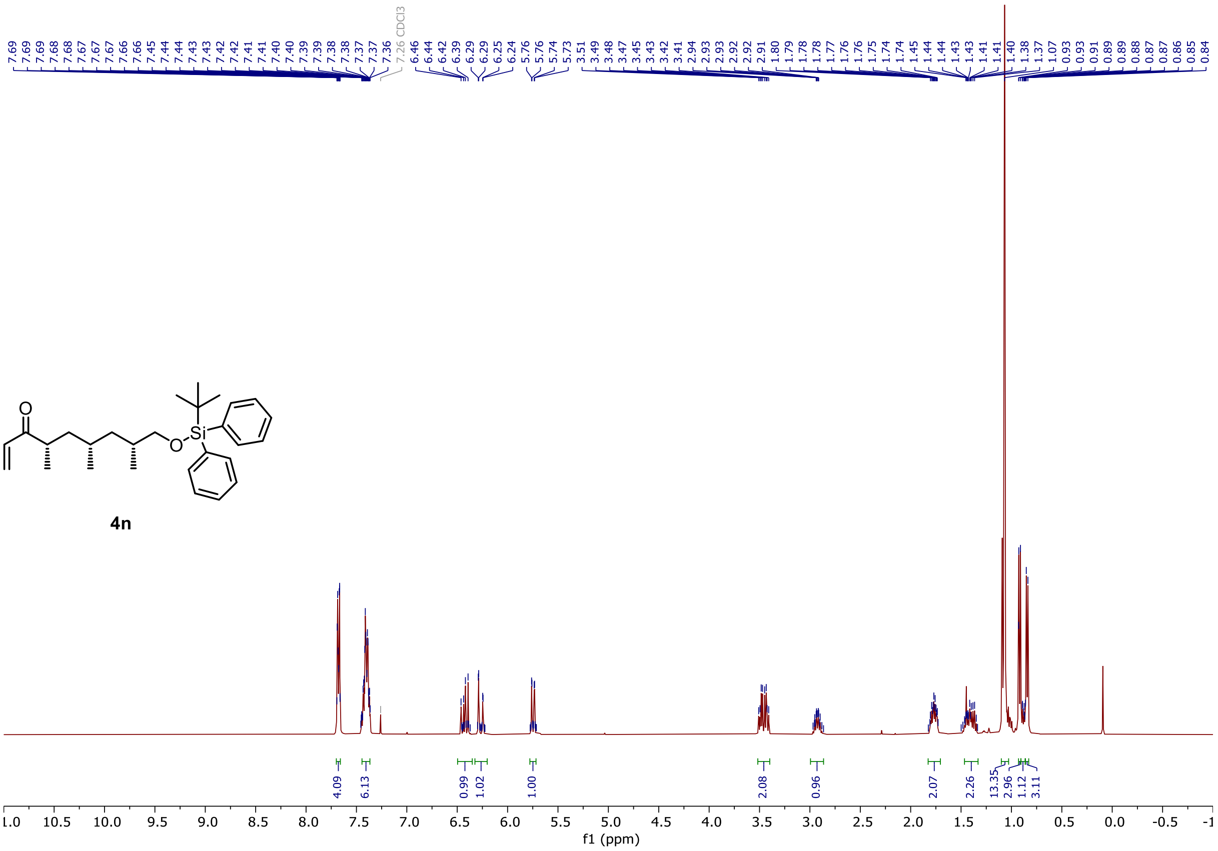

^{13}C NMR (101 MHz, $CDCl_3$) of **4n**

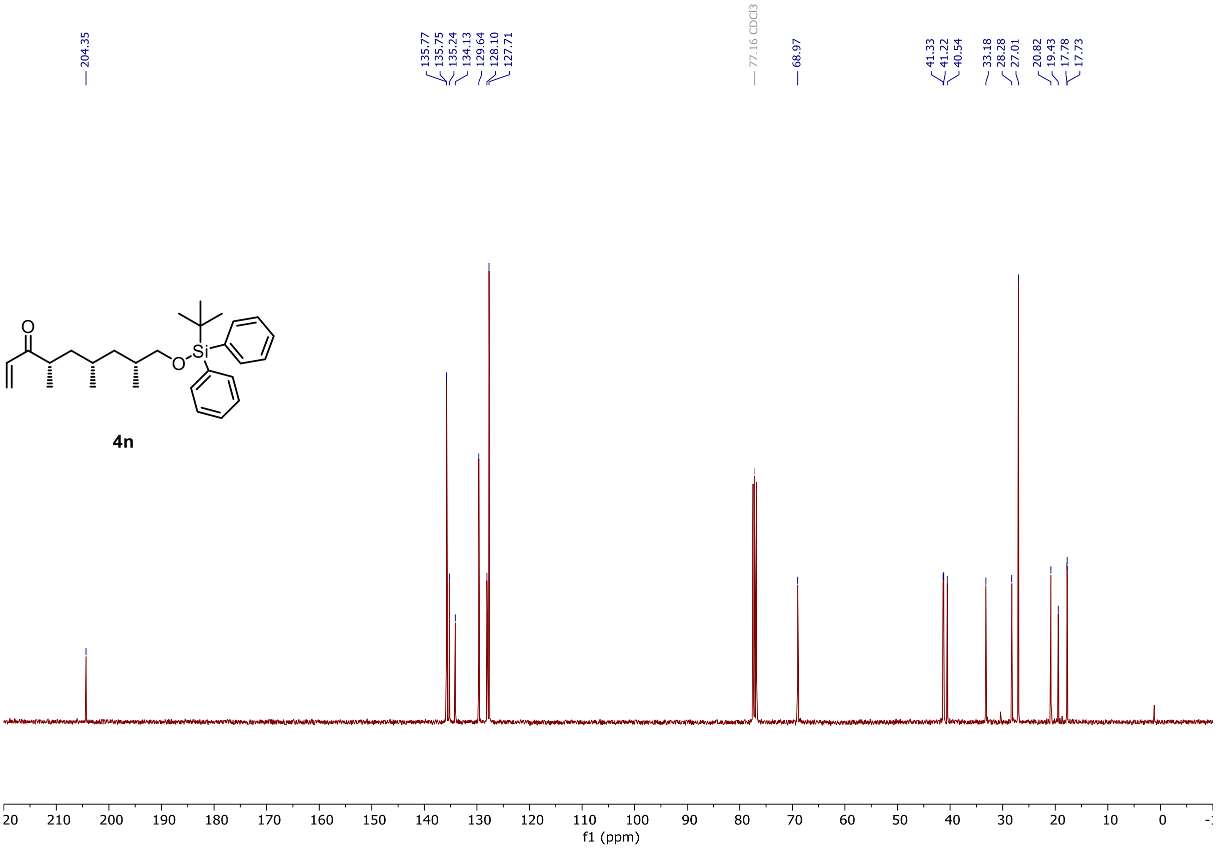

^{1}H NMR (400 MHz, $CDCl_3$) of **4o**

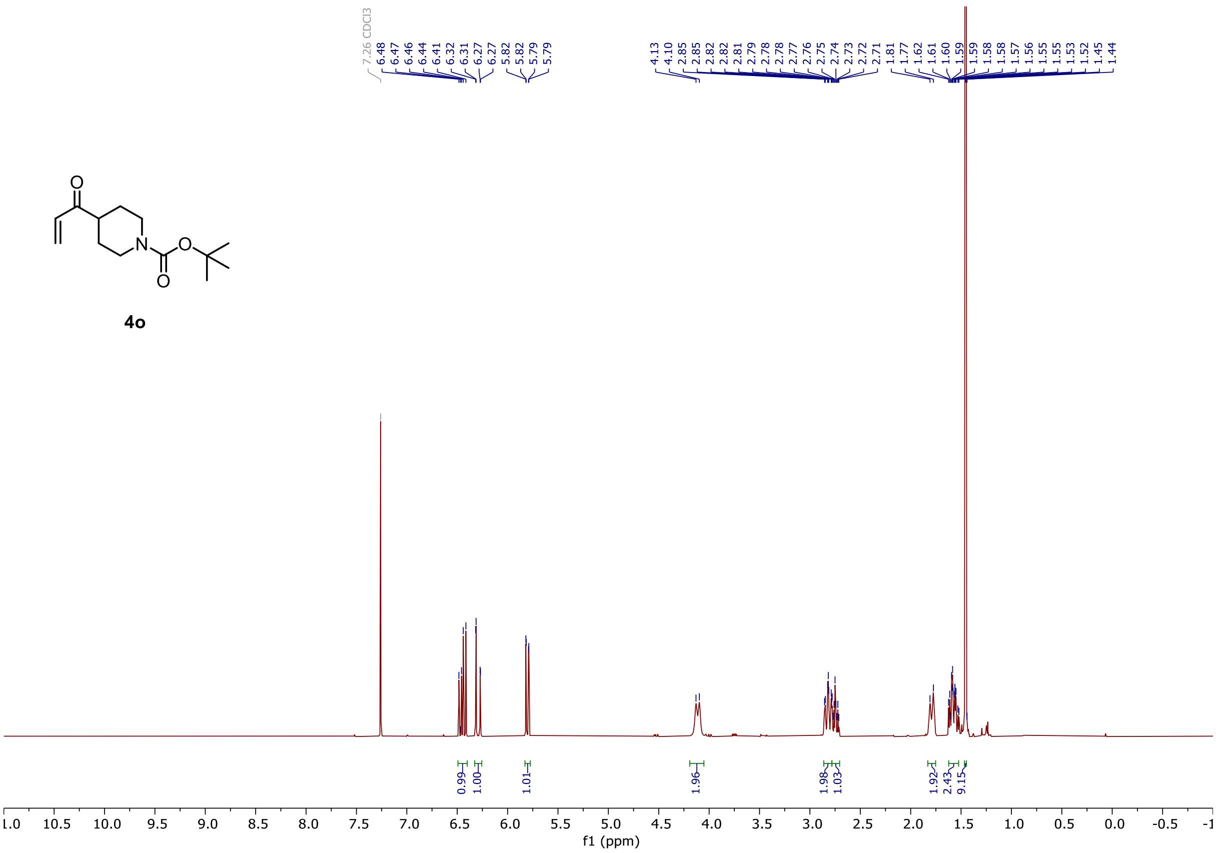

^{13}C NMR (101 MHz, $CDCl_3$) of **4o**

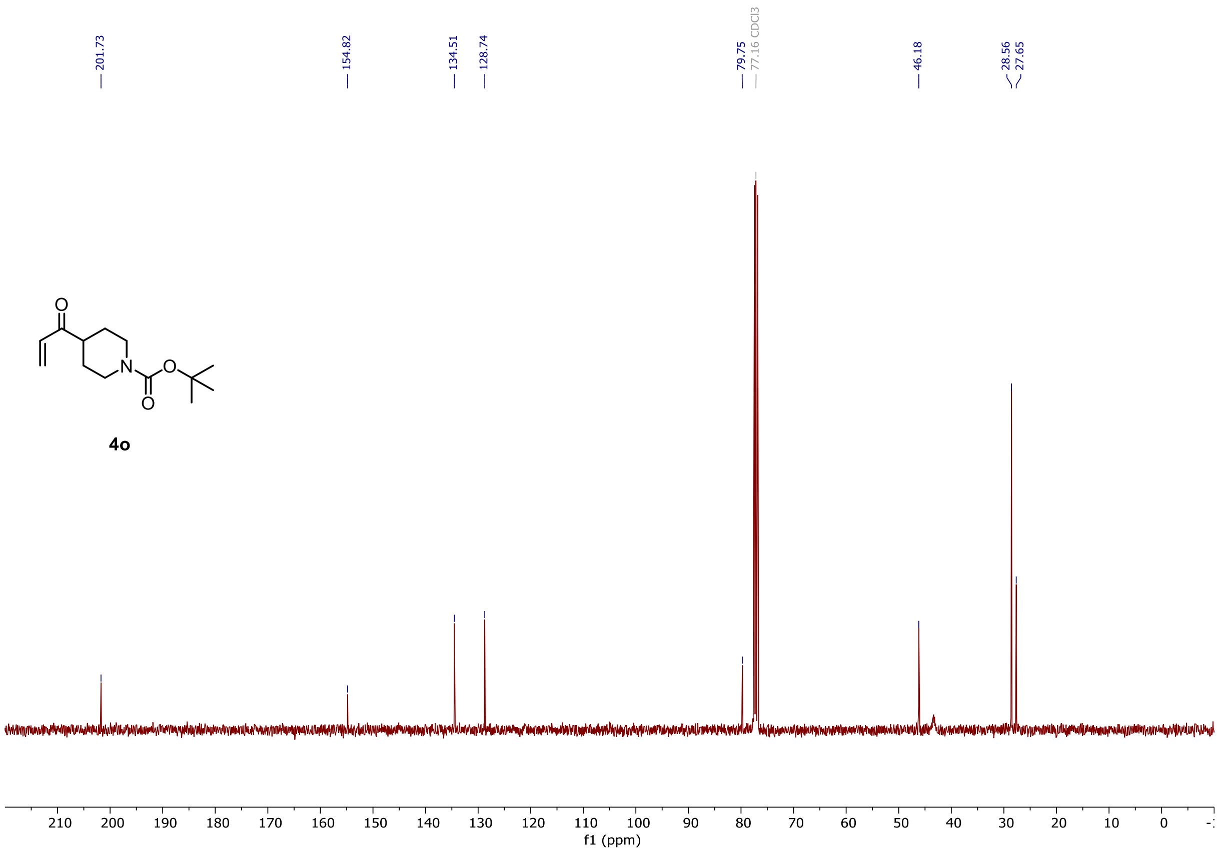

^{1}H NMR (400 MHz, $CDCl_3$) of **4p**

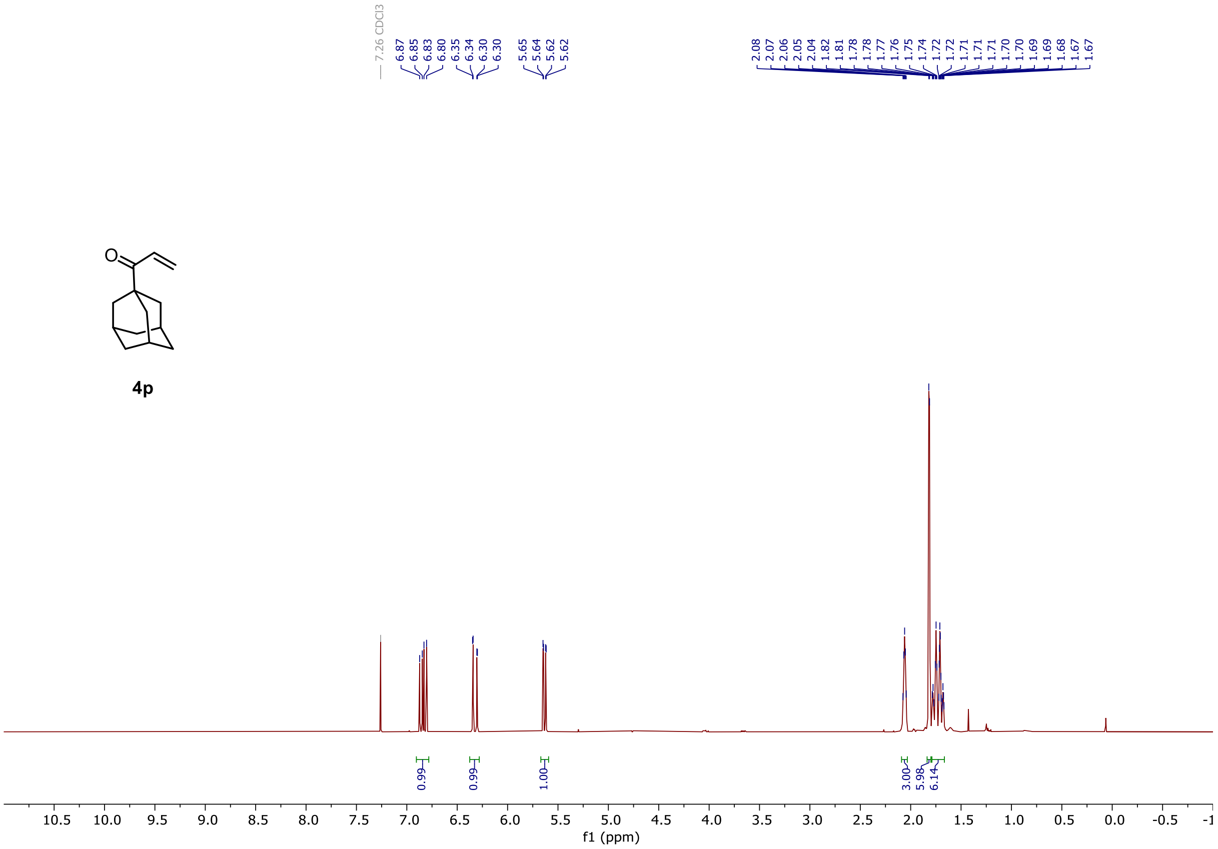

^{13}C NMR (101 MHz, $CDCl_3$) of **4p**

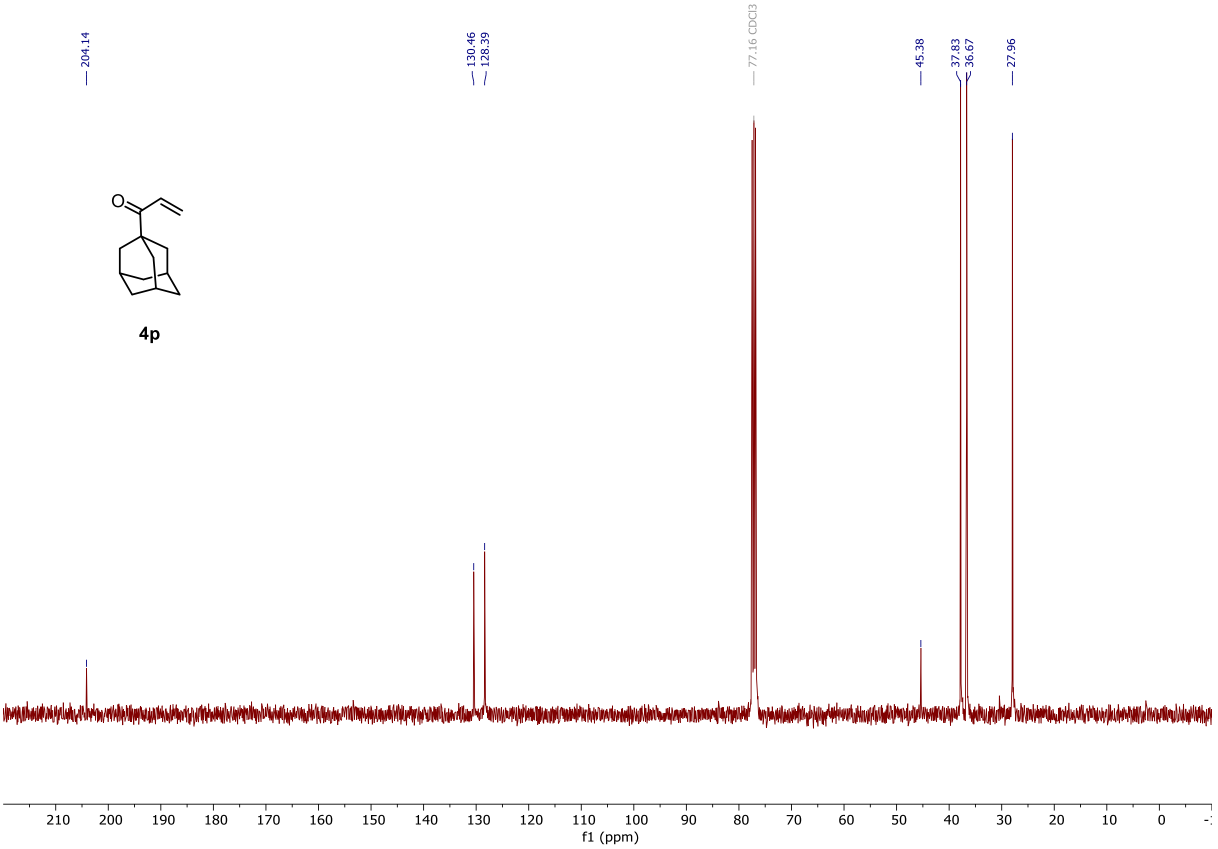

^{1}H NMR (400 MHz, $CDCl_3$) of **4q**

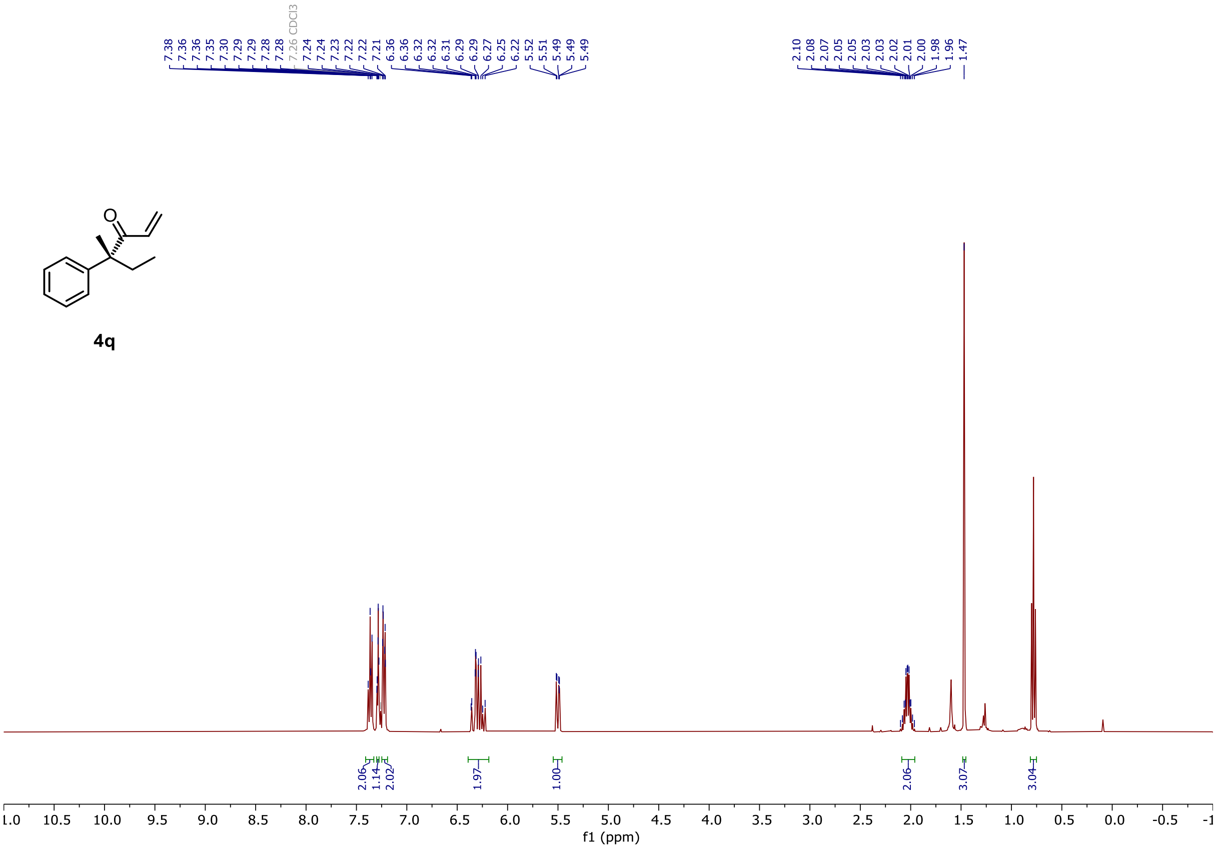

^{13}C NMR (101 MHz, $CDCl_3$) of **4q**

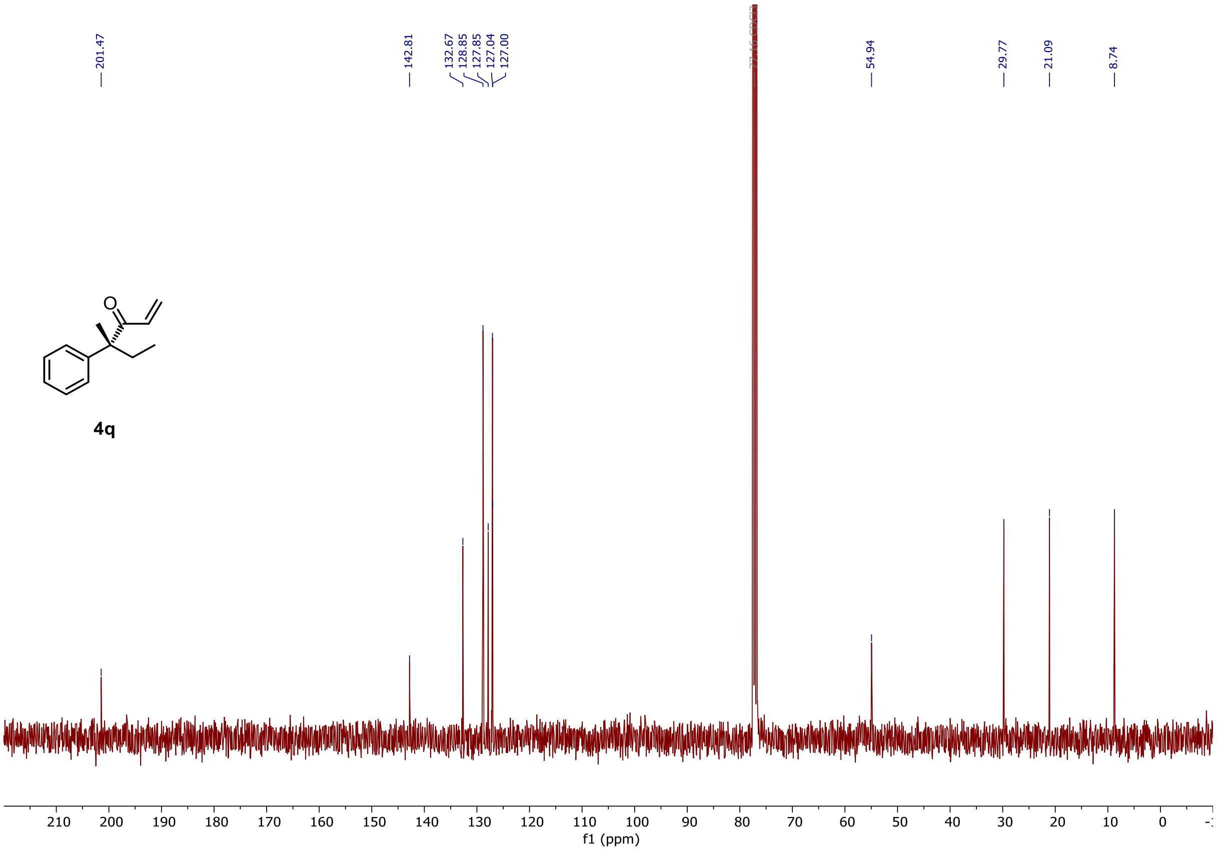

^{1}H NMR (400 MHz, $CDCl_3$) of **4r**

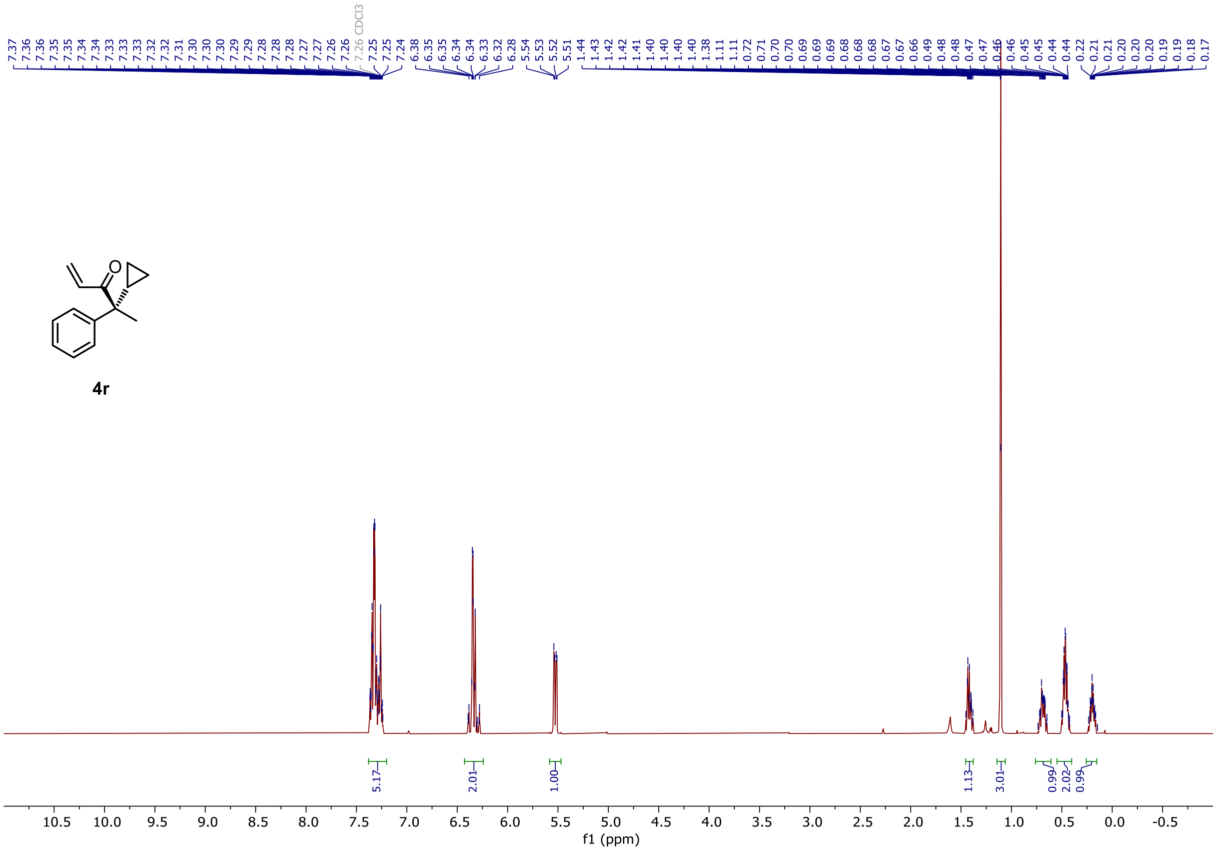

^{13}C NMR (101 MHz, $CDCl_3$) of **4r**

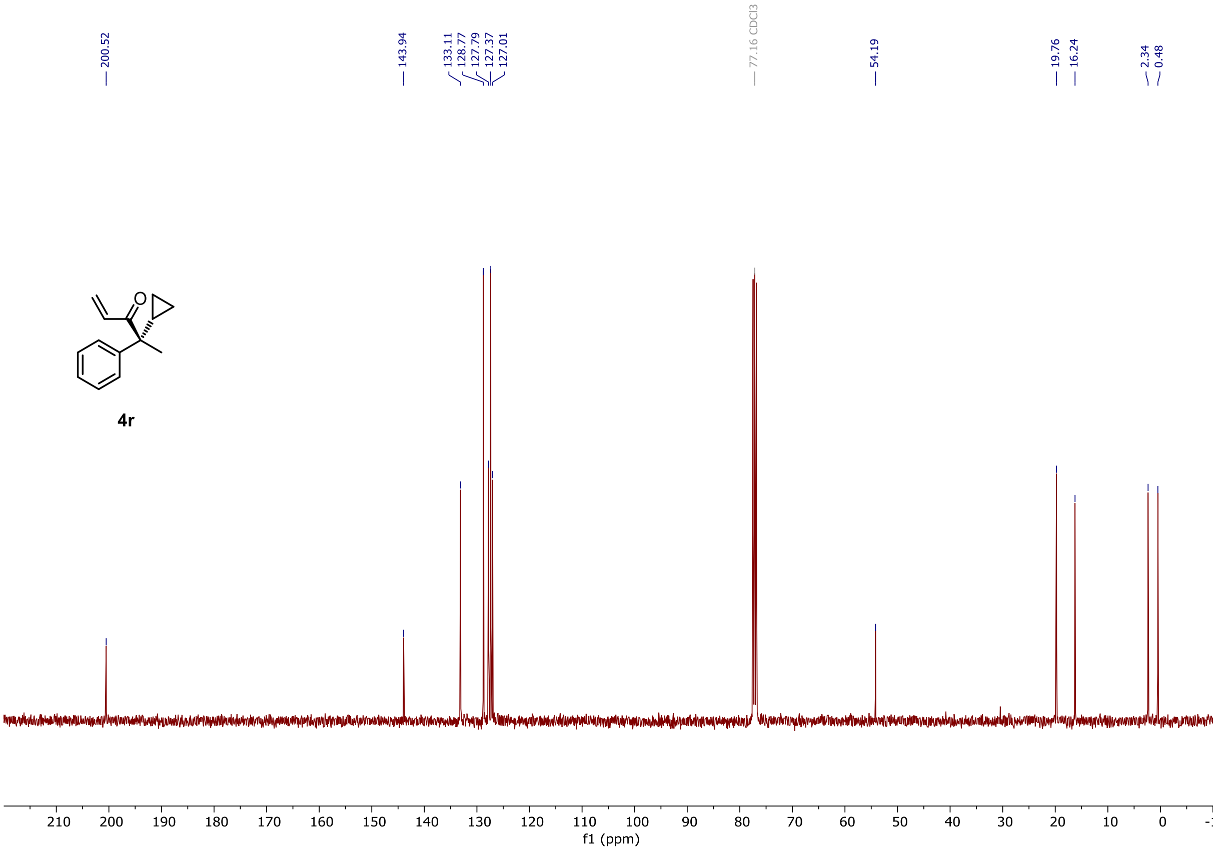

^{1}H NMR (400 MHz, $CDCl_3$) of **4s**

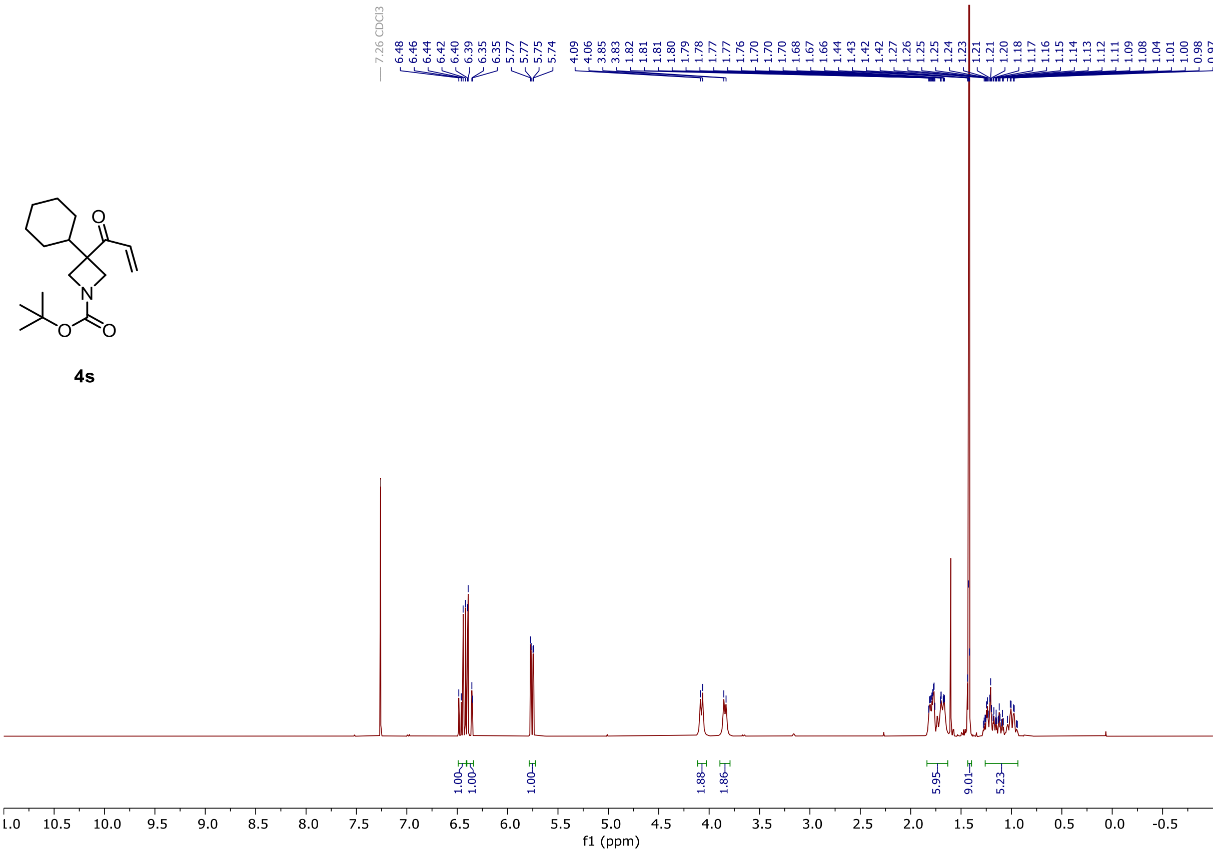

^{13}C NMR (101 MHz, $CDCl_3$) of **4s**

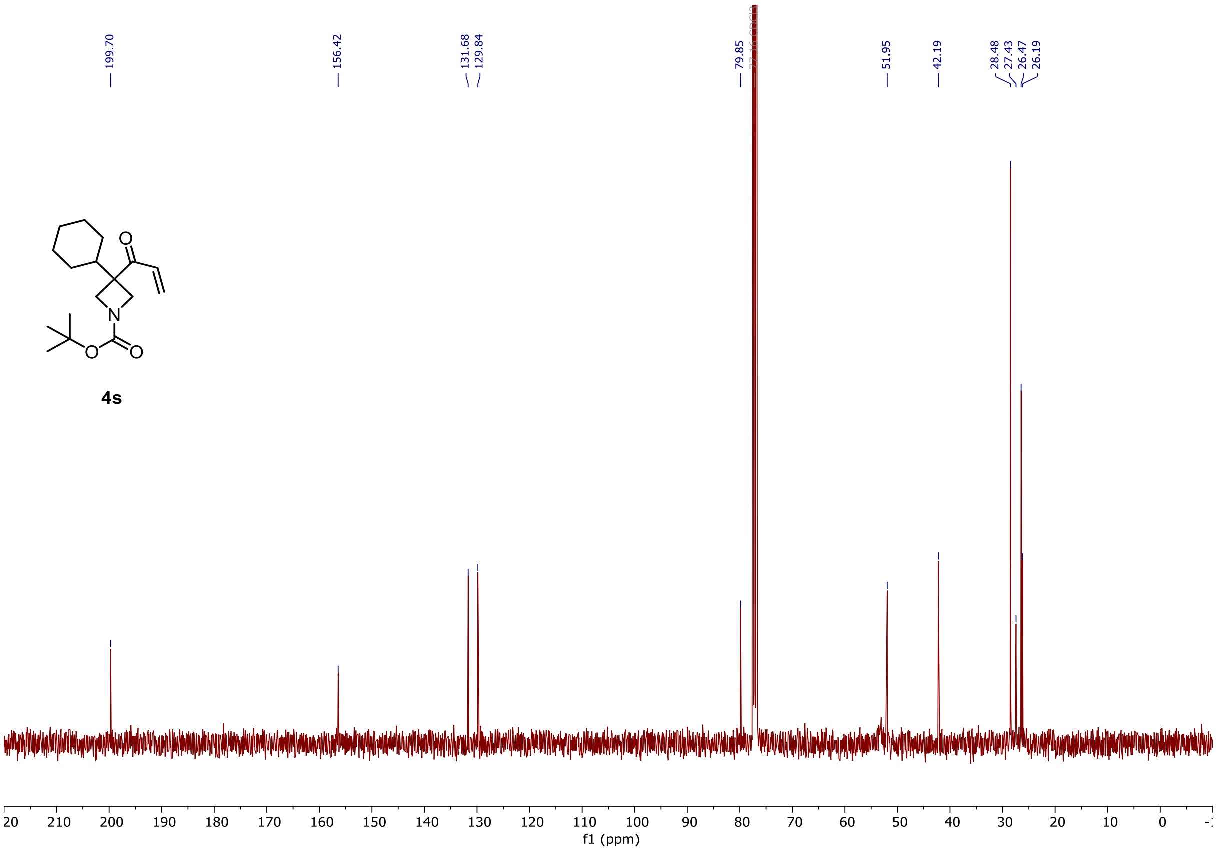

^{1}H NMR (400 MHz, $CDCl_3$) of **4t**

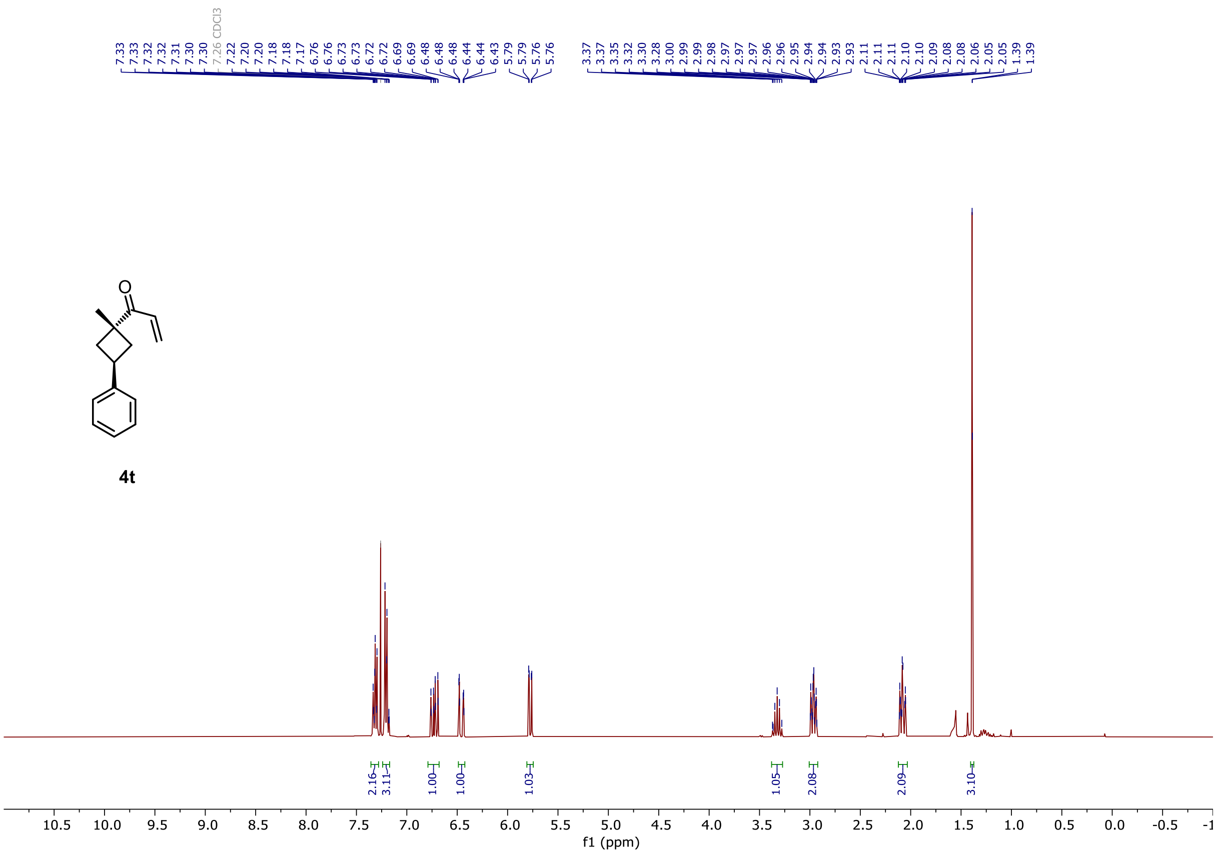

^{13}C NMR (101 MHz, $CDCl_3$) of **4t**

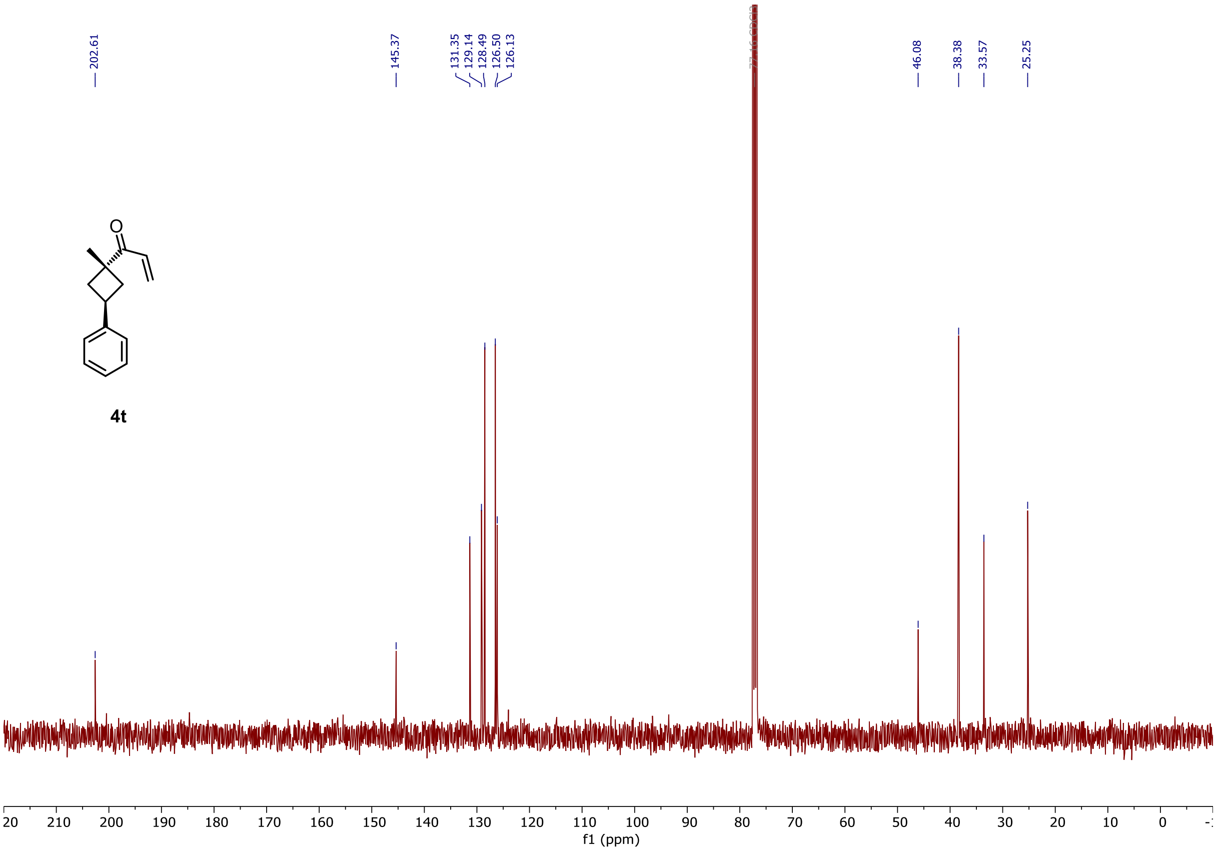

^{1}H NMR (400 MHz, $CDCl_3$) of **7**

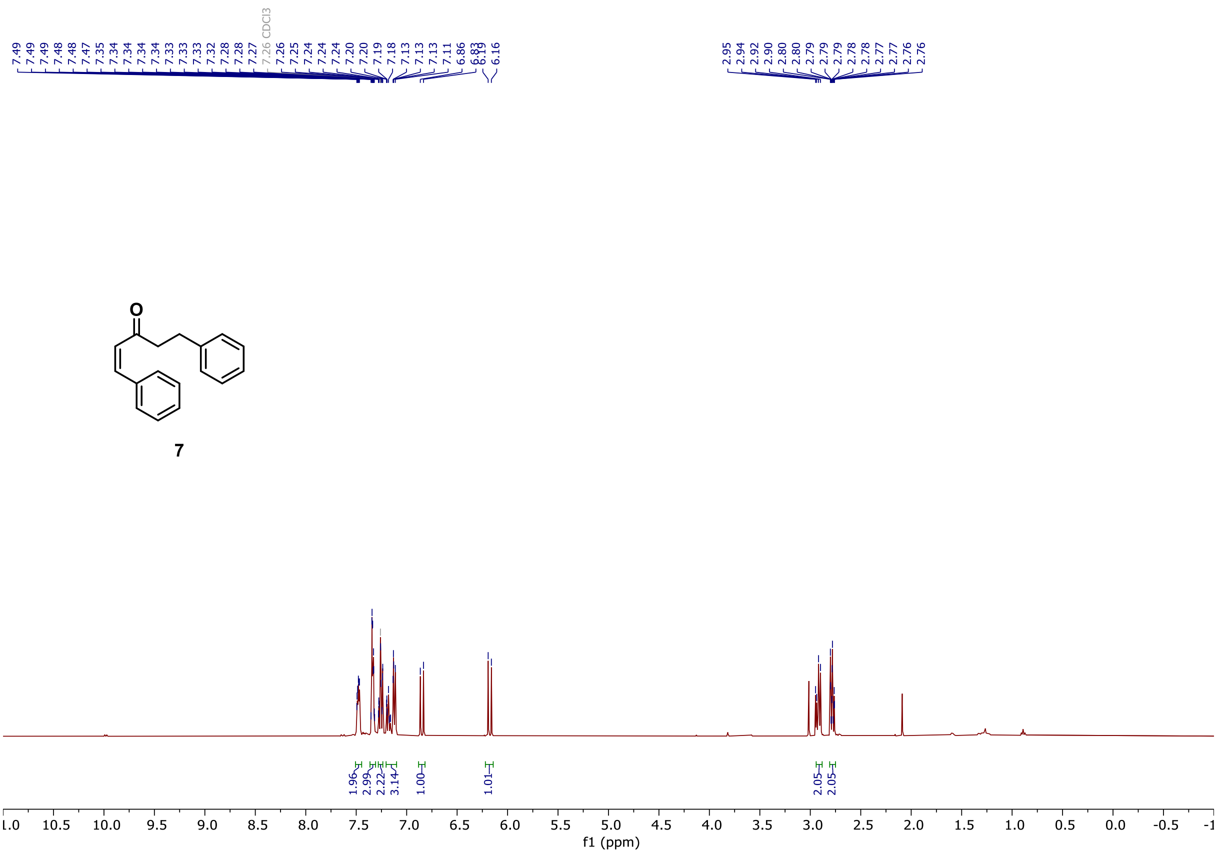

^{13}C NMR (101 MHz, $CDCl_3$) of **7**

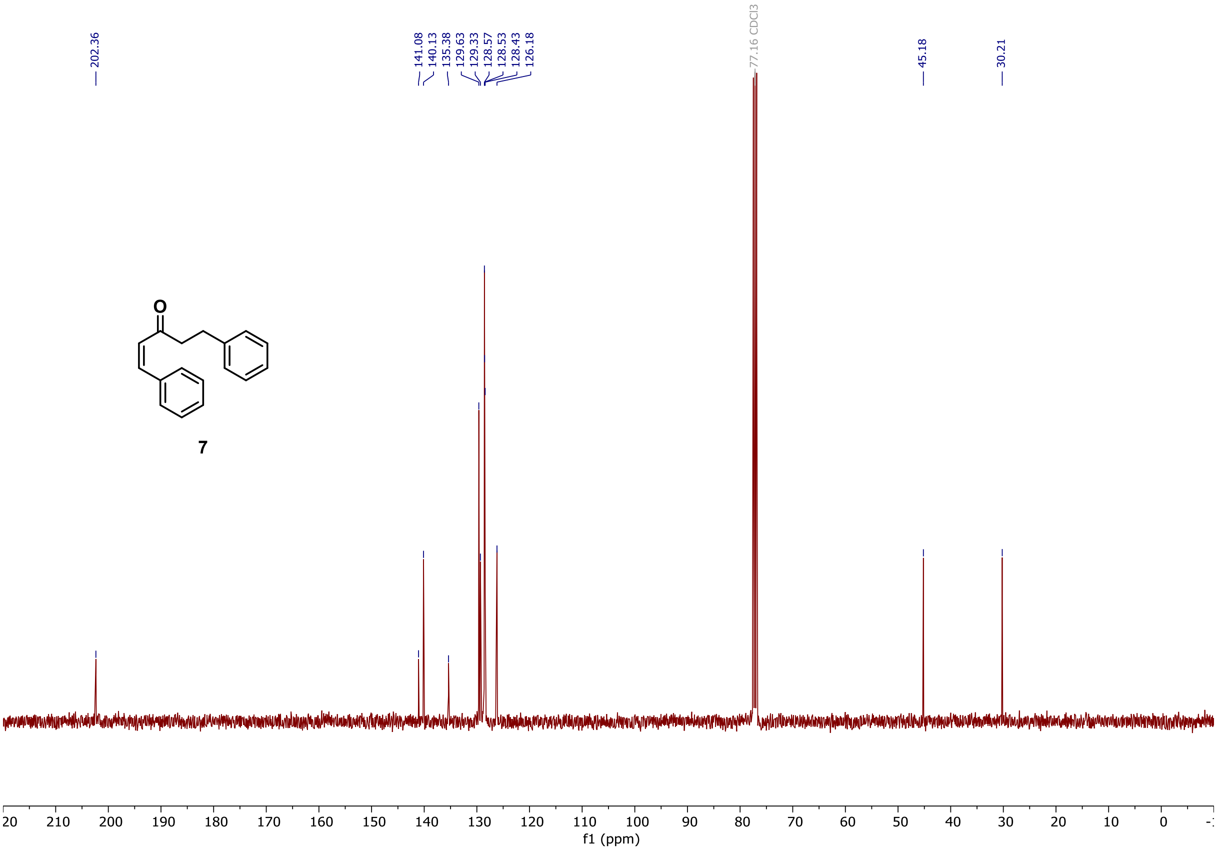

^{1}H NMR (400 MHz, $CDCl_3$) of **8a**

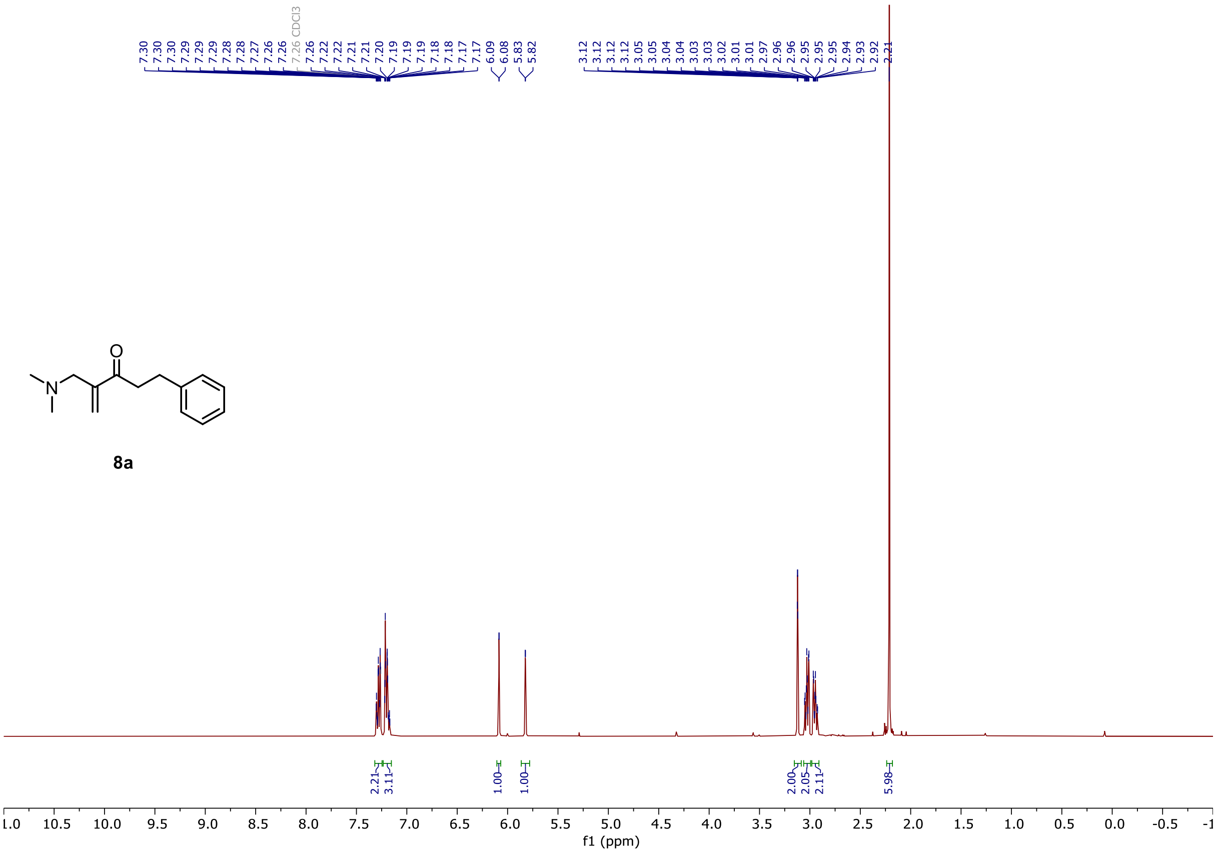

^{13}C NMR (101 MHz, $CDCl_3$) of **8a**

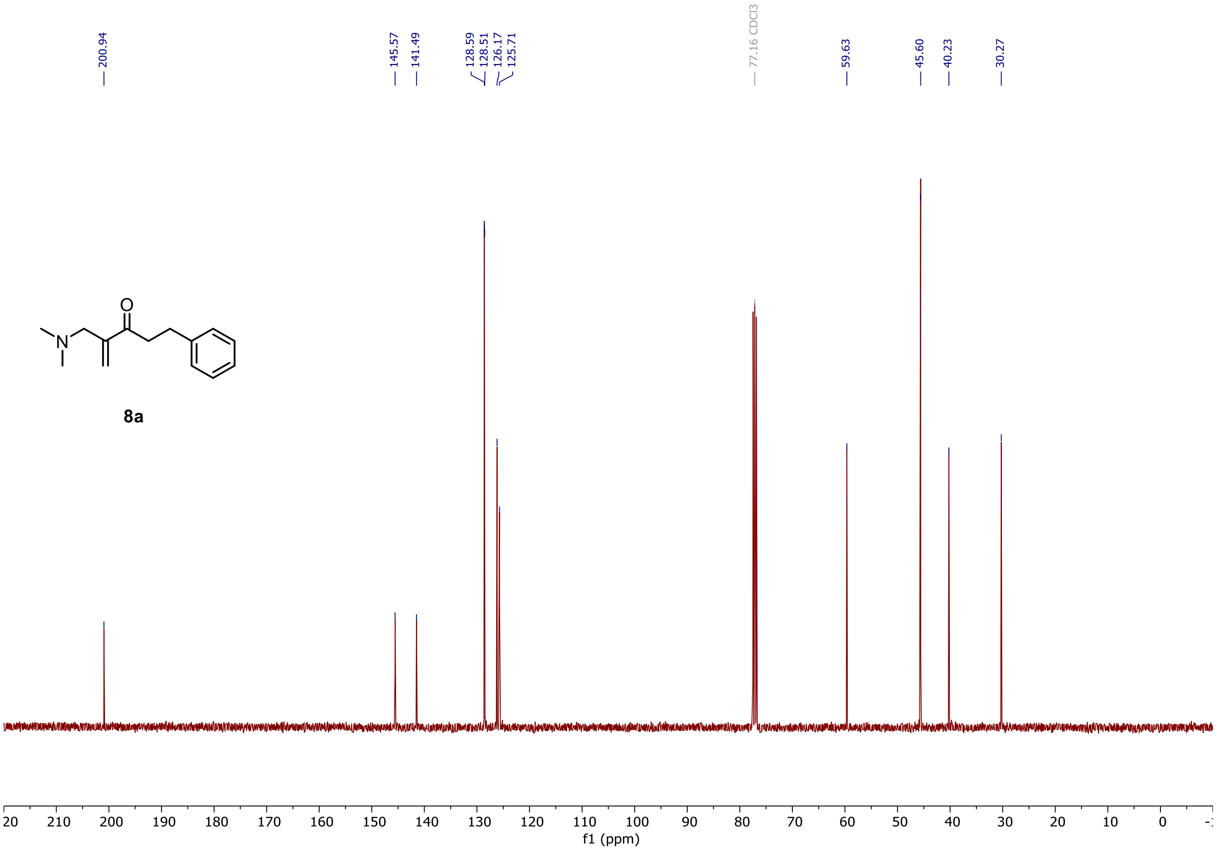

^{1}H NMR (400 MHz, $CDCl_3$) of **8b**

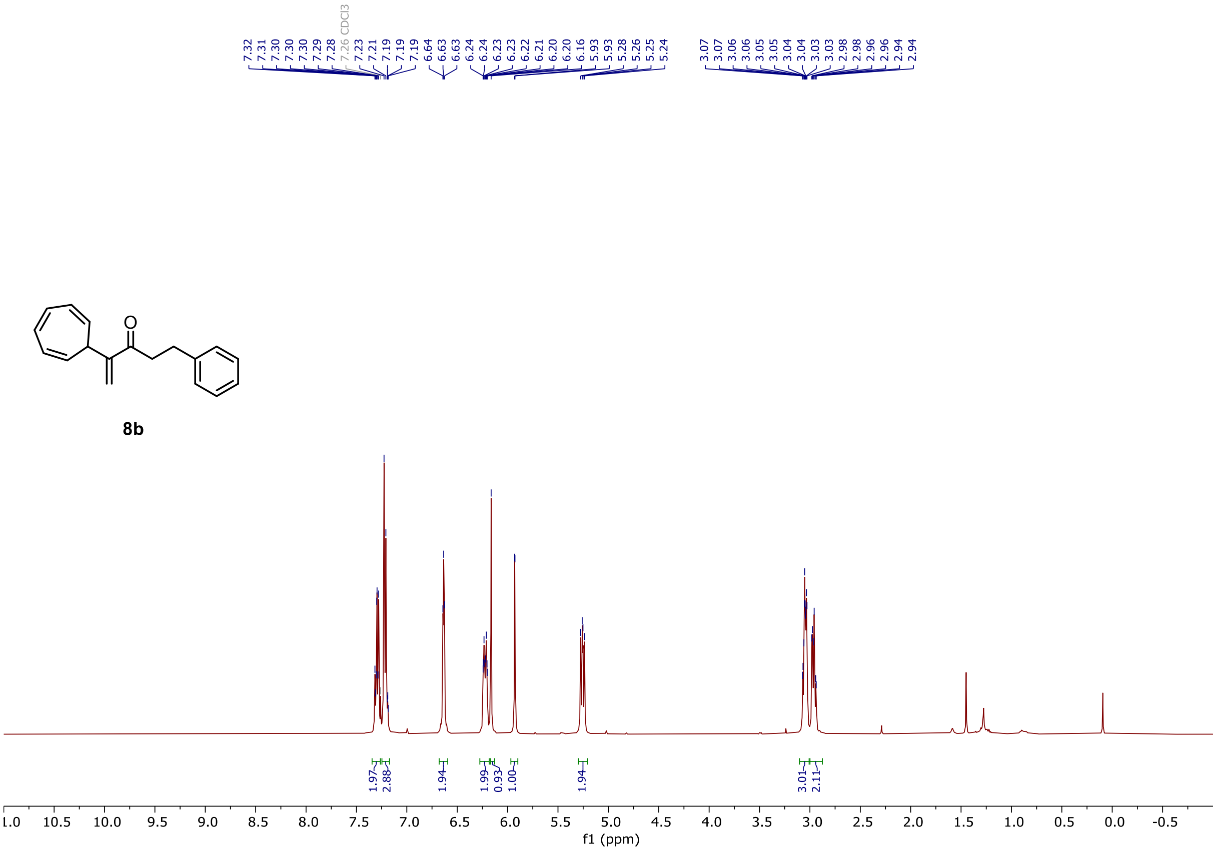

^{13}C NMR (101 MHz, $CDCl_3$) of **8b**

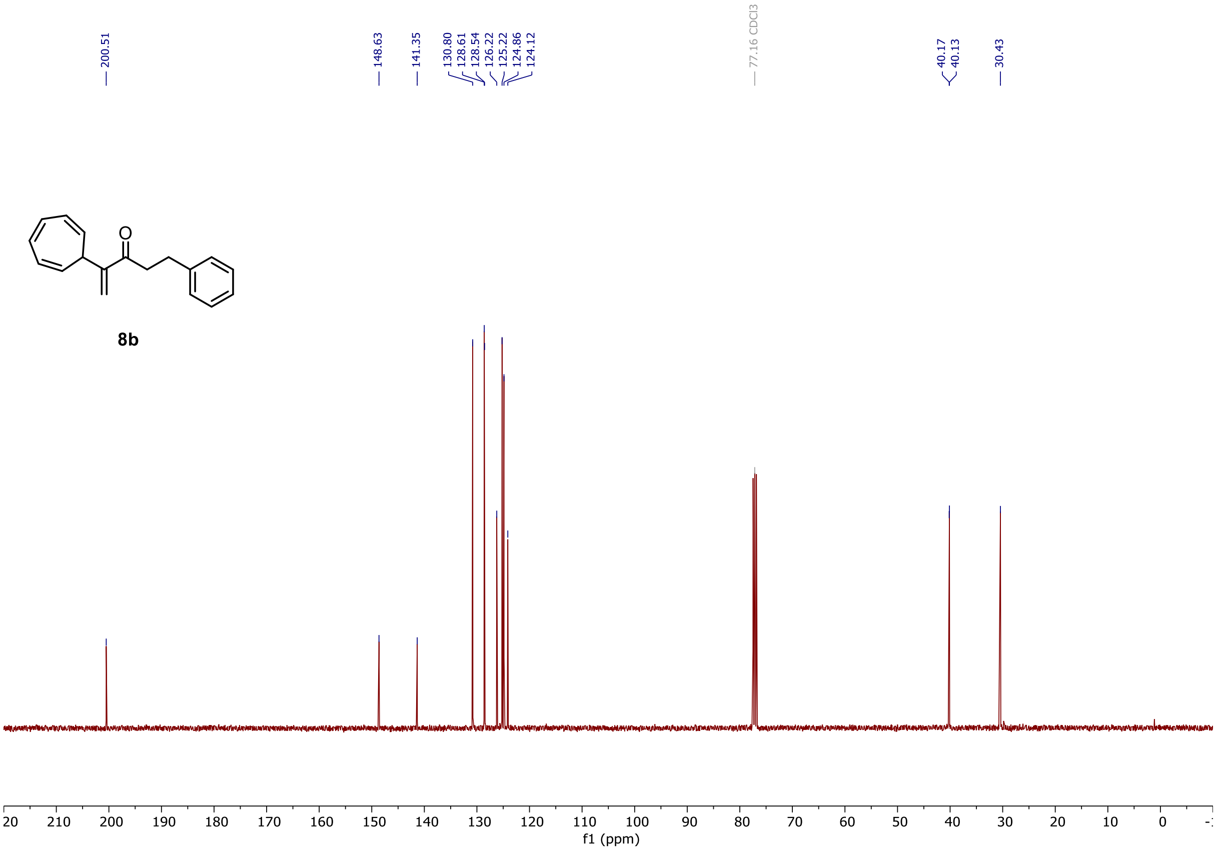

^{1}H NMR (400 MHz, $CDCl_3$) of **8c**

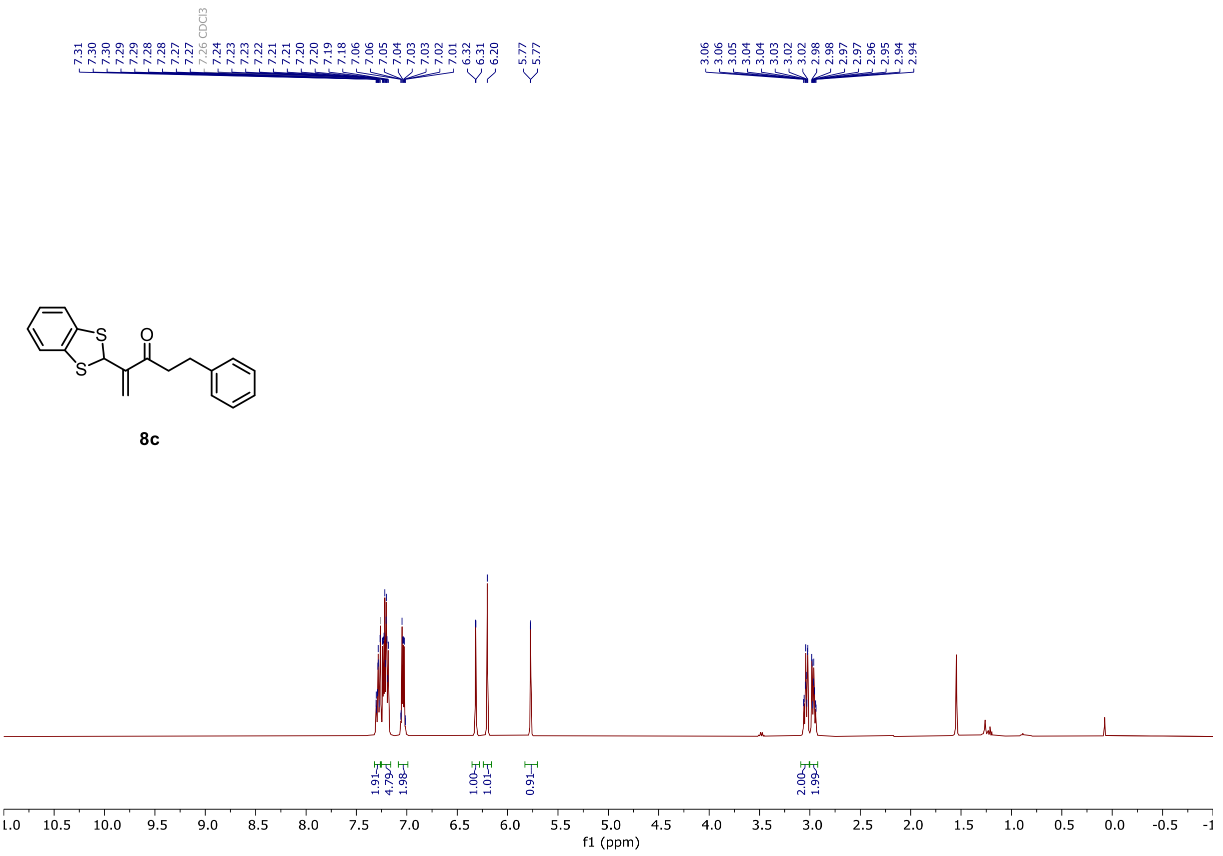

^{13}C NMR (101 MHz, $CDCl_3$) of **8c**

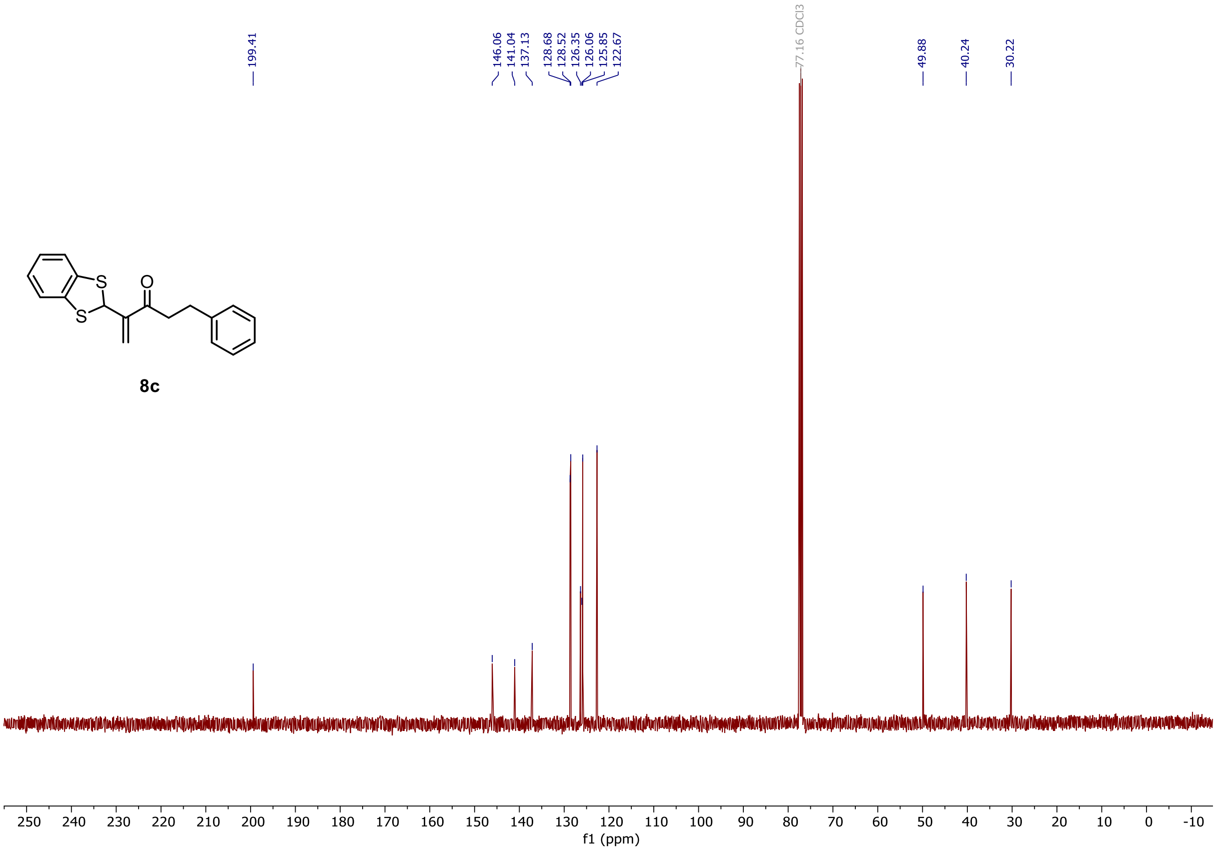

Crude ^{1}H NMR (400 MHz, $CDCl_3$) of **8d**

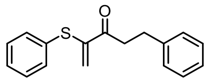

8d

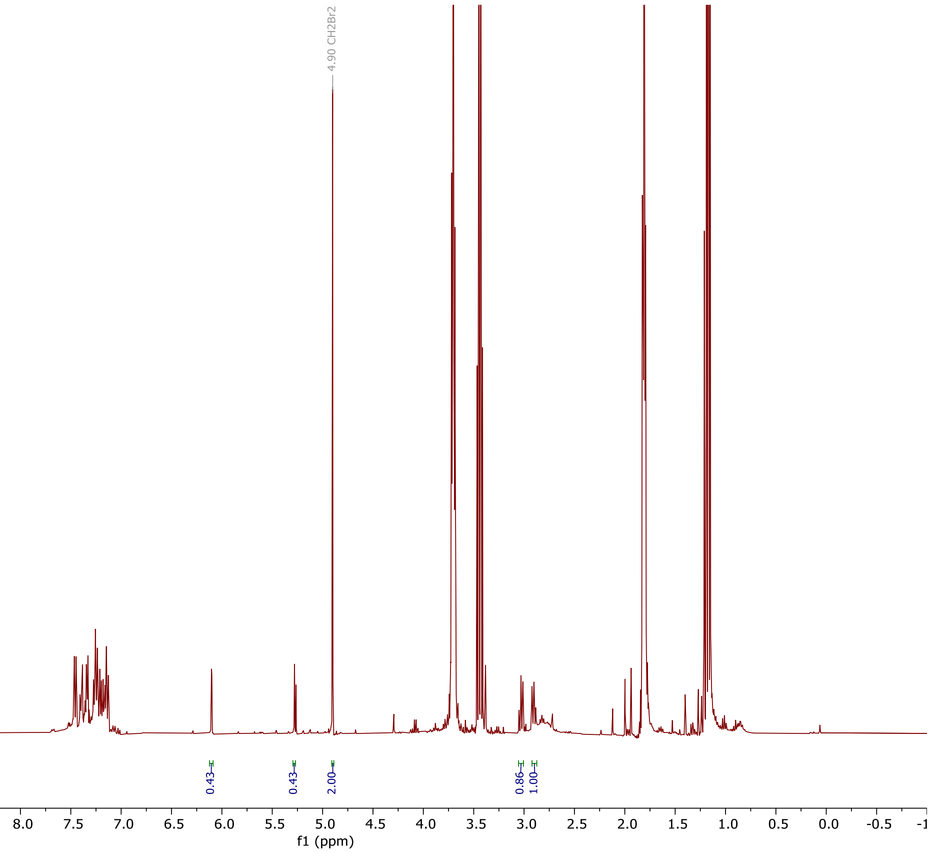

Crude ^{1}H NMR (400 MHz, $CDCl_3$) of **8e**

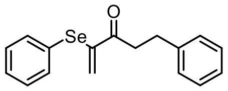

8e

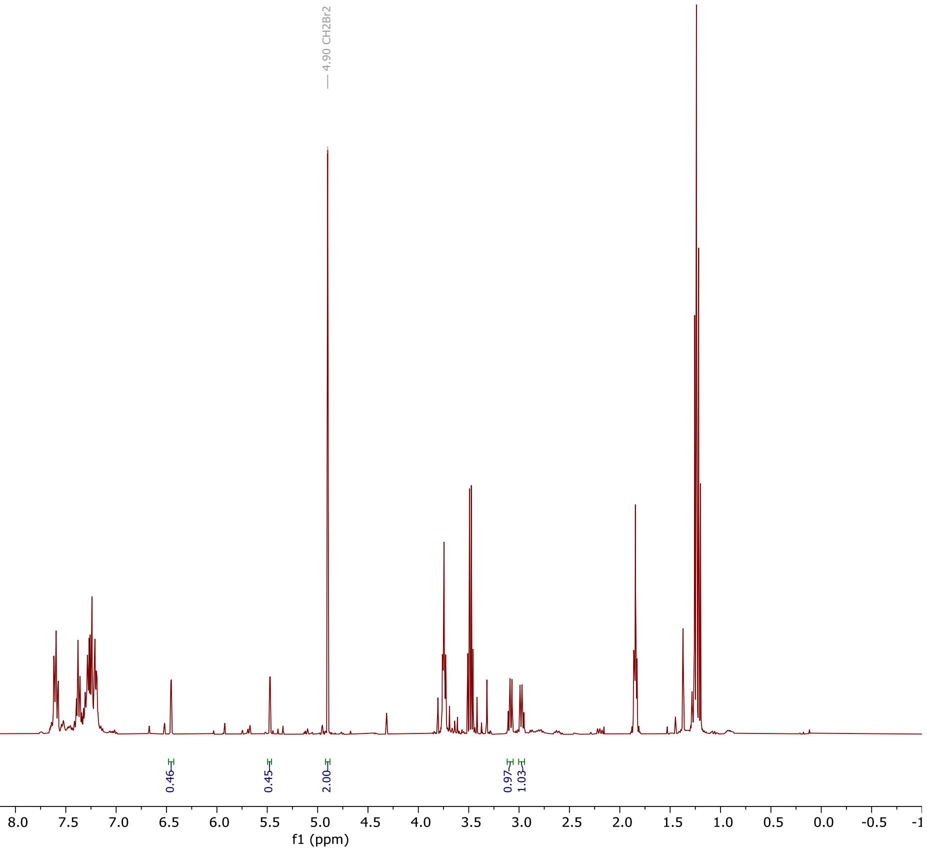

^{1}H NMR (400 MHz, $CDCl_3$) of **8f**

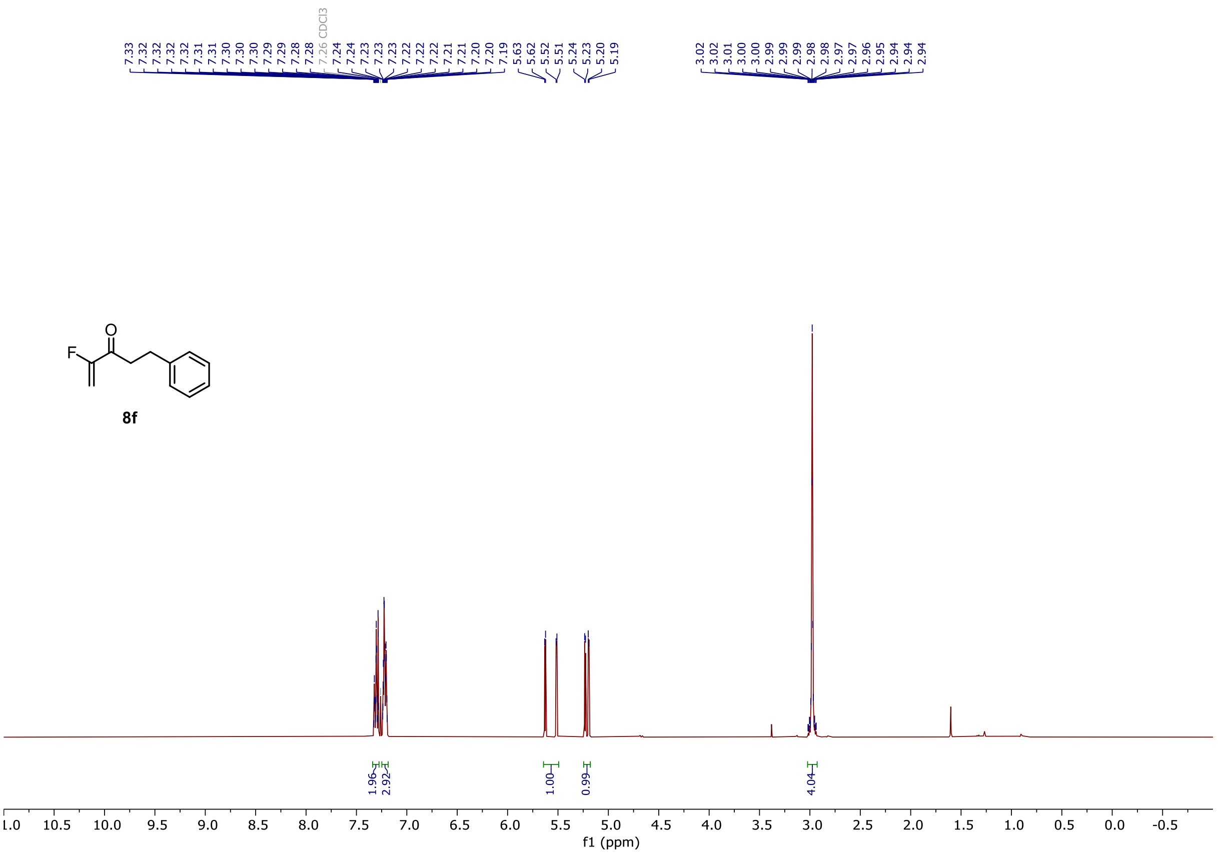

^{13}C NMR (101 MHz, $CDCl_3$) of **8f**

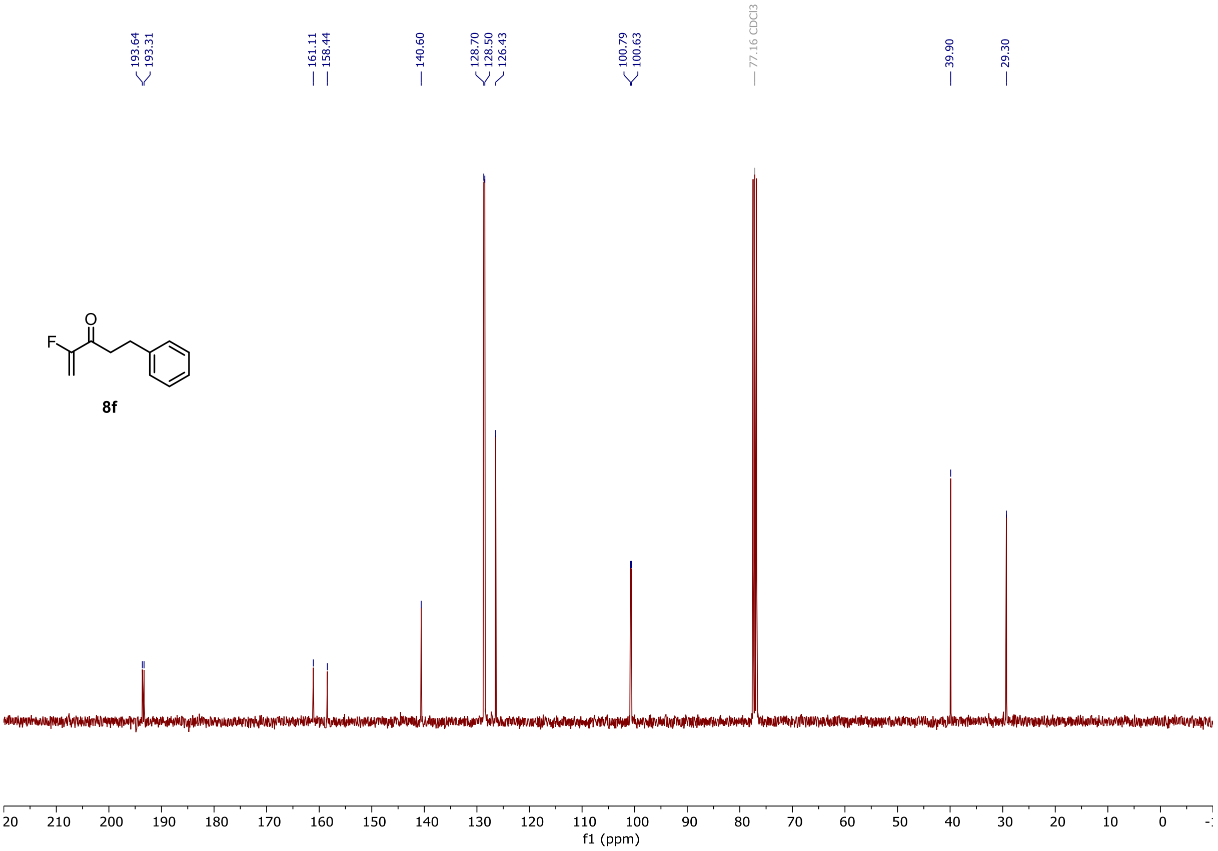

^{19}F NMR (376 MHz, $CDCl_3$) of **8f**

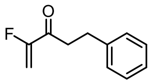

8f

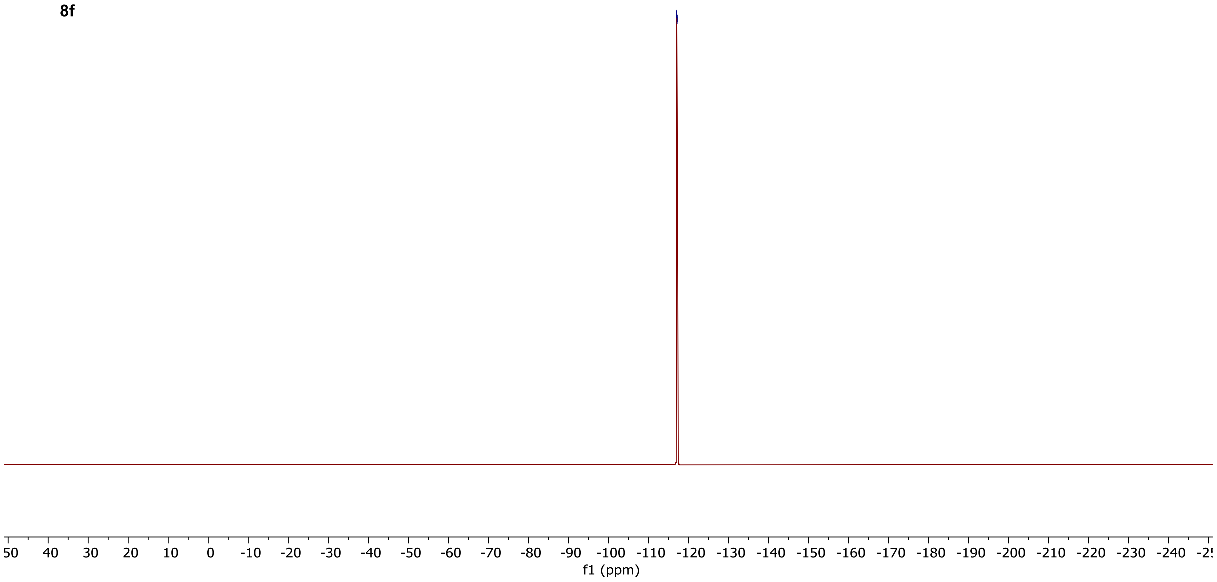

^{1}H NMR (400 MHz, $CDCl_3$) of **8g**

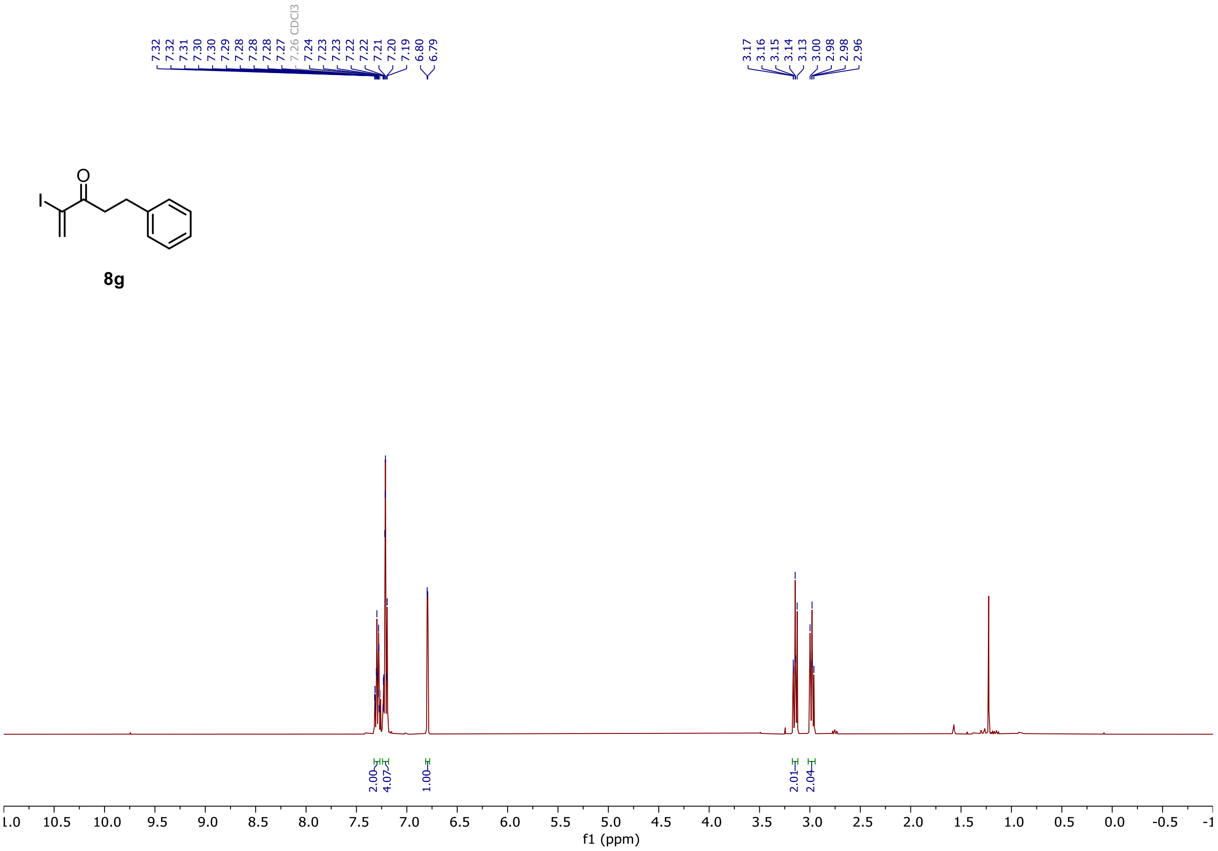

^{13}C NMR (101 MHz, $CDCl_3$) of **8g**

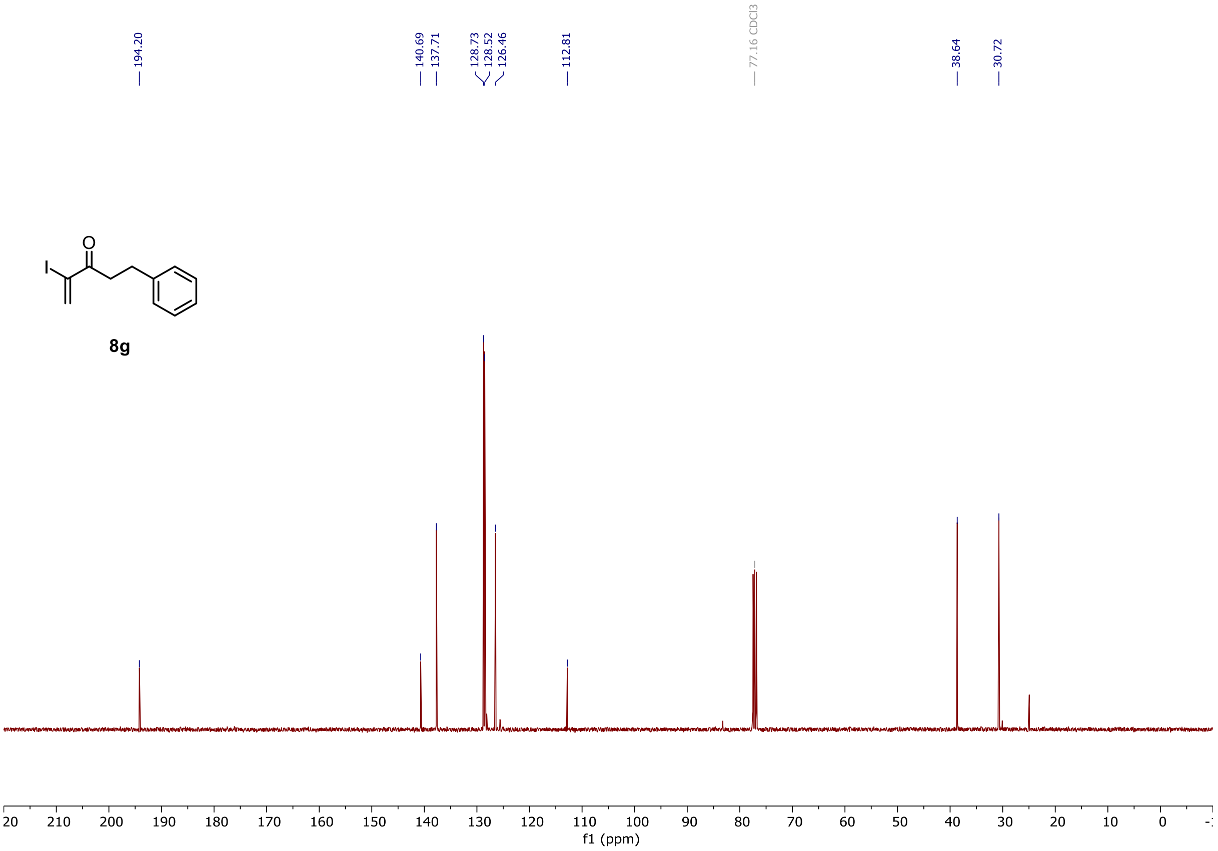

^{1}H NMR (400 MHz, $CDCl_3$) of **9**

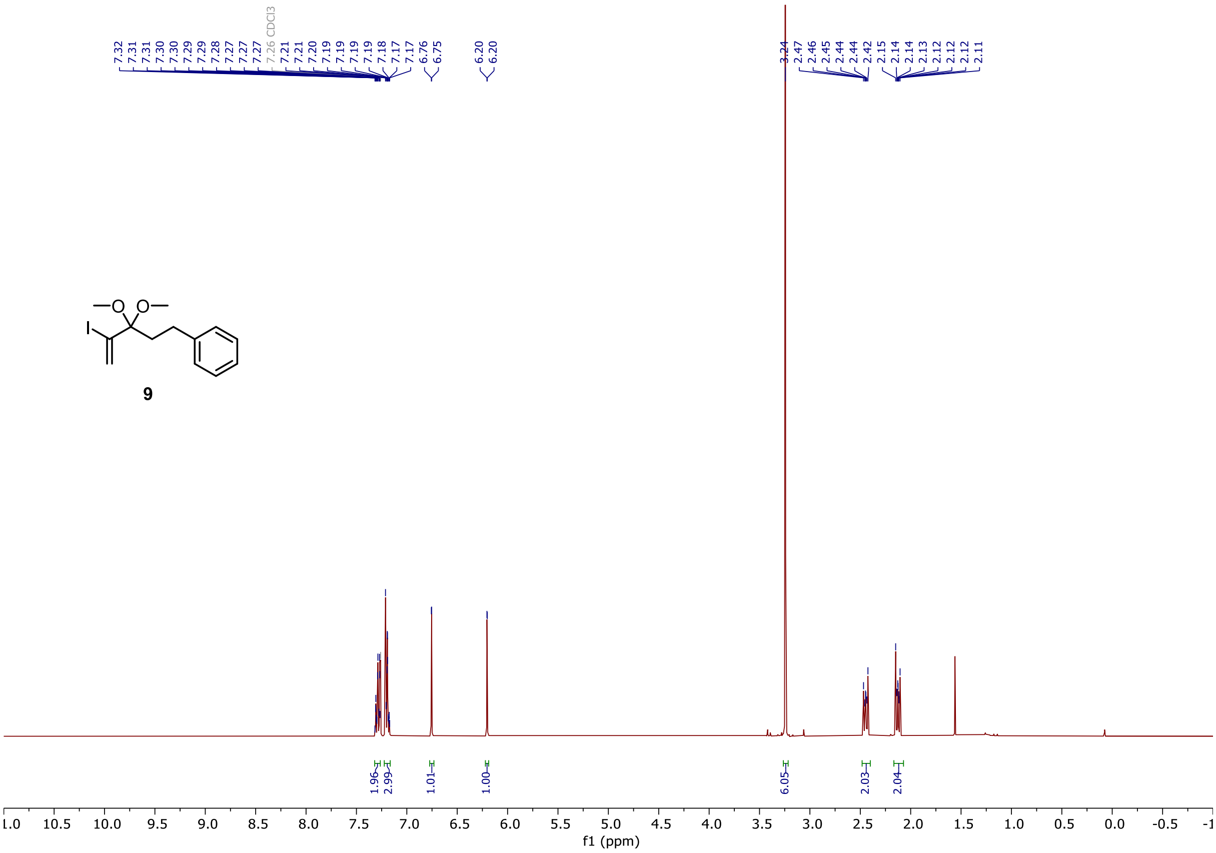

^{13}C NMR (101 MHz, $CDCl_3$) of **9**

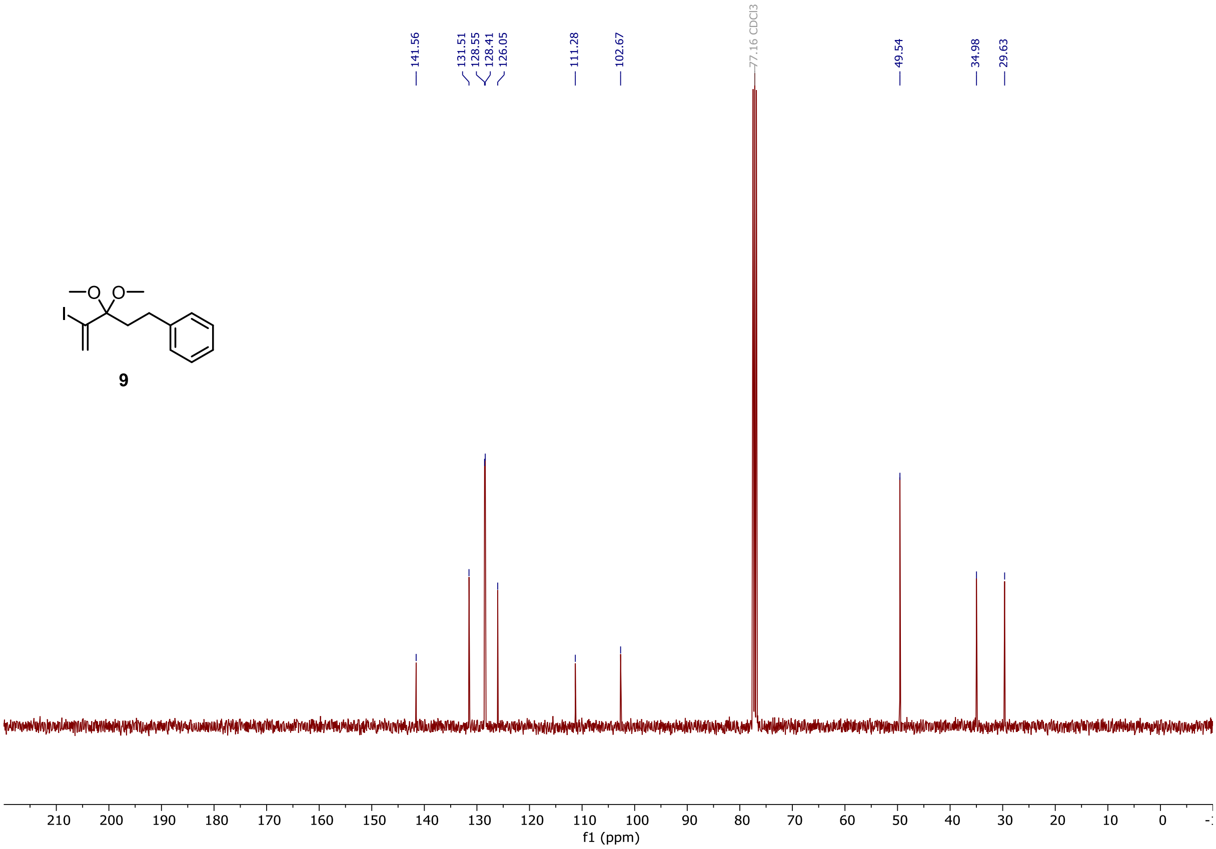

^{1}H NMR (400 MHz, $CDCl_3$) of **10**

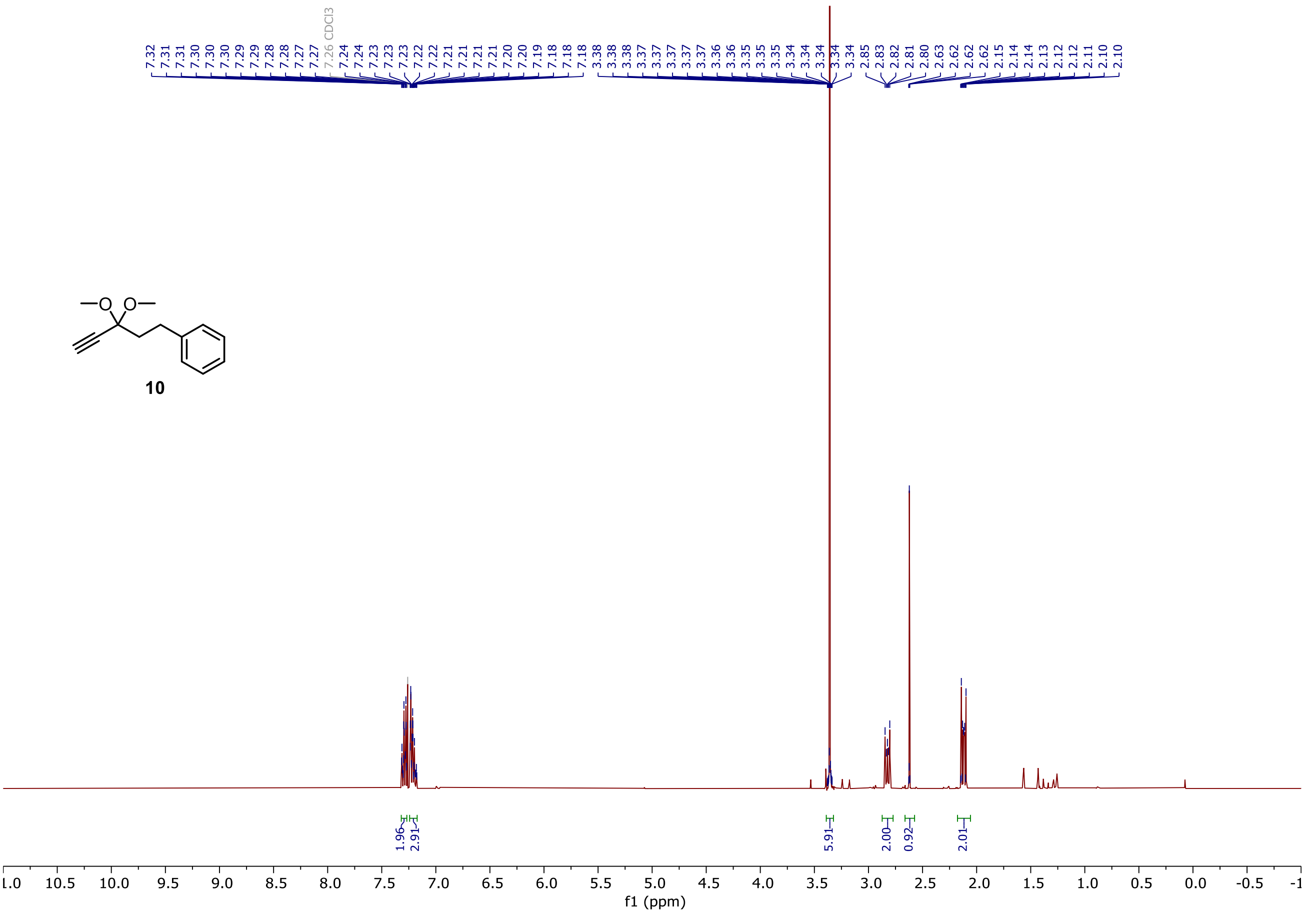

^{13}C NMR (101 MHz, $CDCl_3$) of **10**

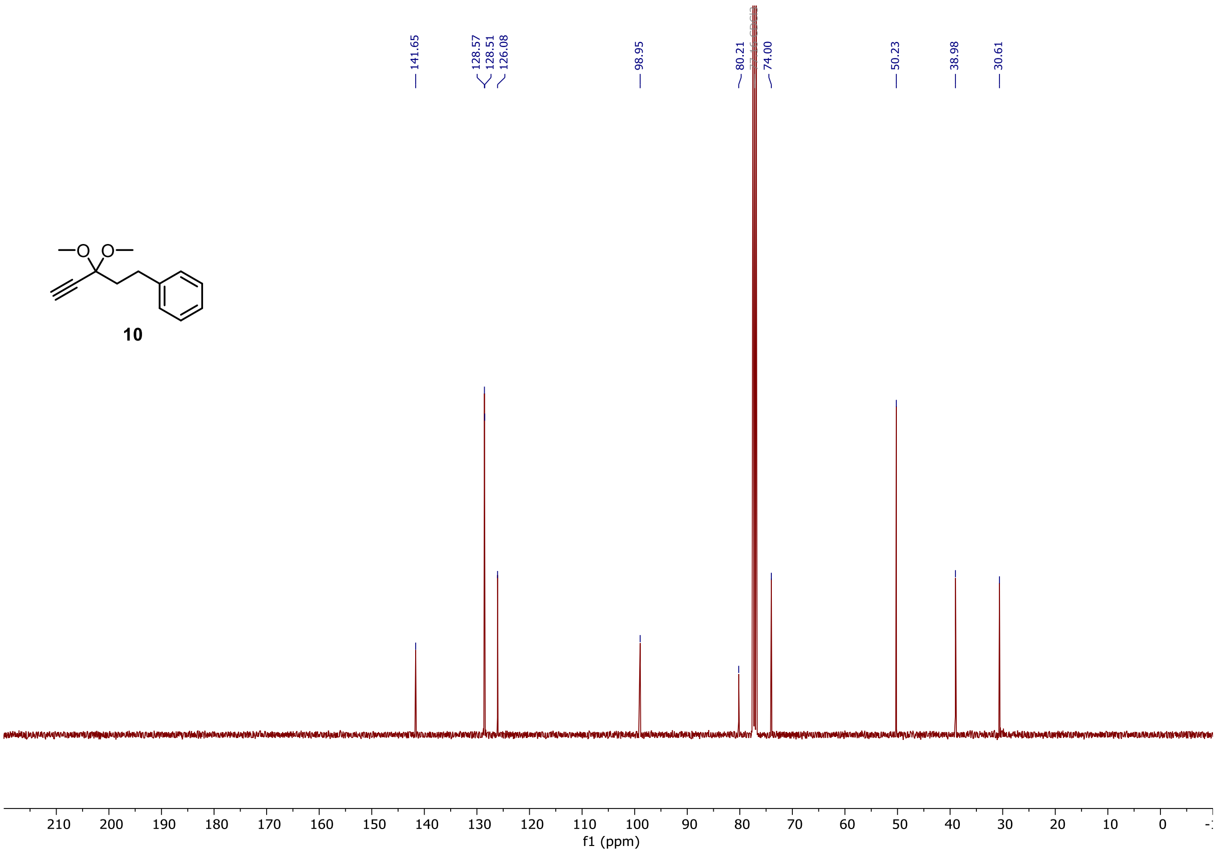

^{1}H NMR (400 MHz, $CDCl_3$) of **11**

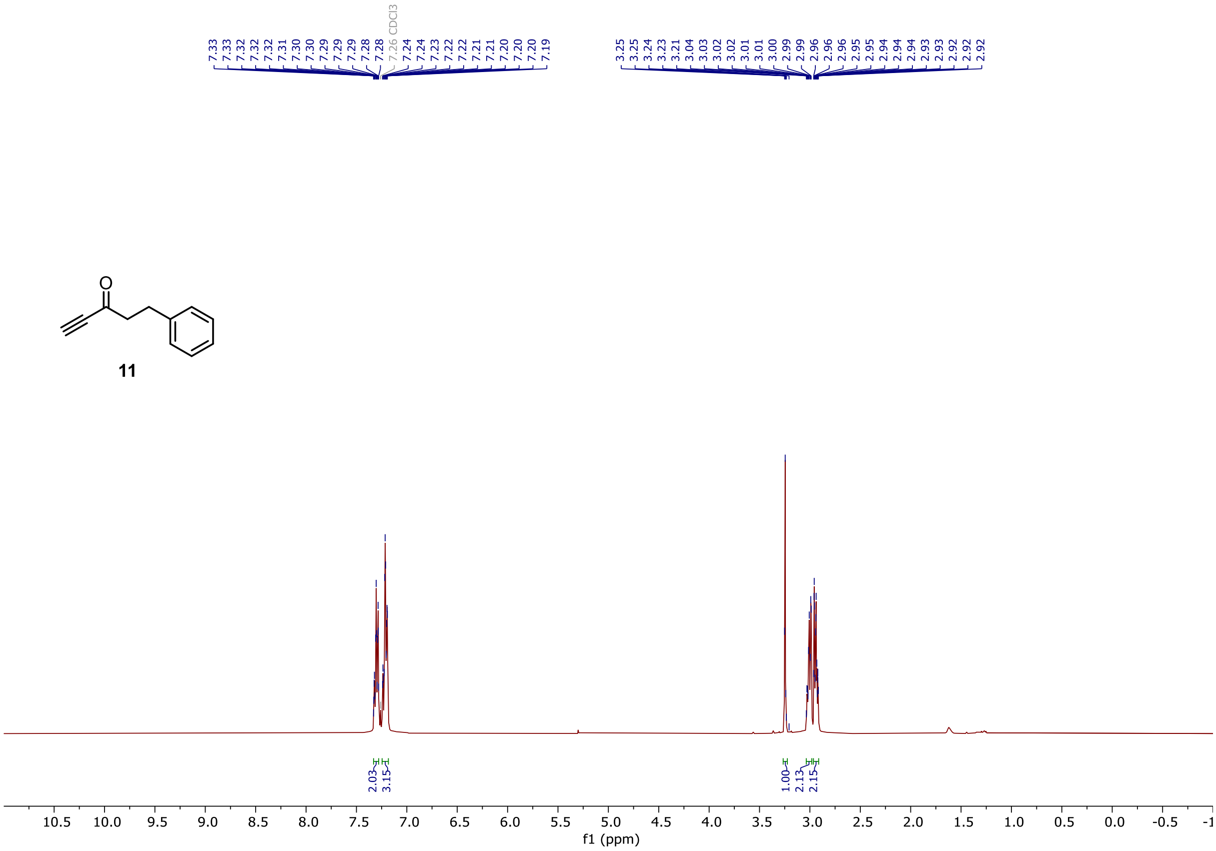

^{13}C NMR (101 MHz, $CDCl_3$) of **11**

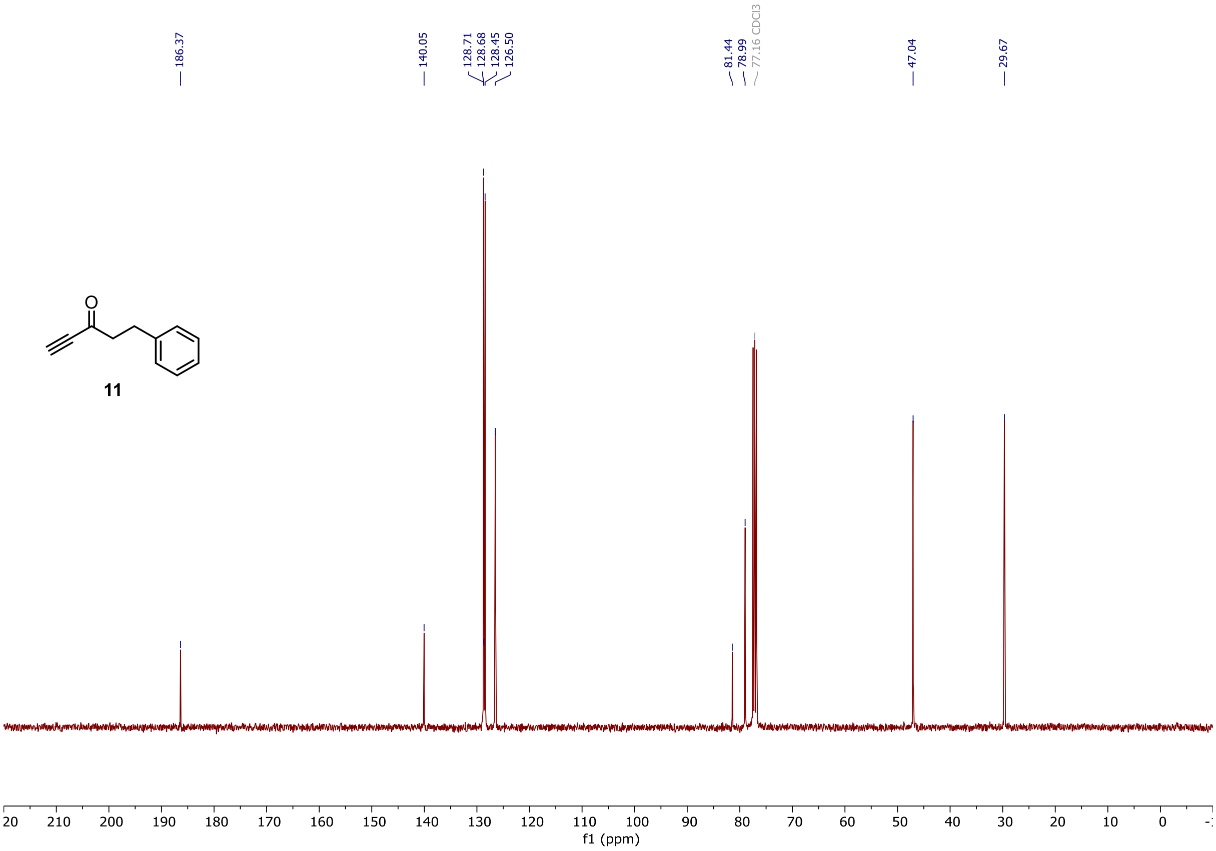

Crude ^{1}H NMR (400 MHz, $CDCl_3$) of **SI-4**

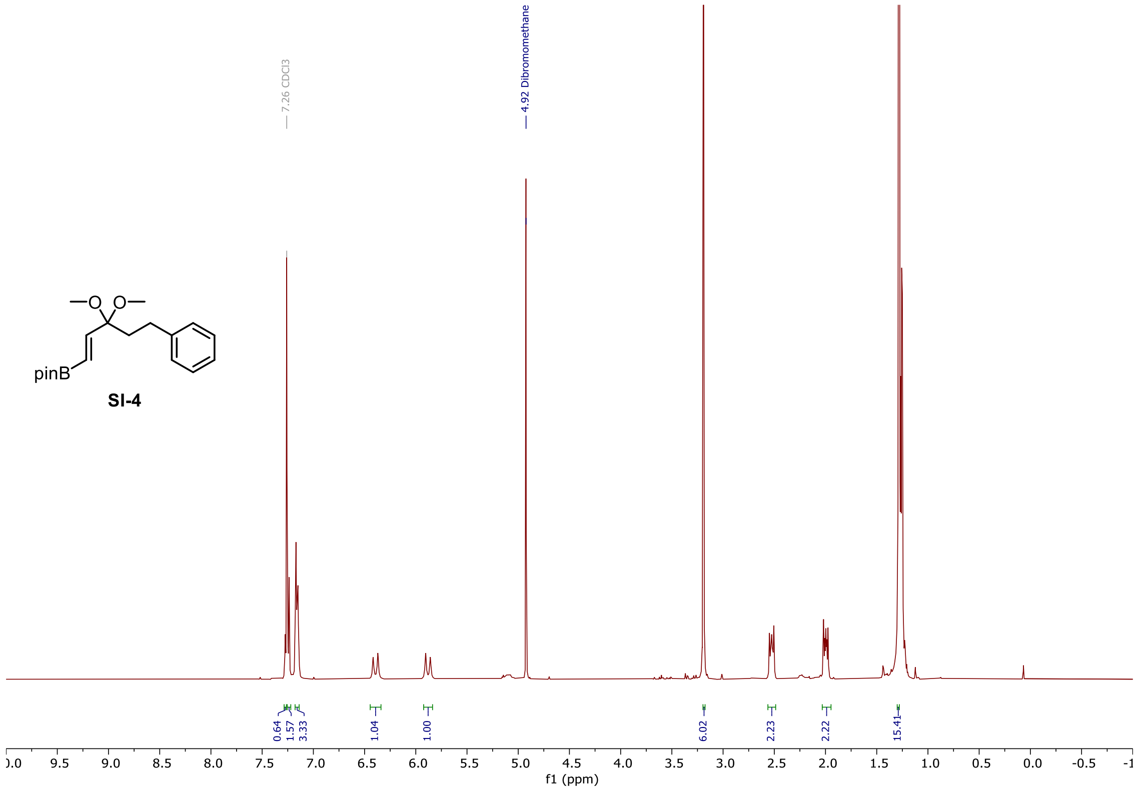

N.B. Singlet at 4.9 is dibromomethane (internal standard)

Crude ^{1}H NMR (101 MHz, $CDCl_3$) of **SI-4**

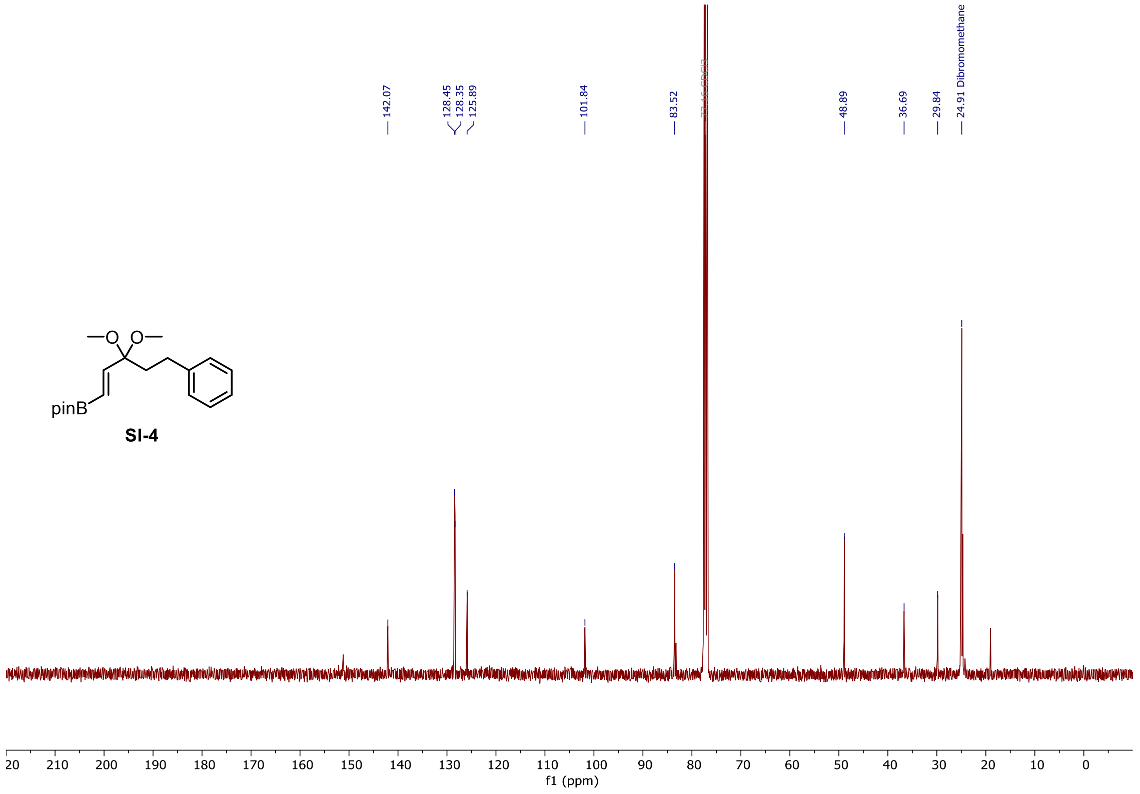

N.B. Peak at 24.9 is dibromomethane (internal standard)

^{1}H NMR (400 MHz, $CDCl_3$) of **12**

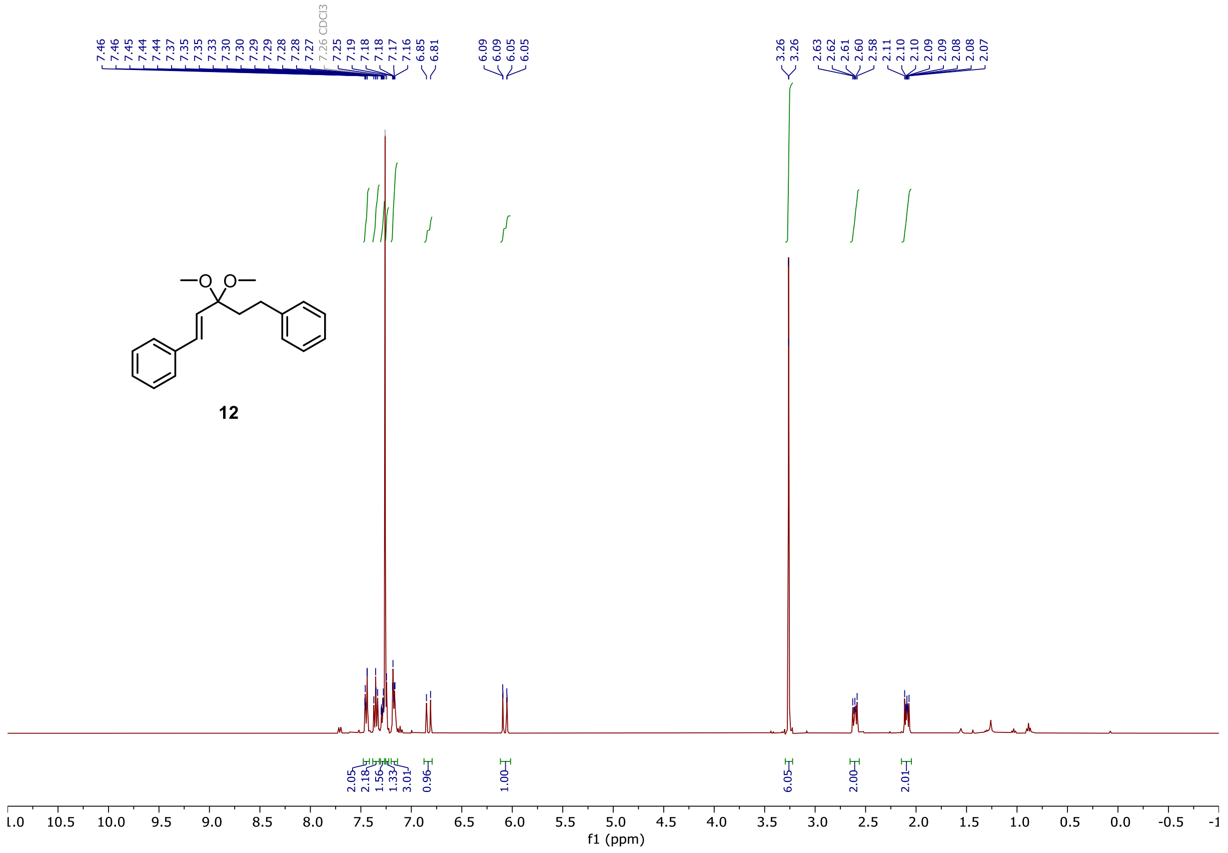

^{13}C NMR (151 MHz, $CDCl_3$) of **12**

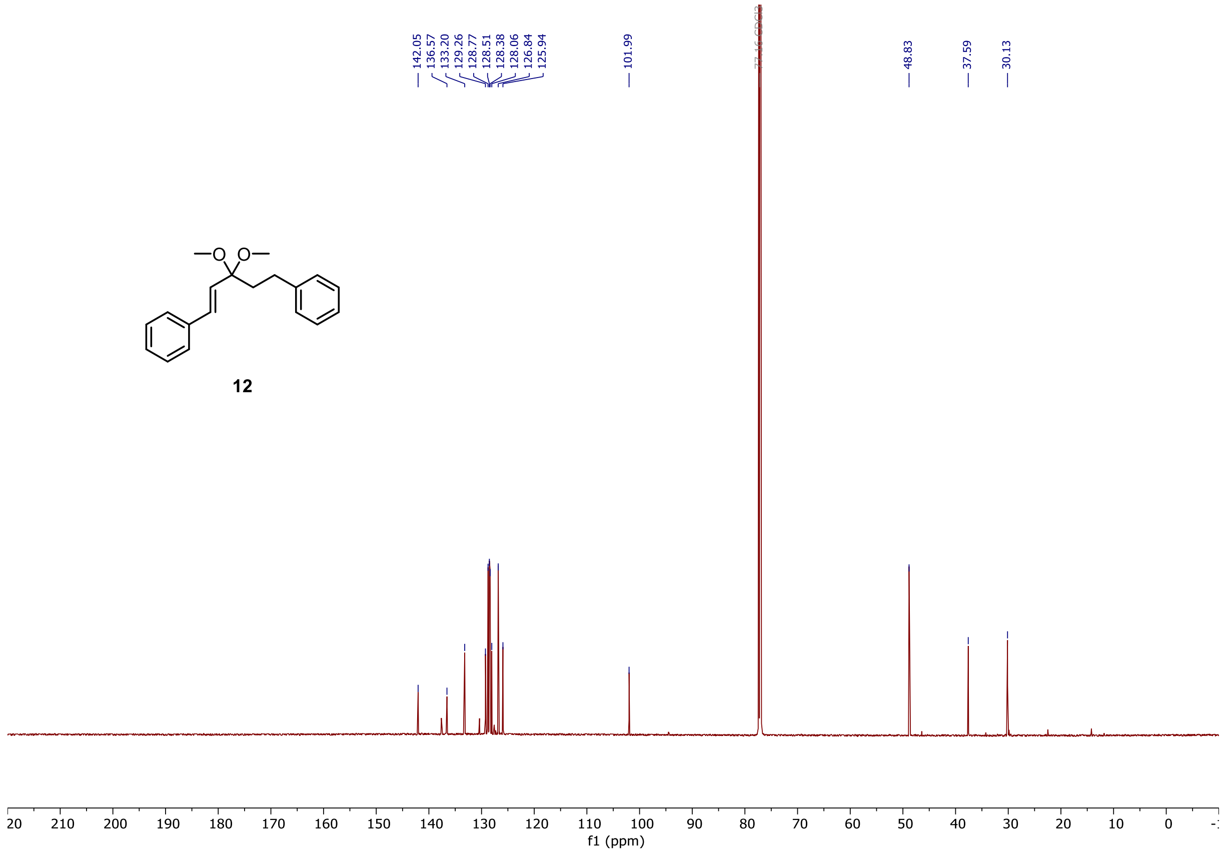

^{1}H NMR (400 MHz, $CDCl_3$) of **14**

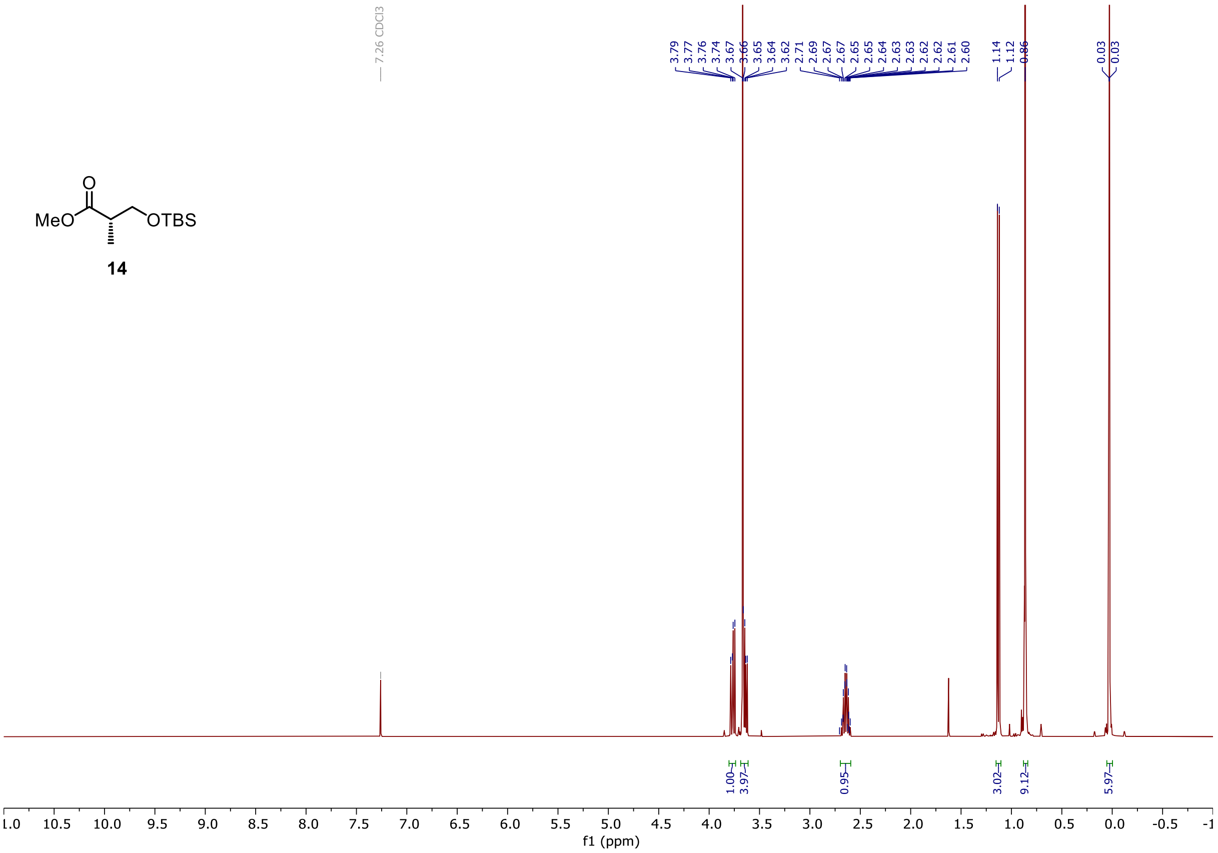

14

^{13}C NMR (101 MHz, $CDCl_3$) of **14**

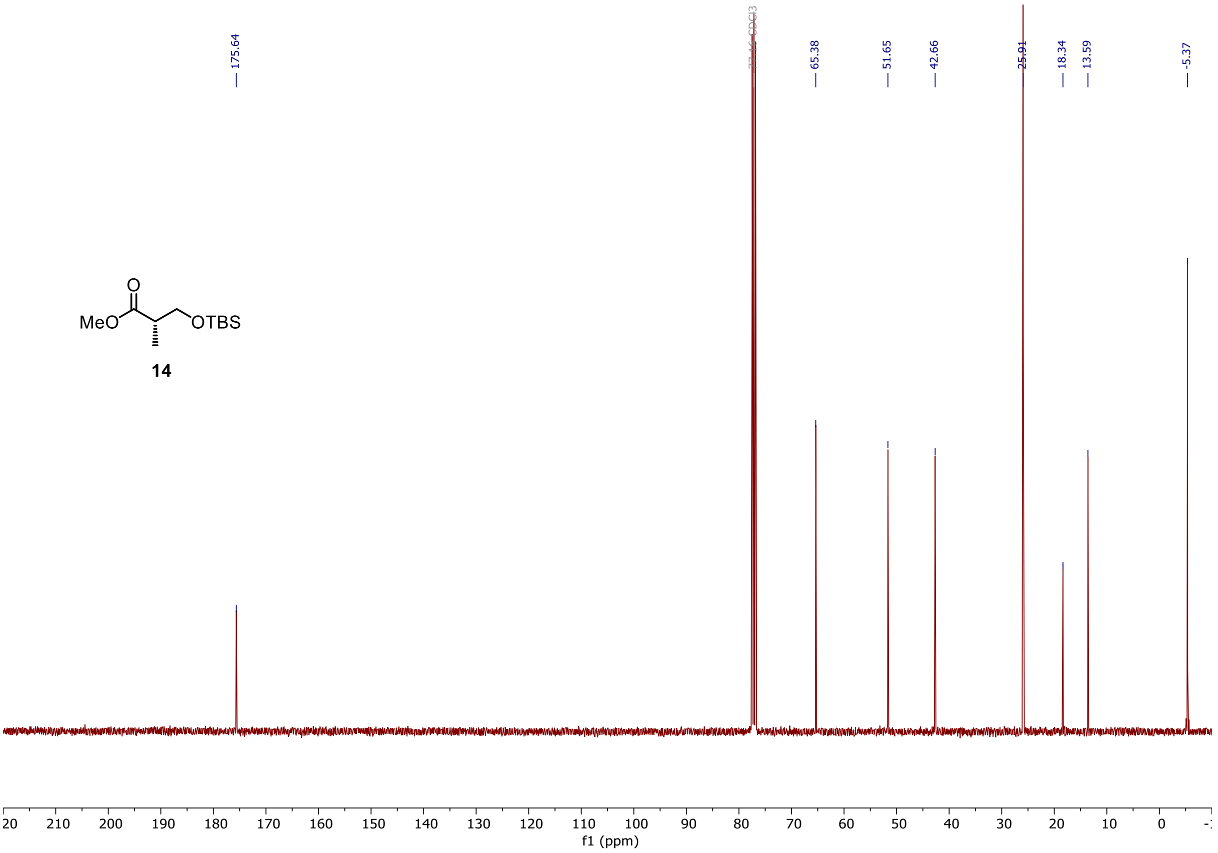

14

^{1}H NMR (400 MHz, $CDCl_3$) of **15**

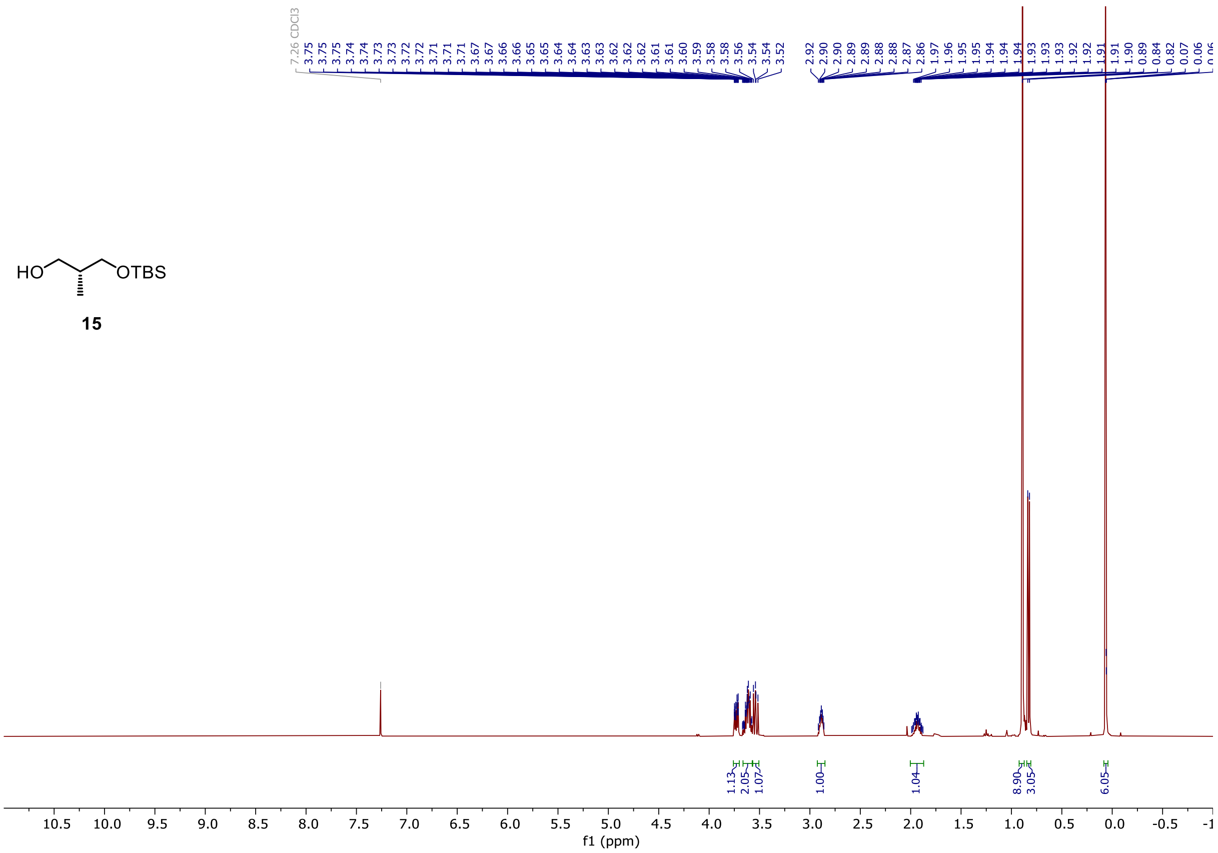

^{13}C NMR (101 MHz, $CDCl_3$) of **15**

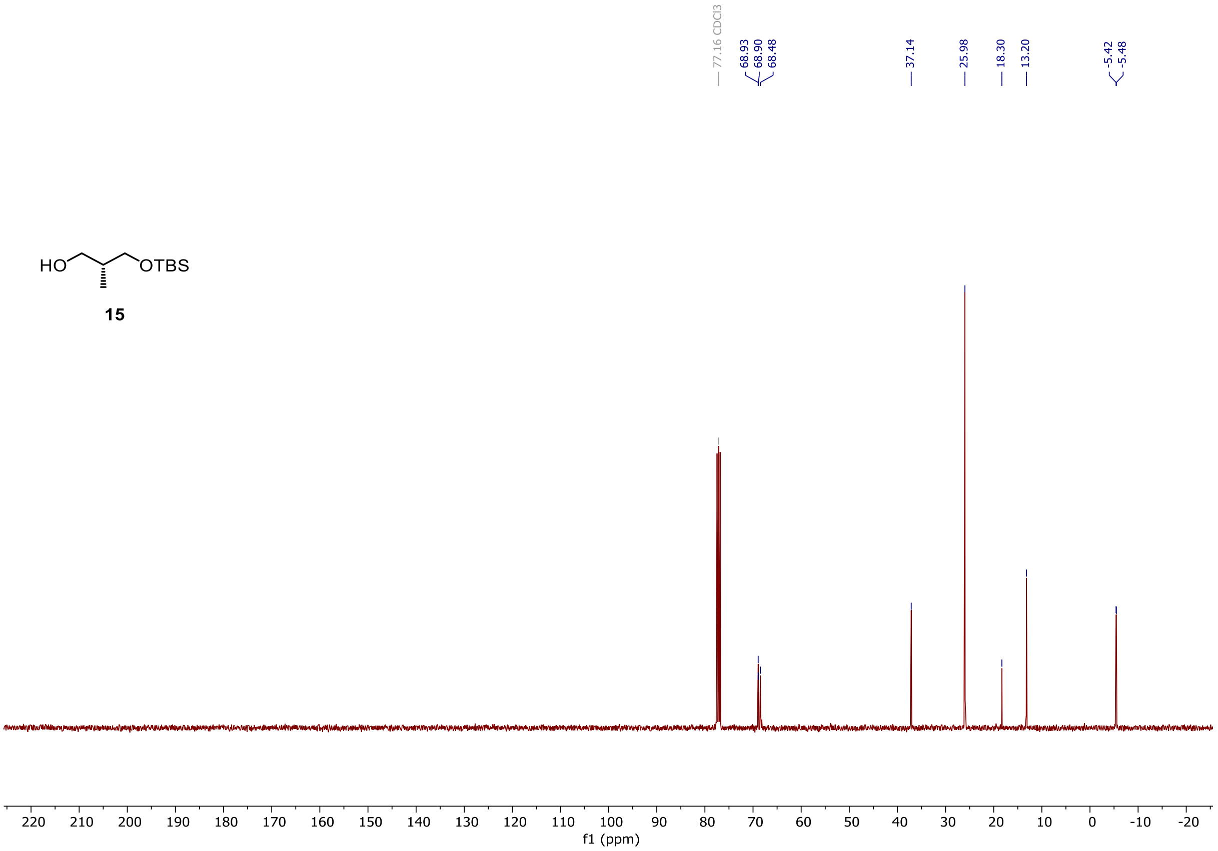

^{1}H NMR (400 MHz, $CDCl_3$) of **16**

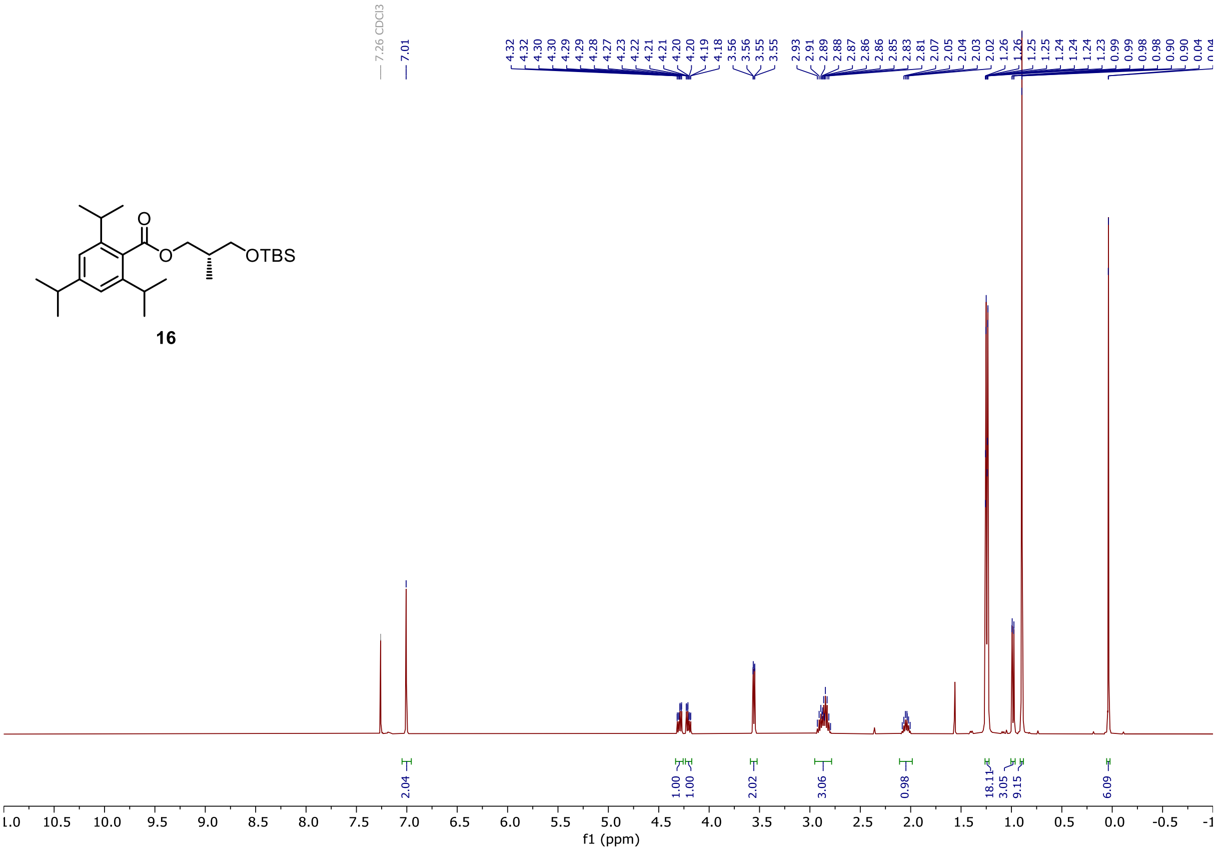

^{13}C NMR (101 MHz, $CDCl_3$) of **16**

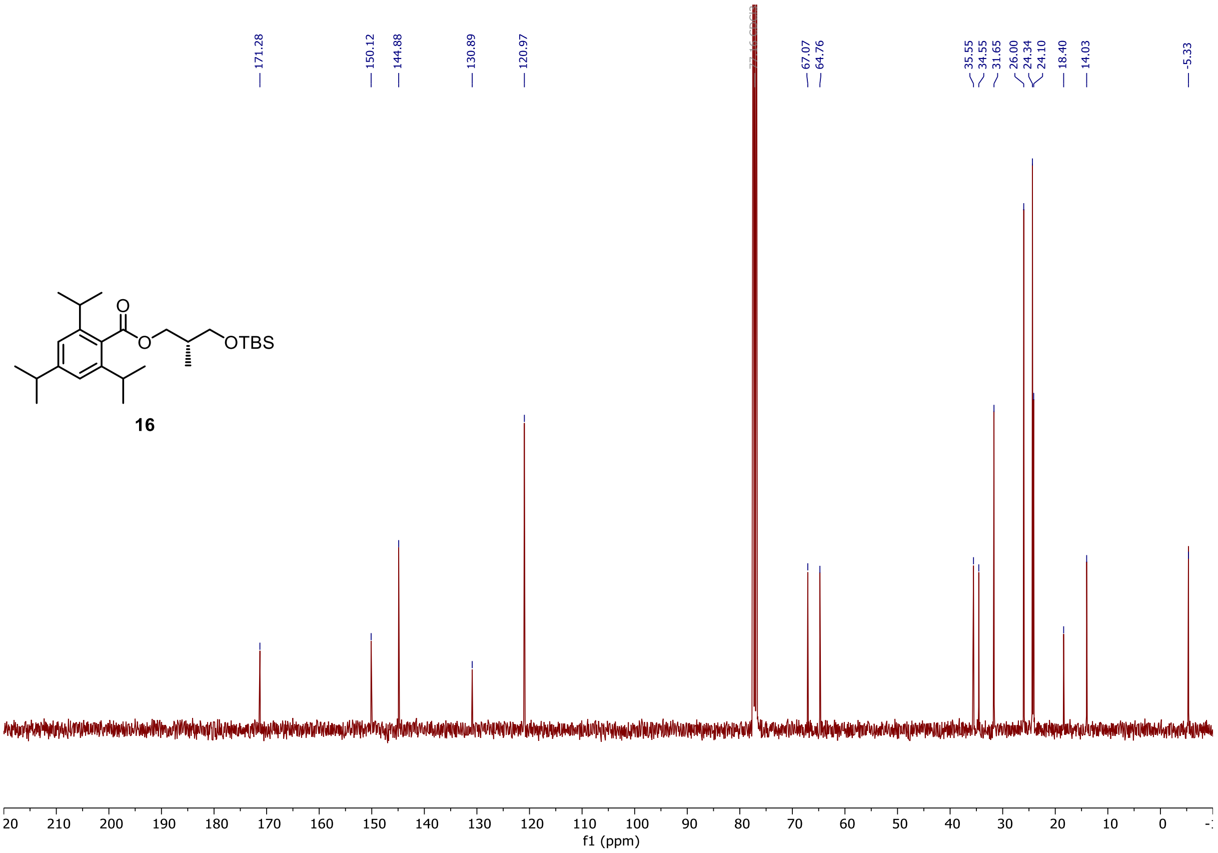

^{1}H NMR (400 MHz, $CDCl_3$) of **17**

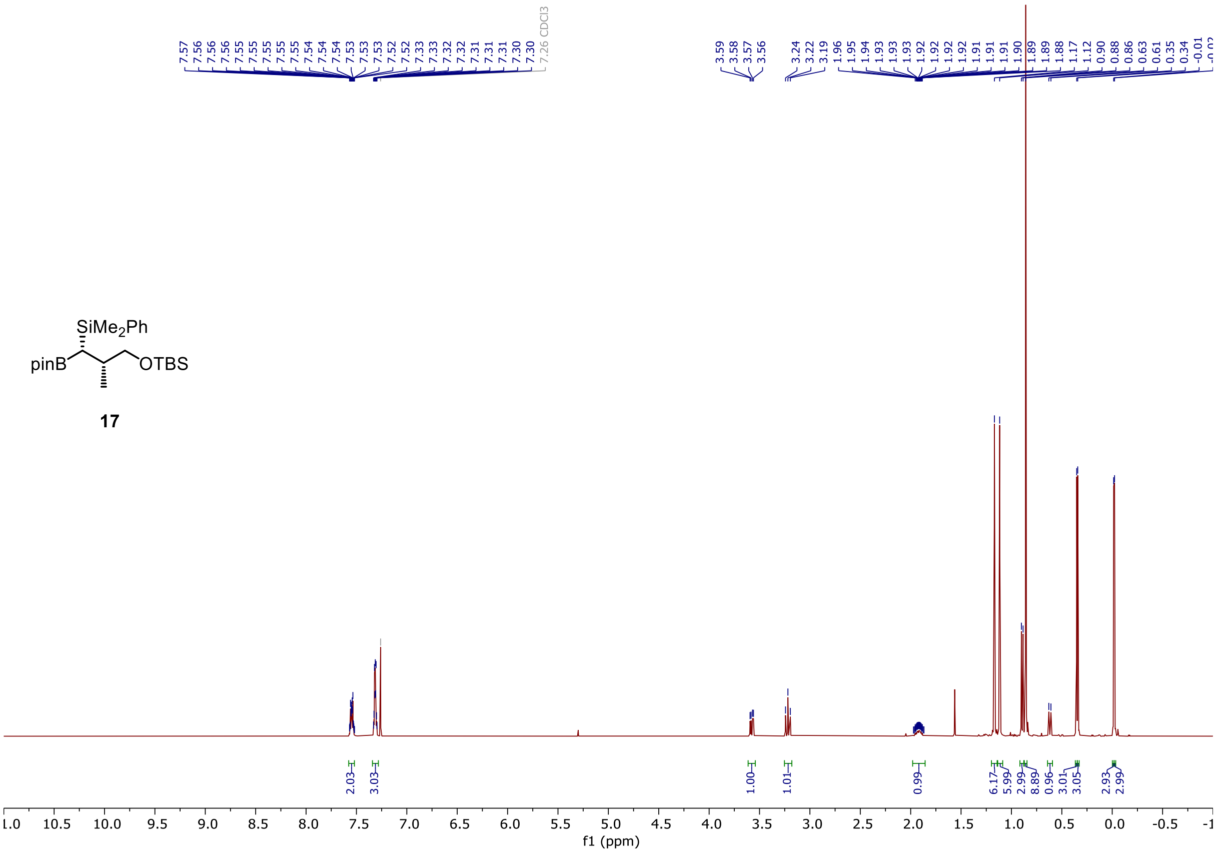

^{13}C NMR (101 MHz, $CDCl_3$) of **17**

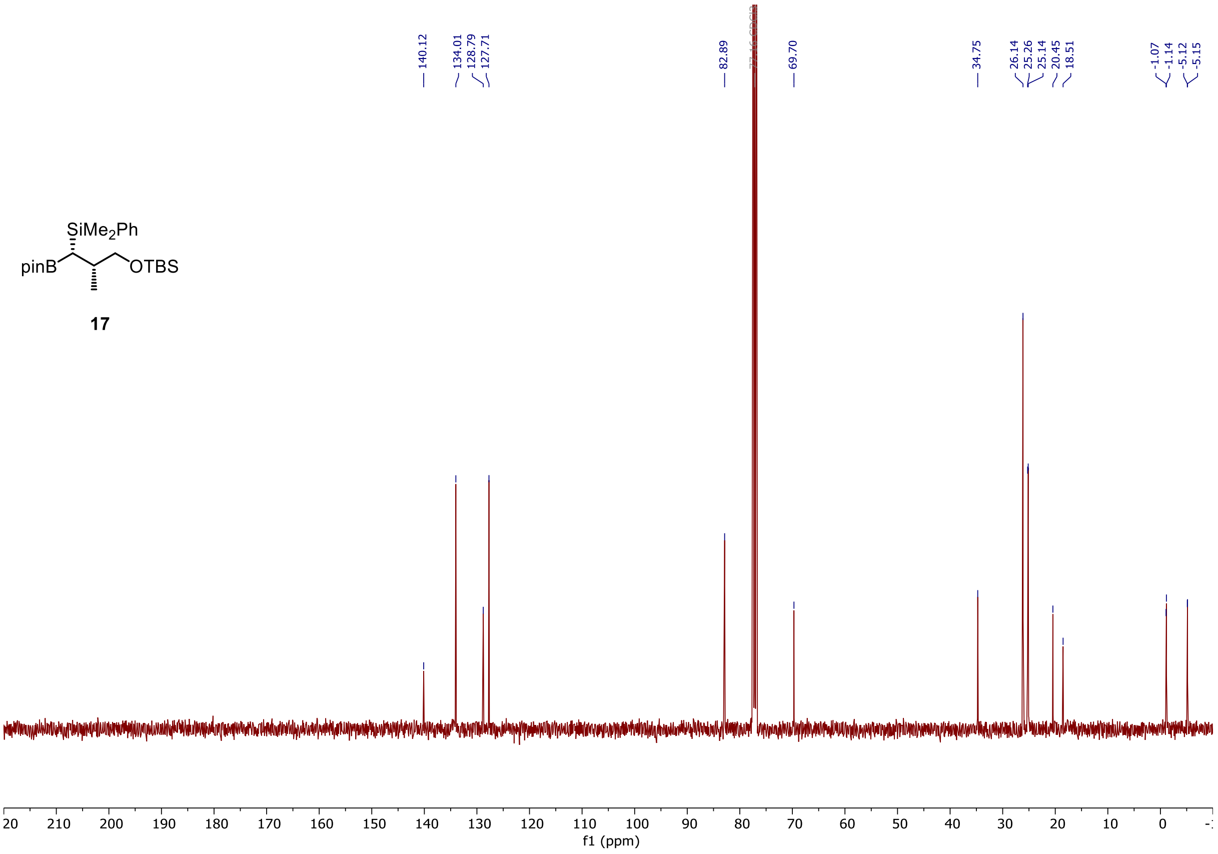

^{1}H NMR (400 MHz, $CDCl_3$) of **18**

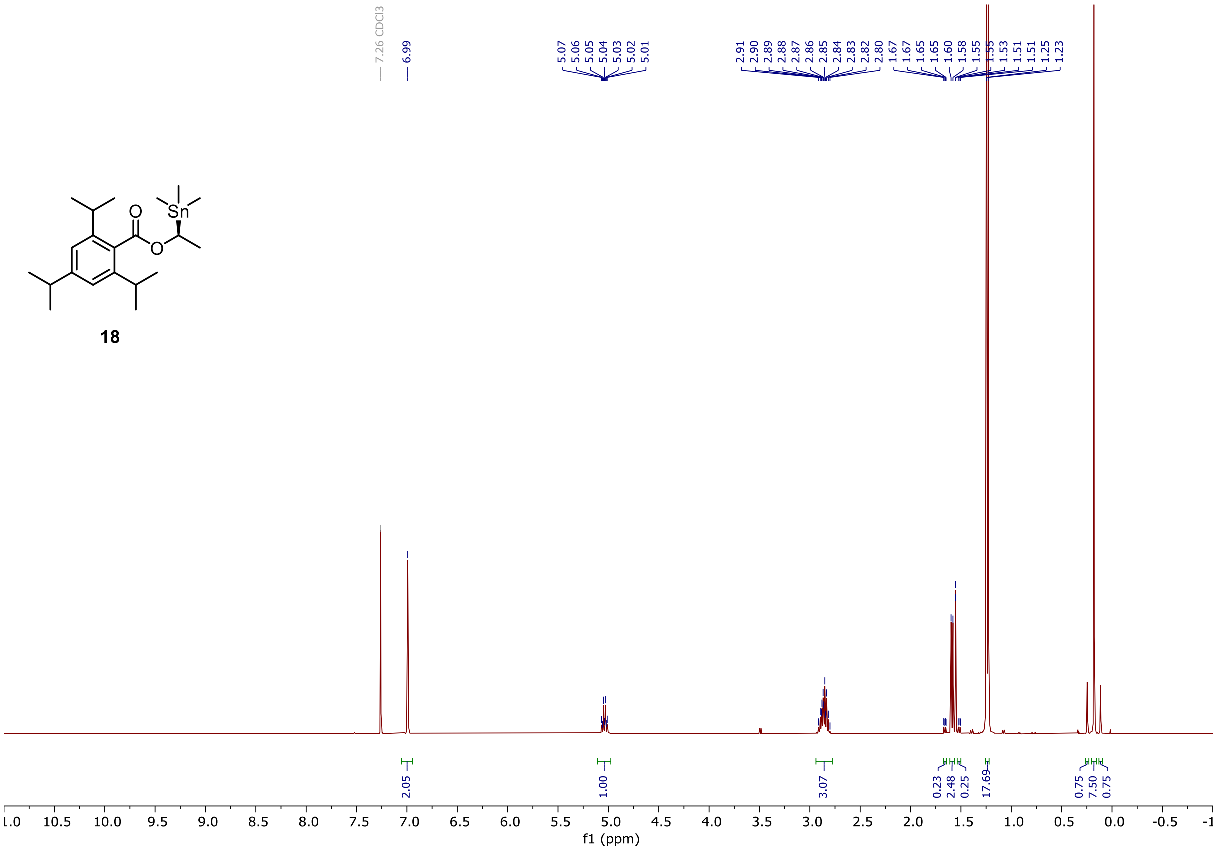

^{13}C NMR (101 MHz, $CDCl_3$) of **18**

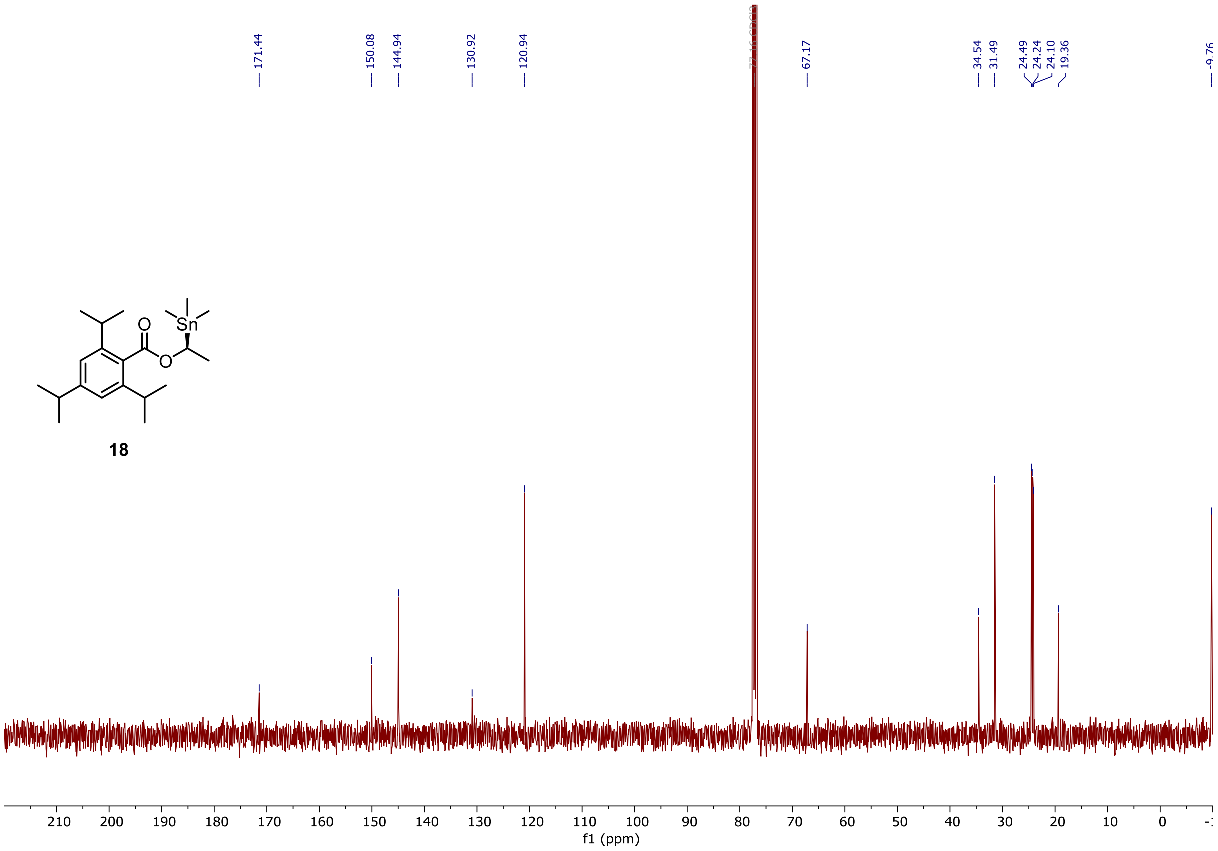

^1H NMR (400 MHz, $CDCl_3$) of **20**

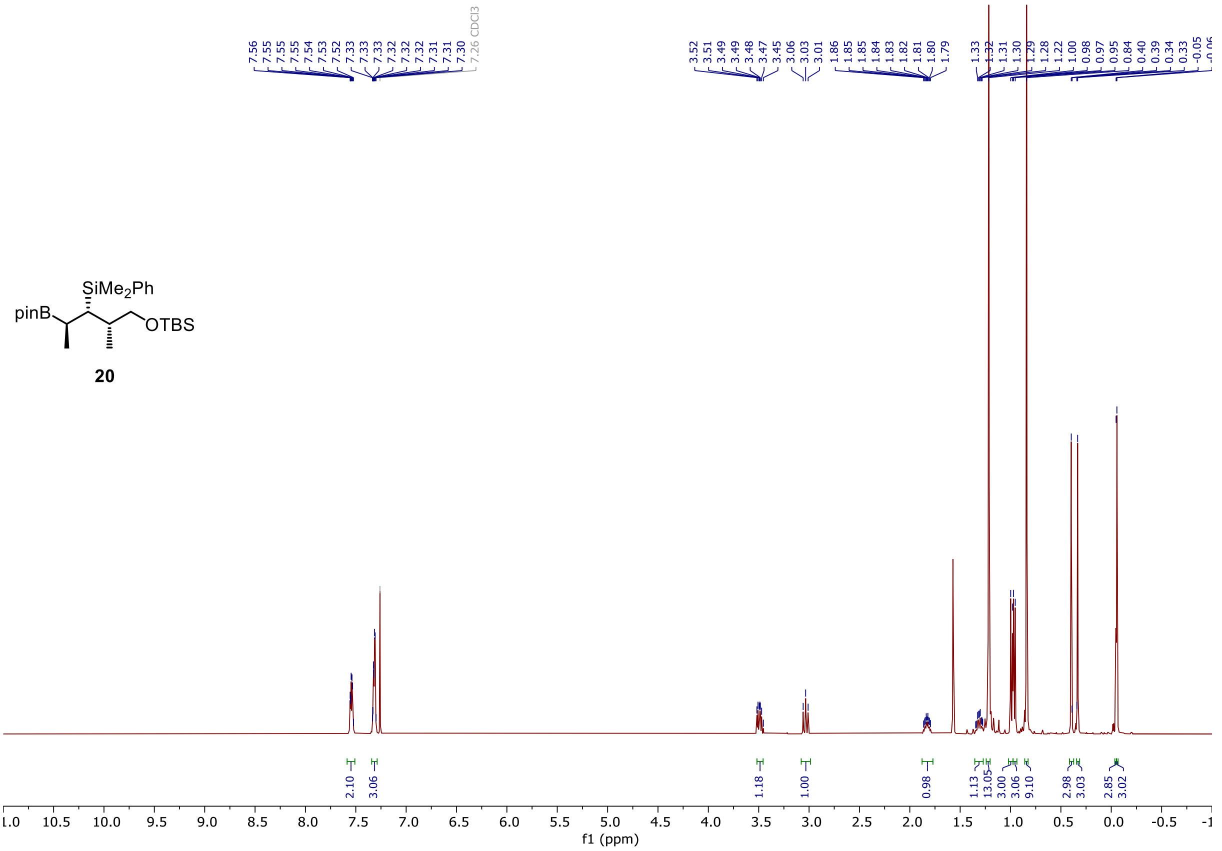

^{13}C NMR (101 MHz, $CDCl_3$) of **20**

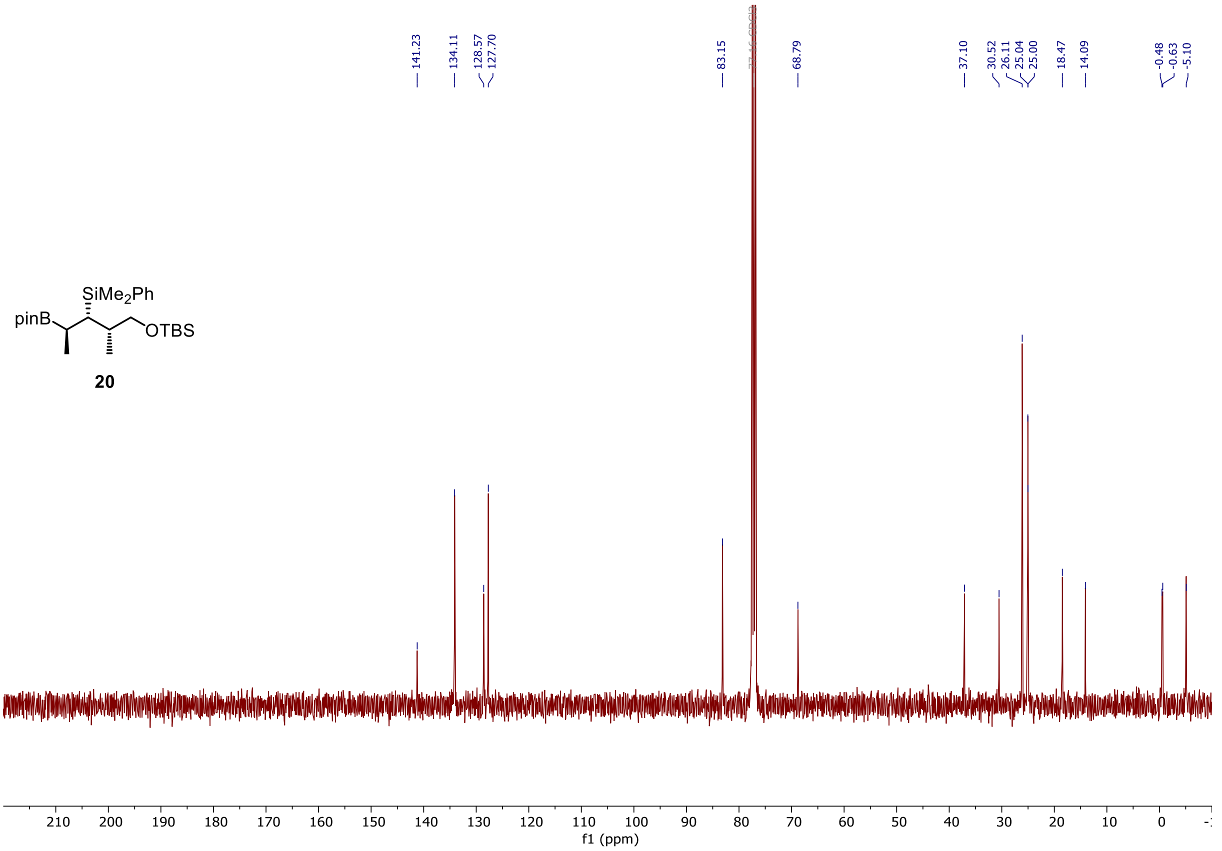

^{1}H NMR (400 MHz, $CDCl_3$) of **22**

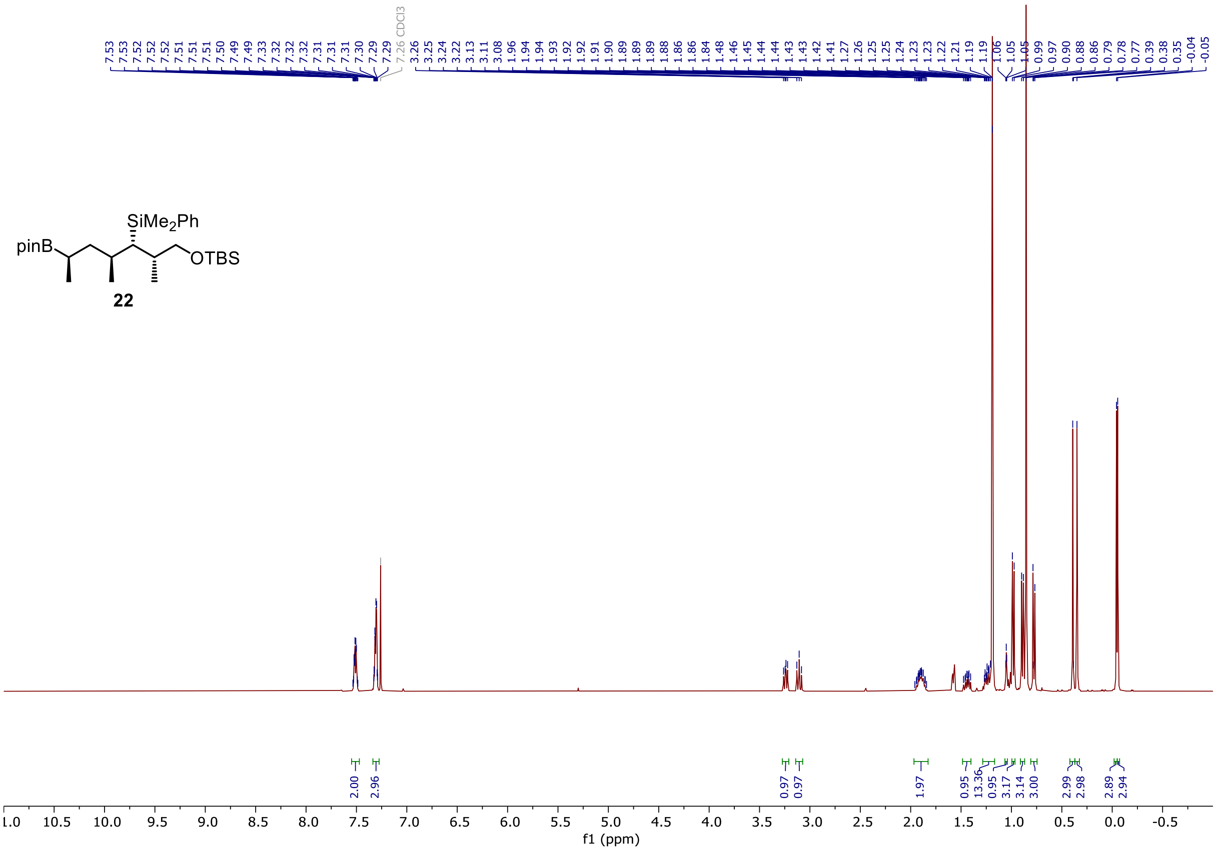

^{13}C NMR (101 MHz, $CDCl_3$) of **22**

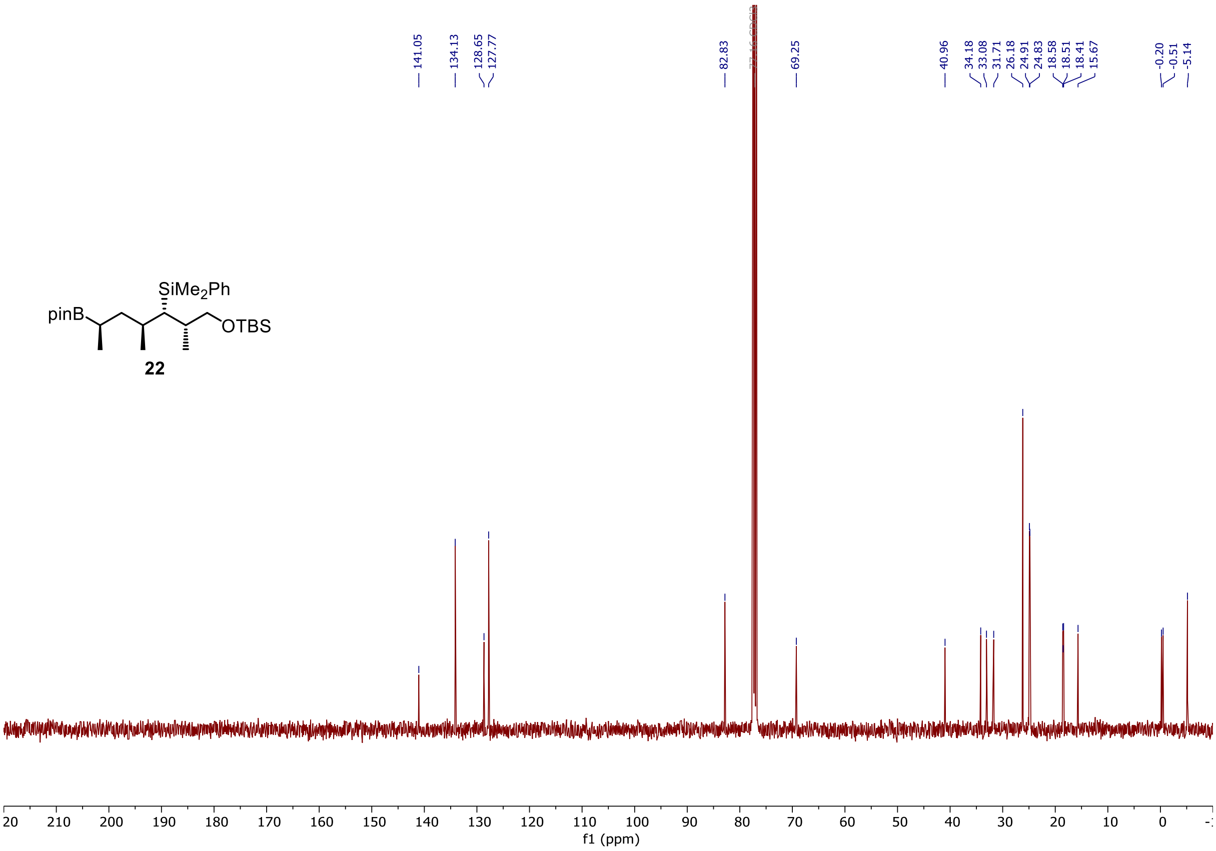

^{1}H NMR (400 MHz, $CDCl_3$) of **24**

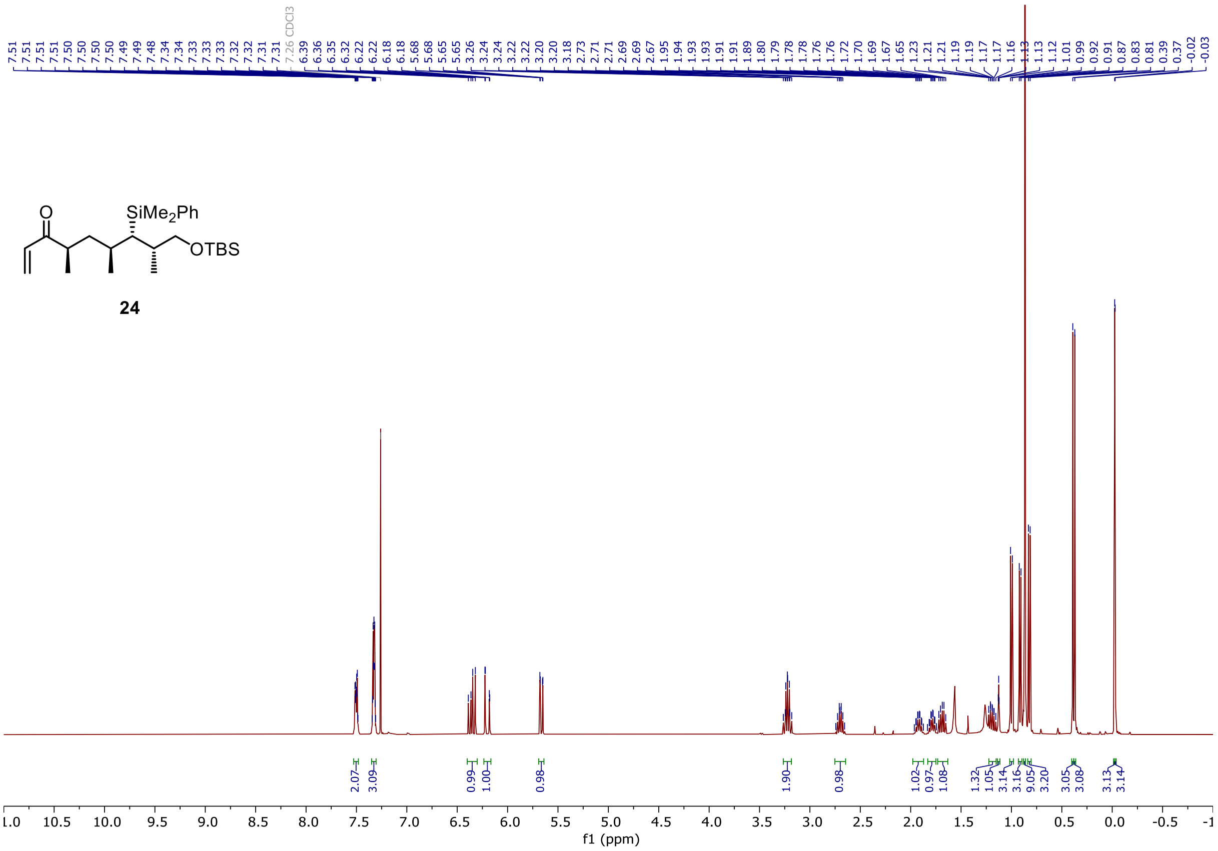

^{13}C NMR (101 MHz, $CDCl_3$) of **24**

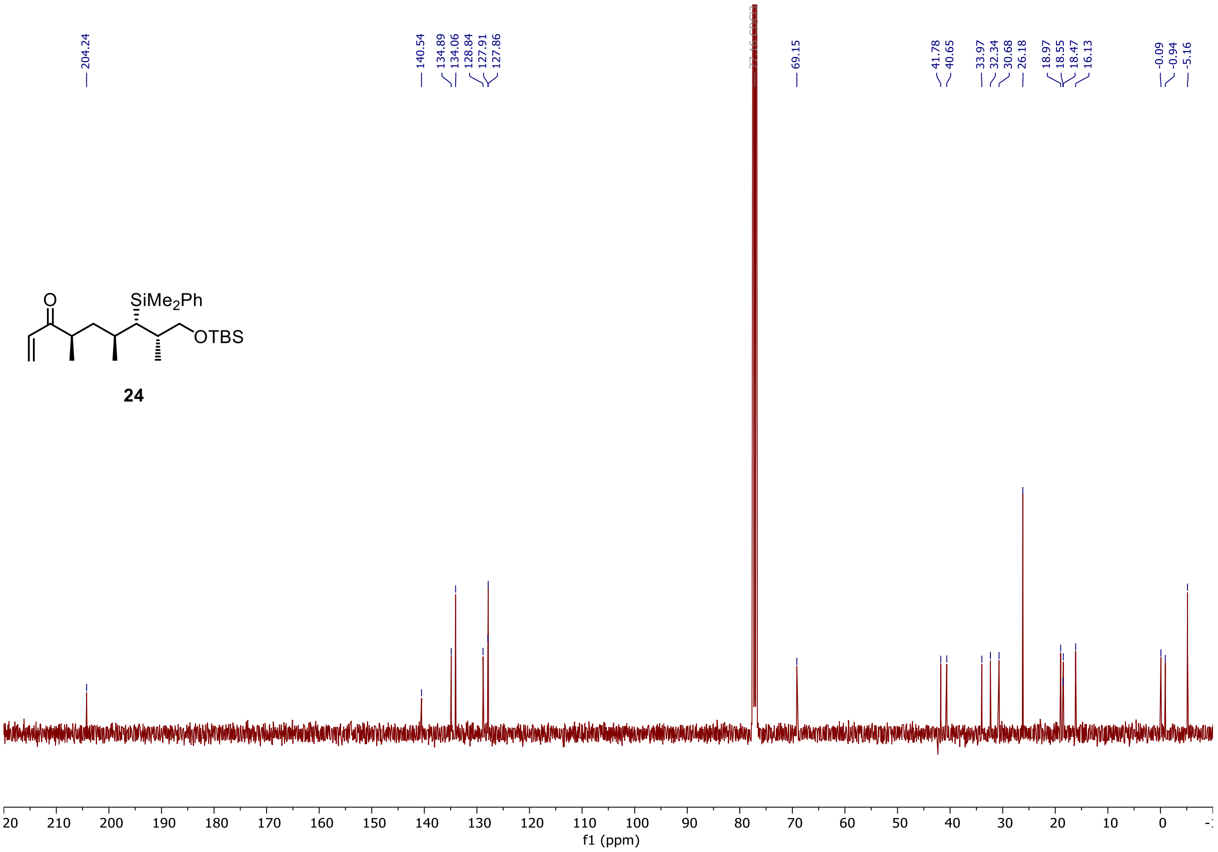

^{1}H NMR (400 MHz, $CDCl_3$) of **25**

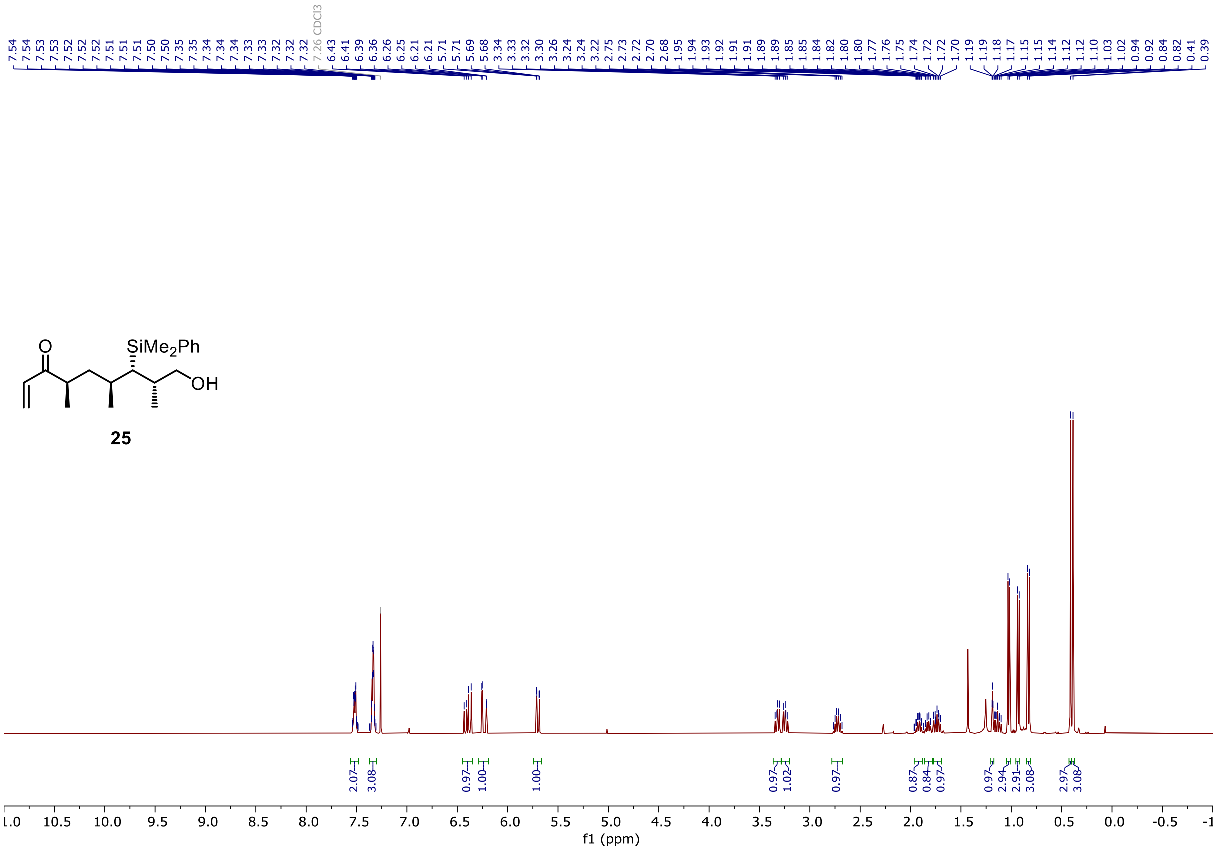

^{13}C NMR (101 MHz, $CDCl_3$) of **25**

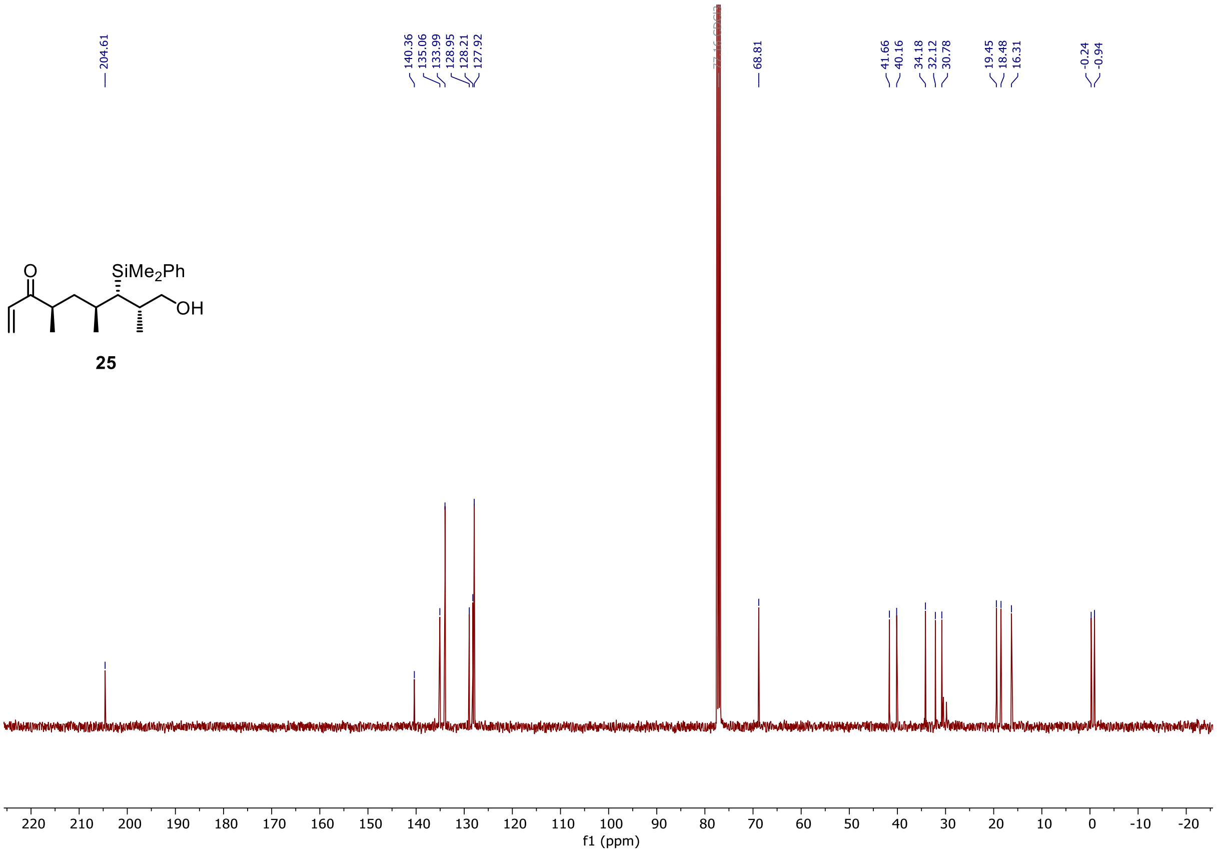

^{1}H NMR (400 MHz, $CDCl_3$) of **26**

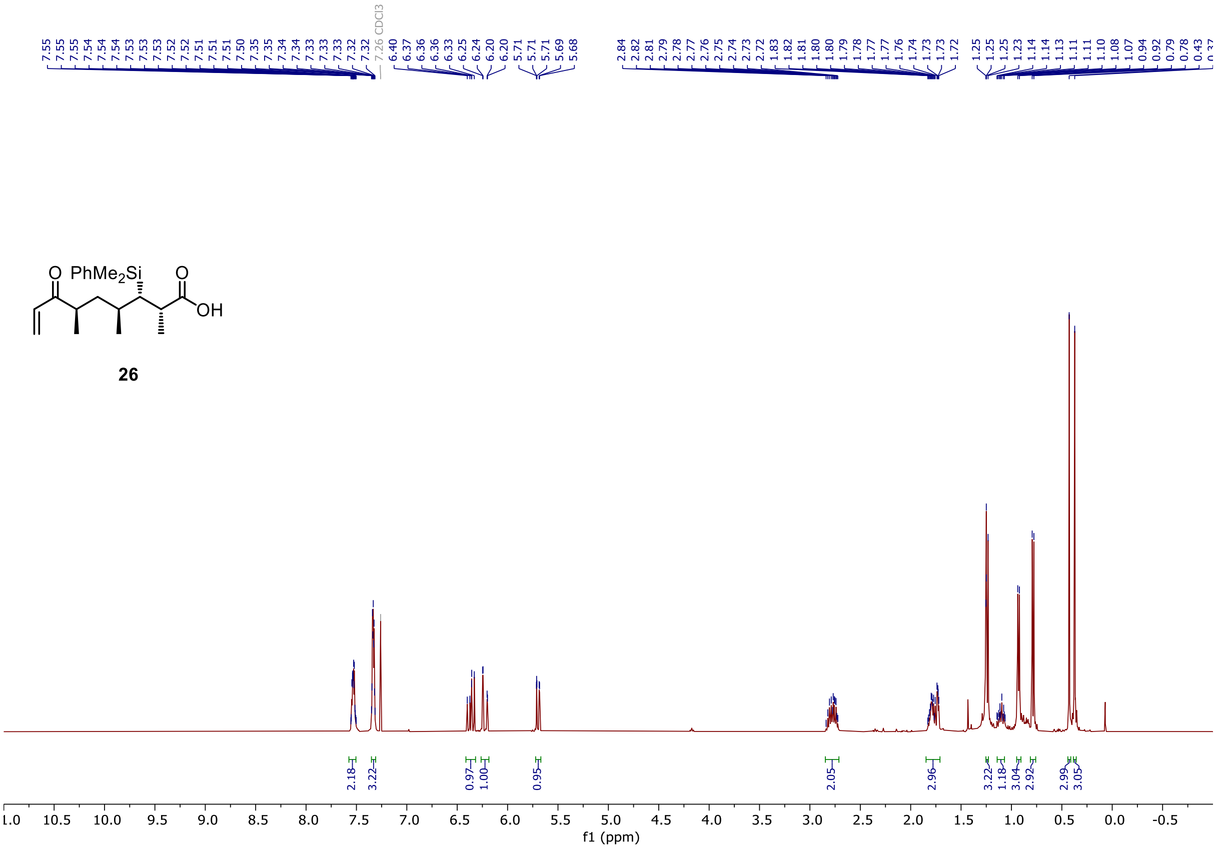

^{13}C NMR (101 MHz, $CDCl_3$) of **26**

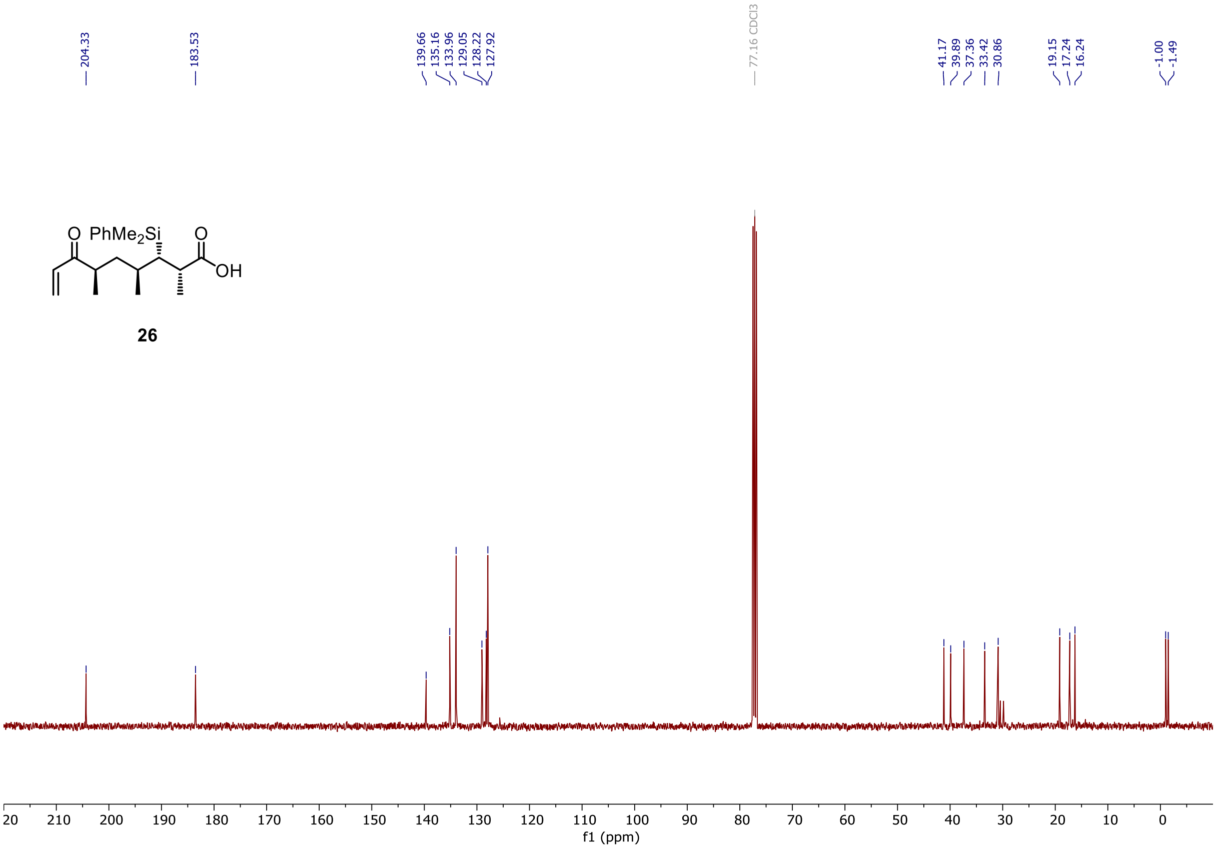

^{1}H NMR (600 MHz, CDCl$_3$) of **28**

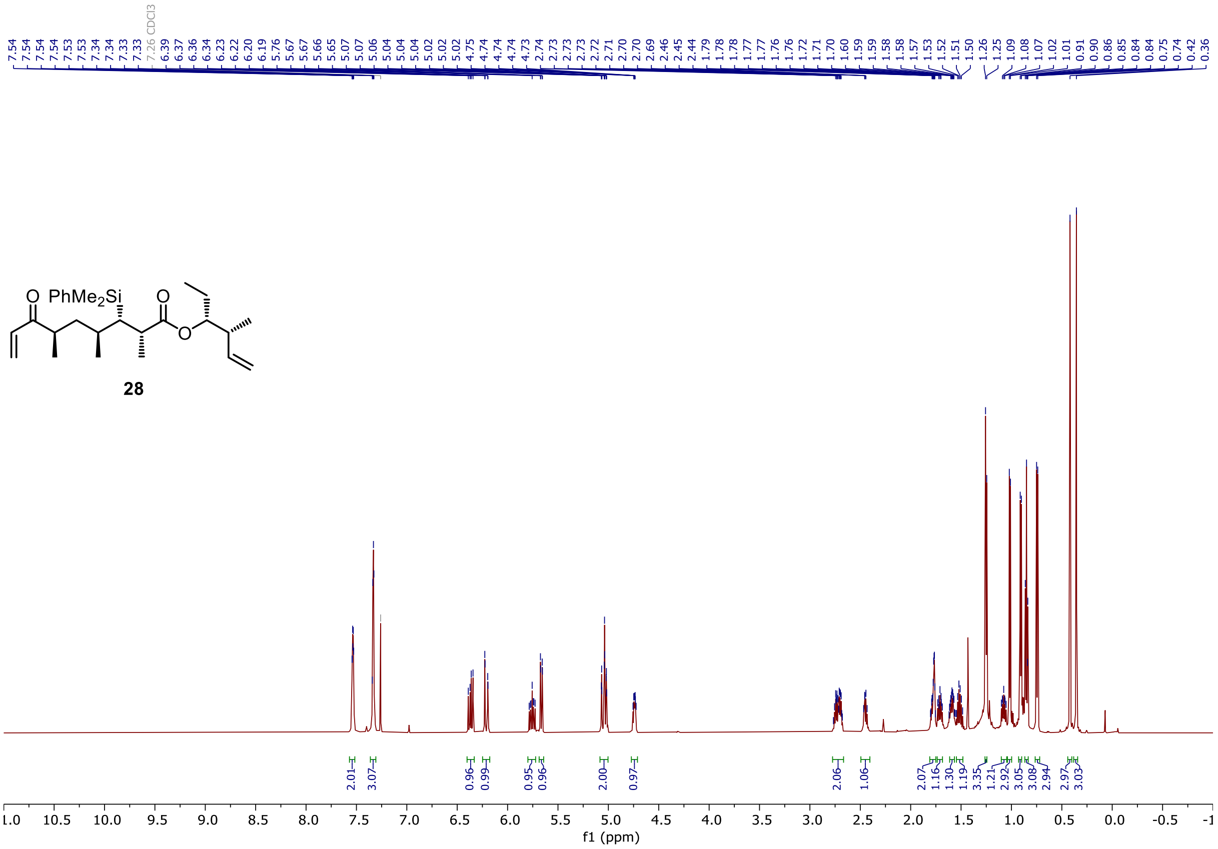

^{13}C NMR (151 MHz, CDCl$_3$) of **28**

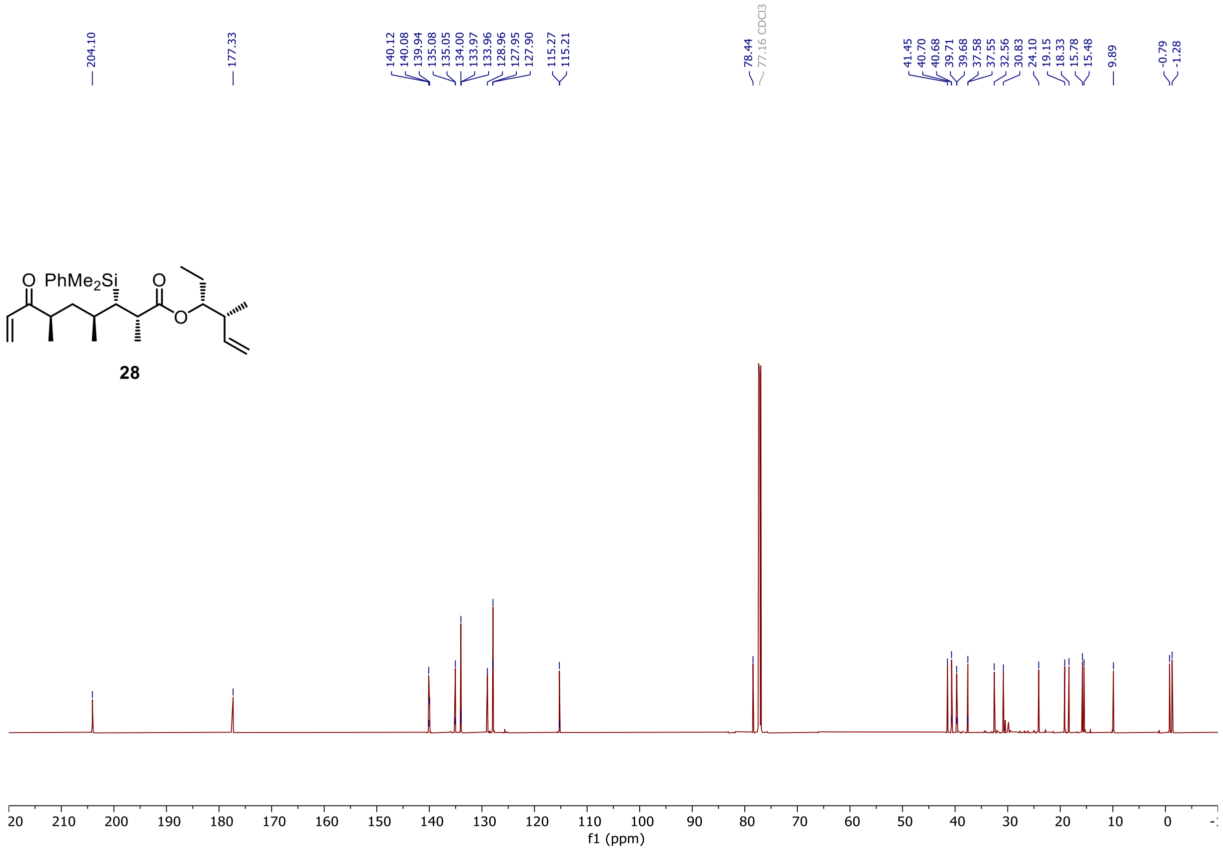

^{1}H NMR (400 MHz, $CDCl_3$) of **29**

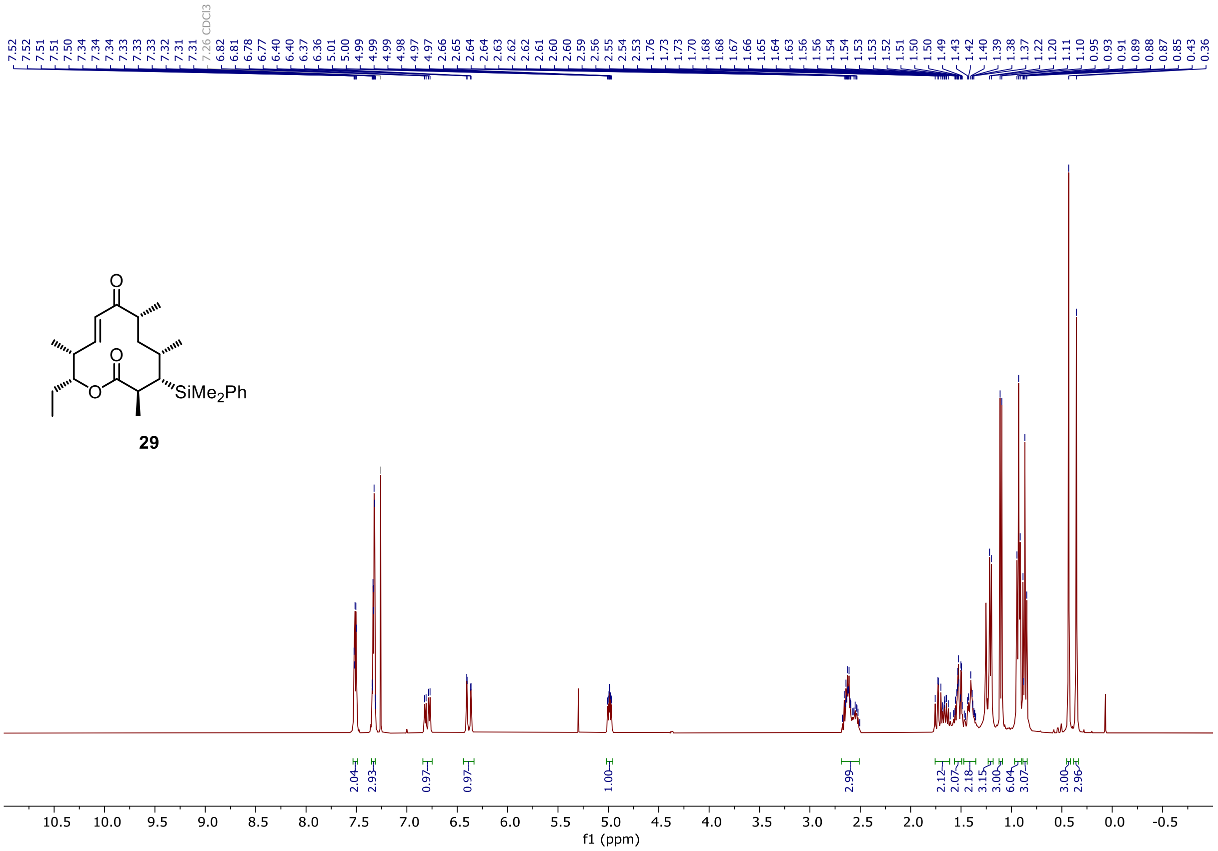

^{13}C NMR (101 MHz, $CDCl_3$) of **29**

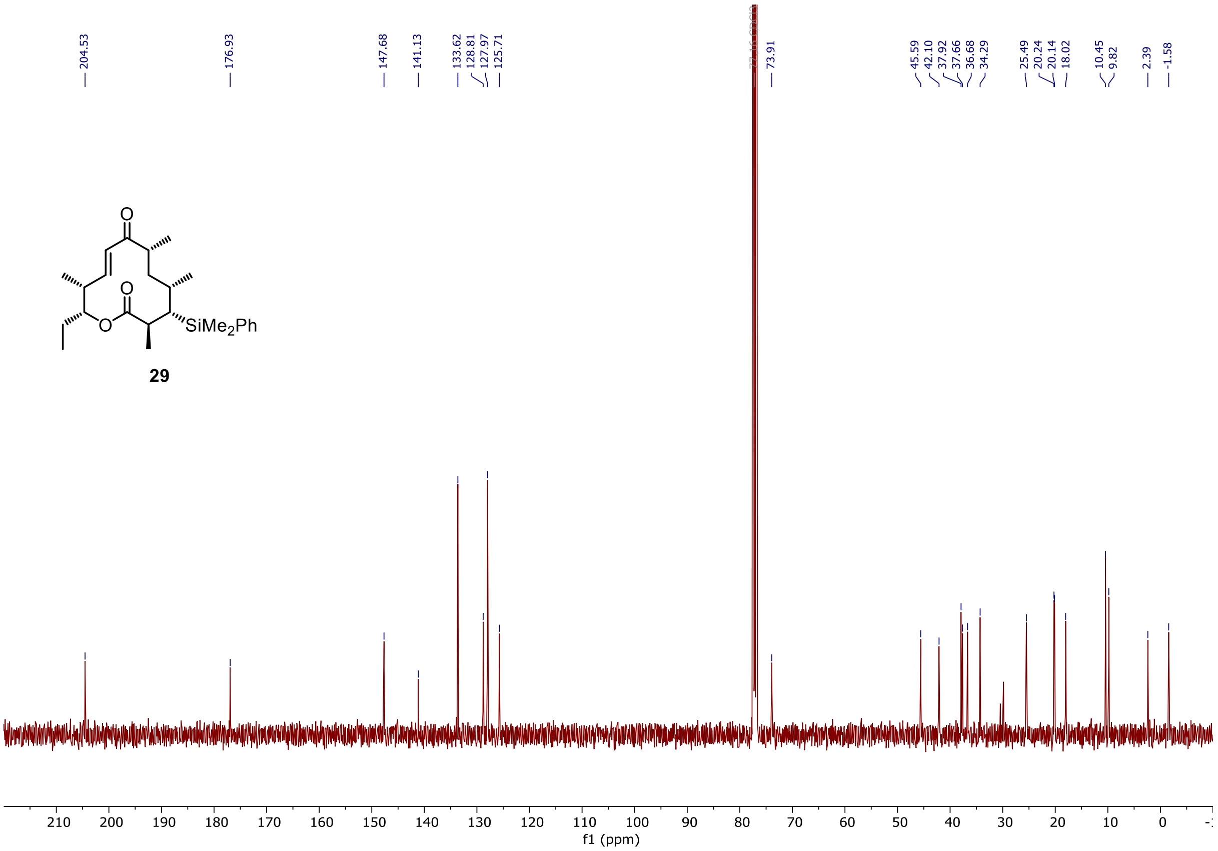

^{1}H NMR (600 MHz, $CDCl_3$) of **30**

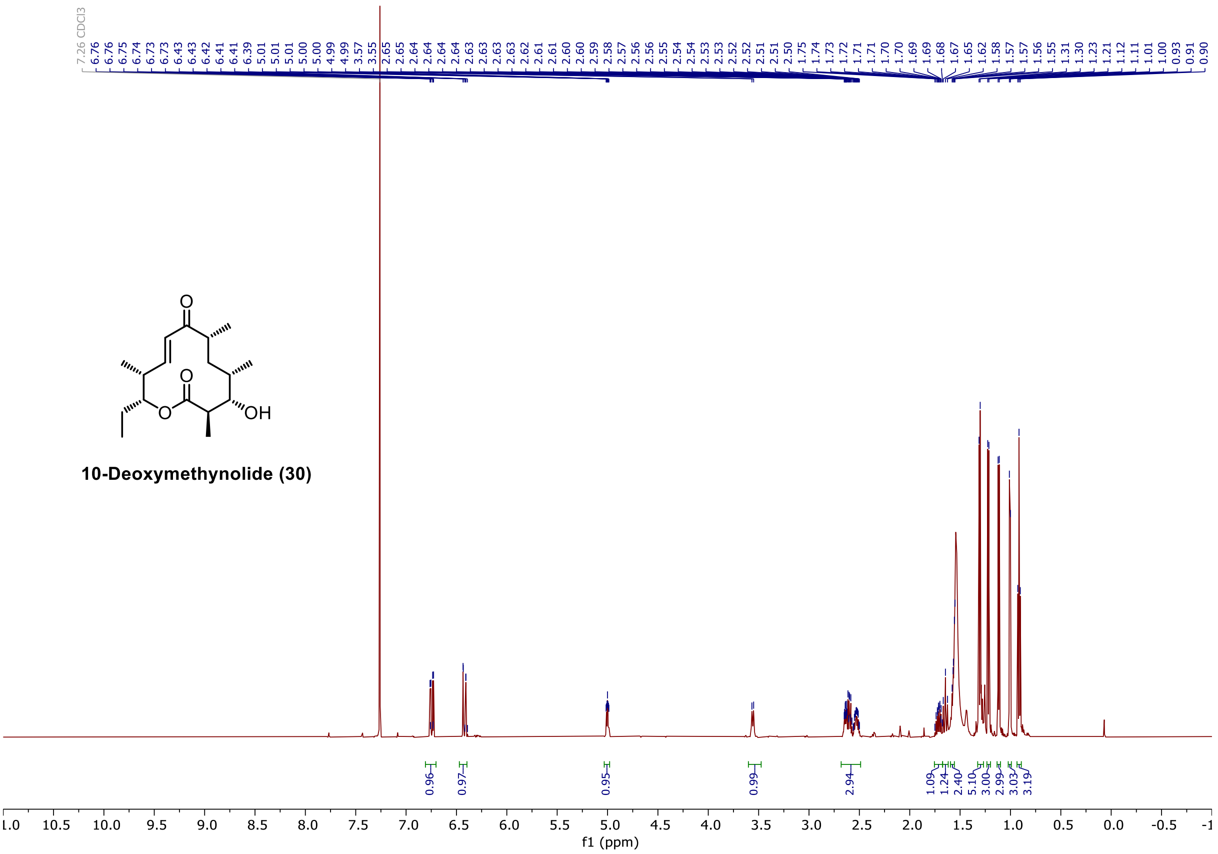

^{13}C NMR (151 MHz, $CDCl_3$) of **30**

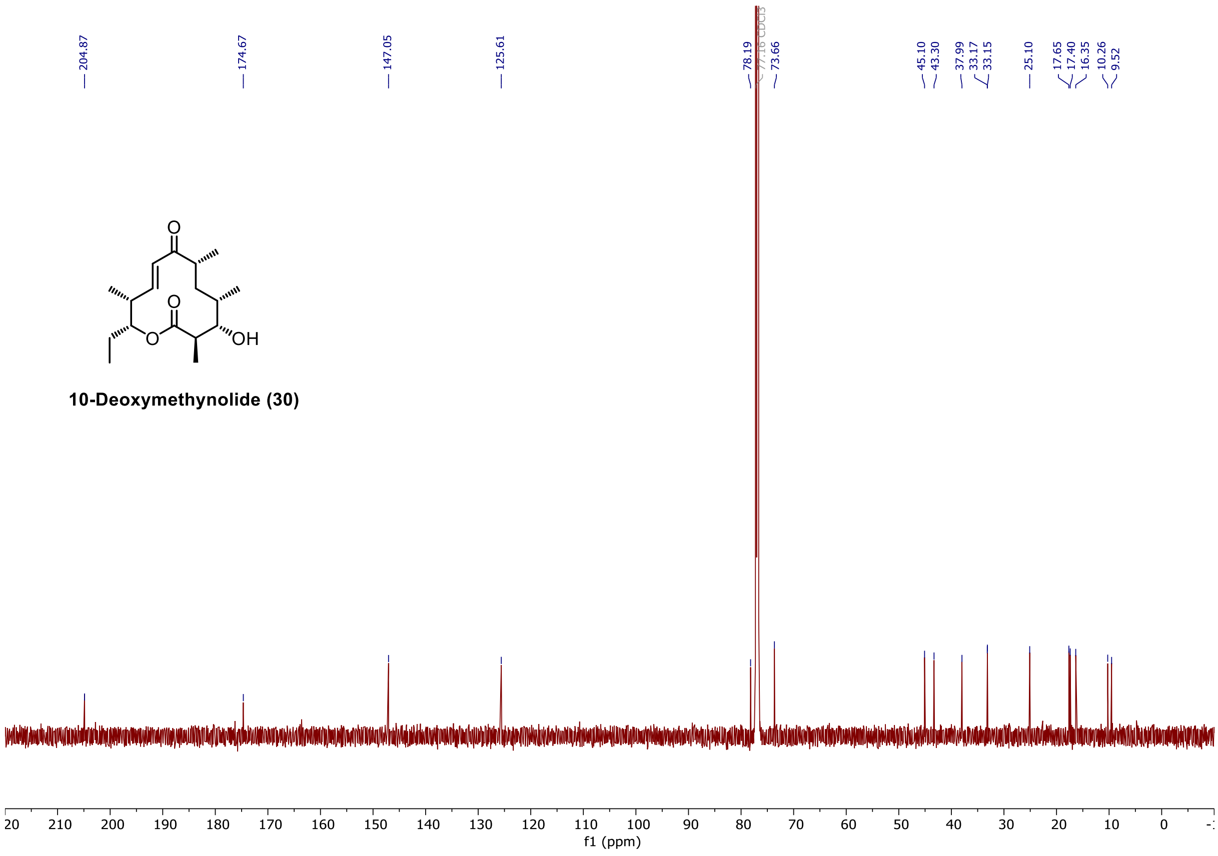

Supplement: Supplementary file 1 — Supporting Information [file ANIE-62-0-s001.pdf]
